# Supplementary material for: Global proteomic analysis of Cryptococcus neoformans clinical strains reveals significant differences between latent and lethal infection
Source: mSystems. 2025 Sep 25;10(10):e00751-25. doi: 10.1128/msystems.00751-25 (PMC12542649; doi:10.1128/msystems.00751-25)
Supplement: Supplemental Material — Figures S1 to S3 and captions for supplemental tables. [file msystems.00751-25-s0001.docx]

**
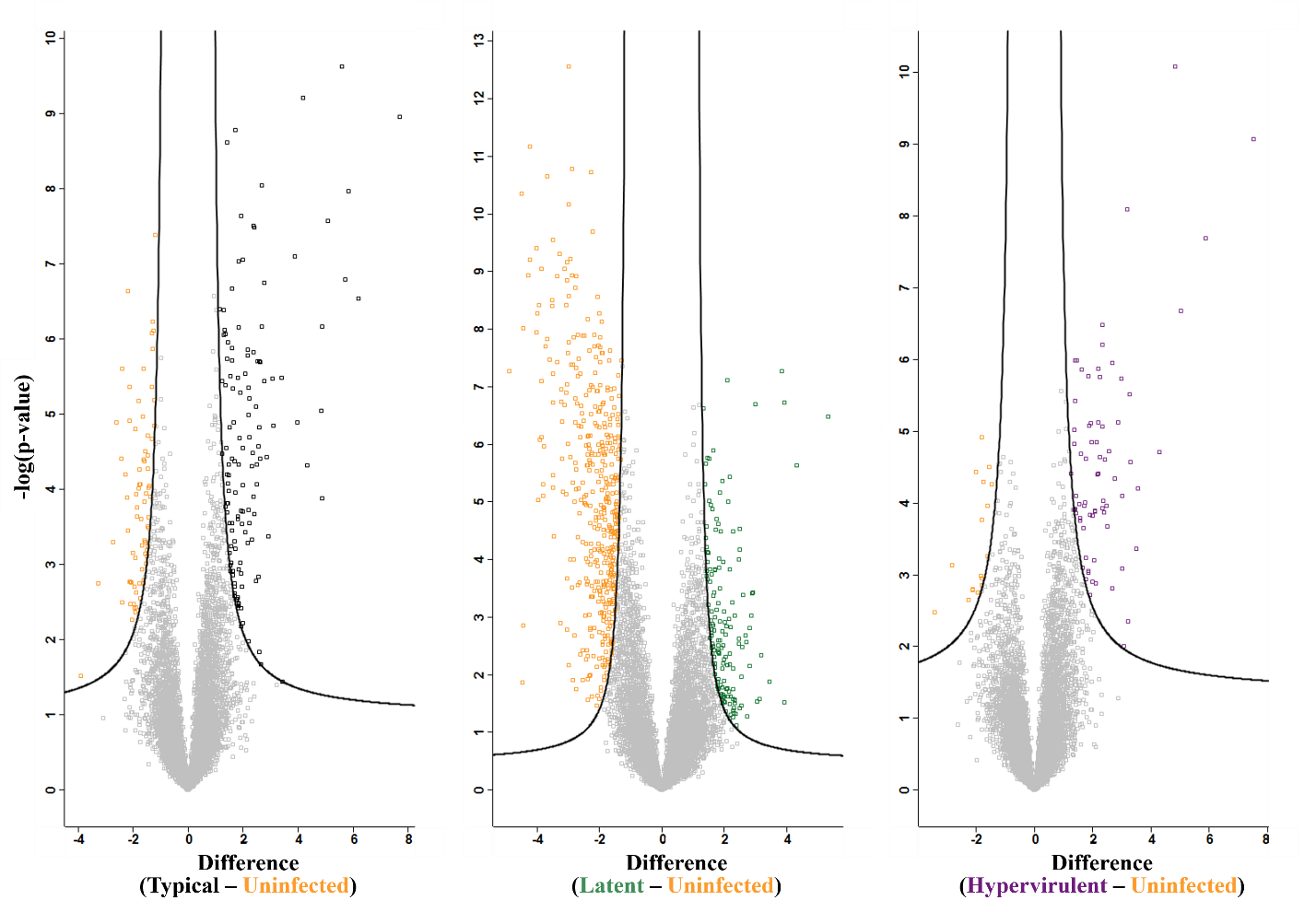
**

**Supplemental Fig. 1: Significant protein identifications from infected versus uninfected mouse proteomes.** Volcano plots were generated to visualize significantly different proteins from Student’s t-test with multiple comparison correction between latent (green), typical (black), or hypervirulent (purple) at 14 days post-infection versus uninfected (orange) mice. Fudge factor (s0) was tuned using the siggenes R package (fudge2()) for each comparison (Left s0 = 0.52, Middle s0 = 1.22, Right s0 = 0.37). Significance was determined at an FDR < 0.05.

**[see Table 1]**

**Supplemental Table 1: Significant mouse protein identifications.** Mice infected with either typical (KN99α), latent (UgCl223), or hypervirulent (UgCl422) *C. neoformans* infections at 14 days post-infection had their lung proteomes compared to each other or uninfected mice. Tables were generated from the identified significant proteins for each Student’s t-test with multiple comparison correction.


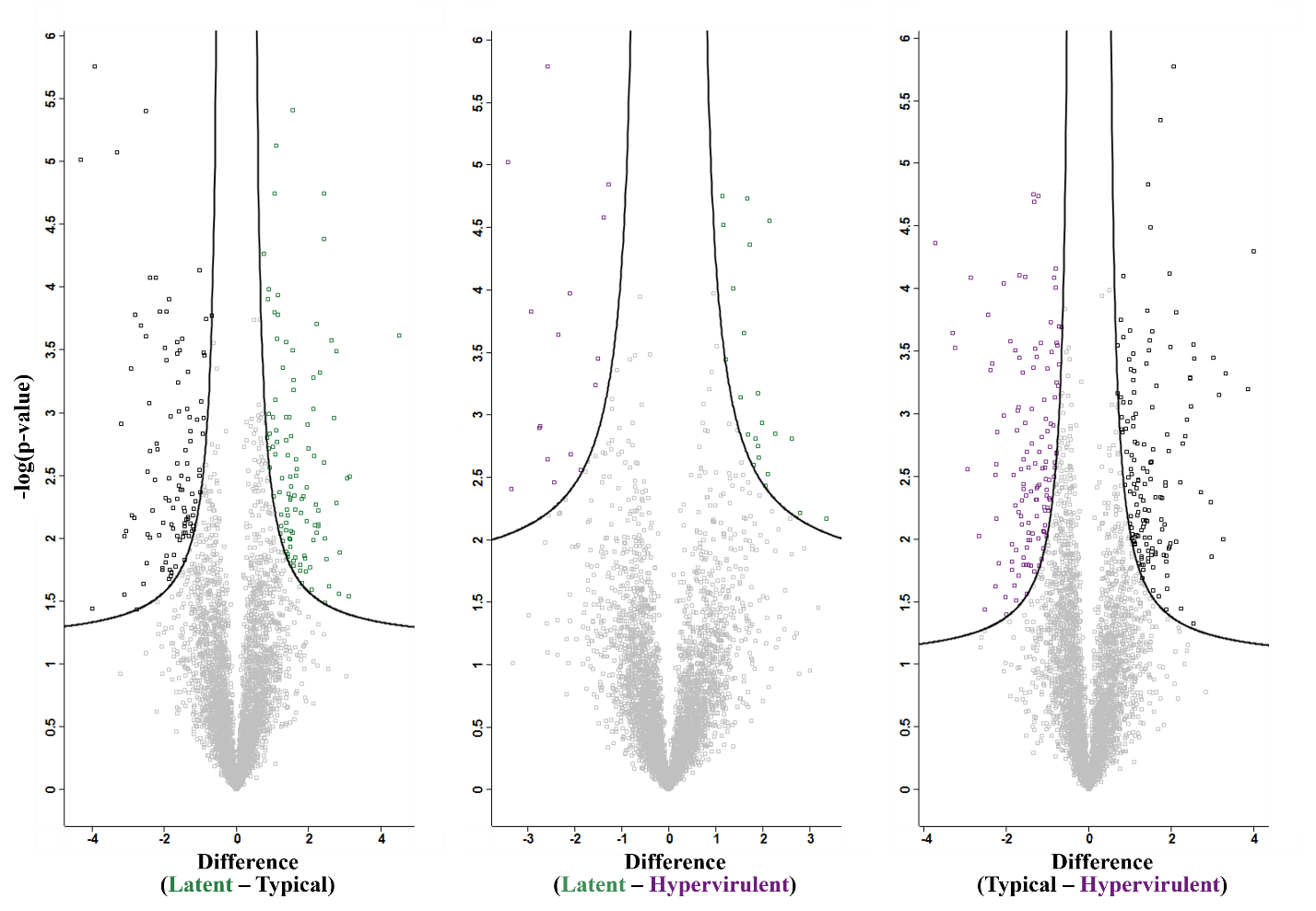


**Supplemental Fig. 2: Significant protein identifications from *C. neoformans* strain *in vitro* culture proteome comparisons.** Volcano plots were generated to identify significantly different protein abundances from Student’s t-test with multiple comparison correction of latent (green), typical (black), or hypervirulent (purple) *C. neoformans* cultures *in vitro*. Fudge factor (s0) was tuned using the siggenes R package (fudge2()) for each comparison (Left s0 = 0.23, Middle s0 = 0.23, Right s0 = 0.25). Significance was determined at an FDR < 0.05.

**[see Table 2]**

**Supplemental Table 2: Significant *C. neoformans* culture protein identifications.** Cultures of typical (KN99α), latent (UgCl223), and hypervirulent (UgCl422) *C. neoformans* strains were grown and processed for LC-MS/MS proteomics. Culture proteomes were compared to each other and significant proteins identified from Student’s t-test with multiple comparison correction statistical analyses. Tables were generated from the identified significant proteins for each comparison.


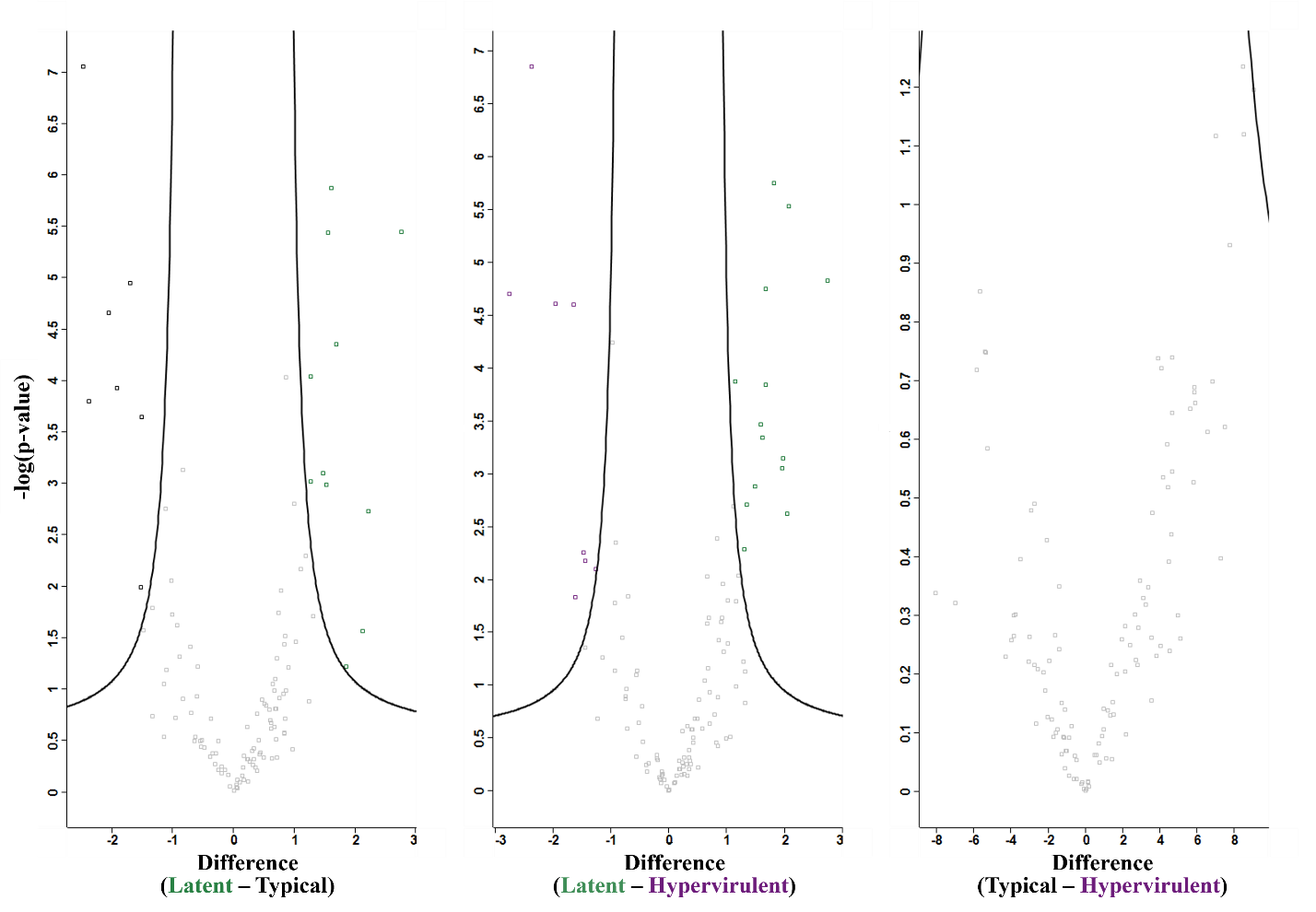


**Supplemental Fig. 3: Significant protein identifications of *C. neoformans* proteins from infected lungs.** Volcano plots were generated to identify significantly different proteins from Student’s t-test with multiple comparison correction between typical (black), latent (green), or hypervirulent (purple) *in vivo* *C. neoformans* proteomes at 14 days post-infection. The comparison of typical versus hypervirulent strains showed no differences in *C. neoformans* proteins. Fudge factor (s0) was tuned using the siggenes R package (fudge2()) for each comparison (Left s0 = 0.86, Middle s0 = 0.86, Right s0 = 0.86). Significance was determined at an FDR < 0.05.

**[See Table 3]**

**Supplemental Table 3: Significant *C. neoformans* infection-derived protein identifications.** *C. neoformans* proteome profiles were assessed from the lungs of mice infected with either typical (KN99α), latent (UgCl223), or hypervirulent (UgCl422) strains. *Cryptococcus* proteomes were then compared to each other and significant proteins identified by Student’s t-test with multiple comparison correction. Tables were generated from the identified significant proteins for each comparison.
