# Supplementary material for: Global proteomic analysis of Cryptococcus neoformans clinical strains reveals significant differences between latent and lethal infection
Source: mSystems. 2025 Sep 25;10(10):e00751-25. doi: 10.1128/msystems.00751-25 (PMC12542649; doi:10.1128/msystems.00751-25)
Supplement: Supplemental Tables — Tables S1 to S3. [file msystems.00751-25-s0002.pdf]

Supplemental Table 1: Significant mouse protein identifications. Mice infected with either typical (KN99α), latent (UgCl223), or hypervirulent (UgCl422) *C. neoformans* infections had their lung proteomes compared to each other or uninfected mice. Tables were generated from the identified significant proteins for each Student's t-test comparison.

**Murine Proteins: Typical versus Uninfected**				
**Enriched in:**	**Fold Change**	**p-value**	**Protein Name**	**Gene**
Uninfected	-3.8903464	0.0302026	Tubulin alpha-1C chain;Detyrosinated tubulin alpha-1C chain;Tubulin alpha chain	Tuba1c;Gm49450
Uninfected	-3.261758804	0.001810232	Myosin light chain 4	Myl4
Uninfected	-2.725898192	0.000503939	Platelet glycoprotein Ib beta chain	Gp1bb
Uninfected	-2.616289478	1.28839E-05	Cytochrome P450 2F2	Cyp2f2
Uninfected	-2.439020538	3.89787E-05	Apolipoprotein A-II;Proapolipoprotein A-II	Apoa2
Uninfected	-2.398702749	0.003200194	Tetraspanin-9	Tspan9
Uninfected	-2.390859138	2.47026E-06	Serum paraoxonase/arylesterase 1;Paraoxonase	Pon1
Uninfected	-2.274617301	6.31927E-05	Aquaporin-5	Aqp5
Uninfected	-2.211455705	0.000130266	Decorin	Dcn
Uninfected	-2.181781642	2.32553E-07	Flavin-containing monooxygenase 3	Fmo3
Uninfected	-2.177976269	0.00035626	Lysine-specific demethylase 3A;Lysine-specific demethylase	Kdm3a
Uninfected	-2.13677881	1.22177E-05	Protein eva-1 homolog B	Eva1b
Uninfected	-2.117671373	0.00171425	Microsomal glutathione S-transferase 1	Mgst1
Uninfected	-2.11708285	4.33357E-06	Cytochrome P450 4B1	Cyp4b1
Uninfected	-2.106597646	0.001711931	Apolipoprotein A-I;Proapolipoprotein A-I;Truncated apolipoprotein A-I	Apoa1
Uninfected	-2.090507211	0.003406427	Myosin-6	Myh6
Uninfected	-2.051835717	0.001728336	Vigilin	Gm382
Uninfected	-2.038808738	0.005391227	Metabotropic glutamate receptor 5	Grm5
Uninfected	-2.00221405	0.000706641	Aldehyde dehydrogenase 1A1	Aldh1a1
Uninfected	-1.97316068	0.003376108	Troponin I, cardiac muscle	Tnni3
Uninfected	-1.942933951	0.00380708	Diacylglycerol O-acyltransferase 1	Dgat1
Uninfected	-1.934219699	0.001971894	Rho GTPase-activating protein 15	Arhgap15
Uninfected	-1.930862681	0.000816819	Ammonium transporter Rh type A	Rhag
Uninfected	-1.920129352	1.56582E-05	Periaxin	Prx
Uninfected	-1.915220642	0.004316665	Fibronectin type III domain containing protein 3C1	Fndc3c1
Uninfected	-1.892539893	0.000117671	Apolipoprotein C-I;Truncated apolipoprotein C-I	Apoc1
Uninfected	-1.880610275	0.002358122	EH domain-containing protein 3	Ehd3
Uninfected	-1.872104687	0.003737795	Apolipoprotein C-IV	Apoc4
Uninfected	-1.861481476	0.000230997	CD99 antigen	Cd99
Uninfected	-1.858058972	0.000176918	Methanethiol oxidase;Selenium-binding protein 2	Selenbp1;Selenbp2
Uninfected	-1.807055537	9.875E-05	Indolethylamine N-methyltransferase	Inmt
Uninfected	-1.783700858	6.7488E-06	Angiotensin-converting enzyme;Angiotensin-converting enzyme, soluble form;Angiotensin-converting enzyme	Ace
Uninfected	-1.769128376	5.51761E-05	Atrial natriuretic peptide receptor 3	Npr3
Uninfected	-1.766741943	8.60474E-05	Thiol methyltransferase 1A1	Tmt1a
Uninfected	-1.759405518	0.001766887	Protein FAM83H	Fam83h
Uninfected	-1.748348067	8.60209E-05	Equilibrative nucleobase transporter 1	Slc43a3
Uninfected	-1.745904329	0.00284355	Ral guanine nucleotide dissociation stimulator-like 3	Rgl3
Uninfected	-1.693463813	0.000251313	Mimecan	Ogn
Uninfected	-1.668864187	0.001159125	DENN domain-containing protein 4B	Dennd4b
Uninfected	-1.663719474	0.000565393	Apolipoprotein A-IV	Apoa4
Uninfected	-1.65761367	1.09395E-05	Tropomodulin-1	Tmod1
Uninfected	-1.628869587	0.001628256	Coagulation factor X;Factor X light chain;Factor X heavy chain;Activated factor Xa heavy chain;Coagulation factor X	F10
Uninfected	-1.617914539	2.49764E-06	Dimethylaniline monooxygenase [N-oxide-forming] 2	Fmo2
Uninfected	-1.614220005	0.001807156	Transmembrane protein 14C	Tmem14c
Uninfected	-1.608295822	4.03448E-05	EH domain-containing protein 4	Ehd4
Uninfected	-1.596683205	0.000147908	Retinoic acid-induced protein 3	Gprc5a
Uninfected	-1.59401936	4.20036E-05	Alpha-2-antiplasmin	Serpinf2
Uninfected	-1.590427314	0.000476699	Caveolae-associated protein 1	Cavin1
Uninfected	-1.56840299	0.000722192	Glutamate-rich protein 5	Erich5
Uninfected	-1.558240043	3.13921E-05	AHNAK nucleoprotein	Ahnak

Uninfected	-1.544942729	0.000500393	UDP-glucuronosyltransferase 2A1;UDP-glucuronosyltransferase 2A2	Gm43638;Ugt2a1;Ugt2a2
Uninfected	-1.520250278	0.000113202	Cytochrome P450;Cytochrome P450 2B10	Cyp2b10
Uninfected	-1.517183982	0.000980441	Chloride intracellular channel protein 5;Chloride intracellular channel protein	Clic5
Uninfected	-1.507090293	0.000651802	Pirin	Pir
Uninfected	-1.489726808	0.00077795	Dipeptidase 1;Dipeptidase	Dpep1
Uninfected	-1.476502736	0.000312913	Aldehyde dehydrogenase, mitochondrial	Aldh2
Uninfected	-1.469898478	0.00056639	DENN domain-containing protein 1A	Dennd1a
Uninfected	-1.468234762	5.38004E-05	Tubulin polymerization-promoting protein family member 3	Tppp3
Uninfected	-1.467767292	3.51762E-05	Tensin-1	Tns1
Uninfected	-1.462750329	1.9218E-05	Alcohol dehydrogenase 1	Adh1
Uninfected	-1.447773404	0.000324932	Calcitonin gene-related peptide type 1 receptor	Calcrl
Uninfected	-1.432194689	0.000237845	Vinexin	Sorbs3
Uninfected	-1.425589244	9.49852E-05	Heat shock 70 kDa protein 12B	Hspa12b
Uninfected	-1.401445474	9.03693E-05	EH domain-containing protein 2	Ehd2
Uninfected	-1.36042205	0.00012538	Cadherin-13	Cdh13
Uninfected	-1.31705886	2.13029E-05	Guanine nucleotide-binding protein G(o) subunit alpha	Gnao1
Uninfected	-1.305910873	8.52764E-07	Sodium channel protein type 7 subunit alpha	Scn7a
Uninfected	-1.303697247	4.30937E-06	Platelet factor 4	Pf4
Uninfected	-1.283807161	1.36941E-06	Epoxide hydrolase 1;Epoxide hydrolase	Ephx1
Uninfected	-1.271624968	5.96609E-07	Myosin-10	Myh10
Uninfected	-1.259520213	7.85384E-07	Plasmalemma vesicle-associated protein	Plvap
Uninfected	-1.233108605	6.6061E-06	Partitioning defective 6 homolog beta	Pard6b
Uninfected	-1.22792511	1.41821E-05	Junction plakoglobin	Jup
Uninfected	-1.195153088	4.10403E-08	Tensin-2	Tns2
Typical	1.142888429	3.99729E-07	Hexokinase-2	Hk2
Typical	1.237340461	3.66369E-06	Leucine-rich repeat-containing protein 59;Leucine-rich repeat-containing protein 59, N-terminally processed	Lrrc59
Typical	1.24892985	3.37362E-05	6-phosphofructo-2-kinase/fructose-2,6-bisphosphatase 4;6-phosphofructo-2-kinase;Fructose-2,6-bisphosphatase	Pfkfb4
Typical	1.306776301	4.13033E-07	Alpha-enolase;phosphopyruvate hydratase	Eno1
Typical	1.307193968	8.8576E-07	1-phosphatidylinositol 4,5-bisphosphate phosphodiesterase gamma-2	Plcg2
Typical	1.335494317	7.54548E-07	6-phosphofructo-2-kinase/fructose-2,6-bisphosphatase 2;6-phosphofructo-2-kinase;Fructose-2,6-bisphosphatase;6-phosphofructo-2-kinase/fructose-2,6-bisphosphatase 2	Pfkfb2
Typical	1.350408427	0.000169817	Bridging integrator 2	Bin2
Typical	1.356679874	8.59057E-07	Transferrin receptor protein 1	Tfrc
Typical	1.366233868	4.17806E-06	Heme oxygenase 1;Heme oxygenase 1 soluble form	Hmox1
Typical	1.379939821	2.86322E-05	Scinderin	Scin
Typical	1.414787843	6.29277E-05	Peroxiredoxin-4	Prdx4
Typical	1.414878803	1.84754E-06	Receptor-type tyrosine-protein phosphatase C;protein-tyrosine-phosphatase	Ptprc
Typical	1.422268592	0.000200861	Coatomer subunit zeta;Coatomer subunit zeta-1	Copz1
Typical	1.429038366	2.45436E-09	Adenylate kinase 2, mitochondrial	Ak2
Typical	1.433562681	0.000155119	Platelet-activating factor acetylhydrolase	Pla2g7
Typical	1.435401493	8.35346E-05	CMP-N-acetylneuraminate-beta-galactosamide-alpha-2,3-sialyltransferase 4	St3gal4
Typical	1.435675006	0.000155888	Carboxypeptidase;Lysosomal protective protein;Lysosomal protective protein 32 kDa chain;Lysosomal protective protein 20 kDa chain	Ctsa
Typical	1.442544132	1.12623E-06	Tyrosine-protein kinase SYK;Tyrosine-protein kinase	Syk
Typical	1.464894019	4.7127E-05	Z-DNA-binding protein 1	Zbp1
Typical	1.470771366	0.000106218	Voltage-gated potassium channel subunit beta-1;Voltage-gated potassium channel subunit beta-2	Kcnab2
Typical	1.485854721	6.52872E-05	Docking protein 2	Dok2
Typical	1.500350613	0.000708435	H-2 class II histocompatibility antigen, E-K alpha chain;H-2 class II histocompatibility antigen, E-D alpha chain	H2-Ea
Typical	1.511413023	0.000280739	Collagen alpha-5(VI) chain	Col6a5
Typical	1.519877921	1.49488E-05	Retinoid-inducible serine carboxypeptidase	Scpep1
Typical	1.533645672	0.001030558	Neutrophil cytosol factor 1	Ncf1
Typical	1.540749529	0.001219689	CD177 antigen	Cd177
Typical	1.551034207	3.12351E-06	Protein disulfide isomerase Creld2	Creld2
Typical	1.572620265	0.000578757	TATA-box binding protein associated factor 7 like 2	Taf7l2
Typical	1.580805884	0.000954246	Interferon-induced protein 44	Ifi44

Typical	1.587898212	1.32673E-06	Pro-cathepsin H;Cathepsin H mini chain;Cathepsin H;Cathepsin H heavy chain;Cathepsin H light chain;Pro-cathepsin H	Ctsh
Typical	1.602117878	0.000353196	Prosaposin;Prosaposin;Saposin-A;Saposin-B-Val;Saposin-B;Saposin-C;Saposin-D	Psap
Typical	1.606269752	3.94562E-05	Cytokine receptor-like factor 3	Crlf3
Typical	1.608284929	1.91797E-06	Cytosolic phospholipase A2 gamma	Pla2g4c
Typical	1.608607864	2.17711E-07	Macrophage mannose receptor 1	Mrc1
Typical	1.610011376	0.000279279	Stromal cell-derived factor 2-like protein 1	Sdf2l1
Typical	1.615792762	0.001240051	Retinol-binding protein 4	Rbp4
Typical	1.626465098	4.58215E-06	Hematopoietic lineage cell-specific protein	Hcls1
Typical	1.637871679	0.001384479	Tenascin	Tnc
Typical	1.660057407	1.28401E-05	Beta-1,4-galactosyltransferase 1;Processed beta-1,4-galactosyltransferase 1;Beta-1,4-galactosyltransferase	B4galt1
Typical	1.68626976	4.21801E-05	Glycine amidinotransferase, mitochondrial	Gatm
Typical	1.69045762	0.002643681	ATP-binding cassette subfamily G member 4	Abcg4
Typical	1.692434523	0.001961811	Cathepsin K	Ctsk
Typical	1.692882411	0.000489749	7-dehydrocholesterol reductase	Dhcr7
Typical	1.6946757	0.001793837	tRNA (32-2-O)-methyltransferase regulator THADA	Thada
Typical	1.700190735	0.002431607	Mediator of RNA polymerase II transcription subunit 4	Med4
Typical	1.706982888	1.67562E-09	Dedicator of cytokinesis protein 2	Dock2
Typical	1.713223606	0.000623553	Solute carrier family 35 member F6	Slc35f6
Typical	1.717988926	0.000112264	H-2 class II histocompatibility antigen, A-K beta chain;H-2 class II histocompatibility antigen, A-F beta chain;H-2 class II histocompatibility antigen, A-U beta chain;H-2 class II histocompatibility antigen, A beta chain	P06343;P06346;P06344;P14483
Typical	1.74029274	0.0028931	Neutrophil cytosol factor 4	Ncf4
Typical	1.789911313	0.002876529	MAP kinase-activated protein kinase 3	Mapkapk3
Typical	1.800175561	0.00024852	Caspase-3;Caspase-3 subunit p17;Caspase-3 subunit p12	Casp3
Typical	1.800235706	0.002742745	SH3 domain-binding glutamic acid-rich-like protein 3	Sh3bgrl3
Typical	1.810012775	0.003792897	SH3 domain-containing protein 21	Sh3d21
Typical	1.81047643	3.29074E-06	Integrin alpha-X	Itgax
Typical	1.81118571	0.000229807	Neutrophil gelatinase-associated lipocalin	Lcn2
Typical	1.82603781	0.00116906	DNA (cytosine-5)-methyltransferase 3B;DNA (cytosine-5-)-methyltransferase	Dnmt3b
Typical	1.826227273	0.003390103	Myeloblastin	Prtn3
Typical	1.826531474	0.003584237	Peptidoglycan recognition protein 1	Pglyrp1
Typical	1.834822803	9.24902E-08	Protein disulfide-isomerase A4	Pdia4
Typical	1.838756561	0.00326792	Interleukin-1 receptor antagonist protein;Interleukin-1	Il1rn
Typical	1.850600137	4.71127E-05	Unconventional myosin-If	Myo1f
Typical	1.851141824	7.13828E-07	Tyrosine-protein phosphatase non-receptor type 6	Ptpn6
Typical	1.865217039	2.08983E-05	Fc fragment of IgG binding protein	Fcgbp
Typical	1.889297782	0.000939126	Plasminogen activator inhibitor 2, macrophage	Serpinb2
Typical	1.898328739	5.18583E-06	Mucin 5, subtype B, tracheobronchial	Muc5b
Typical	1.922814178	2.30492E-08	Creatine kinase U-type, mitochondrial	Ckmt1
Typical	1.931701194	0.001326938	Histone H1.3	H1-3
Typical	1.931843228	0.000192026	Lysosomal alpha-mannosidase	Man2b1
Typical	1.942413288	0.006595199	PWWP domain-containing protein 2B	Pwwp2b
Typical	1.943157789	0.003825181	Cathepsin S	Ctss
Typical	1.945556386	0.000289379	Oxidized low-density lipoprotein receptor 1;Oxidized low-density lipoprotein receptor 1, soluble form	Olr1
Typical	1.969020504	0.001971078	Immunoglobulin kappa variable 14-126	Igkv14-126
Typical	1.977684614	0.006062603	Dynein regulatory complex protein 11	Iqca1
Typical	1.979872386	8.92164E-08	Beta-glucuronidase	Gusb
Typical	1.993260532	2.79552E-05	Transmembrane emp24 domain-containing protein 3	Tmed3
Typical	1.995051448	8.83251E-05	Myeloperoxidase;Myeloperoxidase light chain;Myeloperoxidase heavy chain	Mpo
Typical	2.000655789	0.000200246	M-phase phosphoprotein 6	Mphosph6
Typical	2.078723526	0.000372833	Immunoglobulin J chain	Jchain
Typical	2.083185365	2.93289E-06	Arylsulfatase B	Arsb
Typical	2.160101488	0.000518525	Immunoglobulin heavy constant alpha	Igha
Typical	2.165073268	1.40034E-06	Neutrophil cytosol factor 2	Ncf2
Typical	2.181040149	1.68303E-06	Ras-related C3 botulinum toxin substrate 2	Rac2
Typical	2.187361399	1.01814E-05	Macrophage-capping protein	Capg

Typical	2.191780175	4.51016E-06	Cathepsin B;Cathepsin B light chain;Cathepsin B heavy chain	Ctsb
Typical	2.200200738	0.010407012	Pseudouridylate synthase PUS7L	Pus7l
Typical	2.20413759	0.000189292	Maestro heat-like repeat-containing protein family member 2B	Mroh2b
Typical	2.222065269	2.03982E-05	5-3 exonuclease PLD3	Pld3
Typical	2.223563894	6.17496E-06	BPI fold-containing family B member 1	Bpifb1
Typical	2.240097385	0.000280475	Lysophosphatidylcholine acyltransferase 2	Lpcat2
Typical	2.305179554	4.93444E-05	Coactosin-like protein	Cotl1
Typical	2.322163052	0.000469263	Fructose-1,6-bisphosphatase 1	Fbp1
Typical	2.345358319	3.27425E-05	Reticulocalbin-3	Rcn3
Typical	2.372813161	1.48824E-06	Cathepsin D	Ctsd;Gm49369
Typical	2.375229136	0.000126794	Rho GDP-dissociation inhibitor 2	Arhgdib
Typical	2.384254837	3.15522E-08	Beta-hexosaminidase subunit beta	Hexb
Typical	2.408304765	3.32633E-08	Coronin-1A;Coronin	Coro1a
Typical	2.419163556	0.000213658	Neutrophilic granule protein	Ngp
Typical	2.458104875	0.001667034	Macrosialin	Cd68
Typical	2.475529226	7.91509E-06	Macrophage galactose N-acetyl-galactosamine specific lectin 2	Mgl2
Typical	2.490200043	4.63625E-05	Cytochrome b-245 heavy chain	Cybb
Typical	2.502862464	8.64461E-05	Ig alpha chain C region	P01878
Typical	2.527554279	1.99046E-06	Lactotransferrin	Ltf
Typical	2.549826516	0.001458377	Host cell factor 2	Hcfc2
Typical	2.566139476	2.66678E-05	Polyunsaturated fatty acid lipoxygenase ALOX15	Alox15
Typical	2.578759045	1.52013E-05	Macrophage metalloelastase	Mmp12
Typical	2.592364799	0.014515349	Ribonuclease, RNase A family, 2A	Rnase2a
Typical	2.594527774	1.98569E-06	Chitinase-3-like protein 1	Chi3l1
Typical	2.629729038	3.96879E-05	Brain acid soluble protein 1	Basp1
Typical	2.630251778	2.03019E-06	Galectin-3;Galectin	Lgals3
Typical	2.658108605	0.021229463	Uncharacterized protein CXorf38 homolog	Q8C5K5;Q8C5K5-2
Typical	2.680900828	6.88184E-07	Polymeric immunoglobulin receptor;Secretory component	Pigr
Typical	2.692890761	9.12906E-09	Integrin beta-2;Integrin beta	Itgb2
Typical	2.755876075	3.6301E-06	Low affinity immunoglobulin gamma Fc region receptor II	Fcgr2;Fcgr2b
Typical	2.756715224	1.80837E-07	Integrin alpha-M	Gm49368;Itgam
Typical	2.864708752	3.69763E-05	Legumain	Lgmn
Typical	2.930420007	0.000426839	Anterior gradient protein 2 homolog	Agr2
Typical	3.057472822	3.35775E-06	Cathepsin Z	Ctsz
Typical	3.110195118	1.41565E-05	Acidic mammalian chitinase	Chia;Chia1
Typical	3.391048559	3.29426E-06	Procathepsin L;Cathepsin L;Cathepsin L heavy chain;Cathepsin L light chain	Ctsl
Typical	3.439463594	0.03592232	Predicted gene	Gm17349
Typical	3.887849045	8.01952E-08	Synaptotagmin-16	Syt16
Typical	3.965682644	1.29325E-05	Intelectin-1a	Itln1
Typical	4.168359884	6.2417E-10	Pulmonary surfactant-associated protein D	Sftpd
Typical	4.328240797	4.81288E-05	Eosinophil cationic protein 2	Ear2
Typical	4.850910992	9.05843E-06	Eosinophil-associated, ribonuclease A family, member 10	Ear10
Typical	4.867788484	0.000133009	Eosinophil-associated, ribonuclease A family, member 6	Ear6
Typical	4.887753338	6.84259E-07	Bone marrow proteoglycan;Eosinophil granule major basic protein	Prg2
Typical	5.080402035	2.73701E-08	Arginase-1	Arg1
Typical	5.595628272	2.37979E-10	Chitinase-like protein 3	Chil3
Typical	5.718445926	1.63228E-07	Calcium-activated chloride channel regulator 1	Clca1
Typical	5.822447586	1.08544E-08	Eosinophil peroxidase;Eosinophil peroxidase light chain;Eosinophil peroxidase heavy chain	Epx
Typical	6.195193948	2.92658E-07	Chitinase-like protein 4	Chil4
Typical	7.688739649	1.11892E-09	Resistin-like alpha	Retnla

Murine Proteins: Latent versus Uninfected				
Enriched in:	Fold Change	p-value	Protein Name	Gene
Uninfected	-4.88478642	5.2955E-08	Myoglobin	Mb
Uninfected	-4.49291229	4.445E-11	Indolethylamine N-methyltransferase	Inmt
Uninfected	-4.46252518	0.01356655	Tubulin alpha-1C chain;Detyrosinated tubulin alpha-1C chain;Tubulin alpha chain	Tuba1c;Gm49450
Uninfected	-4.44697266	0.00139093	Tubulin alpha-1B chain;Detyrosinated tubulin alpha-1B chain	Tuba1b
Uninfected	-4.43080215	9.6222E-09	Carboxylic ester hydrolase	Ces1b
Uninfected	-4.27430363	1.1521E-09	Putative protein-lysine deacylase ABHD14B	Abhd14b
Uninfected	-4.23877468	6.845E-12	SEC14-like lipid binding 3	Sec14l3
Uninfected	-4.21714535	6.2076E-10	L-lactate dehydrogenase B chain;L-lactate dehydrogenase	Ldhb
Uninfected	-4.03054657	1.1494E-08	Serotransferrin	Tf
Uninfected	-4.02661438	3.9416E-10	Glutathione S-transferase A3	Gsta3
Uninfected	-3.99159985	5.4101E-09	Glutathione S-transferase Mu 1;Glutathione S-transferase;glutathione transferase	Gstm1
Uninfected	-3.97134151	9.1443E-06	Protein S100-G	S100g
Uninfected	-3.94748821	3.8857E-09	Hemopexin	Hpx
Uninfected	-3.904319	8.3756E-07	Alpha-2-HS-glycoprotein	Ahsg
Uninfected	-3.86856537	7.5332E-07	Carboxylesterase 1C	Ces1c
Uninfected	-3.85078278	9.0752E-10	Pregnancy zone protein;Alpha-2-macroglobulin 165 kDa subunit;Alpha-2-macroglobulin 35 kDa subunit	Pzp
Uninfected	-3.84908733	7.9498E-08	Transthyretin	Ttr
Uninfected	-3.81022987	7.7969E-06	Ankyrin repeat domain-containing protein 49	Ankrd49
Uninfected	-3.8085247	4.9082E-06	Alpha-1-antitrypsin 1-2	Serpina1b
Uninfected	-3.76730595	1.0697E-06	Apolipoprotein A-II;Proapolipoprotein A-II	Apoa2
Uninfected	-3.71630707	2.0013E-08	Alcohol dehydrogenase 1	Adh1
Uninfected	-3.67234077	1.4656E-08	Albumin	Alb
Uninfected	-3.66793003	2.204E-11	Glutathione S-transferase Mu 2;Glutathione S-transferase	Gstm2
Uninfected	-3.58584061	3.374E-08	Methanethiol oxidase;Selenium-binding protein 2	Selenbp1;Selenbp2
Uninfected	-3.5134964	3.136E-09	Peroxiredoxin-6	Prdx6
Uninfected	-3.51120625	3.9856E-09	Alpha-1-antitrypsin 1-4	Serpina1d
Uninfected	-3.48974609	2.8085E-10	Vitamin D-binding protein	Gc
Uninfected	-3.48681717	3.4362E-06	Afamin	Afm
Uninfected	-3.48416309	5.5704E-06	Thymosin beta-10;Thymosin beta-4;Hemoregulatory peptide AcSDKP	Tmsb10;Tmsb4x
Uninfected	-3.48385963	5.8383E-08	Serine (or cysteine) peptidase inhibitor, clade A, member 3K	Serpina3k
Uninfected	-3.48166771	1.8621E-07	Leucine-rich alpha-2-glycoprotein 1	Lrg1
Uninfected	-3.45338955	3.9417E-05	Aldehyde dehydrogenase 1A1	Aldh1a1
Uninfected	-3.35682735	1.1973E-09	Homeodomain-only protein	Hopx
Uninfected	-3.34549809	3.7663E-08	Fumarylacetoacetase	Fah
Uninfected	-3.26344643	4.9184E-10	m7GpppX diphosphatase	Dcps
Uninfected	-3.23915558	0.00126667	Fetuin-B	Fetub
Uninfected	-3.22847366	1.8315E-07	Astrocytic phosphoprotein PEA-15	Pea15;Pea15a
Uninfected	-3.21400566	1.1226E-07	Dihydropyrimidinase-related protein 2	Dpysl2
Uninfected	-3.20689583	3.9783E-07	Tubulin polymerization-promoting protein family member 3	Tppp3
Uninfected	-3.19387665	1.8014E-06	Kininogen-1;Kininogen-1 heavy chain;Bradykinin;Kininogen-1 light chain	Kng1
Uninfected	-3.17917347	6.6865E-06	Glutathione S-transferase theta-1	Gstt1
Uninfected	-3.12022343	8.9131E-10	Carbonic anhydrase 3	Ca3
Uninfected	-3.10693874	2.1063E-07	Ferritin heavy chain;Ferritin heavy chain, N-terminally processed;Ferritin	Fth1
Uninfected	-3.07268639	7.2842E-06	Fatty acid-binding protein, heart	Fabp3
Uninfected	-3.06840286	1.2773E-07	L-xylulose reductase	Dcxr
Uninfected	-3.06530342	3.8129E-09	Xanthine dehydrogenase/oxidase;Xanthine dehydrogenase;Xanthine oxidase	Xdh
Uninfected	-3.04229965	9.1138E-08	Apolipoprotein A-IV	Apoa4
Uninfected	-3.04116268	0.00163933	D-dopachrome decarboxylase	Ddt
Uninfected	-3.0397892	0.00021374	Corticosteroid-binding globulin	Serpina6
Uninfected	-3.03853798	1.4118E-09	Aldo-keto reductase family 1 member B1	Akr1b1
Uninfected	-3.03756256	6.881E-10	Aldehyde dehydrogenase, cytosolic 1	Aldh1a7
Uninfected	-3.02055073	9.6828E-07	Four and a half LIM domains protein 1	Fhl1
Uninfected	-3.01688156	2.6126E-08	glutathione transferase	Gm20441
Uninfected	-2.98972645	0.00679353	Aldo-keto reductase family 1, member C14	Akr1c14
Uninfected	-2.98802147	2.7418E-13	Fatty acid synthase;[Acyl-carrier-protein] S-acetyltransferase;[Acyl-carrier-protein] S-malonyltransferase;3-oxoacyl-[acyl-carrier-protein] synthase;3-oxoacyl-[acyl-carrier-protein] reductase;3-hydroxyacyl-[acyl-carrier-protein] dehydratase;Enoyl-[acyl-carrier-protein] reductase;Acyl-[acyl-carrier-protein] hydrolase;Fatty acid synthase	Fasn
Uninfected	-2.98347816	1.679E-08	Glutathione S-transferase A4	Gsta4
Uninfected	-2.98321476	6.7438E-11	Serine (or cysteine) peptidase inhibitor, clade B, member 9	Serpinb9
Uninfected	-2.98301983	2.6636E-09	Glutathione S-transferase omega-1;Glutathione S-transferase omega	Gsto1
Uninfected	-2.92763462	6.0764E-10	Serpin B6	Serpinb6a;Serpinb6
Uninfected	-2.92541237	1.6815E-06	Alpha-1B-glycoprotein	A1bg

Uninfected	-2.91967583	0.00010017	Pulmonary surfactant-associated protein B	Sftpb	
Uninfected	-2.9119421	4.0566E-08	Peptidyl-prolyl cis-trans isomerase FKBP1A	Fkbp1a	
Uninfected	-2.87834129	1.1553E-09	Phosphoglycerate mutase 1	Pgam1	
Uninfected	-2.8654623	1.6419E-11	Dual specificity protein phosphatase 3;Dual specificity protein phosphatase;protein-serine/threonine phosphatase	Dusp3	
Uninfected	-2.86466274	0.00030989	Serum amyloid P-component	Apcs	
Uninfected	-2.84625797	4.1006E-08	Annexin A3	Anxa3	
Uninfected	-2.84395046	4.578E-06	Creatine kinase B-type	Ckb	
Uninfected	-2.84067631	0.01220232	Glutathione S-transferase theta-3;glutathione transferase	Gstt3	
Uninfected	-2.82008705	0.00010074	Adenylate kinase isoenzyme 1;nucleoside-triphosphate--adenylate kinase	Ak1	
Uninfected	-2.81227875	1.3658E-08	Transgelin-2	Tagln2	
Uninfected	-2.81021423	0.00205001	Ig gamma-1 chain C region secreted form;Ig gamma-1 chain C region, membrane-bound form	Ighg1	
Uninfected	-2.80493011	9.7573E-06	Ester hydrolase C11orf54 homolog	4931406C07Rik	
Uninfected	-2.7755621	1.6064E-07	Apolipoprotein A-I;Proapolipoprotein A-I;Truncated apolipoprotein A-I	Apoa1	
Uninfected	-2.77138481	2.1017E-06	Ig gamma-2A chain C region, membrane-bound form;Ig gamma-2A chain C region, A allele	Igh-1a;Ighg	1a
Uninfected	-2.76331482	4.1885E-07	Cytochrome P450 2F2	Cyp2f2	
Uninfected	-2.76147366	1.9054E-09	Murinoglobulin-1	Mug1	
Uninfected	-2.75701962	0.01751047	Receptor expression-enhancing protein 6	Reep6	
Uninfected	-2.74314651	5.949E-06	Serpin A1	Serpina1a;Serpina1c	
Uninfected	-2.73891315	2.7315E-06	Acidic leucine-rich nuclear phosphoprotein 32 family member B	Anp32b	
Uninfected	-2.73697166	4.7166E-08	Chloride intracellular channel protein 3	Clic3	
Uninfected	-2.73517628	1.2069E-09	ATP-citrate synthase	Acly	
Uninfected	-2.72306442	1.9712E-07	Fatty acid-binding protein, adipocyte	Fabp4	
Uninfected	-2.70148029	1.2732E-08	Calpain-2 catalytic subunit	Capn2	
Uninfected	-2.69183979	4.0981E-05	glycogenin glucosyltransferase;Glycogenin-1	Gyg1	
Uninfected	-2.68659267	6.4951E-08	Acidic leucine-rich nuclear phosphoprotein 32 family member A;Acidic leucine-rich nuclear phosphoprotein 32 family member	Anp32a	
Uninfected	-2.68361206	0.00027907	Glutathione S-transferase P 1;Glutathione S-transferase;glutathione transferase;Glutathione S-transferase P 2	Gstp1;Gstp2	
Uninfected	-2.67798004	4.7762E-07	Calponin-3;Calponin	Cnn3	
Uninfected	-2.67779274	5.8179E-06	Prothymosin alpha;Prothymosin alpha, N-terminally processed;Thymosin alpha	Ptma	
Uninfected	-2.67116375	3.2544E-08	Serum paraoxonase/arylesterase 1;Paraoxonase	Pon1	
Uninfected	-2.6477541	1.5741E-05	Tubulin alpha-1A chain;Detyrosinated tubulin alpha-1A chain	Tuba1a	
Uninfected	-2.62635384	0.0002494	Inositol polyphosphate 1-phosphatase	Inpp1	
Uninfected	-2.62272129	7.5871E-07	Bromodomain testis-specific protein	Brdt	
Uninfected	-2.6045145	5.9521E-08	Tryptophan--tRNA ligase, cytoplasmic;T1-TrpRS;T2-TrpRS	Wars1	
Uninfected	-2.60263615	0.00444865	Protein S100;Protein S100-A1	S100a1	
Uninfected	-2.59685669	2.1671E-07	GTPase IMAP family member 4	Gimap4	
Uninfected	-2.58128262	4.8553E-07	Complement C2;Complement factor B;Complement factor B Ba fragment;Complement factor B Bb fragment;Complement factor B	Gm20547;Cfb	
Uninfected	-2.5780386	9.2511E-06	Tubulin beta-2A chain;Tubulin beta-2B chain	Tubb2a;Tubb2b	
Uninfected	-2.56695213	2.9315E-07	Hepatoma-derived growth factor	Hdgf	
Uninfected	-2.54650917	1.3026E-08	Pyruvate kinase PKM	Pkm	
Uninfected	-2.5417078	0.00373268	Uteroglobin	Scgb1a1	
Uninfected	-2.5404808	4.9051E-05	Stromal membrane-associated protein 1	Smap1	
Uninfected	-2.53454151	4.6093E-06	Parathymosin	Ptms	
Uninfected	-2.53082218	0.01244797	COMM domain-containing protein 8	Commd8	
Uninfected	-2.49929085	3.8781E-07	Isocitrate dehydrogenase [NADP] cytoplasmic	Idh1	
Uninfected	-2.48371258	1.0515E-05	Tubulin beta-5 chain	Tubb5	
Uninfected	-2.48280773	8.1751E-06	Glucose-6-phosphate isomerase	Gpi	
Uninfected	-2.46815987	1.304E-08	Chloride intracellular channel protein 1	Clic1	
Uninfected	-2.45665913	1.0548E-05	Ig gamma-3 chain C region	Ighg3	
Uninfected	-2.44336758	0.00095132	Costars family protein ABRACL	Abracl	
Uninfected	-2.43876591	9.6118E-05	Pyridoxal kinase	Pdxk	
Uninfected	-2.43824978	0.00539538	Tubulin beta-3 chain	Tubb3	
Uninfected	-2.43245087	0.00021014	Transgelin	Tagln	
Uninfected	-2.43202839	1.3955E-07	Malate dehydrogenase, cytoplasmic;Malate dehydrogenase	Mdh1	
Uninfected	-2.43045826	1.0465E-06	Rab GDP dissociation inhibitor alpha	Gdi1	
Uninfected	-2.4257019	1.1234E-05	Sepiapterin reductase	Spr	
Uninfected	-2.4198988	7.4973E-06	MYG1 exonuclease	Myg1	
Uninfected	-2.41589451	5.3372E-05	Acyl-CoA-binding protein	Dbi	
Uninfected	-2.40515575	2.3017E-06	Dihydropyrimidinase-related protein 3	Dpysl3	
Uninfected	-2.39967175	0.00375379	cAMP-dependent protein kinase catalytic subunit alpha	Prkaca	
Uninfected	-2.39649925	0.0002662	Dynein cytoplasmic 1 heavy chain 1	Dync1h1	
Uninfected	-2.38539009	0.00024293	Serpin B6	Serpinb6b	
Uninfected	-2.38131027	0.00010844	Cysteine and glycine-rich protein 1	Csrp1	

Uninfected	-2.38025322	0.00470818	glutamate rich 6B	Erich6b
Uninfected	-2.37678413	0.01197536	Sterile alpha motif domain-containing protein 15	Samd15
Uninfected	-2.36929436	6.7508E-09	Deoxynucleoside triphosphate triphosphohydrolase SAMHD1	Samhd1
Uninfected	-2.36800461	7.2966E-07	Cysteine-rich protein 1	Crip1
Uninfected	-2.36541023	2.3353E-06	Serine protease inhibitor A3M	Serpina3m
Uninfected	-2.36323528	0.02685513	P2R1A-PPP2R2A-interacting phosphatase regulator 1	Pabir1
Uninfected	-2.36186752	1.521E-06	NADP-dependent malic enzyme;Malic enzyme	Me1
Uninfected	-2.35852509	1.0132E-06	Myotrophin	Mtpn
Uninfected	-2.33760147	7.1371E-07	Aldo-keto reductase family 1 member A1	Akr1a1
Uninfected	-2.33285561	0.00092142	Dynamin-2;dynamin GTPase	Dnm2
Uninfected	-2.32479572	0.0105568	ADP-ribosylation factor-like protein 1	Arl1
Uninfected	-2.32390823	2.373E-08	Prothrombin;Activation peptide fragment 1;Activation peptide fragment 2;Thrombin light chain;Thrombin heavy chain;Prothrombin	F2
Uninfected	-2.32314968	2.8508E-06	Heat shock protein HSP 90-alpha	Hsp90aa1
Uninfected	-2.32152958	1.9092E-06	SUMO-activating enzyme subunit 2	Uba2
Uninfected	-2.32013359	1.1661E-07	Ferritin light chain 1;Ferritin;Ferritin light chain 2	Ftl1;Ftl2
Uninfected	-2.31018677	1.1876E-06	Heparin cofactor 2	Serpind1
Uninfected	-2.30713768	2.356E-05	Cytosolic acyl coenzyme A thioester hydrolase	Acot7
Uninfected	-2.30673885	0.00012129	Phosphoglycerate mutase 2	Pgam2
Uninfected	-2.30258884	1.1571E-05	Selenide, water dikinase 1	Sephs1
Uninfected	-2.29849224	2.0891E-06	Hsc70-interacting protein	St13
Uninfected	-2.29052734	0.01007313	ADAMTS-like protein 2	Adamtsl2
Uninfected	-2.28741417	0.001565	Cystatin-B	Cstb
Uninfected	-2.28699551	6.9409E-07	Heat shock protein beta-1	Hspb1
Uninfected	-2.2868351	0.00162703	Acidic leucine-rich nuclear phosphoprotein 32 family member;Acidic leucine-rich nuclear phosphoprotein 32 family member E	Anp32e
Uninfected	-2.28048916	8.5941E-05	Retinol-binding protein 1	Rbp1
Uninfected	-2.27924976	9.4795E-08	Glycogen phosphorylase, brain form	Pygb
Uninfected	-2.26970997	2.9441E-07	Alpha-2-antiplasmin	Serpinf2
Uninfected	-2.26774921	7.116E-05	Adenylosuccinate synthetase isozyme 2	Adss2
Uninfected	-2.26553383	1.8858E-11	Elongation factor 2	Eef2
Uninfected	-2.26363697	5.2632E-05	Rho GDP-dissociation inhibitor 1	Arhgdia
Uninfected	-2.25966988	0.00043522	Gigaxonin	Gan
Uninfected	-2.24973526	0.00029022	Glia maturation factor beta	Gmfb
Uninfected	-2.2489933	1.4947E-06	Arfaptin-1	Arfip1;Gm37240
Uninfected	-2.24706993	0.00044135	Profilin-1;Profilin	Pfn1
Uninfected	-2.24000206	0.00211515	Protein phosphatase 1 regulatory subunit 14A;Protein phosphatase 1 regulatory subunit 14	Ppp1r14a
Uninfected	-2.23889885	1.3908E-06	Alcohol dehydrogenase class-3	Adh5
Uninfected	-2.22941132	0.02358709	Xylosyltransferase 2	Xylt2
Uninfected	-2.22420158	2.0319E-10	14-3-3 protein theta	Ywhaq
Uninfected	-2.22418251	0.00044682	Glutathione S-transferase theta-2;glutathione transferase	Gstt2
Uninfected	-2.2184721	1.0207E-07	Cytoplasmic aconitate hydratase	Aco1
Uninfected	-2.21564636	6.6827E-06	Thioredoxin domain-containing protein 17	Txndc17
Uninfected	-2.21082878	2.2313E-06	Adenosine kinase	Adk
Uninfected	-2.18278656	0.00479036	Synaptotagmin-like protein 4	Sytl4
Uninfected	-2.17506275	1.9769E-08	Zyxin	Zyx
Uninfected	-2.17487812	0.00155301	Copper transport protein ATOX1	Atox1
Uninfected	-2.17301121	2.7374E-05	Ribosome maturation protein SBDS	Sbds
Uninfected	-2.16218567	6.6411E-05	Inhibitor of carbonic anhydrase	Inhca
Uninfected	-2.15514908	0.00034703	RNA 3-terminal phosphate cyclase	RtcA;Rtca
Uninfected	-2.14964752	1.2412E-08	Echinoderm microtubule-associated protein-like 2	Eml2
Uninfected	-2.14751186	0.00011089	Voltage-gated hydrogen channel 1	Hvcn1
Uninfected	-2.14257393	1.4979E-06	Carbonyl reductase [NADPH] 1	Cbr1
Uninfected	-2.13771534	5.8224E-08	N(G),N(G)-dimethylarginine dimethylaminohydrolase 1	Ddah1
Uninfected	-2.13425655	2.2075E-07	Aspartate aminotransferase, cytoplasmic	Got1
Uninfected	-2.1290823	4.2275E-07	von Willebrand factor A domain-containing protein 5A	Vwa5a
Uninfected	-2.11052647	1.4185E-05	RNA-binding protein with multiple splicing	Rbpms
Uninfected	-2.1091711	2.122E-05	Carbonyl reductase [NADPH] 3	Cbr3
Uninfected	-2.10649548	3.0704E-08	Elongation factor 1-alpha 1	Eef1a1
Uninfected	-2.10107746	1.8561E-05	15-hydroxyprostaglandin dehydrogenase [NAD(+)]	Hpgd
Uninfected	-2.09649849	0.00024051	Protein phosphatase 1 regulatory subunit 15A	Ppp1r15a
Uninfected	-2.09569244	0.00717184	Coagulation factor X;Factor X light chain;Factor X heavy chain;Activated factor Xa heavy chain;Coagulation factor X	F10
Uninfected	-2.09435997	0.00010349	Fucose mutarotase;L-fucose mutarotase	Fuom
Uninfected	-2.09062595	4.7595E-07	Inter alpha-trypsin inhibitor, heavy chain 4	Itih4
Uninfected	-2.08969898	2.8761E-06	Plastin-2	Lcp1
Uninfected	-2.08795929	1.1917E-07	Argininosuccinate synthase	Ass1
Uninfected	-2.07979507	0.03416097	Prefoldin subunit 4	Pfdn4

Uninfected	-2.06744556	1.1937E-07	Heat shock protein HSP 90-beta	Hsp90ab1
Uninfected	-2.06543293	0.00024427	Protein S100-A6	S100a6
Uninfected	-2.06444111	0.00013132	Peptidyl-prolyl cis-trans isomerase A;Peptidyl-prolyl cis-trans isomerase A, N-terminally processed;Peptidyl-prolyl cis-trans isomerase	Ppia
Uninfected	-2.0570652	2.949E-05	Destrin	Dstn
Uninfected	-2.05489349	0.0231438	Derlin-2;Derlin;Derlin-3	Derl2;Derl3
Uninfected	-2.04413815	2.7816E-09	Transketolase;transketolase	Tkt
Uninfected	-2.04282532	1.9389E-08	3(2),5-bisphosphate nucleotidase 1	Bpnt1
Uninfected	-2.03804073	4.5346E-06	S-adenosylmethionine synthase isoform type-2;S-adenosylmethionine synthase	Mat2a
Uninfected	-2.03337231	1.8652E-05	Large ribosomal subunit protein eL38	Rpl38
Uninfected	-2.03132687	5.3417E-05	Ral guanine nucleotide dissociation stimulator-like 3	Rgl3
Uninfected	-2.02970848	0.00054052	Sorting nexin-12	Snx12
Uninfected	-2.02771969	1.0197E-06	Thioredoxin reductase 1, cytoplasmic	Txnrd1
Uninfected	-2.02596245	1.4955E-06	ADP-ribosylation factor 4	Arf4
Uninfected	-2.02435398	6.7526E-05	Ubiquitin-like modifier-activating enzyme 5	Uba5
Uninfected	-2.02030125	2.6655E-07	Rho GTPase-activating protein 1	Arhgap1
Uninfected	-2.01713047	0.00030535	Dynein light chain roadblock-type 2	Dynlrb2
Uninfected	-2.01545849	0.02660365	Microsomal glutathione S-transferase 1	Mgst1
Uninfected	-2.01024799	5.3768E-09	Glyoxalase domain-containing protein 4	Glod4
Uninfected	-2.00870476	0.00182241	Thiamin pyrophosphokinase 1	Tpk1
Uninfected	-2.00770359	2.5386E-05	Cytoplasmic dynein 1 light intermediate chain 1	Dync1li1
Uninfected	-2.00479927	0.00034946	Pirin	Pir
Uninfected	-2.0027338	2.6621E-07	Rab GDP dissociation inhibitor beta;Rab GDP dissociation inhibitor	Gdi2
Uninfected	-2.00210419	0.00518854	Putative hydroxypyruvate isomerase	Hyi
Uninfected	-1.99366379	5.0136E-07	Calcium-binding protein 39	Cab39
Uninfected	-1.98929539	2.3191E-07	Cytosolic non-specific dipeptidase	Cndp2
Uninfected	-1.9849987	0.00084736	Sorting nexin-6;Sorting nexin-6, N-terminally processed	Snx6
Uninfected	-1.98349419	1.2912E-06	Complement C3;Complement C3 beta chain;C3-beta-c;Complement C3 alpha chain;C3a anaphylatoxin;Acylation stimulating protein;Complement C3b alpha chain;Complement C3c alpha chain fragment 1;Complement C3dg fragment;Complement C3g fragment;Complement C3d fragment;Complement C3f fragment;Complement C3c alpha chain fragment 2	C3
Uninfected	-1.98225784	3.7437E-08	Plasma protease C1 inhibitor	Serping1
Uninfected	-1.97948647	0.00040872	Ankyrin-3	Ank3
Uninfected	-1.97409897	2.0728E-08	Sorting nexin-2	Snx2
Uninfected	-1.9736208	7.1895E-05	Isochorismatase domain-containing protein 1	Isoc1
Uninfected	-1.973419	1.4016E-08	EH domain-containing protein 1	Ehd1
Uninfected	-1.966786	9.5365E-08	LIM and SH3 domain protein 1	Lasp1
Uninfected	-1.9641304	0.00035326	Aldehyde oxidase 2	Aox2
Uninfected	-1.96344395	1.1404E-05	Glycerol-3-phosphate dehydrogenase [NAD(+)], cytoplasmic;Glycerol-3-phosphate dehydrogenase [NAD(+)]	Gpd1
Uninfected	-1.96044025	0.00016905	E3 ubiquitin-protein ligase RNF181	Rnf181
Uninfected	-1.95744171	0.01661918	Metabotropic glutamate receptor 5	Grm5
Uninfected	-1.95027218	3.572E-06	Histidine-rich glycoprotein	Hrg
Uninfected	-1.949226	0.01898492	Pantothenate kinase 3	Pank3
Uninfected	-1.93718624	1.5301E-05	Annexin A5	Anxa5
Uninfected	-1.9348011	0.00081924	Proteasome activator complex subunit 1	Psme1
Uninfected	-1.93093452	2.8076E-06	Bifunctional phosphoribosylaminoimidazole carboxylase/phosphoribosylaminoimidazole succinocarboxamide synthetase;Phosphoribosylaminoimidazole carboxylase;Phosphoribosylaminoimidazole succinocarboxamide synthetase	Paics
Uninfected	-1.9280695	6.1305E-06	ADP-ribosylation factor 1;ADP-ribosylation factor 3;ADP-ribosylation factor 2	Arf1;Arf3;Arf2
Uninfected	-1.92394314	2.5602E-08	Microtubule-associated protein 4	Map4
Uninfected	-1.91882839	9.2304E-07	Aldo-keto reductase family 1, member B10	Akr1b10
Uninfected	-1.91556702	7.3085E-09	Threonine--tRNA ligase 1, cytoplasmic	Tars1
Uninfected	-1.91476154	1.8476E-05	Phosphoglucomutase-1	Pgm1
Uninfected	-1.91374454	5.8157E-05	Calcyclin-binding protein	Cacybp
Uninfected	-1.90800953	2.6334E-08	Plastin-3	Pls3
Uninfected	-1.90749474	6.6835E-05	KH domain-containing RNA-binding protein QKI	Qki
Uninfected	-1.90366783	1.067E-06	Poly(rC)-binding protein 2	Pcbp2
Uninfected	-1.90303345	1.9509E-06	Tubulin beta-4B chain	Tubb4b
Uninfected	-1.90278301	0.00267116	Alpha-1-antitrypsin 1-5	Serpina1e
Uninfected	-1.9020319	1.9964E-07	Pterin-4-alpha-carbinolamine dehydratase	Pcbd1
Uninfected	-1.90064354	0.00186327	Cytochrome P450 2C44	Cyp2c23
Uninfected	-1.89942837	1.0611E-06	Glutathione S-transferase A1;Glutathione S-transferase A1, N-terminally processed;Glutathione S-transferase;Glutathione S-transferase A2	Gsta1;Gsta13;Gsta2;Gsta5
Uninfected	-1.89271164	0.00047228	Alpha-1-acid glycoprotein 1	Orm1

Uninfected	-1.88997211	0.00505241	U8 snoRNA-decapping enzyme	Nudt16
Uninfected	-1.88959751	0.00670899	Ubiquitin-like FUBI-ribosomal protein eS30 fusion protein;Ubiquitin-like protein FUBI;Small ribosomal subunit protein eS30;40S ribosomal protein S30	Fau
Uninfected	-1.88652096	0.00297273	Contactin-1	Cntn1
Uninfected	-1.88265133	0.00097734	PNMA family member 6E	Pnma6e
Uninfected	-1.88065033	1.2651E-05	Calpain small subunit 1	Capns1
Uninfected	-1.87695293	0.00053816	Nuclear ubiquitous casein and cyclin-dependent kinase substrate 1	Nucks1
Uninfected	-1.87148228	0.00024154	5(3)-deoxyribonucleotidase, cytosolic type	Nt5c
Uninfected	-1.8681778	1.8951E-07	Mitogen-activated protein kinase 3;Mitogen-activated protein kinase;mitogen-activated protein kinase	Mapk3
Uninfected	-1.86707382	0.00027376	Prostaglandin reductase 1	Ptgr1
Uninfected	-1.86662502	3.0089E-05	Protein mono-ADP-ribosyltransferase PARP3;Poly [ADP-ribose] polymerase	Parp3
Uninfected	-1.8649744	0.00029761	Hypoxanthine-guanine phosphoribosyltransferase	Hprt1
Uninfected	-1.86263103	1.4304E-06	14-3-3 protein epsilon	Ywhae
Uninfected	-1.85858574	0.00114971	Aldehyde oxidase 3	Aox3
Uninfected	-1.8571497	8.0341E-05	Protein phosphatase 1F	Ppm1f
Uninfected	-1.84747524	0.00011354	Bifunctional coenzyme A synthase;Phosphopantetheine adenylyltransferase;Dephospho-CoA kinase	Coasy
Uninfected	-1.8468811	0.01087543	Xaa-Pro dipeptidase	Pepd
Uninfected	-1.84631824	0.00097066	Cartilage oligomeric matrix protein	Comp
Uninfected	-1.84609585	0.00027744	Transaldolase	Taldo1
Uninfected	-1.84598274	0.0121728	WW domain-containing transcription regulator protein 1	Wwtr1
Uninfected	-1.84490318	1.392E-05	Coatomer subunit epsilon	Cope
Uninfected	-1.84106674	4.3384E-07	Adapter Sh3bgrl	Sh3bgrl
Uninfected	-1.83927879	0.00084459	Fatty acid-binding protein 5	Fabp5
Uninfected	-1.838521	1.5295E-06	Apolipoprotein N	Apon
Uninfected	-1.83769493	3.8848E-05	Inter-alpha-trypsin inhibitor heavy chain H2	Itih2
Uninfected	-1.83515053	0.00733849	Mitogen-activated protein kinase kinase kinase 20	Map3k20
Uninfected	-1.83258877	1.2887E-05	Coatomer subunit gamma-1	Copg1
Uninfected	-1.83125896	1.5689E-05	Osteoclast-stimulating factor 1	Ostf1
Uninfected	-1.82552834	2.3052E-06	Flavin-containing monooxygenase 3	Fmo3
Uninfected	-1.82222633	0.00214058	Phenylalanine--tRNA ligase beta subunit	Farsb
Uninfected	-1.82103996	0.00027386	Charged multivesicular body protein 4b	Chmp4b
Uninfected	-1.81921444	5.1375E-07	Elongation factor 1-delta	Eef1d
Uninfected	-1.81766014	0.00559928	UDP-glucuronosyltransferase 2A1;UDP-glucuronosyltransferase 2A2	Gm43638;Ugt2a1;Ugt2a2
Uninfected	-1.81615963	0.00239092	N-acetylgalactosamine kinase	Galk2
Uninfected	-1.8145153	2.0481E-05	Antithrombin-III	Serpinc1
Uninfected	-1.8135004	0.00059817	ADP-ribosylation factor-binding protein GGA1	Gga1
Uninfected	-1.80535164	0.00686513	Pyruvate dehydrogenase E1 component subunit alpha, testis-specific form, mitochondrial	Pdha2
Uninfected	-1.80532074	1.5236E-06	Leukotriene A-4 hydrolase	Lta4h
Uninfected	-1.80352516	0.00068296	Triadin	Trdn
Uninfected	-1.80017548	0.00407227	Diacylglycerol kinase alpha	Dgka
Uninfected	-1.7979393	5.6028E-06	Serine--tRNA ligase, cytoplasmic;serine--tRNA ligase	Sars1
Uninfected	-1.79037247	3.0225E-07	Argininosuccinate lyase	Asl
Uninfected	-1.78575668	9.1857E-05	Immunoglobulin kappa chain variable 8	Igkv8-21;Igkv8-27;Igkv8-30
Uninfected	-1.78409367	9.0345E-06	Vacuolar protein sorting-associated protein 26A	Vps26a
Uninfected	-1.78391743	1.1961E-06	Sorting nexin-3	Snx3
Uninfected	-1.78373642	0.00023595	Guanylate-binding protein 1	Gbp1;Gbp2b
Uninfected	-1.7830328	3.9356E-06	Fibroblast growth factor 1;Multifunctional fusion protein;Fibroblast growth factor 1;Fibroblast growth factor	Fgf1
Uninfected	-1.77747478	8.2505E-06	Protein SET	Set
Uninfected	-1.77502136	0.00059826	Zinc-alpha-2-glycoprotein	Azgp1
Uninfected	-1.77389927	6.7363E-07	Farnesyl pyrophosphate synthase	Fdps
Uninfected	-1.77350712	0.00122535	Small ribosomal subunit protein mS34	Mrps34
Uninfected	-1.76831532	0.0003673	Creatine kinase M-type	Ckm
Uninfected	-1.75851078	0.00921739	Coagulation factor IX;Coagulation factor IXa light chain;Coagulation factor IXa heavy chain	F9
Uninfected	-1.75788536	9.7789E-06	14-3-3 protein beta/alpha;14-3-3 protein beta/alpha, N-terminally processed;14-3-3 protein beta/alpha	Ywhab
Uninfected	-1.75413761	4.6473E-06	Phosphatidylinositol-glycan-specific phospholipase D	Gpld1
Uninfected	-1.75054893	6.5157E-05	Peptidyl-prolyl cis-trans isomerase FKBP3;peptidylprolyl isomerase	Fkbp3
Uninfected	-1.74768047	0.00027663	Ubiquitin-like modifier-activating enzyme ATG7	Atg7
Uninfected	-1.74197121	3.1307E-05	Guanylate-binding protein 7	Gbp7
Uninfected	-1.74057388	9.3853E-07	Coatomer subunit delta	Arcn1

Uninfected	-1.73864918	0.00535709	Platelet-activating factor acetylhydrolase IB subunit alpha2;1-alkyl-2-acetylglycerophosphocholine esterase	Pafah1b2
Uninfected	-1.73285828	0.0001149	Serine protease FAM111A	Fam111a
Uninfected	-1.73182697	1.0269E-07	Echinoderm microtubule-associated protein-like 1	Eml1
Uninfected	-1.73096161	7.2146E-06	Proteasome subunit beta type-8;Proteasome subunit beta	Psmb8
Uninfected	-1.72642822	1.1316E-07	Sorting nexin-1	Snx1
Uninfected	-1.72630196	0.00207051	Proteasome subunit beta type-3	Psmb3
Uninfected	-1.7209938	0.00058791	Guanylate kinase;guanylate kinase	Guk1
Uninfected	-1.71810951	0.00016866	Ribonuclease inhibitor	Rnh1
Uninfected	-1.71248646	0.00298493	Citrate synthase	Csl
Uninfected	-1.7121664	7.4809E-07	Calponin-2;Calponin	Cnn2
Uninfected	-1.71082897	8.732E-06	Maleylacetoacetate isomerase;maleylacetoacetate isomerase	Gstz1
Uninfected	-1.70676155	0.00160473	Synaptic vesicle membrane protein VAT-1 homolog	Vat1
Uninfected	-1.70531292	4.8895E-06	Fatty acid-binding protein, liver	Fabp1
Uninfected	-1.70367241	0.00016965	Guanylate-binding protein 2	Gbp2
Uninfected	-1.70033932	0.0060266	Ribose-phosphate pyrophosphokinase 2	Prps2
Uninfected	-1.69962273	0.00031968	Carboxypeptidase N subunit 2	Cpn2
Uninfected	-1.69819603	2.5465E-05	ADP-ribosylation factor 5	Arf5
Uninfected	-1.69507504	0.00108362	Carbonic anhydrase-related protein	Ca8
Uninfected	-1.69433537	0.0005022	C-C chemokine receptor type 1	Ccr1
Uninfected	-1.693116	3.5122E-08	Cytoplasmic dynein 1 heavy chain 1	Dync1h1
Uninfected	-1.6896843	0.0008266	Dynein light chain roadblock-type 1;Dynein light chain roadblock	Dynlrb1
Uninfected	-1.67416592	7.1421E-05	Dynactin subunit 2	Dctn2
Uninfected	-1.67316151	1.0213E-05	Histidine--tRNA ligase, cytoplasmic	Hars1
Uninfected	-1.67032871	0.00012691	EH domain-containing protein 2	Ehd2
Uninfected	-1.66998844	5.008E-06	ferroxidase;Ceruloplasmin	Cp
Uninfected	-1.66588898	0.00055542	DAZ-associated protein 1	Dazap1
Uninfected	-1.66204834	0.00664836	Platelet-activating factor acetylhydrolase IB subunit alpha1	Pafah1b3
Uninfected	-1.66035767	0.00018414	Hepatocyte growth factor activator;Hepatocyte growth factor activator short chain;Hepatocyte growth factor activator long chain	Hgfac
Uninfected	-1.65894184	9.4719E-07	Leukocyte elastase inhibitor A	Serpinb1a
Uninfected	-1.65821056	2.3912E-08	Guanylate cyclase soluble subunit alpha-1	Gucy1a1
Uninfected	-1.65759716	0.00012668	Beta-arrestin-1	Arrb1
Uninfected	-1.65425797	4.6539E-07	Peptidyl-prolyl cis-trans isomerase FKBP4;Peptidyl-prolyl cis-trans isomerase FKBP4, N-terminally processed;peptidylprolyl isomerase	Fkbp4
Uninfected	-1.65159798	0.00142405	Prefoldin subunit 5	Pfdn5
Uninfected	-1.65004158	0.00023039	AP-3 complex subunit mu-1	Ap3m1
Uninfected	-1.64910278	2.7185E-05	Serine/threonine-protein kinase N1	Pkn1
Uninfected	-1.64579754	8.4166E-05	14 kDa phosphohistidine phosphatase	Phpt1
Uninfected	-1.64558525	3.6066E-07	Triosephosphate isomerase	Tpi1
Uninfected	-1.64239464	7.0348E-07	Pulmonary surfactant-associated protein C	Sftpc
Uninfected	-1.63854961	0.00017385	Eukaryotic translation initiation factor 1;Eukaryotic translation initiation factor 1b	Eif1;Eif1b
Uninfected	-1.63785496	0.00015043	Queuosine 5-phosphate N-glycosylase/hydrolase	QNG1
Uninfected	-1.63590183	6.2334E-05	UTP--glucose-1-phosphate uridylyltransferase	Ugp2
Uninfected	-1.63360176	0.00282373	Dual specificity mitogen-activated protein kinase kinase 1	Map2k1
Uninfected	-1.63348255	3.3151E-05	Arf-GAP with coiled-coil, ANK repeat and PH domain-containing protein 2;Arf-GAP with coiled-coil, ANK repeat and PH domain-containing protein	Acap2
Uninfected	-1.63252525	1.1052E-07	Phosphoglycerate kinase 1;Phosphoglycerate kinase	Pgk1
Uninfected	-1.63007603	0.00248172	Plasminogen;Plasmin heavy chain A;Activation peptide;Angiostatin;Plasmin heavy chain A, short form;Plasmin light chain B	Plg
Uninfected	-1.62881222	8.9012E-05	GTP-binding protein SAR1b	Sar1b
Uninfected	-1.62124844	1.3243E-06	Glycerol-3-phosphate dehydrogenase 1-like protein	Gpd1l
Uninfected	-1.61995125	6.3718E-05	Signal transducer and activator of transcription;Signal transducer and activator of transcription 1	Stat1
Uninfected	-1.61899128	4.045E-05	Thioredoxin	Txn
Uninfected	-1.61831799	2.8746E-05	ATP-dependent 6-phosphofructokinase, liver type	Pfkl
Uninfected	-1.61579361	0.0038009	COMM domain-containing protein 3	Commd3
Uninfected	-1.61340504	0.00442094	ADP-ribosylation factor 6	Arf6
Uninfected	-1.61311035	0.00258024	Prostaglandin E synthase 3	Ptges3
Uninfected	-1.61199284	7.4395E-05	Spermidine synthase	Srm
Uninfected	-1.61036568	1.5972E-07	Protein Niban 2	Niban2
Uninfected	-1.60971451	3.0944E-05	Chloride intracellular channel protein 4	Clic4
Uninfected	-1.60767403	2.2547E-06	14-3-3 protein zeta/delta	Ywhaz
Uninfected	-1.60691376	0.00018896	Polypeptide N-acetylgalactosaminyltransferase 5	Galnt5
Uninfected	-1.60689812	0.00013148	Ubiquitin-conjugating enzyme E2 D2;Ubiquitin-conjugating enzyme E2 D3;E2 ubiquitin-conjugating enzyme	Ube2d2;Ube2d3;Ube2d2a
Uninfected	-1.59982395	0.00014959	Glucosamine-6-phosphate isomerase 2;Glucosamine-6-phosphate isomerase	Gnpda2
Uninfected	-1.5980114	0.00120334	Beta-enolase;phosphopyruvate hydratase	Eno3

Uninfected	-1.59553528	3.6981E-06	Latexin	Lxn	
Uninfected	-1.58913746	0.00019742	Glycine--tRNA ligase	Gars1	
Uninfected	-1.5887989	0.00010455	Ubiquitin-conjugating enzyme E2 Z	Ube2z	
Uninfected	-1.58689327	1.5187E-05	Transcription factor p65	Rela	
Uninfected	-1.58406696	0.00288489	p53-induced death domain-containing protein 1;PIDD-N;PIDD-C;PIDD-CC	Pidd1	
Uninfected	-1.58067226	0.00059085	AH receptor-interacting protein	Aip	
Uninfected	-1.58063431	6.0049E-05	Cofilin-1	Cfl1	
Uninfected	-1.58058128	1.1689E-06	Prostaglandin reductase 2	Ptgr2	
Uninfected	-1.57995548	0.00276792	Glycogen phosphorylase, muscle form;Alpha-1,4 glucan phosphorylase	Pygm	
Uninfected	-1.5788559	7.1548E-06	Ethylmalonyl-CoA decarboxylase	Echdc1	
Uninfected	-1.57645359	0.00270919	EH domain-containing protein 4	Ehd4	
Uninfected	-1.57533817	3.1377E-07	Glutamine synthetase;Palmitoyltransferase GLUL	Glul	
Uninfected	-1.57533226	3.2939E-05	Far upstream element-binding protein 1	Fubp1	
Uninfected	-1.57303944	0.00026364	Golgi reassembly-stacking protein 2	Gorasp2	
Uninfected	-1.56845589	0.00028613	14-3-3 protein gamma;14-3-3 protein gamma, N-terminally processed	Ywhag	
Uninfected	-1.56697865	0.00010491	Phosphatidylinositol transfer protein alpha isoform	Pitpna	
Uninfected	-1.56632767	0.00088773	Transmembrane glycoprotein NMB	Gpnmb	
Uninfected	-1.56531868	0.00028038	Ubiquitin-fold modifier 1	Ufm1	
Uninfected	-1.55955238	9.4308E-05	Signal transducer and activator of transcription 3;Signal transducer and activator of transcription	Stat3	
Uninfected	-1.55173149	6.7864E-05	Ubiquitin-conjugating enzyme E2 variant 1	Gm20431;Ube2v1	
Uninfected	-1.54974003	0.00032753	Protein N-terminal asparagine amidohydrolase	Ntan1	
Uninfected	-1.54651756	4.3513E-06	Protein phosphatase 1 regulatory subunit 7	Ppp1r7	
Uninfected	-1.54606552	0.00052119	H-2 class I histocompatibility antigen, Q10 alpha chain	H2-Q10	Q10
Uninfected	-1.54036846	0.00017196	PDZ and LIM domain protein 2	Pdlim2	
Uninfected	-1.53968544	4.9064E-06	Elongation factor 1-gamma	Eef1g	
Uninfected	-1.53954296	0.00048755	Asparagine--tRNA ligase, cytoplasmic;asparagine--tRNA ligase	Nars1	
Uninfected	-1.53942776	3.4161E-05	UMP-CMP kinase	Cmpk1	
Uninfected	-1.53814812	0.00018513	Phosphatidylethanolamine-binding protein 1;Hippocampal cholinergic neurostimulating peptide	Pebp1	
Uninfected	-1.53188648	2.3124E-07	6-phosphogluconate dehydrogenase, decarboxylating	Pgd	
Uninfected	-1.52853928	0.00031571	Tubulin-specific chaperone A	Tbca	
Uninfected	-1.52309589	1.4711E-05	Insulin-like growth factor-binding protein complex acid labile subunit	Igfals	
Uninfected	-1.52249851	0.0005564	Muscleblind-like protein 1;Muscleblind-like protein 2	Mbnl1;Mbnl2	
Uninfected	-1.51978474	4.1096E-05	Ubiquitin-like modifier-activating enzyme 1	Uba1	
Uninfected	-1.51912804	1.076E-07	Kinesin-1 heavy chain;Kinesin-like protein	Kif5b	
Uninfected	-1.51543808	0.00012013	Lipoma-preferred partner homolog	Lpp	
Uninfected	-1.50687199	2.0236E-05	High mobility group protein B1	Hmgb1	
Uninfected	-1.50453682	5.5816E-05	Aspartyl aminopeptidase	Dnpep	
Uninfected	-1.50214977	3.9643E-06	Tetratricopeptide repeat protein 21A	Ttc21a	
Uninfected	-1.50163136	3.3865E-05	Glycogen phosphorylase, liver form;Alpha-1,4 glucan phosphorylase	Pygl	
Uninfected	-1.50132294	0.00024858	Alpha-parvin	Parva	
Uninfected	-1.50129509	0.00074291	Immunoglobulin heavy chain variable 9	Ighv9-4;Ighv9-1;Ighv9-3;Ighv9-2	4
Uninfected	-1.49517021	1.8138E-05	Synaptobrevin homolog YKT6	Ykt6	
Uninfected	-1.49185925	9.036E-07	Testin	Tes	
Uninfected	-1.4893774	7.7376E-07	AP-1 complex subunit mu-2	Ap1m2	
Uninfected	-1.48563576	3.2034E-05	COP9 signalosome complex subunit 6	Cops6	
Uninfected	-1.48529644	2.3958E-05	Transforming protein RhoA;Rho-related GTP-binding protein RhoC	Rhoa;Rhoc	
Uninfected	-1.48523407	0.00039186	Proteasome subunit beta type-9	Psmb9	
Uninfected	-1.48159275	1.4379E-05	Septin-4	Septin4	
Uninfected	-1.48086796	3.8539E-06	Peroxiredoxin-1	Prdx1	
Uninfected	-1.47671509	1.0979E-05	L-lactate dehydrogenase A chain;L-lactate dehydrogenase	Ldha	
Uninfected	-1.47665386	1.4661E-06	Coatomer subunit beta	Copb2	
Uninfected	-1.46977043	3.4342E-06	DnaJ homolog subfamily A member 1	Dnaja1	
Uninfected	-1.44925003	1.2061E-05	Transcription elongation factor A protein 1;Transcription elongation factor	Tcea1	
Uninfected	-1.44757748	4.2254E-06	Coatomer subunit beta	Copb1	
Uninfected	-1.44606476	9.8678E-06	Actin-related protein 3	Actr3	
Uninfected	-1.44325752	6.8172E-06	Protein transport protein Sec31A	Sec31a	
Uninfected	-1.44163399	1.6703E-05	Proteasome activator complex subunit 2	Psme2	
Uninfected	-1.4384716	1.742E-05	Acetyl-CoA acetyltransferase, cytosolic;acetyl-CoA C-acetyltransferase	Acat2;Acat3	
Uninfected	-1.43378849	1.8336E-05	Ras GTPase-activating-like protein IQGAP1	Iqgap1	
Uninfected	-1.42492733	3.1559E-05	Ketimine reductase mu-crystallin	Crym	
Uninfected	-1.41680737	3.8107E-06	Switch-associated protein 70	Swap70	
Uninfected	-1.41286526	3.6914E-07	Cytoplasmic dynein 1 light intermediate chain 2;Dynein light intermediate chain	Dync1li2	
Uninfected	-1.41091137	2.3917E-05	Xaa-Pro aminopeptidase 1	Xpnpep1	
Uninfected	-1.40578289	4.0155E-05	V-type proton ATPase subunit H	Atp6v1h	

Uninfected	-1.40096798	4.3154E-05	Glyceraldehyde-3-phosphate dehydrogenase	Gapdh;Gapdhrt;Gapdhrt2
Uninfected	-1.39677067	7.3532E-07	A-kinase anchor protein 12	Akap12
Uninfected	-1.39661083	6.4084E-05	Glycogen synthase kinase-3 beta	Gsk3b
Uninfected	-1.3950592	1.2554E-06	Septin-2	Septin2
Uninfected	-1.39457245	3.1527E-05	Ribosyldihydronicotinamide dehydrogenase [quinone]	Nqo2
Uninfected	-1.39358101	6.3442E-08	Coatomer subunit alpha;Xenin;Proxenin;Coatomer subunit alpha	Copa
Uninfected	-1.39198971	1.7468E-06	Poly(rC)-binding protein 1	Pcbp1
Uninfected	-1.39047604	4.5992E-07	Vacuolar protein sorting-associated protein 35	Vps35
Uninfected	-1.38813305	7.8969E-07	Cullin-associated NEDD8-dissociated protein 1	Cand1
Uninfected	-1.38652992	2.2501E-05	Dynactin subunit 4	Dctn4
Uninfected	-1.38071117	3.0207E-07	Puromycin-sensitive aminopeptidase;Aminopeptidase	Npepps
Uninfected	-1.37463112	1.4257E-07	Valine--tRNA ligase;valine--tRNA ligase	Vars1
Uninfected	-1.36912518	1.8681E-05	Protein diaphanous homolog 1	Diaph1
Uninfected	-1.3285656	1.0543E-06	Adenylyl cyclase-associated protein 1	Cap1
Uninfected	-1.32710323	1.8304E-06	Vasodilator-stimulated phosphoprotein	Vasp
Uninfected	-1.29808826	3.4405E-08	Eukaryotic initiation factor 4A-I	Eif4a1
Latent	1.346557236	2.3396E-07	SUN domain-containing protein 2	Sun2
Latent	1.385668755	4.9408E-06	EMILIN-1	Emilin1
Latent	1.404438972	4.2607E-05	Regulator of chromosome condensation	Rcc1
Latent	1.411663055	2.1323E-06	Palmitoyltransferase ZDHHC18	Zdhhc18
Latent	1.431986237	2.6795E-05	Cytosolic iron-sulfur assembly component 2B	Ciao2b
Latent	1.433917427	6.0794E-05	Cathepsin D	Ctsd;Gm49369
Latent	1.436263657	0.00019519	Constitutive coactivator of peroxisome proliferator-activated receptor gamma	Fam120b
Latent	1.444517136	0.00015831	Alpha-actinin-2	Actn2
Latent	1.460106277	0.00021864	H/ACA ribonucleoprotein complex subunit 3	Nop10
Latent	1.461992645	1.6909E-06	Immunoglobulin kappa chain variable 6	Igkv6-32
Latent	1.469824219	7.3996E-05	Cytochrome b-245 chaperone 1;Essential for reactive oxygen species protein	Cybc1
Latent	1.476005745	0.00010946	Basement membrane-specific heparan sulfate proteoglycan core protein;Endorepellin;LG3 peptide	Hspg2
Latent	1.492490768	0.0006204	CTD small phosphatase-like protein 2	Ctdspl2
Latent	1.508160591	7.72E-05	Laminin subunit gamma-2	Lamc2
Latent	1.51581707	1.7631E-06	Collagen alpha-1(III) chain	Col3a1
Latent	1.534700012	9.6938E-06	Musculoskeletal embryonic nuclear protein 1	Mustn1
Latent	1.535124397	0.00024493	Ankyrin repeat and SOCS box protein 1	Asb1
Latent	1.548081779	0.00014779	Carbonic anhydrase 1	Ca1
Latent	1.55892334	0.00227392	Basic helix-loop-helix ARNT-like protein 1	Bmal1
Latent	1.576826859	1.1276E-05	Lamin-B1	Lmnb1
Latent	1.582398796	0.00115526	Keratin, type II cytoskeletal 1b	Krt77
Latent	1.590481758	0.00190469	Dual specificity mitogen-activated protein kinase kinase 5	Map2k5
Latent	1.594910049	0.00337639	Macrophage galactose N-acetyl-galactosamine specific lectin 2	Mgl2
Latent	1.597774696	0.00079436	Probable E3 ubiquitin-protein ligase TRIML1	Triml1
Latent	1.602639198	0.00208665	Diencephalon/mesencephalon homeobox protein 1	Dmbx1
Latent	1.605960274	0.00018286	Copine-9	Cpne9
Latent	1.607230949	0.00166431	Titin	Ttn
Latent	1.624636269	0.00428379	Cytochrome P450, family 4, subfamily a, polypeptide 29	Cyp4a29
Latent	1.631901741	0.00130004	Receptor-interacting serine/threonine-protein kinase 4	Ripk4
Latent	1.633724213	1.3444E-05	Immunoglobulin heavy chain variable 8	Ighv8-11
Latent	1.63822937	0.00288745	PHD finger protein 14	Phf14
Latent	1.651158714	0.00042484	Non-histone chromosomal protein HMG-17	Hmgn2
Latent	1.652779961	0.00320968	CEP295 N-terminal-like protein	Cep295nl
Latent	1.656334877	1.2726E-06	Dedicator of cytokinesis 9	Dock9
Latent	1.657310104	0.00137986	NACHT, LRR and PYD domains-containing protein 12	Nlrp12
Latent	1.65824604	0.00638439	H(+)/Cl(-) exchange transporter 5	Clcn5
Latent	1.670298195	0.00494687	Chondroadherin	Chad
Latent	1.670782089	0.00374236	Selection and upkeep of intraepithelial T-cells protein 5	Skint5
Latent	1.673212814	0.00037009	Pro-neuregulin-1, membrane-bound isoform;Pro-neuregulin-1, membrane-bound isoform;Neuregulin-1	Nrg1
Latent	1.686477089	0.00168264	Potassium channel tetramerisation domain containing 17	Kctd17
Latent	1.695353699	0.00741294	Keratin, type I cytoskeletal 15	Krt15
Latent	1.695983315	0.00015422	GA-binding protein subunit beta-2	Gabpb2
Latent	1.696976852	0.00629948	Myosin light chain 1/3, skeletal muscle isoform	Myl1
Latent	1.701415062	0.00101837	Coiled-coil domain-containing protein 96	Ccdc96
Latent	1.705030251	0.00632121	Solute carrier family 35 member F6	Slc35f6
Latent	1.705619049	0.00981467	Cardiac-enriched FHL2-interacting protein	CEFIP
Latent	1.710651398	0.00015006	Methyl-CpG-binding domain protein 1	Mbd1
Latent	1.72057476	3.0359E-05	TSC22 domain family protein 1	Tsc22d1
Latent	1.76037178	2.0064E-05	Trophoblast glycoprotein	Tpbg

Latent	1.76703949	0.00075496	U3 small nucleolar ribonucleoprotein protein MPP10	Mphosph10
Latent	1.7757864	0.01337477	V-set and immunoglobulin domain-containing protein 10	Vsig10
Latent	1.782867432	0.01607376	Coiled-coil domain containing 170	Ccdc170
Latent	1.800479698	0.01212242	Octanoyl-[acyl-carrier-protein]:protein N-octanoyltransferase LIPT2, mitochondrial	Lipt2
Latent	1.803296471	0.00406342	Alsin	Als2
Latent	1.804281807	0.00308633	Cilia- and flagella- associated protein 210	Cfap210
Latent	1.80668354	2.4447E-05	Immunoglobulin superfamily member 8	Igsf8
Latent	1.817729378	0.00056861	Chromobox protein homolog 1	Cbx1
Latent	1.818573189	0.01726963	Replication protein A 14 kDa subunit	Rpa3
Latent	1.821868324	0.01777585	GTP-binding protein REM 1	Rem1
Latent	1.823207664	0.0037982	Plakophilin-1	Pkp1
Latent	1.826121712	0.00254554	6-phosphofructo-2-kinase/fructose-2,6-bisphosphatase 4;6-phosphofructo-2-kinase;Fructose-2,6-bisphosphatase	Pfkfb4
Latent	1.827950859	0.00417154	Syntaxin-19	Stx19
Latent	1.831843948	9.742E-05	Flavin-containing monooxygenase	Fmo9
Latent	1.842987251	0.00428712	Mediator of RNA polymerase II transcription subunit 4	Med4
Latent	1.846253204	0.00014063	Methyltransferase N6AMT1	N6amt1
Latent	1.859226227	0.00031367	Collagen alpha-2(V) chain	Col5a2
Latent	1.861398888	0.00255956	LIX1-like protein	Lix1l
Latent	1.861517143	0.00117648	Phosphorylase b kinase gamma catalytic chain, liver/testis isoform	Phkg2
Latent	1.878991508	6.8421E-06	Metalloreductase STEAP4	Steap4
Latent	1.887376976	0.01509252	Growth/differentiation factor 10	Gdf10
Latent	1.896621895	3.2242E-05	Zinc finger BED domain-containing protein 6	Zbed6
Latent	1.921191216	0.00342594	Transcription factor IIIB 50 kDa subunit	Brf2
Latent	1.922575951	0.02162377	RNA-binding protein 38;RNA-binding protein 24	Rbm38;Rbm24
Latent	1.924333	0.02322876	Uncharacterized serine/threonine-protein kinase SBK3	Sbk3
Latent	1.939790916	0.00173758	tRNA-specific adenosine deaminase 1	Adat1
Latent	1.940017319	0.03066822	MORC family CW-type zinc finger protein 3	Morc3
Latent	1.942439461	4.3225E-06	Ig kappa chain V-VI region NQ5-78.2.6;Ig kappa chain V-VI region NQ6-8.3.1;Ig kappa chain V-VI region NQ2-48.2.2;Ig kappa chain V-VI region NQ2-17.4.1	Igkv4-70;Igkv4-59
Latent	1.946139908	0.01634358	Paired box protein Pax-7	Pax7
Latent	1.951999855	0.01660431	spermatogenesis associated glutamate (E)-rich protein	Gm1979;Speer4a3;Speer4a2
Latent	1.957589722	0.00780387	NEDD4 binding protein 2	N4bp2
Latent	1.96177578	0.02215039	Demilune cell and parotid protein	Dcpp1;Dcpp3;Dcpp2
Latent	1.966237259	0.02474597	UPF3 regulator of nonsense transcripts homolog A	Upf3a
Latent	1.968439674	0.0062808	Basic helix-loop-helix ARNT-like protein 2	Bmal2
Latent	1.971135902	0.00010827	Collagen alpha-2(I) chain	Col1a2
Latent	1.985049057	0.0030835	Axonemal dynein light intermediate polypeptide 1	Dnali1
Latent	1.985376549	0.00080847	M-phase phosphoprotein 6	Mphosph6
Latent	1.987307549	0.01269381	Collagen alpha-4(IV) chain	Col4a4
Latent	1.998327065	0.02384626	Histone H2A.J;Histone H2A;Histone H2A type 1-F;Histone H2A type 1-E;Histone H2A type 1-P;Histone H2A type 1-O;Histone H2A type 1-N;Histone H2A type 1-I;Histone H2A type 1-C;Histone H2A type 1-D;Histone H2A type 1-B;Histone H2A type 2-A;Histone H2A type 1-H;Histone H2A type 3;Histone H2A type 2-C;Histone H2A type 1-K;Histone H2A type 1-G	H2aj;Hist1h2af;H2ac8;Hist1h2ap;Hist1h2ao;Hist1h2an;H2ac13;H2ac6;H2ac7;H2ac4;Hist2h2aa1;H2ac12;H2ac1;H2ac25;H2ac20;H2ac15;H2ac11
Latent	2.000049591	0.00535697	Extracellular sulfatase Sulf-2;Extracellular sulfatase Sulf-2 secreted form	Sulf2
Latent	2.011363983	0.02861463	Solute carrier family 9 member C1	Slc9c1
Latent	2.012981033	0.03013421	Junctophilin-3	Jph3
Latent	2.020479584	0.00122197	Ubiquitin-associated protein 1	Ubap1
Latent	2.061275482	0.00494578	Bone marrow proteoglycan;Eosinophil granule major basic protein	Prg2
Latent	2.061878204	0.00198939	Transmembrane protein 8B	Tmem8b
Latent	2.062418747	0.04260843	E3 ubiquitin-protein ligase TRIM31	Trim31
Latent	2.077910805	0.01746839	RNA-binding protein 43	Rbm43
Latent	2.079833984	0.01787138	Rho GTPase-activating protein 8	Arhgap8
Latent	2.087350273	0.0014421	Telomere-associated protein RIF1	Rif1
Latent	2.10661869	7.6451E-08	T-cell activation Rho GTPase-activating protein;T-cell activation GTPase-activating protein 1	Tagap;Tagap1
Latent	2.106740379	0.00686441	Interferon gamma receptor 1	Ifngr1
Latent	2.106768036	0.01689155	Galactocerebrosidase	Galc
Latent	2.138802528	0.02351746	Ephrin type-B receptor 6	Ephb6
Latent	2.140569687	9.9497E-06	4-hydroxybenzoate polyprenyltransferase, mitochondrial	Coq2
Latent	2.146634865	0.00107336	Protein FAM149A	Fam149a

Latent	2.161307526	0.01966667	Interleukin-36 gamma	Il36g
Latent	2.171557236	0.00648736	StAR-related lipid transfer protein 5	Stard5
Latent	2.180994225	3.7291E-06	Profilin-2;Profilin	Pfn2
Latent	2.184324265	0.00431345	Sphingosine 1-phosphate receptor 5	S1pr5
Latent	2.184870338	0.00014794	Collagen alpha-1(I) chain	Col1a1
Latent	2.187132645	0.03248489	Collagen and calcium-binding EGF domain-containing protein 1	Ccbe1
Latent	2.188357544	0.05724247	Uncharacterized protein CXorf38 homolog	Q8C5K5;Q8C5K5-2
Latent	2.197709656	0.02399404	Disintegrin and metalloproteinase domain-containing protein 22	Adam22
Latent	2.209891701	0.04900925	Fasciculation and elongation protein zeta-2	Fez2
Latent	2.217987251	0.00149091	Phosphoinositide 3-kinase adapter protein 1	Pik3ap1
Latent	2.252067566	0.00056177	Pyroglutamyl-peptidase 1;Pyroglutamyl-peptidase I	Pgpep1
Latent	2.278652763	0.00769673	Protein Smaug homolog 2	Samd4b
Latent	2.27937088	0.02627947	Cyclin-L1	Ccnl1
Latent	2.285359764	3.2305E-05	EEF1A lysine methyltransferase 2	Eef1akmt2
Latent	2.285678291	0.04155387	RILP-like protein 2	Rilpl2
Latent	2.291149902	0.0177269	Ankyrin repeat domain-containing protein 27	Ankrd27
Latent	2.291926765	0.05407505	Macrophage-stimulating protein receptor;Macrophage-stimulating protein receptor alpha chain;Macrophage-stimulating protein receptor beta chain	Mst1r
Latent	2.310064888	0.04647732	Cerebral cavernous malformations 2 protein-like	Ccm2l
Latent	2.328302193	0.03731178	Nuclear distribution protein nudE-like 1	Ndel1
Latent	2.330469322	0.03103257	Zinc finger MYND domain-containing protein 11	Zmynd11
Latent	2.330596352	0.02755625	Immunoglobulin superfamily DCC subclass member 3	Igdcc3
Latent	2.331829834	0.0277195	Atlastin-1	Atl1
Latent	2.350478172	0.00094647	PWWP domain-containing protein 2B	Pwwp2b
Latent	2.369155884	0.04747988	Small ribosomal subunit protein uS2m	Mrps2
Latent	2.404800987	0.07544535	Pseudouridylate synthase PUS7L	Pus7l
Latent	2.469631004	0.00237938	Chitinase-like protein 4	Chil4
Latent	2.473347855	0.00292224	Fucose-1-phosphate guanylyltransferase	Fpgt
Latent	2.476032448	0.00010082	Paired-Ig-like receptor A12	Pira12
Latent	2.492004776	6.7351E-05	ADP-ribosylation factor-like protein 6	Arl6
Latent	2.493194008	2.9049E-05	Eosinophil peroxidase;Eosinophil peroxidase light chain;Eosinophil peroxidase heavy chain	Epx
Latent	2.509503555	0.03800342	Coiled-coil domain-containing protein 62	Ccdc62
Latent	2.541968727	0.0106325	Doublecortin domain containing 2C	Dcdc2c
Latent	2.568329239	0.03477924	F-box only protein 40	Fbxo40
Latent	2.568581581	0.00330823	Golgi phosphoprotein 3-like	Golph3l
Latent	2.609721184	0.00041144	Voltage-dependent L-type calcium channel subunit alpha;Voltage-dependent L-type calcium channel subunit alpha-1F	Cacna1f
Latent	2.691983986	0.00205168	Serum response factor	Srf
Latent	2.710082054	0.00271915	AT-rich interactive domain-containing protein 3B;AT-rich interactive domain-containing protein 3	Arid3b
Latent	2.744406319	0.05339583	Uncharacterized protein C7orf50 homolog	3110082I17Rik
Latent	2.812148285	0.00155863	Growth/differentiation factor 5	Gdf5
Latent	2.820663071	0.00693082	DDB1- and CUL4-associated factor 6	Dcaf6
Latent	2.884078979	0.00093153	Prostamide/prostaglandin F synthase	Prxl2b
Latent	2.890505219	0.00038716	Intelectin-1a	Itln1
Latent	2.928928185	0.00037264	G protein pathway suppressor 2	Gps2
Latent	3.009933853	2.0414E-07	Chitinase-like protein 3	Chil3
Latent	3.063491631	0.02927085	Centrosomal protein of 78 kDa	Cep78
Latent	3.146261978	0.02608538	Polyphosphoinositide phosphatase	Fig4
Latent	3.180517769	0.00451239	N-terminal Xaa-Pro-Lys N-methyltransferase 1;N-terminal Xaa-Pro-Lys N-methyltransferase 1, N-terminally processed;N-terminal Xaa-Pro-Lys N-methyltransferase 1	Ntmt1
Latent	3.466514397	0.01312788	Cell cycle exit and neuronal differentiation protein 1	Cend1
Latent	3.841226578	5.4225E-08	Pulmonary surfactant-associated protein D	Sftpd
Latent	3.92734642	1.9031E-07	Synaptotagmin-16	Syt16
Latent	3.941350174	0.03048746	Predicted gene	Gm17349
Latent	4.337623215	2.3483E-06	Calcium-activated chloride channel regulator 1	Clca1
Latent	5.330669212	3.2511E-07	Resistin-like alpha	Retnla

Murine Proteins: Hypervirulent versus Uninfected				
**Enriched in:**	**Fold Change**	**p-value**	**Protein Name**	**Gene**
Uninfected	-3.407803917	0.003312516	DNA-binding protein inhibitor ID-1	Id1
Uninfected	-2.826295471	0.000727135	Platelet glycoprotein Ib beta chain	Gp1bb
Uninfected	-2.264973068	0.00226099	Protein eva-1 homolog B	Eva1b
Uninfected	-2.123908234	0.001604525	Aquaporin-5	Aqp5
Uninfected	-2.117045212	0.001626523	Lysine-specific demethylase 3A;Lysine-specific demethylase	Kdm3a
Uninfected	-2.009379578	3.7259E-05	Cytochrome P450 2F2	Cyp2f2
Uninfected	-1.937247467	0.001769135	ubiquitinyl hydrolase 1	Usp35
Uninfected	-1.815440559	0.001040804	Apolipoprotein C-IV	Apoc4
Uninfected	-1.806498909	0.000170702	UDP-glucuronosyltransferase 2A1;UDP-glucuronosyltransferase 2A2	Gm43638;Ugt2a1;Ugt2a2
Uninfected	-1.798660278	0.00140866	Hepatocyte growth factor activator;Hepatocyte growth factor activator short chain;Hepatocyte growth factor activator long chain	Hgfac
Uninfected	-1.792180061	1.2163E-05	Serum paraoxonase/arylesterase 1;Paraoxonase	Pon1
Uninfected	-1.790593529	0.001139106	Transmembrane protein 14C	Tmem14c
Uninfected	-1.755235672	5.12796E-05	Apolipoprotein A-II;Proapolipoprotein A-II	Apoa2
Uninfected	-1.59520874	0.00010851	Cytochrome P450 4B1	Cyp4b1
Uninfected	-1.588695335	0.000556976	Ephrin-B1;Ephrin-B1 C-terminal fragment;Ephrin-B1 intracellular domain	Efnb1
Uninfected	-1.53457756	3.12935E-05	Flavin-containing monooxygenase 3	Fmo3
Uninfected	-1.4687006	5.41495E-05	Atrial natriuretic peptide receptor 3	Npr3
Hypervirulent	1.26809845	3.9025E-05	Transferrin receptor protein 1	Tfrc
Hypervirulent	1.364987373	1.48641E-05	Macrophage mannose receptor 1	Mrc1
Hypervirulent	1.374203873	9.48944E-06	Pyrin and HIN domain-containing protein 1;Pyrin and HIN domain-containing protein 1-like	Pyhin1;Ifi214;Ifi209
Hypervirulent	1.379395485	0.000125873	Zinc finger, MYM-type 6	Zmym6
Hypervirulent	1.403589821	1.031E-06	Dedicator of cytokinesis protein 2	Dock2
Hypervirulent	1.413978577	0.000121973	Scinderin	Scin
Hypervirulent	1.415878677	3.72466E-06	Protein disulfide-isomerase A4	Pdia4
Hypervirulent	1.42399807	0.000138825	Unconventional myosin-If	Myo1f
Hypervirulent	1.430512428	8.05365E-05	Creatine kinase U-type, mitochondrial	Ckmt1
Hypervirulent	1.461199188	1.04233E-06	Glutathione S-transferase omega-1;Glutathione S-transferase omega	Gsto1
Hypervirulent	1.555813217	0.000103883	Large ribosomal subunit protein eL28	Rpl28
Hypervirulent	1.563794899	0.000177435	Tyrosine-protein phosphatase non-receptor type 6	Ptpn6
Hypervirulent	1.585313225	0.000164375	Transmembrane emp24 domain-containing protein 3	Tmed3
Hypervirulent	1.602909851	2.03802E-05	Neutrophil cytosol factor 2	Ncf2
Hypervirulent	1.626453018	1.36687E-06	Metalloreductase STEAP4	Steap4
Hypervirulent	1.664585495	0.000794091	H-2 class II histocompatibility antigen, E-K alpha chain;H-2 class II histocompatibility antigen, E-D alpha chain	P04224;A0A494B9M4;P01904;P14439
Hypervirulent	1.684173012	0.000224944	Macrophage-capping protein	Capg
Hypervirulent	1.700726318	0.000108946	Polyunsaturated fatty acid lipoxygenase ALOX15	Alox15
Hypervirulent	1.750772858	9.83523E-05	Beta-hexosaminidase subunit beta	Hexb
Hypervirulent	1.761365318	2.41653E-05	C-X-C motif chemokine 15	Cxcl15
Hypervirulent	1.762282944	0.001145835	Stathmin-2	Stmn2
Hypervirulent	1.765148354	0.0005798	SH3 domain-binding glutamic acid-rich-like protein 3	Sh3bgrl3
Hypervirulent	1.850983429	0.000895362	Allograft inflammatory factor 1	Aif1
Hypervirulent	1.851555634	1.6957E-06	Hematopoietic lineage cell-specific protein	Hcls1
Hypervirulent	1.852338791	0.000940716	Brain acid soluble protein 1	Basp1
Hypervirulent	1.878590012	8.25976E-06	Mucin 5, subtype B, tracheobronchial	Muc5b
Hypervirulent	1.904432678	0.000146329	Lysophosphatidylcholine acyltransferase 2	Lpcat2
Hypervirulent	1.916991234	0.001913486	Acidic mammalian chitinase	Chia;Chia1
Hypervirulent	1.945803642	7.62633E-06	Coronin-1A;Coronin	Coro1a
Hypervirulent	1.970275688	1.40212E-05	H-2 class II histocompatibility antigen, A-K beta chain;H-2 class II histocompatibility antigen, A-F beta chain;H-2 class II histocompatibility antigen, A-U beta chain;H-2 class II histocompatibility antigen, A beta chain	P06343;P06346;P06344;P14483;P06345;P01921;A0A494B9U2;A0A494B9G5
Hypervirulent	2.006721497	0.001257674	Immunity-related GTPase family M protein 2	Irgm2
Hypervirulent	2.010553741	0.000149822	Oxidized low-density lipoprotein receptor 1;Oxidized low-density lipoprotein receptor 1, soluble form	Olr1
Hypervirulent	2.055690193	0.000631001	CD177 antigen	Cd177
Hypervirulent	2.078791046	0.000132941	Legumain	Lgmn
Hypervirulent	2.093294525	0.000128401	Rho GDP-dissociation inhibitor 2	Arhgdib
Hypervirulent	2.110425186	0.001336269	Interferon-induced protein 44	Ifi44
Hypervirulent	2.148504639	1.40509E-05	BPI fold-containing family B member 1	Bpifb1
Hypervirulent	2.162637329	4.01447E-05	Integrin alpha-M	Gm49368;Itgam
Hypervirulent	2.183550453	3.93215E-05	Galectin-3;Galectin	Lgals3
Hypervirulent	2.184448814	1.35299E-06	Fc fragment of IgG binding protein	Fcgbp
Hypervirulent	2.20724411	7.58316E-06	Ras-related C3 botulinum toxin substrate 2	Rac2
Hypervirulent	2.24394455	1.72823E-06	Integrin beta-2;Integrin beta	Itgb2
Hypervirulent	2.251399803	2.29759E-05	Polymeric immunoglobulin receptor;Secretory component	Pigr
Hypervirulent	2.324294281	8.63729E-06	Neutrophil gelatinase-associated lipocalin	Lcn2
Hypervirulent	2.324385834	6.27341E-07	Chitinase-3-like protein 1	Chi3l1
Hypervirulent	2.327884865	0.000119219	Macrosialin	Cd68
Hypervirulent	2.339541626	3.27314E-07	Cytochrome b-245 heavy chain	Cybb
Hypervirulent	2.362221336	9.26553E-05	Neutrophil elastase	Elane
Hypervirulent	2.402619743	0.000136287	Macrophage galactose N-acetyl-galactosamine specific lectin 2	Mgl2
Hypervirulent	2.425754738	2.45598E-05	Protein S100-A9	S100a9
Hypervirulent	2.48315506	0.000110042	Cathepsin Z	Ctsz
Hypervirulent	2.520822716	0.000210418	Procathepsin L;Cathepsin L;Cathepsin L heavy chain;Cathepsin L light chain	Ctsl
Hypervirulent	2.552942085	1.90285E-05	Low affinity immunoglobulin gamma Fc region receptor II	Fcgr2;Fcgr2b
Hypervirulent	2.665180206	1.10457E-06	Myeloperoxidase;Myeloperoxidase light chain;Myeloperoxidase heavy chain	Mpo
Hypervirulent	2.665421486	0.001556496	Aryl hydrocarbon receptor nuclear translocator	Arnt
Hypervirulent	2.766335106	4.54853E-05	Anterior gradient protein 2 homolog	Agr2
Hypervirulent	2.865119362	7.5427E-06	Arginase-1	Arg1
Hypervirulent	2.981369591	1.85215E-06	Lactotransferrin	Ltf
Hypervirulent	3.01776104	7.91438E-05	Myeloblastin	Prtn3
Hypervirulent	3.023199844	0.000820773	Eosinophil cationic protein 2	Ear2
Hypervirulent	3.07788105	0.00999593	Eosinophil-associated, ribonuclease A family, member 6	Ear6
Hypervirulent	3.17506485	8.03983E-09	Pulmonary surfactant-associated protein D	Sftpd
Hypervirulent	3.218737984	0.004510505	Eosinophil-associated, ribonuclease A family, member 10	Ear10
Hypervirulent	3.285422516	3.01369E-06	Neutrophilic granule protein	Ngp
Hypervirulent	3.309161186	2.66779E-05	Synaptotagmin-16	Syt16
Hypervirulent	3.505771065	0.000438791	Bone marrow proteoglycan;Eosinophil granule major basic protein	Prg2
Hypervirulent	3.542105675	6.3461E-05	Intelectin-1a	Itln1
Hypervirulent	4.290656853	1.97564E-05	Eosinophil peroxidase;Eosinophil peroxidase light chain;Eosinophil peroxidase heavy chain	Epx
Hypervirulent	4.840749931	8.19042E-11	Chitinase-like protein 3	Chil3
Hypervirulent	5.022075653	2.10324E-07	Chitinase-like protein 4	Chil4
Hypervirulent	5.892672348	2.04502E-08	Calcium-activated chloride channel regulator 1	Clca1
Hypervirulent	7.515281296	8.50057E-10	Resistin-like alpha	Retnla

Murine Proteins: Latent versus Typical				
**Enriched in:**	**Fold Change**	**p-value**	**Protein Name**	**Gene**
Typical	-5.141526837	4.58383E-10	Arginase-1	Arg1
Typical	-4.222981622	2.44985E-13	Glutathione S-transferase omega-1;Glutathione S-transferase omega	Gsto1
Typical	-4.092154672	1.76864E-06	Eosinophil-associated ribonuclease 6	Ear6
Typical	-3.935868602	7.92568E-08	Protein S100-G	S100g
Typical	-3.915180779	5.83146E-08	Thymosin beta-10;Thymosin beta-4;Hemoregulatory peptide AcSDKP	Tmsb10;Tmsb4x
Typical	-3.818039491	4.89589E-08	Prothymosin alpha;Prothymosin alpha, N-terminally processed;Thymosin alpha	Ptma
Typical	-3.765082105	8.04082E-11	L-lactate dehydrogenase B chain;L-lactate dehydrogenase	Ldhb
Typical	-3.725562943	5.466E-07	Chitinase-like protein 4	Chil4
Typical	-3.684694693	6.19632E-10	Myoglobin	Mb
Typical	-3.617526542	3.38406E-11	Serotransferrin	Tf
Typical	-3.540346824	6.87455E-07	Leucine-rich repeats and immunoglobulin-like domains protein 1	Lrg1
Typical	-3.515428925	3.24687E-13	m7GpppX diphosphatase	Dcps
Typical	-3.505584568	4.51646E-06	Eosinophil-associated ribonuclease 10	Ear10
Typical	-3.504903242	8.08631E-12	Phosphoglycerate mutase 1	Pgam1
Typical	-3.481881248	2.11395E-08	Alpha-2-HS-glycoprotein	Ahsg
Typical	-3.431919649	0.000196543	Ankyrin repeat domain-containing protein 49	Ankrd49
Typical	-3.419183625	1.5459E-11	Hemopexin	Hpx
Typical	-3.350293308	2.88672E-18	Fatty acid synthase;[Acyl-carrier-protein] S-acetyltransferase;[Acyl-carrier-protein] S-malonyltransferase;3-oxoacyl-[acyl-carrier-protein] synthase;3-oxoacyl-[acyl-carrier-protein] reductase;3-hydroxyacyl-[acyl-carrier-protein] dehydratase;Enoyl-[acyl-carrier-protein] reductase;Acyl-[acyl-carrier-protein] hydrolase;Fatty acid synthase	Fasn
Typical	-3.329253578	8.07759E-08	Eosinophil peroxidase;Eosinophil peroxidase light chain;Eosinophil peroxidase heavy chain	Epx
Typical	-3.323896747	3.66259E-12	Albumin	Alb
Typical	-3.30747384	3.1482E-08	Astrocytic phosphoprotein PEA-15	Pea15;Pea15a
Typical	-3.303427717	1.15676E-06	Eosinophil cationic protein 2	Ear2
Typical	-3.286973657	5.20883E-09	Carboxylic ester hydrolase	Ces1b
Typical	-3.26228608	0.001155689	ATP synthase subunit O, mitochondrial	Atp5po
Typical	-3.233976512	1.394E-08	Coactosin-like protein	Cotl1
Typical	-3.226155345	2.16925E-05	Ig gamma-1 chain C region secreted form;Ig gamma-1 chain C region, membrane-bound form	Ighg1
Typical	-3.218043984	4.97896E-05	Fetuin-B	Fetub
Typical	-3.20437105	8.83419E-08	Carboxylesterase 1C	Ces1c
Typical	-3.202596622	5.77571E-11	Argininosuccinate synthase	Ass1
Typical	-3.192892647	1.97839E-09	Fumarylacetoacetase	Fah
Typical	-3.16876439	2.22527E-11	Cytosolic non-specific dipeptidase	Cndp2
Typical	-3.16395323	1.48749E-09	NADP-dependent malic enzyme;Malic enzyme	Me1
Typical	-3.148893505	5.26211E-06	Protein S100-A9	S100a9
Typical	-3.123383586	1.15873E-10	Macrophage-capping protein	Capg
Typical	-3.121533394	9.7015E-07	Creatine kinase B-type	Ckb
Typical	-3.105895784	1.06152E-12	Elongation factor 2	Eef2
Typical	-3.077511448	9.66247E-10	Plastin-2	Lcp1
Typical	-3.062857098	5.22038E-06	Profilin-1;Profilin	Pfn1
Typical	-3.061943711	6.84147E-15	Transketolase;transketolase	Tkt
Typical	-3.054265573	2.34632E-06	Protein S100-A6	S100a6
Typical	-3.050297292	8.81617E-13	Xanthine dehydrogenase/oxidase;Xanthine dehydrogenase;Xanthine oxidase	Xdh
Typical	-3.050201586	1.11223E-10	Coronin-1A;Coronin	Coro1a
Typical	-3.024853219	6.06418E-09	Ribonuclease inhibitor	Rnh1
Typical	-3.01880614	5.31072E-09	Rho GDP-dissociation inhibitor 2	Arhgdib
Typical	-3.002721193	1.07909E-08	Kininogen-1;Kininogen-1 heavy chain;Bradykinin;Kininogen-1 light chain	Kng1
Typical	-2.963333935	5.3616E-12	Pyruvate kinase PKM	Pkm
Typical	-2.952772967	4.72593E-08	Transaldolase	Taldo1
Typical	-2.951864137	3.52828E-11	Complement C2;Complement factor B;Complement factor B Ba fragment;Complement factor B Bb fragment;Complement factor B	Gm20547;Cfb
Typical	-2.939669185	8.50411E-10	Serine (or cysteine) peptidase inhibitor, clade B, member 9	Serpinb9
Typical	-2.924562518	1.56578E-05	Serum amyloid P-component	Apcs
Typical	-2.906445927	7.55278E-09	SEC14-like lipid binding 3	Sec14l3

Typical	-2.90524398	1.58281E-08	Ferritin light chain 1;Ferritin;Ferritin light chain 2	Ftl1;Ftl2
Typical	-2.901915487	1.75163E-10	Hepatoma-derived growth factor	Hdgf
Typical	-2.868949657	4.66548E-12	Leukocyte elastase inhibitor A	Serpinb1a
Typical	-2.86591604	3.8935E-05	Immunoglobulin heavy constant alpha	Igha
Typical	-2.861376826	0.000318815	Protein S100-A8	S100a8
Typical	-2.85511375	9.69746E-09	Putative protein-lysine deacylase ABHD14B	Abhd14b
Typical	-2.851243507	1.48007E-05	Cystatin-B	Cstb
Typical	-2.850363689	3.3595E-09	Chitinase-3-like protein 1	Chi3l1
Typical	-2.846068192	6.19501E-10	Pregnancy zone protein;Alpha-2-macroglobulin 165 kDa subunit;Alpha-2-macroglobulin 35 kDa subunit	Pzp
Typical	-2.844727622	1.41854E-06	Fatty acid-binding protein 5	Fabp5
Typical	-2.843395382	7.02307E-08	Glucose-6-phosphate isomerase	Gpi
Typical	-2.826477856	5.96573E-05	Bone marrow proteoglycan;Eosinophil granule major basic protein	Prg2
Typical	-2.824503941	1.16515E-09	Acidic leucine-rich nuclear phosphoprotein 32 family member A;Acidic leucine-rich nuclear phosphoprotein 32 family member	Anp32a
Typical	-2.821140162	8.05372E-07	Cytosolic acyl coenzyme A thioester hydrolase	Acot7
Typical	-2.820500565	8.17083E-09	Myotrophin	Mtpn
Typical	-2.792280918	4.40696E-09	L-xylulose reductase	Dcxr
Typical	-2.769010925	2.09543E-10	Isocitrate dehydrogenase [NADP] cytoplasmic	Idh1
Typical	-2.747998322	1.2613E-06	Ig heavy chain V region 914	P18527
Typical	-2.746498828	1.44281E-10	Chloride intracellular channel protein 1	Clic1
Typical	-2.743742561	7.4891E-07	Transthyretin	Ttr
Typical	-2.739943674	1.76064E-08	Ubiquitin-like modifier-activating enzyme ATG7	Atg7
Typical	-2.731444634	1.42595E-10	Aldo-keto reductase family 1 member B1	Akr1b1
Typical	-2.717217488	2.92795E-08	Acidic leucine-rich nuclear phosphoprotein 32 family member B	Anp32b
Typical	-2.714319738	1.49865E-10	Leukotriene A-4 hydrolase	Lta4h
Typical	-2.713661321	2.00713E-14	Vitamin D-binding protein	Gc
Typical	-2.708197912	5.60452E-06	Ig lambda-1 chain C region	Iglc1
Typical	-2.69170439	1.13022E-11	Deoxynucleoside triphosphate triphosphohydrolase SAMHD1	Samhd1
Typical	-2.687912348	2.9776E-10	Cytoplasmic aconitate hydratase	Aco1
Typical	-2.685856756	1.10349E-08	Indolethylamine N-methyltransferase	Inmt
Typical	-2.684549798	7.63746E-10	Aldo-keto reductase family 1 member A1	Akr1a1
Typical	-2.679384952	7.61818E-07	Acidic mammalian chitinase	Chia;Chia1
Typical	-2.650956175	1.05543E-06	Ester hydrolase C11orf54 homolog	4931406C07Rik
Typical	-2.647788599	0.000136246	Protein N-terminal asparagine amidohydrolase	Ntan1
Typical	-2.636225594	6.55565E-12	Tyrosine-protein phosphatase non-receptor type 6	Ptpn6
Typical	-2.631728554	0.001200527	RNA exonuclease 4	Rexo4
Typical	-2.627405696	8.75684E-08	Glutathione S-transferase Mu 1;Glutathione S-transferase;glutathione transferase	Gstm1
Typical	-2.625151486	2.07184E-09	Aldehyde dehydrogenase, cytosolic 1	Aldh1a7
Typical	-2.616519038	2.74628E-06	Polyunsaturated fatty acid lipoxygenase ALOX15	Alox15
Typical	-2.612421099	1.83325E-07	Ferritin heavy chain;Ferritin heavy chain, N-terminally processed;Ferritin	Fth1
Typical	-2.61133052	8.8628E-09	Peptidyl-prolyl cis-trans isomerase FKBP1A	Fkbp1a
Typical	-2.605719482	1.21199E-09	Thioredoxin reductase 1, cytoplasmic	Txnrd1
Typical	-2.604143482	1.15648E-05	Corticosteroid-binding globulin	Serpina6
Typical	-2.590909619	2.94619E-06	Glutathione S-transferase A3	Gsta3
Typical	-2.586036004	0.000147481	Alpha-1-antitrypsin 1-2	Serpina1b
Typical	-2.585694419	6.30026E-09	Chitinase-like protein 3	Chil3
Typical	-2.583923064	9.0688E-12	6-phosphogluconate dehydrogenase, decarboxylating	Pgd
Typical	-2.578653611	0.002569511	Protein diaphanous homolog 3	Diaph3
Typical	-2.578537538	2.44E-05	Adenylate kinase isoenzyme 1;nucleoside-triphosphate--adenylate kinase	Ak1
Typical	-2.575522232	9.44933E-05	Interleukin-1 receptor antagonist protein;Interleukin-1	Il1rn
Typical	-2.571703296	1.05812E-06	Ras-related C3 botulinum toxin substrate 2	Rac2
Typical	-2.542107561	4.96019E-08	Fatty acid-binding protein, adipocyte	Fabp4
Typical	-2.533630011	4.1814E-08	Large ribosomal subunit protein eL38	Rpl38
Typical	-2.530825551	8.16312E-10	Peroxiredoxin-6	Prdx6
Typical	-2.529672517	0.0191173	Family with sequence similarity 186, member B	Fam186b
Typical	-2.525469017	3.15178E-08	Phosphoglucomutase-1	Pgm1
Typical	-2.52447016	3.97264E-07	Protein SET	Set
Typical	-2.524228287	2.36318E-05	Charged multivesicular body protein 4b	Chmp4b
Typical	-2.518096987	3.08924E-06	Parathymosin	Ptms

Typical	-2.496956889	8.77742E-06	Serine protease inhibitor A3K	Serpina3k
Typical	-2.489764531	3.67932E-07	Cytoplasmic dynein 1 heavy chain 1	Dync1h1
Typical	-2.475421333	3.37122E-11	ATP-citrate synthase	Acly
Typical	-2.474956152	1.29135E-08	Annexin A3	Anxa3
Typical	-2.464537027	3.66232E-09	Glycogen phosphorylase, liver form;Alpha-1,4 glucan phosphorylase	Pygl
Typical	-2.463923836	1.75767E-09	Pterin-4-alpha-carbinolamine dehydratase	Pcbd1
Typical	-2.463493729	0.027826562	Bromodomain-containing protein 8	Brd8
Typical	-2.444336383	1.02454E-07	Osteoclast-stimulating factor 1	Ostf1
Typical	-2.443476889	2.21045E-10	Aspartate aminotransferase, cytoplasmic	Got1
Typical	-2.440988435	0.0043464	Tubulin beta-3 chain	Tubb3
Typical	-2.435474883	0.001188531	Ribonuclease, RNase A family, 2A	Rnase2a
Typical	-2.427513991	1.17335E-08	Coatomer subunit gamma-1	Copg1
Typical	-2.425614357	4.22835E-08	Glutathione S-transferase Mu 2;Glutathione S-transferase	Gstm2
Typical	-2.419755024	3.65239E-08	Immunoglobulin kappa variable 14-126	Igkv14-126
Typical	-2.41521776	5.0027E-06	Serine (Or cysteine) peptidase inhibitor, clade B, member 6B	Serpinb6b
Typical	-2.413996103	3.88201E-07	Hypoxanthine-guanine phosphoribosyltransferase	Hprt1
Typical	-2.411503474	1.10077E-06	Afamin	Afm
Typical	-2.407039558	8.42873E-11	Triosephosphate isomerase	Tpi1
Typical	-2.395624797	4.11949E-08	ADP-ribosylation factor 4	Arf4
Typical	-2.394352362	1.8081E-06	Adenylosuccinate synthetase isozyme 2	Adss2
Typical	-2.385360146	1.2116E-07	Carbonyl reductase [NADPH] 3	Cbr3
Typical	-2.378796344	5.32493E-06	Brain acid soluble protein 1	Basp1
Typical	-2.378303337	2.41179E-09	UTP--glucose-1-phosphate uridylyltransferase	Ugp2
Typical	-2.37743543	4.13279E-07	Thioredoxin domain-containing protein 17	Txndc17
Typical	-2.376429388	0.000943423	Costars family protein ABRACL	Abracl
Typical	-2.371551768	1.92001E-11	Coatomer subunit delta	Arcn1
Typical	-2.358070437	6.19579E-05	Resistin-like alpha	Retnla
Typical	-2.350787311	7.03032E-08	Lactotransferrin	Ltf
Typical	-2.350705931	8.7847E-06	Tetratricopeptide repeat protein 1	Ttc1
Typical	-2.334000248	3.45299E-07	SUMO-activating enzyme subunit 2	Uba2
Typical	-2.326504729	3.01636E-11	Phosphoglycerate kinase 1;Phosphoglycerate kinase	Pgk1
Typical	-2.324392594	0.000202351	C-C chemokine receptor type 1	Ccr1
Typical	-2.322833188	0.000632217	V-type proton ATPase subunit G 1	Atp6v1g1
Typical	-2.316529232	6.28434E-09	Malate dehydrogenase, cytoplasmic;Malate dehydrogenase	Mdh1
Typical	-2.311445978	9.74171E-10	Heat shock protein HSP 90-beta	Hsp90ab1
Typical	-2.308115451	1.68073E-12	Alpha-enolase;phosphopyruvate hydratase	Eno1
Typical	-2.306842295	5.95122E-06	Peptidyl-prolyl cis-trans isomerase FKBP3;peptidylprolyl isomerase	Fkbp3
Typical	-2.304993269	1.50538E-08	Ig gamma-2A chain C region, membrane-bound form;Ig gamma-2A chain C region, A allele	Igh-1a;Ighg
Typical	-2.303689236	9.56001E-14	V-type proton ATPase subunit B, brain isoform	Atp6v1b2
Typical	-2.291110675	4.7722E-10	von Willebrand factor A domain-containing protein 5A	Vwa5a
Typical	-2.288678296	3.24935E-07	P2R1A-PPP2R2A-interacting phosphatase regulator 1	Pabir1
Typical	-2.284740554	9.4672E-12	V-type proton ATPase catalytic subunit A	Atp6v1a
Typical	-2.280404705	1.39745E-10	Echinoderm microtubule-associated protein-like 2	Eml2
Typical	-2.280167664	0.000845875	Ankyrin repeat domain-containing protein 63	Ankrd63
Typical	-2.277233463	0.018344608	Cysteine protease ATG4A;Cysteine protease	Atg4a;Atg4a-ps
Typical	-2.276622539	8.10521E-09	Farnesyl pyrophosphate synthase	Fdps
Typical	-2.275606177	8.60935E-11	Integrin alpha-M	Gm49368;Itgam
Typical	-2.274425379	1.36006E-09	Polymeric immunoglobulin receptor;Secretory component	Pigr
Typical	-2.273206753	7.86429E-10	L-lactate dehydrogenase A chain;L-lactate dehydrogenase	Ldha
Typical	-2.270244005	1.01473E-06	Proliferating cell nuclear antigen	Pcna
Typical	-2.268308512	0.000100363	Alpha-1-antitrypsin 1-1;Alpha-1-antitrypsin 1-3	Serpina1a;Serpina1c
Typical	-2.264455774	1.08125E-09	Carbonic anhydrase 3	Ca3
Typical	-2.263661978	2.77473E-05	MYG1 exonuclease	Myg1
Typical	-2.257197931	8.23779E-07	Dual specificity protein phosphatase 3;Dual specificity protein phosphatase;protein-serine/threonine phosphatase	Dusp3
Typical	-2.255591287	1.00412E-08	Fructose-bisphosphate aldolase A;Fructose-bisphosphate aldolase	Aldoa;Aldoart1
Typical	-2.254980299	8.76909E-12	Valine--tRNA ligase;valine--tRNA ligase	Vars1
Typical	-2.253556739	1.37697E-07	Alcohol dehydrogenase 1	Adh1
Typical	-2.249867969	6.31036E-06	Partner of Y14 and mago	Pym1

Typical	-2.249439833	3.27125E-07	CTP synthase 1	Ctps1
Typical	-2.244116656	7.34888E-08	Hsc70-interacting protein	St13
Typical	-2.242319849	2.17495E-08	Alpha-1-antitrypsin 1-4	Serpina1d
Typical	-2.241563119	0.000260969	Xaa-Pro dipeptidase	Pepd
Typical	-2.240691461	6.96431E-06	Aldehyde oxidase 2	Aox2
Typical	-2.232981512	6.61775E-05	Glutamine--fructose-6-phosphate aminotransferase [isomerizing] 1	Gfpt1
Typical	-2.219603009	4.17048E-06	Sorting nexin-6;Sorting nexin-6, N-terminally processed	Snx6
Typical	-2.214576064	0.000290895	Ubiquitin-like FUBI-ribosomal protein eS30 fusion protein;Ubiquitin-like protein FUBI;Small ribosomal subunit protein eS30;40S ribosomal protein S30	Fau
Typical	-2.209956784	3.17503E-07	Macrophage metalloelastase	Mmp12
Typical	-2.20768723	1.61021E-08	Elongation factor 1-alpha 1	Eef1a1
Typical	-2.203139369	5.81235E-10	Sorting nexin-5	Snx5
Typical	-2.200216124	2.3243E-05	Transgelin	Tagln
Typical	-2.199795002	1.2034E-09	Serpin B6	Serpinb6a;Serpinb6
Typical	-2.195275667	6.36262E-08	Cofilin-1	Cfl1
Typical	-2.183781073	1.36884E-08	Protein RCC2	Rcc2
Typical	-2.183744791	1.06717E-09	Cytosol aminopeptidase	Lap3
Typical	-2.183433914	1.0639E-06	Mannose-1-phosphate guanyltransferase alpha	Gmppa
Typical	-2.182093175	0.032497591	Tubulin alpha-1B chain;Detyrosinated tubulin alpha-1B chain	Tuba1b
Typical	-2.181698354	8.36548E-05	Glia maturation factor gamma;Glia maturation factor	Gmfg
Typical	-2.177123663	1.24375E-07	Glutathione reductase, mitochondrial	Gsr
Typical	-2.176133516	4.20022E-07	Ubiquitin-conjugating enzyme E2 Z	Ube2z
Typical	-2.172547213	3.97121E-07	Four and a half LIM domains protein 1	Fhl1
Typical	-2.17239577	0.001202604	Uncharacterized protein C11orf97 homolog	1700012B09Rik
Typical	-2.171918403	0.000458179	Ig alpha chain C region	P01878
Typical	-2.17076878	4.29991E-06	Proteasome subunit beta type-10	Psmb10
Typical	-2.165867318	7.05752E-08	Acetyl-CoA acetyltransferase, cytosolic;acetyl-CoA C-acetyltransferase	Acat2;Acat3
Typical	-2.16567635	3.29111E-05	Rho GDP-dissociation inhibitor 1	Arhgdia
Typical	-2.16175872	1.72046E-08	Arfaptin-1	Arfip1;Gm37240
Typical	-2.150699849	2.49051E-07	Calpain-2 catalytic subunit	Capn2
Typical	-2.148056666	3.72085E-10	14-3-3 protein theta	Ywhaq
Typical	-2.146818754	7.88313E-14	Threonine--tRNA ligase 1, cytoplasmic	Tars1
Typical	-2.145248371	6.64402E-05	Ankyrin-3	Ank3
Typical	-2.142161857	1.90998E-11	Adenylyl cyclase-associated protein 1	Cap1
Typical	-2.136441061	2.53792E-07	Heat shock protein HSP 90-alpha	Hsp90aa1
Typical	-2.133636898	0.007623755	Anaphase-promoting complex subunit 7	Anapc7
Typical	-2.13315453	0.000137721	Apoptosis regulator BAX	Bax
Typical	-2.128007126	2.03298E-07	Bifunctional phosphoribosylaminoimidazole carboxylase/phosphoribosylaminoimidazole succinocarboxamide synthetase;Phosphoribosylaminoimidazole carboxylase;Phosphoribosylaminoimidazole succinocarboxamide synthetase	Paics
Typical	-2.124256473	8.95892E-07	Dihydropyrimidinase-related protein 2	Dpysl2
Typical	-2.124178929	0.003937797	Vertnin	Vrtn
Typical	-2.121161885	9.2823E-06	Calcyclin-binding protein	Cacybp
Typical	-2.120945252	2.30171E-08	Tryptophan--tRNA ligase, cytoplasmic;T1-TrpRS;T2-TrpRS	Wars1
Typical	-2.116773987	6.77894E-05	Proteasome activator complex subunit 1	Psme1
Typical	-2.111017842	3.43236E-10	Sialic acid synthase	Nans
Typical	-2.110630099	4.30513E-06	Sorting nexin-3	Snx3
Typical	-2.110528331	1.30099E-06	Dihydropyrimidinase-related protein 3	Dpysl3
Typical	-2.105228424	1.72789E-05	Aldehyde oxidase 3	Aox3
Typical	-2.099225659	1.89332E-07	Proteasome subunit beta type-9	Psmb9
Typical	-2.095549901	0.001983289	Protein Largen	Prr16
Typical	-2.090592469	3.00798E-08	Large ribosomal subunit protein eL42	Rpl36a;Gm6525
Typical	-2.083170573	1.31562E-05	DAZ-associated protein 1	Dazap1
Typical	-2.080911085	2.73847E-07	Actin-like 11	Actl11
Typical	-2.080070665	4.35561E-06	14-3-3 protein gamma;14-3-3 protein gamma, N-terminally processed	Ywhag
Typical	-2.079718632	2.39933E-09	Aldo-keto reductase family 1, member B10	Akr1b10
Typical	-2.078702778	3.57847E-05	N-acylglucosamine 2-epimerase	Renbp
Typical	-2.067977333	4.13772E-08	Glycogen phosphorylase, brain form	Pygb

Typical	-2.06234879	3.38298E-05	F-box only protein 50	Nccrp1
Typical	-2.058400917	7.89917E-05	Spermidine synthase	Srm
Typical	-2.05644652	0.000231735	V-type proton ATPase subunit F	Atp6v1f
Typical	-2.054919603	1.23745E-06	NEDD8-conjugating enzyme Ubc12;E2 ubiquitin-conjugating enzyme	Ube2m
Typical	-2.054791408	7.07429E-05	Protein S100-A4	S100a4
Typical	-2.054734124	0.001291033	Immunoglobulin kappa chain variable 6-17	Igkv6-17
Typical	-2.054506365	0.00012891	Small ubiquitin-related modifier 2;Small ubiquitin-related modifier 3;Small ubiquitin-related modifier	Sumo2;Sumo3;Gm49325
Typical	-2.053549788	6.70211E-08	Programmed cell death 1 ligand 2	Pdcd1lg2
Typical	-2.051240095	0.000124188	Alpha-1-antitrypsin 1-5	Serpina1e
Typical	-2.051146995	4.17061E-09	Transcription elongation factor A protein 1;Transcription elongation factor	Tcea1
Typical	-2.046089639	1.36694E-10	Protein Niban 2	Niban2
Typical	-2.044187249	3.43636E-05	glutathione transferase	Gm20441
Typical	-2.043519105	0.027329087	Glutathione S-transferase theta-3;glutathione transferase	Gstt3
Typical	-2.043191422	1.25851E-08	14-3-3 protein epsilon	Ywhae
Typical	-2.042212613	0.000139835	Procathepsin L;Cathepsin L;Cathepsin L heavy chain;Cathepsin L light chain	Ctsl
Typical	-2.041945606	2.88246E-07	Peptidyl-prolyl cis-trans isomerase FKBP4;Peptidyl-prolyl cis-trans isomerase FKBP4, N-terminally processed;peptidylprolyl isomerase	Fkbp4
Typical	-2.041544321	0.004351141	Uteroglobin	Scgb1a1
Typical	-2.041086303	7.23658E-06	Cytokine receptor-like factor 3	Crlf3
Typical	-2.031123119	3.47534E-05	SH3 domain-binding glutamic acid-rich-like protein 3	Sh3bgrl3
Typical	-2.030902502	7.77096E-06	High mobility group protein B3	Hmgb3
Typical	-2.030449592	3.0601E-06	Calcium/calmodulin-dependent protein kinase type 1D	Camk1d
Typical	-2.029498461	8.39346E-06	Immunoglobulin heavy variable 9	Ighv9-4;Ighv9-1;Ighv9-3;Ighv9-2
Typical	-2.02924082	1.25227E-05	Adenine phosphoribosyltransferase	Aprt
Typical	-2.026199341	2.21052E-09	Signal transducer and activator of transcription;Signal transducer and activator of transcription 1	Stat1
Typical	-2.023377419	2.23079E-08	Glycerol-3-phosphate dehydrogenase 1-like protein	Gpd1l
Typical	-2.023295763	1.48819E-09	Transgelin-2	Tagln2
Typical	-2.019968457	9.10763E-08	Immunoglobulin heavy variable 14	Ighv14-3;Ighv14-1;Ighv14-4;Ighv14-2
Typical	-2.019194158	3.93962E-07	Alcohol dehydrogenase class-3	Adh5
Typical	-2.017422507	1.34485E-06	Apoptosis-associated speck-like protein containing a CARD	Pycard
Typical	-2.013991653	0.000220203	Cell division cycle-associated protein 2	Cdca2
Typical	-2.012339189	0.000674919	Translin-associated protein X	Tsnax
Typical	-2.012318696	4.20903E-06	Mannose-1-phosphate guanyltransferase beta	Gmppb
Typical	-2.010898823	1.50101E-05	Ribosome maturation protein SBDS	Sbds
Typical	-2.008267212	1.15784E-08	WD repeat-containing protein 1	Wdr1
Typical	-2.006731711	0.000179681	Tubulin-specific chaperone A	Tbca
Typical	-2.001947191	9.31058E-07	Proteasome subunit beta type-8;Proteasome subunit beta	Psmb8
Typical	-1.998547257	5.28672E-10	Coatomer subunit beta	Copb1
Typical	-1.995375167	3.75782E-05	p53-induced death domain-containing protein 1;PIDD-N;PIDD-C;PIDD-CC	Pidd1
Typical	-1.991016028	8.9347E-08	Protein diaphanous homolog 1	Diaph1
Typical	-1.990920088	7.70014E-06	Pantothenate kinase 3	Pank3
Typical	-1.98930876	0.000580717	BLOC-2 complex member HPS3;Hermansky-Pudlak syndrome 3 protein homolog	Hps3
Typical	-1.98861245	5.70157E-09	Twinfilin-2	Twf2
Typical	-1.988400078	0.00011347	Glutathione S-transferase theta-1	Gstt1
Typical	-1.987489001	2.68651E-06	Myeloperoxidase;Myeloperoxidase light chain;Myeloperoxidase heavy chain	Mpo
Typical	-1.986134487	3.6903E-07	Rho GTPase-activating protein 1	Arhgap1
Typical	-1.984899245	1.31531E-08	Transcription factor p65	Rela
Typical	-1.982277552	3.25936E-09	14-3-3 protein zeta/delta	Ywhaz
Typical	-1.980959574	2.4734E-08	Actin-related protein 3	Actr3
Typical	-1.980292214	1.50684E-07	V-type proton ATPase subunit E 1	Atp6v1e1
Typical	-1.975211461	2.27998E-05	Glutathione S-transferase A4	Gsta4
Typical	-1.973899778	0.000817626	Proteoglycan 3	Prg3
Typical	-1.972500144	6.98139E-09	Protein transport protein Sec31A	Sec31a
Typical	-1.970268483	5.24229E-08	Glycerol-3-phosphate dehydrogenase [NAD(+)], cytoplasmic;Glycerol-3-phosphate dehydrogenase [NAD(+)]	Gpd1

Typical	-1.968862385	4.91426E-05	Neutrophilic granule protein	Ngp
Typical	-1.968753666	0.001514724	Copper transport protein ATOX1	Atox1
Typical	-1.966788758	0.002024502	E3 ubiquitin-protein ligase MGRN1	Mgrn1
Typical	-1.963797739	2.28842E-08	Elongation factor 1-delta	Eef1d
Typical	-1.960221778	3.36739E-09	Complement C3;Complement C3 beta chain;C3-beta-c;Complement C3 alpha chain;C3a anaphylatoxin;Acylation stimulating protein;Complement C3b alpha chain;Complement C3c alpha chain fragment 1;Complement C3dg fragment;Complement C3g fragment;Complement C3d fragment;Complement C3f fragment;Complement C3c alpha chain fragment 2	C3
Typical	-1.957228724	2.65126E-08	Inositol-3-phosphate synthase 1	Isyna1
Typical	-1.956828287	0.001464404	NEDD8	Nedd8
Typical	-1.953809717	9.36255E-10	V-type proton ATPase subunit C 1	Atp6v1c1
Typical	-1.950100623	2.68427E-08	Small glutamine-rich tetratricopeptide repeat-containing protein alpha	Sgta
Typical	-1.950053067	7.27784E-06	Immunoglobulin kappa constant	Igkc
Typical	-1.949039459	0.00042999	Neutrophil cytosol factor 4	Ncf4
Typical	-1.947871335	5.66518E-08	Drebrin-like protein	Dbnl
Typical	-1.943940756	3.92138E-10	Integrin beta-2;Integrin beta	Itgb2
Typical	-1.941643037	1.44392E-05	Cathepsin B;Cathepsin B light chain;Cathepsin B heavy chain	Ctsb
Typical	-1.940510941	3.73805E-05	COMM domain-containing protein 3	Commd3
Typical	-1.938315879	3.60126E-06	Guanylate-binding protein 1	Gbp1;Gbp2b
Typical	-1.934690645	1.10594E-05	Immunoglobulin kappa variable 8	Igkv8-21;Igkv8-27;Igkv8-30
Typical	-1.93449671	0.003215278	Protein S100;Protein S100-A1	S100a1
Typical	-1.933856053	0.000445874	Tubulin beta-6 chain	Tubb6
Typical	-1.928898345	0.001597875	GTP-binding protein SAR1b	Sar1b
Typical	-1.928594738	0.006006708	glycogenin glucosyltransferase;Glycogenin-1	Gyg1
Typical	-1.925774829	0.003378056	Nuclear migration protein nudC	Nudc
Typical	-1.924339549	1.68257E-08	14-3-3 protein beta/alpha;14-3-3 protein beta/alpha, N-terminally processed;14-3-3 protein beta/alpha	Ywhab
Typical	-1.922509914	1.1676E-05	S-adenosylmethionine synthase isoform type-2;S-adenosylmethionine synthase	Mat2a
Typical	-1.919061534	0.003188821	GTPase Era, mitochondrial	Eral1
Typical	-1.916556104	4.29778E-07	LIM and SH3 domain protein 1	Lasp1
Typical	-1.915930261	1.0485E-08	Synaptobrevin homolog YKT6	Ykt6
Typical	-1.912101788	3.44849E-05	Dual specificity mitogen-activated protein kinase kinase 1	Map2k1
Typical	-1.910394054	0.000150136	Acyl-CoA-binding protein	Dbi
Typical	-1.909619204	4.90828E-06	Homeodomain-only protein	Hopx
Typical	-1.907193036	6.84525E-05	Phenylalanine--tRNA ligase beta subunit	Farsb
Typical	-1.903146574	0.000136313	Sorting nexin-12	Snx12
Typical	-1.899282731	6.29082E-08	Coatomer subunit beta	Copb2
Typical	-1.898508517	0.003863869	Immunoglobulin heavy constant gamma 2B	Ighg2b;Ighg2c
Typical	-1.898467233	1.35181E-05	Peptidyl-prolyl cis-trans isomerase A;Peptidyl-prolyl cis-trans isomerase A, N-terminally processed;Peptidyl-prolyl cis-trans isomerase	Ppia
Typical	-1.896529388	2.55769E-05	Immunoglobulin kappa variable 4	Igkv4-57-1
Typical	-1.895992639	2.69156E-08	Actin-related protein 2/3 complex subunit 3	Arpc3
Typical	-1.891633267	0.000185565	Acyl-protein thioesterase 2	Lypla2
Typical	-1.891212357	1.6288E-06	Glyceraldehyde-3-phosphate dehydrogenase	Gapdh;Gapdhrt;Gapdhrt2
Typical	-1.890712992	3.24496E-06	Destrin	Dstn
Typical	-1.889793841	4.46987E-07	Histidine--tRNA ligase, cytoplasmic	Hars1
Typical	-1.88910514	1.0324E-08	Poly(rC)-binding protein 2	Pcbp2
Typical	-1.888023631	0.000744798	Putative hydroxypyruvate isomerase	Hyi
Typical	-1.883571773	1.45444E-05	Tubulin-folding cofactor B	Tbcb
Typical	-1.880958854	9.49711E-07	Y-box-binding protein 1	Ybx1
Typical	-1.880326292	3.81447E-05	Caspase-9;Caspase-9 subunit p35;Caspase-9 subunit p10	Casp9
Typical	-1.879905785	1.07108E-07	ATP-dependent 6-phosphofructokinase, platelet type;ATP-dependent 6-phosphofructokinase	Pfkp
Typical	-1.879134051	2.62435E-07	Neutrophil cytosol factor 2	Ncf2
Typical	-1.877857696	4.6478E-05	Nuclear ubiquitous casein and cyclin-dependent kinase substrate 1	Nucks1
Typical	-1.876507632	8.05569E-08	Hematopoietic lineage cell-specific protein	Hcls1
Typical	-1.875796254	2.93334E-05	Transmembrane glycoprotein NMB	Gpnmb

Typical	-1.870552847	0.003168554	Potassium voltage-gated channel subfamily B member 2	Kcnb2
Typical	-1.869954957	5.66715E-08	Xaa-Pro aminopeptidase 1	Xpnpep1
Typical	-1.866116079	7.97805E-07	Dipeptidyl peptidase 3	Dpp3
Typical	-1.861727291	2.44657E-06	Golgi reassembly-stacking protein 2	Gorasp2
Typical	-1.861176258	4.93661E-06	Prosaposin;Prosaposin;Saposin-A;Saposin-B-Val;Saposin-B;Saposin-C;Saposin-D	Psap
Typical	-1.858284781	1.69161E-08	Ig kappa chain V-VI region NQ2-6.1	Igkv4-55
Typical	-1.856727134	1.04E-08	Glucose-6-phosphate 1-dehydrogenase X;Glucose-6-phosphate 1-dehydrogenase	G6pdx
Typical	-1.852966075	0.000145462	Selenide, water dikinase 1	Sephs1
Typical	-1.851148563	3.98828E-05	Prostaglandin reductase 1	Ptgr1
Typical	-1.843547863	0.000541634	MOB kinase activator 2	Mob2
Typical	-1.838724857	6.00066E-08	Gasdermin-D;Gasdermin-D, N-terminal;Gasdermin-D, C-terminal;Gasdermin-D, p13;Gasdermin-D, p40	Gsdmd
Typical	-1.83656434	3.69718E-06	AP-3 complex subunit mu-1	Ap3m1
Typical	-1.833467547	5.94686E-08	UMP-CMP kinase	Cmpk1
Typical	-1.832308261	7.3018E-05	Neutrophil gelatinase-associated lipocalin	Lcn2
Typical	-1.830778652	1.98962E-05	Ig gamma-3 chain C region	Ighg3
Typical	-1.828427039	9.8552E-06	Actin-related protein 2/3 complex subunit 1B;Actin-related protein 2/3 complex subunit	Arpc1b
Typical	-1.828074116	0.000275723	Testin	Tes
Typical	-1.828007147	0.000281824	Pyridoxal kinase	Pdxk
Typical	-1.825101789	4.55539E-06	Glycine--tRNA ligase	Gars1
Typical	-1.824152671	2.76619E-06	Far upstream element-binding protein 1	Fubp1
Typical	-1.822313012	1.95269E-06	Aspartyl aminopeptidase	Dnpep
Typical	-1.821178203	7.36951E-07	Aldehyde dehydrogenase family 16 member A1	Aldh16a1
Typical	-1.820150714	1.86899E-07	Coatomer subunit alpha;Xenin;Proxenin;Coatomer subunit alpha	Copa
Typical	-1.819852426	1.86083E-06	Stathmin	Stmn1
Typical	-1.818455505	0.004949102	Transient receptor potential cation channel subfamily V member 4	Trpv4
Typical	-1.818167856	0.006765187	Thrombospondin-type laminin G domain and EAR repeat-containing protein	Tspear
Typical	-1.817299016	0.000425821	Glia maturation factor beta	Gmfb
Typical	-1.813595496	5.76091E-07	3(2),5-bisphosphate nucleotidase 1	Bpnt1
Typical	-1.812857967	3.7278E-07	eIF5-mimic protein 2	Bzw1
Typical	-1.811757957	0.000739947	Coatomer subunit zeta;Coatomer subunit zeta-1	Copz1
Typical	-1.81082681	2.01891E-05	DNA repair nuclease/redox regulator APEX1;DNA repair nuclease/redox regulator APEX1, mitochondrial;DNA-(apurinic or apyrimidinic site) endonuclease	Apex1
Typical	-1.8079756	2.88113E-08	Argininosuccinate lyase	Asl
Typical	-1.807317395	1.56623E-05	Elongation factor 1-gamma	Eef1g
Typical	-1.80580207	4.55222E-07	Chloride intracellular channel protein 3	Clic3
Typical	-1.803124237	1.429E-07	EF-hand domain-containing protein D2	Efhd2
Typical	-1.800488853	1.77339E-06	Far upstream element-binding protein 2	Khsrp
Typical	-1.79926423	1.5679E-07	Proteasome activator complex subunit 2	Psme2
Typical	-1.794551341	7.59546E-07	Prostaglandin reductase 2	Ptgr2
Typical	-1.793681505	1.69941E-05	GTPase IMAP family member 4	Gimap4
Typical	-1.790601582	5.7561E-05	Legumain	Lgmn
Typical	-1.787663523	8.38019E-08	Ribosyldihydronicotinamide dehydrogenase [quinone]	Nqo2
Typical	-1.783931923	4.13821E-05	Lysosome-associated membrane glycoprotein 3	Lamp3
Typical	-1.777594058	7.53812E-07	Ras GTPase-activating-like protein IQGAP1	Iqgap1
Typical	-1.776769723	3.52687E-08	Sorting nexin-1	Snx1
Typical	-1.776144663	6.62444E-07	Periodic tryptophan protein 1 homolog	Pwp1
Typical	-1.775951343	0.000186916	Fatty acid-binding protein, heart	Fabp3
Typical	-1.774399334	3.24442E-09	Puromycin-sensitive aminopeptidase;Aminopeptidase	Npepps
Typical	-1.770576604	3.37726E-05	Small ribosomal subunit protein eS26	Rps26
Typical	-1.768272209	0.000278877	Ig heavy chain V region AC38 205.12;Ig heavy chain V region MOPC 104E;Ig heavy chain V region J558	Ighv1-31;Ighv1-42;Ighv1-26;Ighv1-34
Typical	-1.768113984	1.63423E-06	Rab GDP dissociation inhibitor beta;Rab GDP dissociation inhibitor	Gdi2
Typical	-1.767940436	5.35139E-06	Coatomer subunit epsilon	Cope
Typical	-1.764796172	3.13378E-06	Rab GDP dissociation inhibitor alpha	Gdi1
Typical	-1.761253081	1.86995E-06	Phosphatidylinositol transfer protein alpha isoform	Pitpna

Typical	-1.759037717	1.78672E-05	Na(+)/H(+) exchange regulatory cofactor NHE-RF1	Nherf1
Typical	-1.757409371	0.000330281	Ribonuclease H2 subunit A;Ribonuclease	Rnaseh2a
Typical	-1.756575245	2.39671E-08	Alanine--glyoxylate aminotransferase	Agxt
Typical	-1.75538169	3.41176E-06	Adenosine kinase	Adk
Typical	-1.755271615	3.10256E-05	CYFIP-related Rac1 interactor B	Cyrib
Typical	-1.752721829	0.000851609	Phosphoacetylglucosamine mutase	Pgm3
Typical	-1.748493173	2.24327E-06	Haptoglobin;Haptoglobin alpha chain;Haptoglobin beta chain	Hp
Typical	-1.746674262	2.17576E-09	Elongation factor 1-beta	Eef1b;Eef1b2
Typical	-1.746556875	5.93389E-07	Actin-related protein 2/3 complex subunit 2;Arp2/3 complex 34 kDa subunit	Arpc2
Typical	-1.744178878	6.15822E-07	V-type proton ATPase subunit H	Atp6v1h
Typical	-1.744128948	4.39163E-09	Tyrosine-protein kinase SYK;Tyrosine-protein kinase	Syk
Typical	-1.741872406	6.06042E-09	Alanine--tRNA ligase, cytoplasmic	Aars1
Typical	-1.741821649	0.001228962	Fanconi anemia group J protein homolog	Brip1
Typical	-1.741113027	0.00072337	Inositol polyphosphate 1-phosphatase	Inpp1
Typical	-1.739669334	9.49083E-05	Glucosamine-6-phosphate isomerase 1	Gnpda1
Typical	-1.738661067	1.81816E-05	Tubulin polymerization-promoting protein family member 3	Tppp3
Typical	-1.737415335	2.64877E-07	Serine/threonine-protein kinase PAK 2;PAK-2p27;PAK-2p34	Pak2
Typical	-1.736895985	5.90539E-08	Peroxiredoxin-1	Prdx1
Typical	-1.733347278	0.000289322	Prostaglandin E synthase 3	Ptges3
Typical	-1.731789038	2.46825E-05	Ubiquitin-like-conjugating enzyme ATG3	Atg3
Typical	-1.730184089	3.20442E-06	KH domain-containing RNA-binding protein QKI	Qki
Typical	-1.729051548	1.35895E-05	Signal transducer and activator of transcription 3;Signal transducer and activator of transcription	Stat3
Typical	-1.727781635	1.74401E-05	Methanethiol oxidase;Selenium-binding protein 2	Selenbp1;Selenbp2
Typical	-1.7255707	1.76178E-06	Anamorsin	Ciapin1
Typical	-1.724576547	1.85878E-05	Cytoplasmic dynein 1 light intermediate chain 1	Dync1li1
Typical	-1.724195056	0.001333728	Glutamate-rich 6B	Erich6b
Typical	-1.723393228	5.7952E-05	Tubulin beta-2A chain;Tubulin beta-2B chain	Tubb2a;Tubb2b
Typical	-1.722785801	8.12649E-05	Eukaryotic translation initiation factor 1A, X-chromosomal;Eukaryotic translation initiation factor 1A;Eukaryotic translation initiation factor 4C	Eif1ax;Eif1a;Eif1ad15
Typical	-1.719636981	1.18457E-05	tRNA (32-2-O)-methyltransferase regulator THADA	Thada
Typical	-1.717168617	5.26743E-05	Aminopeptidase B	Rnpep
Typical	-1.716015795	4.12625E-07	Sorting nexin-2	Snx2
Typical	-1.71415721	9.27985E-05	C-X-C motif chemokine 15	Cxcl15
Typical	-1.711356841	4.93523E-07	Ubiquitin-like modifier-activating enzyme 1	Uba1
Typical	-1.711325603	0.00010496	Large ribosomal subunit protein uL14m	Mrpl14
Typical	-1.710578579	0.000294766	Fructose-1,6-bisphosphatase 1	Fbp1
Typical	-1.709248013	0.00018181	AP-3 complex subunit sigma-1	Ap3s1
Typical	-1.708010991	0.001017626	Oviduct-specific glycoprotein	Ovgp1
Typical	-1.706104279	4.60741E-08	Large ribosomal subunit protein eL31	Rpl31
Typical	-1.7058815	0.001257301	Pulmonary surfactant-associated protein B	Sftpb
Typical	-1.705602031	1.70735E-07	Complement factor H	Cfh
Typical	-1.70139247	1.24609E-08	Retinoid-inducible serine carboxypeptidase	Scpep1
Typical	-1.700806745	2.78188E-06	Trk-fused protein	Tfg
Typical	-1.699505509	8.09143E-07	Unconventional myosin-If	Myo1f
Typical	-1.698567157	0.000419803	RNA 3-terminal phosphate cyclase	RtcA;Rtca
Typical	-1.695956866	6.95038E-07	Bridging integrator 2	Bin2
Typical	-1.695395364	6.03435E-05	Inhibitor of carbonic anhydrase	Inhca
Typical	-1.693560516	3.96499E-06	Queuosine 5-phosphate N-glycosylase/hydrolase	QNG1
Typical	-1.693541103	0.000258456	Tubulin beta-5 chain	Tubb5
Typical	-1.691505962	4.12528E-06	14-3-3 protein sigma	Sfn
Typical	-1.690850258	0.000416901	Immunoglobulin kappa variable 1	Igkv1-135;Igkv1-133
Typical	-1.689861743	1.7624E-05	Actin-related protein 2/3 complex subunit 4	Arpc4
Typical	-1.688156594	3.21456E-05	Pleckstrin homology domain-containing family O member 2	Plekho2
Typical	-1.686166488	1.41105E-05	Low affinity immunoglobulin gamma Fc region receptor II	Fcgr2;Fcgr2b
Typical	-1.684516886	3.01949E-06	Eukaryotic initiation factor 4A-I	Eif4a1
Typical	-1.683797582	8.54829E-07	Serine--tRNA ligase, cytoplasmic;serine--tRNA ligase	Sars1
Typical	-1.680787214	4.29729E-05	Signal recognition particle subunit SRP68	Srp68
Typical	-1.680538474	0.000412686	Stromal membrane-associated protein 2	Smap2

Typical	-1.678344557	2.17556E-07	Coatomer subunit gamma-2	Copg2
Typical	-1.675378503	7.82473E-06	Pulmonary surfactant-associated protein C	Sftpc
Typical	-1.674506611	1.83166E-05	Cyclin-dependent kinase 2	Cdk2
Typical	-1.67398031	5.68461E-05	Thioredoxin	Txn
Typical	-1.673109245	2.9369E-05	6-phosphogluconolactonase	Pgls
Typical	-1.671127595	2.01877E-06	6-pyruvoyl tetrahydrobiopterin synthase	Pts
Typical	-1.665160349	0.000561766	ADP-ribosylation factor-binding protein GGA1	Gga1
Typical	-1.661695671	0.000100855	Poly(rC)-binding protein 1	Pcbp1
Typical	-1.657637448	0.000161015	Inositol monophosphatase 2	Impa2
Typical	-1.65599361	2.46116E-05	Large ribosomal subunit protein uL14	Rpl23
Typical	-1.655365245	6.29379E-07	Small ribosomal subunit protein eS6	Rps6
Typical	-1.654251777	8.59966E-05	Serine/threonine-protein kinase N1	Pkn1
Typical	-1.652939076	0.000169976	Large ribosomal subunit protein bL28m	Mrpl28
Typical	-1.647199843	0.001531476	Large ribosomal subunit protein uL29	Rpl35
Typical	-1.644496621	0.001559959	Anterior gradient protein 2 homolog	Agr2
Typical	-1.644070138	0.000228719	Prefoldin subunit 6	Pfdn6
Typical	-1.64375	4.65515E-05	Serine protease inhibitor A3M	Serpina3m
Typical	-1.642359373	1.09737E-05	Translationally-controlled tumor protein	Tpt1
Typical	-1.642238638	7.04794E-06	Platelet-activating factor acetylhydrolase	Pla2g7
Typical	-1.641941494	0.000446266	Platelet-activating factor acetylhydrolase IB subunit alpha1	Pafah1b3
Typical	-1.641233063	5.856E-09	Exportin-2	Cse1l
Typical	-1.639498711	6.16446E-07	Glycylpeptide N-tetradecanoyltransferase 1	Nmt1
Typical	-1.632658407	4.20402E-06	Ubiquitin-conjugating enzyme E2 N;E2 ubiquitin-conjugating enzyme	Ube2n
Typical	-1.630428081	7.66014E-07	Calponin-2;Calponin	Cnn2
Typical	-1.629768032	5.92975E-05	Replication protein A 32 kDa subunit	Rpa2
Typical	-1.629363569	0.00011731	Vacuolar protein sorting-associated protein 29	Vps29
Typical	-1.627127414	0.000419232	1-acylglycerol-3-phosphate O-acyltransferase ABHD5	Abhd5
Typical	-1.626173676	3.88437E-05	Golgi to ER traffic protein 4 homolog	Get4
Typical	-1.626116053	0.00015239	ADP-ribosylation factor 5	Arf5
Typical	-1.626112302	8.56476E-08	Isoleucine--tRNA ligase, cytoplasmic	Iars1
Typical	-1.625976457	0.001528194	BPI fold-containing family A member 1	Bpifa1
Typical	-1.624548743	1.74602E-05	14-3-3 protein eta	Ywhah
Typical	-1.623704995	3.48551E-05	S-methyl-5-thioadenosine phosphorylase	Mtap
Typical	-1.620253944	2.31405E-06	Small ribosomal subunit protein uS17	Rps11
Typical	-1.619908206	1.13827E-07	Coronin-7	Coro7
Typical	-1.618067975	0.000625651	Ig kappa chain V-II region 7S34.1	Igkv2-137
Typical	-1.616934734	2.98949E-06	Formin-binding protein 1	Fnbp1
Typical	-1.616182857	6.09515E-05	AH receptor-interacting protein	Aip
Typical	-1.614518929	0.00038718	N-acetylgalactosamine kinase	Galk2
Typical	-1.613818487	9.22017E-06	Large ribosomal subunit protein uL16;Large ribosomal subunit protein uL16-like	Rpl10;Rpl10l
Typical	-1.613462024	5.56028E-07	Beta-1,4-galactosyltransferase 1;Processed beta-1,4-galactosyltransferase 1;Beta-1,4-galactosyltransferase	B4galt1
Typical	-1.611370193	6.73322E-06	Ubiquitin-conjugating enzyme E2 H;E2 ubiquitin-conjugating enzyme	Ube2h
Typical	-1.610552428	1.64216E-07	Mitogen-activated protein kinase 3;Mitogen-activated protein kinase;mitogen-activated protein kinase	Mapk3
Typical	-1.606591119	0.00017051	Myeloblastin	Prtn3
Typical	-1.605944676	4.80107E-08	Inter alpha-trypsin inhibitor, heavy chain 4	Itih4
Typical	-1.605546591	0.000118264	14 kDa phosphohistidine phosphatase	Phpt1
Typical	-1.605427297	1.69711E-06	Glutamate--cysteine ligase catalytic subunit	Gclc
Typical	-1.603457387	2.92423E-07	Annexin A1;Annexin Ac2-26;Annexin	Anxa1
Typical	-1.602104908	6.4771E-05	Voltage-gated potassium channel subunit beta-1;Voltage-gated potassium channel subunit beta-2	Kcnab2
Typical	-1.595759265	6.00125E-05	Density-regulated protein	Denr
Typical	-1.594955826	3.59701E-09	Programmed cell death 6-interacting protein	Pdcd6ip
Typical	-1.592917527	8.88108E-08	SUMO-conjugating enzyme UBC9	Ube2i
Typical	-1.592517069	1.26642E-08	Switch-associated protein 70	Swap70
Typical	-1.589215151	0.000444571	Developmentally-regulated GTP-binding protein 1	Drg1
Typical	-1.580340725	3.06744E-06	Pro-cathepsin H;Cathepsin H mini chain;Cathepsin H;Cathepsin H heavy chain;Cathepsin H light chain;Pro-cathepsin H	Ctsh
Typical	-1.578755485	4.06538E-10	Sec24-related gene family, member C	Sec24c

Typical	-1.578436957	5.01959E-07	Large ribosomal subunit protein uL10	Rplp0
Typical	-1.577055719	9.3854E-05	Protein phosphatase 1G	Ppm1g
Typical	-1.57589283	0.000132019	Asparagine--tRNA ligase, cytoplasmic;asparagine--tRNA ligase	Nars1
Typical	-1.574882825	1.01833E-05	Vasodilator-stimulated phosphoprotein	Vasp
Typical	-1.574088923	0.000148104	Small ribosomal subunit protein uS12	Rps23
Typical	-1.573420016	8.29333E-09	Eukaryotic peptide chain release factor subunit 1	Etf1
Typical	-1.571809938	0.000123403	Tumor necrosis factor alpha-induced protein 8-like protein 2	Tnfaip8l2
Typical	-1.568894047	0.000144458	Large ribosomal subunit protein uL23	Rpl23a
Typical	-1.567726983	1.17004E-05	Sorting nexin-29	Snx29
Typical	-1.566699791	0.000370238	ADP-ribosylation factor 1;ADP-ribosylation factor 3;ADP-ribosylation factor 2	Arf1;Arf3;Arf2
Typical	-1.566003927	6.52513E-05	Calcium-binding protein 39	Cab39
Typical	-1.564253998	0.000195161	N-acetyl-D-glucosamine kinase	Nagk
Typical	-1.564036751	7.27963E-08	Microtubule-associated protein 4	Map4
Typical	-1.563387087	2.53237E-05	Actin-related protein 2	Actr2
Typical	-1.563258298	1.63546E-05	Ras-related GTP-binding protein C;Ras-related GTP-binding protein D;Ras-related GTP-binding protein	Rragc;Rragd
Typical	-1.560491795	7.61623E-10	Glyoxalase domain-containing protein 4	Glod4
Typical	-1.55933609	0.000515542	Small ribosomal subunit protein uS11	Rps14
Typical	-1.558262719	0.000628905	Double C2-like domain-containing protein gamma;Double C2-like domain-containing protein	Doc2g
Typical	-1.557190874	4.0275E-06	Nucleoside diphosphate kinase A;Nucleoside diphosphate kinase	Nme1
Typical	-1.557189115	0.000134369	Large ribosomal subunit protein eL24	Rpl24
Typical	-1.556518661	0.000288074	Proteasomal ubiquitin receptor ADRM1	Adrm1;Adrm1b
Typical	-1.556512176	0.000114075	Plasminogen activator inhibitor 2, macrophage	Serpinb2
Typical	-1.554327668	1.86613E-08	Dedicator of cytokinesis protein 2	Dock2
Typical	-1.553128094	3.35482E-05	Lysosomal alpha-mannosidase	Man2b1
Typical	-1.546589873	3.28079E-08	Polyadenylate-binding protein 1	Pabpc1
Typical	-1.546231291	1.24819E-06	Amyloid beta A4 precursor protein-binding family B member 1-interacting protein	Apbb1ip
Typical	-1.545960108	3.72894E-05	Selenocysteine lyase	Scly
Typical	-1.541901292	3.24295E-09	Bifunctional glutamate/proline--tRNA ligase;Glutamate--tRNA ligase;Proline--tRNA ligase	Eprs1
Typical	-1.541250081	0.000279785	Reticulocalbin-3	Rcn3
Typical	-1.537390561	4.1802E-05	Galactokinase	Galk1
Typical	-1.536242718	1.11886E-05	Calponin-3;Calponin	Cnn3
Typical	-1.534526719	0.000389517	Cytochrome b-245 light chain	Cyba
Typical	-1.533562872	0.000271984	GMP synthase [glutamine-hydrolyzing]	Gmps
Typical	-1.53164285	2.10583E-07	Murinoglobulin-1	Mug1
Typical	-1.530475574	5.26872E-05	Large ribosomal subunit protein uL22	Rpl17
Typical	-1.530370882	4.81699E-06	Arylsulfatase B	Arsb
Typical	-1.52970615	5.20408E-08	Eukaryotic translation initiation factor 2A;Eukaryotic translation initiation factor 2A, N-terminally processed	Eif2a
Typical	-1.529000537	1.54879E-09	Leucine-rich repeat-containing protein 47	Lrrc47
Typical	-1.527653058	6.62443E-05	BRISC and BRCA1-A complex member 2	Babam2
Typical	-1.52719805	0.000136994	Bromodomain testis-specific protein	Brdt
Typical	-1.523978933	8.37557E-06	Myc box-dependent-interacting protein 1	Bin1
Typical	-1.523936759	5.51176E-05	Ubiquitin-conjugating enzyme E2 variant 1	Gm20431;Ube2v1
Typical	-1.522605112	2.30801E-05	Immunoglobulin heavy constant mu;Mu chain	Ighm
Typical	-1.521476025	6.79293E-06	Carbonyl reductase [NADPH] 1	Cbr1
Typical	-1.52012469	4.55542E-09	Beta-glucuronidase	Gusb
Typical	-1.519436306	3.04301E-06	Dihydropteridine reductase	Qdpr
Typical	-1.518434037	4.7679E-05	Microtubule-associated protein RP/EB family member 1	Mapre1
Typical	-1.515805372	0.000249794	Citrate synthase	Csl
Typical	-1.515785217	8.05665E-05	Cytochrome b-245 heavy chain	Cybb
Typical	-1.514503945	0.000247088	Ubiquitin-conjugating enzyme E2 D2;Ubiquitin-conjugating enzyme E2 D3;E2 ubiquitin-conjugating enzyme	Ube2d2;Ube2d3;Ube2d2a
Typical	-1.510320091	5.37375E-05	Large ribosomal subunit protein eL14	Rpl14
Typical	-1.508941396	6.06503E-05	Ubiquitin-fold modifier 1	Ufm1
Typical	-1.507100529	6.66913E-05	Proteasome subunit beta type-4	Psmb4

Typical	-1.505500687	3.03854E-08	Rabankyrin-5	Ankfy1
Typical	-1.505250253	3.46282E-05	Galectin-3;Galectin	Lgals3
Typical	-1.504656707	7.29467E-05	Muscleblind-like protein 1;Muscleblind-like protein 2	Mbnl1;Mbnl2
Typical	-1.504157427	0.000146248	Chloride intracellular channel protein 4	Clic4
Typical	-1.500283347	3.63222E-06	5-3 exonuclease PLD3	Pld3
Typical	-1.499466345	2.68526E-05	Large ribosomal subunit protein uL24	Rpl26
Typical	-1.497274039	7.40823E-06	Rho guanine nucleotide exchange factor 6	Arhgef6
Typical	-1.497240512	2.75782E-05	Protein mono-ADP-ribosyltransferase PARP3;Poly [ADP-ribose] polymerase	Parp3
Typical	-1.493308597	1.11512E-06	DnaJ homolog subfamily A member 1	Dnaja1
Typical	-1.492976316	9.97824E-05	Ig heavy chain V-III region J606;Ig heavy chain V-III region ABE-47N;Ig heavy chain V-III region U61;Ig heavy chain V-III region A4;Ig heavy chain V-III region HPC76;Ig heavy chain V region AMPC1;Ig heavy chain V-III region T957;Ig heavy chain V-III region W3082	Ighv6-3;Ighv6-6
Typical	-1.492418332	1.9151E-06	Nuclear factor NF-kappa-B p105 subunit;Nuclear factor NF-kappa-B p50 subunit	Nfkb1
Typical	-1.492337354	0.000123066	Protein kinase C iota type	Prkci
Typical	-1.490805393	3.85725E-06	Pumilio homolog 1	Pum1
Typical	-1.488632054	9.87161E-07	Biliverdin reductase A	Blvra
Typical	-1.486884223	4.10511E-06	Inter-alpha-trypsin inhibitor heavy chain H2	Itih2
Typical	-1.481098048	2.4758E-07	Ras-related GTP-binding protein B;Ras-related GTP-binding protein A	Rragb;Rraga
Typical	-1.472714255	7.82778E-06	High mobility group protein B1	Hmgb1
Typical	-1.466532114	1.40147E-09	Heat shock protein 105 kDa	Hsph1
Typical	-1.464445072	4.17692E-05	Inositol-1-monophosphatase;Inositol monophosphatase 1	Impa1
Typical	-1.46065801	5.88186E-07	Vigilin	Hdlbp
Typical	-1.457640584	3.34604E-06	Integrin alpha-X	Itgax
Typical	-1.45667589	1.90996E-08	Arginine--tRNA ligase, cytoplasmic	Rars1
Typical	-1.456102859	1.53497E-05	Arf-GAP with coiled-coil, ANK repeat and PH domain-containing protein 2;Arf-GAP with coiled-coil, ANK repeat and PH domain-containing protein	Acap2
Typical	-1.44590204	4.9046E-09	Proteasome subunit alpha type-4;Proteasome subunit alpha type	Psma4
Typical	-1.443195576	6.67095E-06	Voltage-gated hydrogen channel 1	Hvcn1
Typical	-1.441612816	2.44161E-08	Beta-hexosaminidase subunit beta	Hexb
Typical	-1.440004561	1.37488E-09	Kinesin-1 heavy chain;Kinesin-like protein	Kif5b
Typical	-1.437198046	1.69364E-05	Guanylate-binding protein 2	Gbp2
Typical	-1.435860422	2.95007E-06	DNA topoisomerase 3-alpha;DNA topoisomerase	Top3a
Typical	-1.43128696	1.32651E-08	Protein disulfide-isomerase A4	Pdia4
Typical	-1.423726039	1.11577E-06	N(G),N(G)-dimethylarginine dimethylaminohydrolase 1	Ddah1
Typical	-1.419615046	1.00057E-06	Small ribosomal subunit protein eS19	Rps19
Typical	-1.417944633	9.6319E-08	Guanylate-binding protein 7	Gbp7
Typical	-1.409728728	1.52473E-07	Alpha-N-acetylglucosaminidase	Naglu
Typical	-1.407035573	5.85018E-06	Ubiquitin-like protein 5	Ubl5;Ubl5b
Typical	-1.405308278	2.91464E-08	Inosine-5-monophosphate dehydrogenase 2;IMP dehydrogenase	Impdh2
Typical	-1.405275408	8.78422E-08	General vesicular transport factor p115	Uso1
Typical	-1.400436253	1.4801E-07	Methionine aminopeptidase 2	Metap2
Typical	-1.3962581	8.60189E-08	AP-1 complex subunit mu-1	Ap1m1
Typical	-1.380091434	5.97708E-08	Small ribosomal subunit protein uS5	Rps2
Typical	-1.377523571	2.30619E-08	Plastin-3	Pls3
Typical	-1.348080444	1.14678E-08	Eukaryotic translation initiation factor 3 subunit L	Eif3l
Latent	1.375610987	6.7082E-07	Tight junction protein ZO-1	Tjp1
Latent	1.390652741	4.8253E-09	Endoglin	Eng
Latent	1.391377555	2.15494E-07	4-hydroxybenzoate polyprenyltransferase, mitochondrial	Coq2
Latent	1.392004416	3.31527E-07	Latent-transforming growth factor beta-binding protein 4	Ltbp4
Latent	1.404696104	1.641E-07	Myosin-10	Myh10
Latent	1.407288742	6.87156E-08	Occludin	Ocln
Latent	1.415914154	9.37998E-07	Laminin subunit alpha-4	Lama4
Latent	1.453826226	1.24208E-06	Core histone macro-H2A.1	Macroh2a1
Latent	1.454068057	3.86717E-06	Cytochrome P450 4B1	Cyp4b1
Latent	1.456857321	8.05069E-07	Periostin	Postn
Latent	1.45700317	2.43423E-05	Alkaline phosphatase, tissue-nonspecific isozyme	Alpl
Latent	1.460938221	3.38034E-05	Integrin beta-1	Itgb1
Latent	1.473581759	2.95092E-07	Intercellular adhesion molecule 2	Icam2

Latent	1.479126506	7.20317E-05	Na(+)/H(+) exchange regulatory cofactor NHE-RF2;Na(+)/H(+) exchange regulatory cofactor NHE-RF	Nherf2
Latent	1.489447721	1.25326E-05	Zinc finger protein 22	Znf22
Latent	1.493239233	6.40705E-05	Peroxiredoxin-2	Prdx2
Latent	1.499288665	1.93348E-07	Myocyte-specific enhancer factor 2B	Mef2b
Latent	1.499696965	8.93498E-07	Ig kappa chain V-VI region NQ5-78.2.6;Ig kappa chain V-VI region NQ6-8.3.1;Ig kappa chain V-VI region NQ2-48.2.2;Ig kappa chain V-VI region NQ2-17.4.1	Igkv4-70;Igkv4-59
Latent	1.503442658	6.49877E-06	Cadherin-5	Cdh5
Latent	1.507104683	4.78978E-08	Beta-adducin	Add2
Latent	1.512753932	1.47691E-08	Plasmalemma vesicle-associated protein	Plvap
Latent	1.51316611	1.57562E-06	Collagen alpha-1(VI) chain	Col6a1
Latent	1.51552887	2.59621E-05	Trophoblast glycoprotein	Tpbg
Latent	1.517101818	9.08034E-06	Periaxin	Prx
Latent	1.522661421	0.000144363	CREB-regulated transcription coactivator 2	Crtc2
Latent	1.523446083	8.63215E-09	Junction plakoglobin	Jup
Latent	1.536011993	2.59347E-07	Integrin alpha-1	Itga1
Latent	1.541392771	4.66021E-06	Lamin-B2	Lmnb2
Latent	1.554393747	3.14492E-05	Phosphorylase b kinase gamma catalytic chain, liver/testis isoform	Phkg2
Latent	1.557449129	9.2828E-06	Collagen alpha-1(XVIII) chain;Endostatin;Non-collagenous domain 1	Col18a1
Latent	1.567060174	3.92233E-06	Flavin-containing monooxygenase 1	Fmo1
Latent	1.577518802	0.000215824	Dematin	Dmtn
Latent	1.578989601	6.30476E-10	Dedicator of cytokinesis protein 9	Dock9
Latent	1.585797479	1.06238E-05	Actin, alpha cardiac muscle 1;Actin, alpha cardiac muscle 1, intermediate form;Actin, aortic smooth muscle;Actin, aortic smooth muscle, intermediate form;Actin, alpha skeletal muscle;Actin, alpha skeletal muscle, intermediate form;Actin, gamma-enteric smooth muscle;Actin, gamma-enteric smooth muscle, intermediate form;Actin, aortic smooth muscle	Actc1;Acta2;Acta1;Actg2
Latent	1.586820539	0.000412348	Amyloid beta precursor like protein 2	Aplp2
Latent	1.590278498	2.79103E-06	Heat shock 70 kDa protein 12B	Hspa12b
Latent	1.604747518	8.36153E-05	Zinc finger protein ZXDC	Zxdc
Latent	1.607932663	5.92452E-09	Methyltransferase N6AMT1	N6amt1
Latent	1.609424973	0.000464326	Transmembrane protein 8B	Tmem8b
Latent	1.610651504	1.6048E-05	Tropomodulin-1	Tmod1
Latent	1.610820749	0.000225167	Titin	Ttn
Latent	1.611388948	0.000452047	Peroxisome proliferator-activated receptor gamma coactivator 1-alpha	Ppargc1a
Latent	1.61159473	0.00043145	Prostate tumor-overexpressed gene 1 protein homolog	Ptov1
Latent	1.616697502	1.48968E-05	Probable E3 ubiquitin-protein ligase TRIML1	Triml1
Latent	1.619572025	1.10676E-05	Prelamin-A/C;Lamin-A/C	Lmna
Latent	1.626526663	7.39739E-06	Mast cell protease 4	Mcpt4
Latent	1.630030696	0.000385901	5-AMP-activated protein kinase subunit gamma-2	Prkag2
Latent	1.630984476	0.000429798	Solute carrier family 22 member 23	Slc22a23
Latent	1.631373999	2.8303E-07	Collagen alpha-2(VI) chain	Col6a2
Latent	1.633845986	0.001508278	Ceramide kinase	Cerk
Latent	1.634335857	5.41524E-06	Semaphorin-3C	Sema3c
Latent	1.635600366	1.76032E-07	Musculoskeletal embryonic nuclear protein 1	Mustn1
Latent	1.636303775	0.001994443	Collagen, type IV, alpha 5	Col4a5
Latent	1.637570635	6.08E-08	Neprilysin	Mme
Latent	1.642687925	4.9691E-05	DNA excision repair protein ERCC-5	Ercc5
Latent	1.647240787	7.36523E-07	Laminin subunit beta-1	Lamb1
Latent	1.647938199	2.95784E-06	Centromere protein V	Cenpv
Latent	1.652313296	0.000801539	Paraneoplastic antigen Ma3 homolog	Pnma3
Latent	1.660589049	4.69682E-06	Collagen, type VI, alpha 3	Col6a3
Latent	1.670050579	0.003234124	Replication protein A 14 kDa subunit	Rpa3
Latent	1.674458758	0.000457632	DCN1-like protein 5;DCN1-like protein	Dcun1d5
Latent	1.679307895	0.001285044	Cilia- and flagella-associated protein 410	Cfap410
Latent	1.681772741	0.000131423	Transportin-2	Tnpo2
Latent	1.688236152	0.001038541	DNA-directed RNA polymerase I subunit RPA49	Polr1e
Latent	1.697584004	1.06742E-05	Fibrinogen gamma chain	Fgg
Latent	1.701957639	0.001406134	Iron-sulfur cluster assembly 1 homolog, mitochondrial	Isca1;AK157302
Latent	1.703148248	0.003369175	Suppressor of fused homolog	Sufu

Latent	1.706875102	0.000484659	Golgi associated RAB2 interactor family member 5B	Garin5b
Latent	1.707435502	2.08192E-06	Podocalyxin	Podxl
Latent	1.710299598	3.56783E-06	Angiotensin-converting enzyme;Angiotensin-converting enzyme, soluble form;Angiotensin-converting enzyme	Ace
Latent	1.7189092	1.16464E-05	Pyruvate kinase;Pyruvate kinase PKLR	Pklr
Latent	1.721480115	5.39741E-05	Thioredoxin reductase 2, mitochondrial;thioredoxin-disulfide reductase	Txnrd2
Latent	1.722311868	2.17584E-05	Solute carrier organic anion transporter family member 2A1	Slco2a1
Latent	1.729811011	0.004249535	Nuclear receptor subfamily 1 group D member 2	Nr1d2
Latent	1.733757401	3.24358E-07	Amine oxidase [copper-containing] 3;Amine oxidase	Aoc3
Latent	1.735501289	0.001156695	WW domain-containing oxidoreductase	Wwox
Latent	1.735911857	4.31468E-06	Stomatin	Stom
Latent	1.738769658	1.51687E-07	Core histone macro-H2A.2	Macroh2a2
Latent	1.740239249	9.00435E-07	Cadherin-13	Cdh13
Latent	1.753256861	0.00295064	Histone deacetylase;histone deacetylase	Hdac9
Latent	1.755856726	0.002568987	Profilin-2;Profilin	Pfn2
Latent	1.757800632	0.000163065	PHD and RING finger domain-containing protein 1	Phrf1
Latent	1.784498511	5.24233E-05	Caveolae-associated protein 1	Cavin1
Latent	1.793475066	2.56651E-07	Guanine nucleotide-binding protein G(o) subunit alpha	Gnao1
Latent	1.798641947	4.56361E-06	Fibrinogen beta chain;Fibrinopeptide B;Fibrinogen beta chain	Fgb
Latent	1.8021531	0.001370146	1-acyl-sn-glycerol-3-phosphate acyltransferase epsilon	Agpat5
Latent	1.803367466	9.84358E-07	Laminin subunit gamma-1	Lamc1
Latent	1.809978951	0.002844812	Jouberin	Ahi1
Latent	1.81132448	3.11097E-06	Dipeptidase 1;Dipeptidase	Dpep1
Latent	1.811359257	3.01218E-05	Fibrinogen alpha chain;Fibrinopeptide A;Fibrinogen alpha chain	Fga
Latent	1.821487766	6.75368E-10	Immunoglobulin kappa variable 6	Igkv6-32
Latent	1.825798607	0.00822543	Collagen alpha-4(IV) chain	Col4a4
Latent	1.835131539	0.012048577	Coiled-coil domain-containing protein 138	Ccdc138
Latent	1.839116753	0.003426794	Caveolin-2	Cav2
Latent	1.841620933	1.44871E-05	Protein 4.1	Epb41
Latent	1.853434308	0.00074244	Troponin I, cardiac muscle	Tnni3
Latent	1.857246611	0.000173745	Embryonal Fyn-associated substrate	Efs
Latent	1.859017881	2.20168E-08	Myocardial zonula adherens protein	Myzap
Latent	1.860892508	8.36708E-05	Chondroadherin	Chad
Latent	1.862632561	6.4322E-05	Claudin-5	Cldn5
Latent	1.863135465	7.89001E-06	EMI domain-containing protein 1	Emid1
Latent	1.86534712	9.13118E-07	Histone H1.0;Histone H1.0, N-terminally processed	H1-0
Latent	1.867451964	5.83326E-06	F-box only protein 31	Fbxo31
Latent	1.873334058	4.47258E-07	Collagen alpha-2(IV) chain;Canstatin	Col4a2
Latent	1.874737337	0.000279122	Growth/differentiation factor 5	Gdf5
Latent	1.881245189	0.000390796	Collagen alpha-1(XXIV) chain	Col24a1
Latent	1.886445215	0.001101117	Thrombomodulin	Thbd
Latent	1.88803162	0.000197274	Junctional adhesion molecule A	F11r
Latent	1.890669145	0.000418517	Zonadhesin	Zan
Latent	1.892673302	3.45279E-05	Myc target protein 1	Myct1
Latent	1.903503969	0.013883693	Protein shortage in chiasmata 1 ortholog	Shoc1
Latent	1.908367602	0.003091055	SPATS2-like protein	Spats2l
Latent	1.912422498	2.04103E-06	Caveolin-1;Caveolin	Cav1
Latent	1.914308696	4.55722E-05	Protein 4.2	Epb42
Latent	1.923463207	6.74993E-06	Caveolae-associated protein 2	Cavin2
Latent	1.933314747	0.017632189	5 exonuclease Apollo	Dclre1b
Latent	1.940881877	0.005085129	Serum response factor	Srf
Latent	1.943581369	0.00111154	Collagen alpha-1(V) chain	Col5a1
Latent	1.944350963	0.014866003	Uncharacterized serine/threonine-protein kinase SBK3	Sbk3
Latent	1.947248162	0.001935952	Sphingosine 1-phosphate receptor 5	S1pr5
Latent	1.947641839	0.0002576	Interleukin-1 receptor-associated kinase 3	Irak3
Latent	1.948813332	0.000387111	RNA N6-adenosine-methyltransferase METTL16;U6 small nuclear RNA (adenine-(43)-N(6))-methyltransferase	Mettl16
Latent	1.952822791	0.007385116	BEN domain-containing protein 5	Bend5
Latent	1.953542921	0.001218969	Proto-oncogene tyrosine-protein kinase ROS;receptor protein-tyrosine kinase	Ros1
Latent	1.954256524	0.008791025	Protocadherin beta 22	Pcdhb22
Latent	1.959621281	0.000608209	Fatty acid desaturase 3	Fads3

Latent	1.965615739	0.00358703	Trichohyalin-like protein 1	Tchhl1
Latent	1.973250304	0.001116447	Matrix Gla protein	Mgp
Latent	1.974287669	0.000476928	CD81 antigen	Cd81
Latent	1.981835471	4.72651E-09	Basement membrane-specific heparan sulfate proteoglycan core protein	Hspg2
Latent	1.986123	2.91134E-06	Carbonic anhydrase 2	Ca2
Latent	1.999818887	0.019798893	Insulin-like growth factor-binding protein 2	Igfbp2
Latent	2.002453974	0.013695117	Proline-rich nuclear receptor coactivator 2	Pnrc2
Latent	2.004092026	1.02079E-06	Nidogen-1	Nid1
Latent	2.004803297	9.45353E-09	Nephronectin	Npnt
Latent	2.01329085	6.5274E-09	Nidogen-2	Nid2
Latent	2.016631042	0.014739885	Phosphatidylinositol phosphatase PTPRQ	Ptprq
Latent	2.022193972	2.47318E-05	ATP-binding cassette sub-family G member 5	Abcg5
Latent	2.027290429	0.000411453	StAR-related lipid transfer protein 5	Stard5
Latent	2.030822733	0.000438457	RNA-binding protein 43	Rbm43
Latent	2.036438306	3.2362E-07	Large neutral amino acids transporter small subunit 3	Slc43a1
Latent	2.03819987	0.001053073	Epithelial splicing regulatory protein 1	Esrp1
Latent	2.039482986	0.002851306	Cilia- and flagella- associated protein 210	Cfap210
Latent	2.046581311	0.003520464	Oxysterol-binding protein-related protein 3;Oxysterol-binding protein	Osbpl3
Latent	2.047455703	0.0001851	Protein FAM149A	Fam149a
Latent	2.049120394	0.000138113	Unconventional myosin-XVIIIa	Myo18a
Latent	2.055310991	1.05445E-06	Protein eva-1 homolog B	Eva1b
Latent	2.067742263	0.012417725	Serine/threonine-protein kinase LATS2;non-specific serine/threonine protein kinase	Lats2;Gm49361
Latent	2.069819917	6.56766E-07	EKC/KEOPS complex subunit Lage3	Lage3
Latent	2.070658323	0.000229805	Ubiquitin-associated protein 1	Ubap1
Latent	2.075586912	0.000177411	Equilibrative nucleobase transporter 1	Slc43a3
Latent	2.090355025	0.023347124	Mitochondrial ornithine transporter 1	Slc25a15
Latent	2.091884041	5.10596E-05	Paired-Ig-like receptor A12	Pira12
Latent	2.104089991	0.01252968	DDB1- and CUL4-associated factor 6	Dcaf6
Latent	2.111127578	2.14185E-05	Elastin	Eln
Latent	2.11355114	0.000529625	Advanced glycosylation end product-specific receptor	Ager
Latent	2.11382779	5.22768E-10	Laminin subunit beta-3	Lamb3
Latent	2.117530039	0.000787129	Golgi phosphoprotein 3-like	Golph3l
Latent	2.12127552	0.000335319	Troponin C, slow skeletal and cardiac muscles	Tnnc1
Latent	2.122021866	1.23435E-08	Laminin subunit beta-2	Lamb2
Latent	2.125063366	0.000214433	Myosin regulatory light chain 2, atrial isoform	Myl7
Latent	2.129205873	0.007034497	Cyclin-L1	Ccnl1
Latent	2.135944303	7.09757E-08	Laminin subunit alpha-5	Lama5
Latent	2.142669233	0.002191913	Chemokine subfamily B Cys-X-Cys	Ppbp
Latent	2.154385757	1.54543E-06	Tubulointerstitial nephritis antigen-like	Tinagl1
Latent	2.170938407	0.003248228	Protein BNIP5	Bnip5
Latent	2.17623522	0.006204371	Rab GTPase-activating protein 1-like	Rabgap1l
Latent	2.181080945	3.46877E-05	Tubulin--tyrosine ligase	Ttl
Latent	2.193730799	4.8829E-09	Laminin subunit alpha-3	Lama3
Latent	2.199000676	3.58168E-05	Tetraspanin;Tetraspanin-7	Tspan7
Latent	2.199518882	3.35499E-06	Mimecan	Ogn
Latent	2.204507891	0.000408339	Zinc finger protein 235	Zfp235
Latent	2.209409184	1.34337E-07	Laminin subunit gamma-2	Lamc2
Latent	2.215907817	0.001742482	Vacuolar protein sorting-associated protein 37A	Vps37a
Latent	2.224802611	0.002295833	HAUS augmin-like complex, subunit 6	Haus6
Latent	2.225037956	0.001713555	Amine oxidase	Aoc1l1
Latent	2.240926128	0.000513224	UPF0729 protein C18orf32 homolog	Q91WE4
Latent	2.247533332	1.19382E-09	Spectrin beta chain;Spectrin beta chain, erythrocytic	Sptb
Latent	2.247790994	1.38607E-07	Integrin alpha-8;Integrin alpha-8 heavy chain;Integrin alpha-8 light chain	Itga8
Latent	2.269231796	7.87569E-08	Alpha-actinin-2	Actn2
Latent	2.276579391	3.86321E-07	Ankyrin-1	Ank1
Latent	2.28316644	9.43688E-06	Bisphosphoglycerate mutase;Phosphoglycerate mutase	Bpgm
Latent	2.284688695	1.44142E-08	Spectrin alpha chain, erythrocytic 1	Spta1
Latent	2.293088256	0.001127052	PACRG-like protein	Pacrgl;5730480H06Rik
Latent	2.303103595	7.80278E-07	Collagen alpha-2(V) chain	Col5a2

Latent	2.334331852	0.017686302	Small ribosomal subunit protein uS2m	Mrps2
Latent	2.334463416	3.11755E-05	Flavin reductase (NADPH)	Blvrb
Latent	2.339519374	0.002940225	Chondroitin sulfate synthase 1	Chsy1
Latent	2.35106483	0.000859421	Cell adhesion molecule DSCAM	Dscam
Latent	2.355555068	0.01793516	Toll-like receptor 8	Tlr8
Latent	2.361667972	0.042195895	Zinc finger protein 579	Znf579;Zfp579
Latent	2.365160857	0.038232348	Homeodomain-interacting protein kinase 2	Hipk2
Latent	2.397556326	0.000235498	H(+)/Cl(-) exchange transporter 5	Clcn5
Latent	2.416130702	0.001481356	LINE-1 retrotransposable element ORF2 protein;Reverse transcriptase;Endonuclease	Pol
Latent	2.429814466	0.00469804	Fasciculation and elongation protein zeta-2	Fez2
Latent	2.43725552	0.00215117	Atlastin-1	Atl1
Latent	2.464892917	0.000123966	Prostamide/prostaglandin F synthase	Prxl2b
Latent	2.4806491	0.000961655	Tubby-like protein;Tubby-related protein 1	Tulp1
Latent	2.481369993	0.00621394	Dachshund homolog 2	Dach2
Latent	2.508330663	0.019741103	IQ motif and ankyrin repeat domain-containing protein 1	Iqank1
Latent	2.534683673	7.03999E-08	T-cell activation Rho GTPase-activating protein;T-cell activation GTPase-activating protein 1	Tagap;Tagap1
Latent	2.542245166	2.00479E-06	Alpha-synuclein	Snca
Latent	2.561633301	7.61508E-09	Aquaporin-1	Aqp1
Latent	2.563952933	0.000346552	Rho GTPase-activating protein 8	Arhgap8
Latent	2.566963535	0.000253386	Tyrosine-protein phosphatase non-receptor type 4;protein-tyrosine-phosphatase	Ptpn4
Latent	2.575354407	0.009451113	Cerebral cavernous malformations 2 protein-like	Ccm2l
Latent	2.585866589	2.74784E-08	Band 3 anion transport protein	Slc4a1
Latent	2.619236861	3.66046E-08	Carbonic anhydrase 1	Ca1
Latent	2.622818714	4.01435E-07	ADP-ribosylation factor-like protein 6	Arl6
Latent	2.637774489	0.000277962	Platelet glycoprotein Ib beta chain	Gp1bb
Latent	2.656313769	0.000212937	Ammonium transporter Rh type A	Rhag
Latent	2.664561356	0.000251947	Myelin protein P0	Mpz
Latent	2.713152207	6.1248E-06	Syntaxin-19	Stx19
Latent	2.738465733	3.17115E-08	Basement membrane-specific heparan sulfate proteoglycan core protein;Endorepellin;LG3 peptide	Hspg2
Latent	2.753483136	0.084967338	EH domain-binding protein 1	Ehbp1
Latent	2.769891887	1.41798E-05	Voltage-dependent L-type calcium channel subunit alpha;Voltage-dependent L-type calcium channel subunit alpha-1F	Cacna1f
Latent	2.787144915	1.43121E-07	Phosphoinositide 3-kinase adapter protein 1	Pik3ap1
Latent	2.809102228	0.000925626	BAI1-associated protein 3	Baiap3
Latent	2.853356531	1.92335E-08	Decorin	Dcn
Latent	2.894348802	0.000283574	AT-rich interactive domain-containing protein 3B;AT-rich interactive domain-containing protein 3	Arid3b
Latent	2.908590974	0.007794486	Immunoglobulin superfamily DCC subclass member 3	Igdcc3
Latent	2.964824592	0.005974423	1-phosphatidylinositol 4,5-bisphosphate phosphodiesterase eta-1;Phosphoinositide phospholipase C	Plch1
Latent	2.98845395	1.25581E-05	Protein Smaug homolog 2	Samd4b
Latent	2.989407878	1.24934E-06	Transmembrane protein 14C	Tmem14c
Latent	3.0131734	0.002674369	N-terminal Xaa-Pro-Lys N-methyltransferase 1;N-terminal Xaa-Pro-Lys N-methyltransferase 1, N-terminally processed;N-terminal Xaa-Pro-Lys N-methyltransferase 1	Ntmt1
Latent	3.032787132	9.38041E-05	RILP-like protein 2	Rilpl2
Latent	3.087810813	7.07329E-09	Aquaporin-5	Aqp5
Latent	3.110277727	0.001298387	Pleckstrin homology domain-containing family G member 2	Plekhg2
Latent	3.162230958	4.40565E-07	Myosin-6	Myh6
Latent	3.162583775	5.74654E-08	EEF1A lysine methyltransferase 2	Eef1akmt2
Latent	3.167682351	1.30283E-06	Tetratricopeptide repeat protein 9C	Ttc9c
Latent	3.177583588	9.99432E-07	Collagen alpha-2(I) chain	Col1a2
Latent	3.237136163	0.00922613	Speriolin	Spatc1
Latent	3.249770588	0.001278502	Uncharacterized protein C7orf50 homolog	3110082I17Rik
Latent	3.294893286	6.8356E-08	Collagen alpha-1(I) chain	Col1a1
Latent	3.313652717	1.28399E-05	Axonemal dynein light intermediate polypeptide 1	Dnali1
Latent	3.38284075	0.050584993	Leiomodin-3	Lmod3

Latent	3.941265572	0.00079899	Cell cycle exit and neuronal differentiation protein 1	Cend1
Latent	4.130877113	4.30039E-06	Myosin light chain 4	Myl4

Murine Proteins: Latent versus Hypervirulent				
**Enriched in:**	**Fold Change**	**p-value**	**Protein Name**	**Gene**
Hypervirulent	-4.444219017	3.81484E-15	Glutathione S-transferase omega-1;Glutathione S-transferase omega	Gsto1
Hypervirulent	-4.230661964	3.14631E-07	Myoglobin	Mb
Hypervirulent	-4.093535614	9.66122E-08	Protein S100-A9	S100a9
Hypervirulent	-3.907828522	5.03993E-09	Alpha-2-HS-glycoprotein	Ahsg
Hypervirulent	-3.798801422	1.68846E-06	Protein S100-A8	S100a8
Hypervirulent	-3.781996155	3.14557E-08	Leucine-rich alpha-2-glycoprotein 1	Lrg1
Hypervirulent	-3.773666	4.38113E-11	L-lactate dehydrogenase B chain;L-lactate dehydrogenase	Ldhb
Hypervirulent	-3.679283333	1.66999E-09	Astrocytic phosphoprotein PEA-15	Pea15;Pea15a
Hypervirulent	-3.621226311	2.23241E-12	m7GpppX diphosphatase	Dcps
Hypervirulent	-3.538742447	9.12504E-07	Protein S100-G	S100g
Hypervirulent	-3.438600731	4.94806E-12	Glutathione S-transferase A3	Gsta3
Hypervirulent	-3.411320114	9.12537E-13	Serotransferrin	Tf
Hypervirulent	-3.389738655	1.62213E-11	Argininosuccinate synthase	Ass1
Hypervirulent	-3.360971642	1.44204E-12	Hemopexin	Hpx
Hypervirulent	-3.349352837	4.16223E-06	Tubulin beta-3 chain	Tubb3
Hypervirulent	-3.343748093	8.65641E-18	Fatty acid synthase;[Acyl-carrier-protein] S-acetyltransferase;[Acyl-carrier-protein] S-malonyltransferase;3-oxoacyl-[acyl-carrier-protein] synthase;3-oxoacyl-[acyl-carrier-protein] reductase;3-hydroxyacyl-[acyl-carrier-protein] dehydratase;Enoyl-[acyl-carrier-protein] reductase;Acyl-[acyl-carrier-protein] hydrolase;Fatty acid synthase	Fasn
Hypervirulent	-3.342450523	7.59947E-13	Xanthine dehydrogenase/oxidase;Xanthine dehydrogenase;Xanthine oxidase	Xdh
Hypervirulent	-3.320318222	8.29315E-13	Indolethylamine N-methyltransferase	Inmt
Hypervirulent	-3.283625984	2.32426E-09	Kininogen-1;Kininogen-1 heavy chain;Bradykinin;Kininogen-1 light chain	Kng1
Hypervirulent	-3.281856728	2.30798E-11	Albumin	Alb
Hypervirulent	-3.25615406	8.38379E-09	Carboxylesterase 1C	Ces1c
Hypervirulent	-3.214826965	1.94068E-10	Fumarylacetoacetase	Fah
Hypervirulent	-3.197944069	6.8989E-06	Ig gamma-1 chain C region secreted form;Ig gamma-1 chain C region, membrane-bound form	Ighg1
Hypervirulent	-3.190671349	4.31275E-10	SEC14-like 3	Sec14l3
Hypervirulent	-3.179518509	0.00080134	Ankyrin repeat domain-containing protein 49	Ankrd49
Hypervirulent	-3.177906609	4.61136E-12	Pregnancy zone protein;Alpha-2-macroglobulin 165 kDa subunit;Alpha-2-macroglobulin 35 kDa subunit	Pzp
Hypervirulent	-3.156196404	0.001035509	Thymosin beta-10;Thymosin beta-4;Hemoregulatory peptide AcSDKP	Tmsb10;Tmsb4x
Hypervirulent	-3.133503723	3.60745E-05	Fetuin-B	Fetub
Hypervirulent	-3.096251488	0.000885598	Prothymosin alpha;Prothymosin alpha, N-terminally processed;Thymosin alpha	Ptma
Hypervirulent	-3.076107788	9.22276E-14	Aldo-keto reductase family 1 member B1	Akr1b1
Hypervirulent	-3.065190697	8.67771E-11	Serine (Or cysteine) peptidase inhibitor, clade B, member 9	Serpinb9
Hypervirulent	-3.03598156	1.17466E-08	Putative protein-lysine deacylase ABHD14B	Abhd14b
Hypervirulent	-3.028243637	3.3626E-11	Leukocyte elastase inhibitor A	Serpinb1a
Hypervirulent	-2.999603844	1.82017E-07	Carbonic anhydrase 3	Ca3
Hypervirulent	-2.985351753	6.40802E-13	Elongation factor 2	Eef2
Hypervirulent	-2.976700211	2.79081E-12	NADP-dependent malic enzyme;Malic enzyme	Me1
Hypervirulent	-2.947999573	1.57569E-08	Carboxylic ester hydrolase	Ces1b
Hypervirulent	-2.94712162	2.58456E-10	Homeodomain-only protein	Hopx
Hypervirulent	-2.93883934	1.25107E-09	Hepatoma-derived growth factor	Hdgf
Hypervirulent	-2.932906342	2.4471E-14	Transketolase;transketolase	Tkt
Hypervirulent	-2.926244164	7.29427E-07	Arginase-1	Arg1
Hypervirulent	-2.92461071	3.44123E-11	Peroxiredoxin-6	Prdx6
Hypervirulent	-2.916851807	1.11297E-09	Transthyretin	Ttr
Hypervirulent	-2.90991497	1.36832E-09	Glutathione S-transferase Mu 1;Glutathione S-transferase;glutathione transferase	Gstm1
Hypervirulent	-2.899230957	3.91977E-09	Four and a half LIM domains protein 1	Fhl1
Hypervirulent	-2.893774223	2.73846E-09	Phosphoglycerate mutase 1	Pgam1
Hypervirulent	-2.869651794	2.71825E-10	Complement C2;Complement factor B;Complement factor B Ba fragment;Complement factor B Bb fragment;Complement factor B	Gm20547;Cfb
Hypervirulent	-2.848779106	2.93282E-09	Plastin-2	Lcp1
Hypervirulent	-2.842346191	5.81462E-08	Protein S100-A6	S100a6

Hypervirulent	-2.836982346	5.92168E-11	Cytosolic non-specific dipeptidase	Cndp2
Hypervirulent	-2.835121346	1.17096E-07	Neutrophilic granule protein	Ngp
Hypervirulent	-2.834073067	5.13009E-08	Guanylate-binding protein 1	Gbp1;Gbp2b
Hypervirulent	-2.833704948	5.33094E-13	Vitamin D-binding protein	Gc
Hypervirulent	-2.824541092	0.000795903	Tubulin alpha-1B chain;Detyrosinated tubulin alpha-1B chain	Tuba1b
Hypervirulent	-2.818420792	6.43982E-10	L-xylulose reductase	Dcxr
Hypervirulent	-2.804602623	2.06107E-08	Lactotransferrin	Ltf
Hypervirulent	-2.798124886	3.78137E-07	Myeloblastin	Prtn3
Hypervirulent	-2.796834946	3.02642E-07	Profilin-1;Profilin	Pfn1
Hypervirulent	-2.778324509	8.45487E-09	Signal transducer and activator of transcription;Signal transducer and activator of transcription 1	Stat1
Hypervirulent	-2.772330475	3.22102E-13	ATP-citrate synthase	Acly
Hypervirulent	-2.758039856	7.57003E-11	Pyruvate kinase PKM	Pkm
Hypervirulent	-2.745597267	4.08035E-08	Transaldolase	Taldo1
Hypervirulent	-2.736871529	3.03581E-09	Rho GDP-dissociation inhibitor 2	Arhgdib
Hypervirulent	-2.731684303	2.78483E-08	Proteasome subunit beta type-10	Psmb10
Hypervirulent	-2.728494263	2.95544E-05	Serum amyloid P-component	Apcs
Hypervirulent	-2.725213814	2.04776E-09	Peptidyl-prolyl cis-trans isomerase FKBP1A	Fkbp1a
Hypervirulent	-2.714027214	1.2893E-07	Afamin	Afm
Hypervirulent	-2.70498333	8.69467E-09	Alcohol dehydrogenase 1	Adh1
Hypervirulent	-2.689461327	1.65656E-08	Ribonuclease inhibitor	Rnh1
Hypervirulent	-2.678481483	3.32355E-09	Glutathione S-transferase Mu 2;Glutathione S-transferase	Gstm2
Hypervirulent	-2.661478233	5.34105E-08	Ig gamma-2A chain C region, membrane-bound form;Ig gamma-2A chain C region, A allele	Igh-1a;Ighg
Hypervirulent	-2.65761776	8.91295E-09	Myeloperoxidase;Myeloperoxidase light chain;Myeloperoxidase heavy chain	Mpo
Hypervirulent	-2.653632927	4.88334E-11	Deoxynucleoside triphosphate triphosphohydrolase SAMHD1	Samhd1
Hypervirulent	-2.650187492	3.85704E-07	Peptidyl-prolyl cis-trans isomerase FKBP3;peptidylprolyl isomerase	Fkbp3
Hypervirulent	-2.642530823	4.60993E-12	Aspartate aminotransferase, cytoplasmic	Got1
Hypervirulent	-2.636253357	3.90306E-12	Cytoplasmic aconitate hydratase	Aco1
Hypervirulent	-2.6273592	1.04513E-07	Charged multivesicular body protein 4b	Chmp4b
Hypervirulent	-2.620195198	3.34989E-09	Macrophage-capping protein	Capg
Hypervirulent	-2.616108704	5.37791E-10	Thioredoxin reductase 1, cytoplasmic	Txnrd1
Hypervirulent	-2.609730721	3.23859E-05	Adenylate kinase isoenzyme 1;nucleoside-triphosphate--adenylate kinase	Ak1
Hypervirulent	-2.597907257	9.02834E-07	Ras-related C3 botulinum toxin substrate 2	Rac2
Hypervirulent	-2.589510918	4.07253E-12	Coatomer subunit delta	Arcn1
Hypervirulent	-2.587700462	5.68297E-09	Coronin-1A;Coronin	Coro1a
Hypervirulent	-2.580221748	1.23229E-09	Chitinase-3-like protein 1	Chi3l1
Hypervirulent	-2.573729706	2.86132E-10	Isocitrate dehydrogenase [NADP] cytoplasmic	Idh1
Hypervirulent	-2.571541786	2.35443E-07	DAZ-associated protein 1	Dazap1
Hypervirulent	-2.571219826	2.2444E-10	Malate dehydrogenase, cytoplasmic;Malate dehydrogenase	Mdh1
Hypervirulent	-2.568120575	4.05341E-07	Coactosin-like protein	Cotl1
Hypervirulent	-2.566945457	6.50095E-09	Chloride intracellular channel protein 1	Clic1
Hypervirulent	-2.565865707	4.12043E-08	Acidic leucine-rich nuclear phosphoprotein 32 family member B	Anp32b
Hypervirulent	-2.562168503	2.94767E-06	Ferritin heavy chain;Ferritin heavy chain, N-terminally processed;Ferritin	Fth1
Hypervirulent	-2.558031082	2.45604E-09	Annexin A3	Anxa3
Hypervirulent	-2.552444649	8.30484E-06	Chitinase-like protein 4	Chil4
Hypervirulent	-2.547634888	3.00629E-07	Nuclear ubiquitous casein and cyclin-dependent kinase substrate 1	Nucks1
Hypervirulent	-2.544744301	8.38039E-08	Ester hydrolase C11orf54 homolog	4931406C07Rik
Hypervirulent	-2.544161606	5.74108E-08	Glucose-6-phosphate isomerase	Gpi
Hypervirulent	-2.53831749	4.01978E-06	Inositol polyphosphate 1-phosphatase	Inpp1
Hypervirulent	-2.529198456	8.24136E-11	Serpin B6	Serpinb6a;Serpinb6
Hypervirulent	-2.520018196	4.36446E-11	Farnesyl pyrophosphate synthase	Fdps
Hypervirulent	-2.51922226	3.93364E-07	Aldehyde dehydrogenase, cytosolic 1	Aldh1a7
Hypervirulent	-2.518000221	9.16157E-10	Myotrophin	Mtpn
Hypervirulent	-2.514768791	2.27592E-05	Alpha-1-antitrypsin 1-2	Serpina1b
Hypervirulent	-2.506560516	7.43187E-09	Glycerol-3-phosphate dehydrogenase [NAD(+)], cytoplasmic;Glycerol-3-phosphate dehydrogenase [NAD(+)]	Gpd1
Hypervirulent	-2.503651237	1.79152E-07	Parathymosin	Ptms
Hypervirulent	-2.498278427	3.52137E-08	Fatty acid-binding protein, adipocyte	Fabp4

Hypervirulent	-2.495317841	3.14102E-06	Cytosolic acyl coenzyme A thioester hydrolase	Acot7
Hypervirulent	-2.481636429	1.63819E-09	Heat shock protein HSP 90-alpha	Hsp90aa1
Hypervirulent	-2.471858597	2.05352E-05	Interferon-inducible GTPase 1	Iigp1
Hypervirulent	-2.46505928	1.17795E-06	Serine (Or cysteine) peptidase inhibitor, clade B, member 6B	Serpinb6b
Hypervirulent	-2.459755898	0.000239866	Ig heavy chain V region 914	
Hypervirulent	-2.457427788	3.53756E-06	Pterin-4-alpha-carbinolamine dehydratase	Pcbd1
Hypervirulent	-2.456608391	1.04868E-08	Phosphoglucomutase-1	Pgm1
Hypervirulent	-2.455794144	1.4933E-08	Tryptophan--tRNA ligase, cytoplasmic;T1-TrpRS;T2-TrpRS	Wars1
Hypervirulent	-2.449146461	4.88528E-11	Heat shock protein HSP 90-beta	Hsp90ab1
Hypervirulent	-2.449077225	5.37248E-09	ADP-ribosylation factor 4	Arf4
Hypervirulent	-2.444177818	1.12831E-08	Acidic leucine-rich nuclear phosphoprotein 32 family member A;Acidic leucine-rich nuclear phosphoprotein 32 family member	Anp32a
Hypervirulent	-2.43152771	8.51774E-07	Alpha-1B-glycoprotein	A1bg
Hypervirulent	-2.426592445	1.56805E-05	Corticosteroid-binding globulin	Serpina6
Hypervirulent	-2.413357925	4.76947E-05	Xaa-Pro dipeptidase	Pepd
Hypervirulent	-2.404998016	8.19304E-10	Elongation factor 1-alpha 1	Eef1a1
Hypervirulent	-2.400605774	4.90862E-12	Valine--tRNA ligase;valine--tRNA ligase	Vars1
Hypervirulent	-2.395155716	1.46773E-07	Carbonyl reductase [NADPH] 3	Cbr3
Hypervirulent	-2.387531853	6.37579E-11	Leukotriene A-4 hydrolase	Lta4h
Hypervirulent	-2.377426147	4.85703E-08	Methanethiol oxidase;Selenium-binding protein 2	Selenbp1;Selenbp2
Hypervirulent	-2.377301598	2.70984E-07	Ubiquitin-like modifier-activating enzyme ATG7	Atg7
Hypervirulent	-2.364990997	6.79582E-10	GTPase IMAP family member 4	Gimap4
Hypervirulent	-2.363425446	3.23804E-12	14-3-3 protein theta	Ywhaq
Hypervirulent	-2.350796318	1.04384E-06	Neutrophil elastase	Elane
Hypervirulent	-2.349322701	6.24888E-11	Transgelin-2	Tagln2
Hypervirulent	-2.34887867	4.13219E-09	Tyrosine-protein phosphatase non-receptor type 6	Ptpn6
Hypervirulent	-2.348045731	1.2111E-08	Bromodomain testis-specific protein	Brdt
Hypervirulent	-2.346667862	4.96152E-05	Ig lambda-1 chain C region	Iglc1
Hypervirulent	-2.345416832	1.45576E-06	Neutrophil gelatinase-associated lipocalin	Lcn2
Hypervirulent	-2.337838364	6.68023E-08	Dihydropyrimidinase-related protein 2	Dpysl2
Hypervirulent	-2.326345634	7.43333E-09	Tubulin-folding cofactor B	Tbcb
Hypervirulent	-2.315270424	2.93818E-08	Hypoxanthine-guanine phosphoribosyltransferase	Hprt1
Hypervirulent	-2.314618874	6.2697E-07	Selenide, water dikinase 1	Sephs1
Hypervirulent	-2.314068222	1.28007E-06	Fatty acid-binding protein 5	Fabp5
Hypervirulent	-2.313287735	5.08776E-08	Ubiquitin-conjugating enzyme E2 Z	Ube2z
Hypervirulent	-2.308351135	4.86809E-06	AP-3 complex subunit sigma-1	Ap3s1
Hypervirulent	-2.307668304	3.40965E-08	CTP synthase 1	Ctps1
Hypervirulent	-2.307511139	2.00569E-10	L-lactate dehydrogenase A chain;L-lactate dehydrogenase	Ldha
Hypervirulent	-2.30677681	1.16699E-07	Partner of Y14 and mago	Pym1
Hypervirulent	-2.302247238	0.003381536	Eosinophil-associated, ribonuclease A family, member 6	Ear6
Hypervirulent	-2.298529053	8.81273E-09	Coatomer subunit gamma-1	Copg1
Hypervirulent	-2.29248333	2.63248E-08	Dual specificity protein phosphatase 3;Dual specificity protein phosphatase;protein-serine/threonine phosphatase	Dusp3
Hypervirulent	-2.292428017	0.000138813	Cystatin-B	Cstb
Hypervirulent	-2.292043114	1.88269E-09	Guanylate-binding protein 7	Gbp7
Hypervirulent	-2.28946476	2.60718E-07	Cysteine and glycine-rich protein 1	Csrp1
Hypervirulent	-2.283170319	0.000568599	Protein CutA	Cuta
Hypervirulent	-2.28276062	5.60426E-11	Triosephosphate isomerase	Tpi1
Hypervirulent	-2.282312393	2.83027E-07	Guanylate-binding protein 3	Gbp3
Hypervirulent	-2.282063866	7.66516E-09	SUMO-activating enzyme subunit 2	Uba2
Hypervirulent	-2.280328941	1.75841E-11	6-phosphogluconate dehydrogenase, decarboxylating	Pgd
Hypervirulent	-2.273036194	1.0309E-07	Calponin-3;Calponin	Cnn3
Hypervirulent	-2.270433044	0.132050042	Tubulin alpha-1C chain;Detyrosinated tubulin alpha-1C chain;Tubulin alpha chain	Tuba1c;Gm49450
Hypervirulent	-2.267702484	2.70845E-06	C-C chemokine receptor type 1	Ccr1
Hypervirulent	-2.264391708	1.33074E-07	Ribosome maturation protein SBDS	Sbds
Hypervirulent	-2.263584137	1.18185E-07	Proliferating cell nuclear antigen	Pcna
Hypervirulent	-2.262076187	9.45619E-10	Peptidyl-prolyl cis-trans isomerase FKBP4;Peptidyl-prolyl cis-trans isomerase FKBP4, N-terminally processed;peptidylprolyl isomerase	Fkbp4
Hypervirulent	-2.259462547	1.62185E-07	Tubulin polymerization-promoting protein family member 3	Tppp3

Hypervirulent	-2.250266266	2.09539E-05	Glia maturation factor gamma;Glia maturation factor	Gmfg
Hypervirulent	-2.246278381	5.69968E-06	Fatty acid-binding protein, heart	Fabp3
Hypervirulent	-2.245138741	9.80232E-07	Pyridoxal kinase	Pdxk
Hypervirulent	-2.240453148	1.39986E-10	Poly(rC)-binding protein 2	Pcbp2
Hypervirulent	-2.238996315	2.6586E-06	Sorting nexin-6;Sorting nexin-6, N-terminally processed	Snx6
Hypervirulent	-2.23717804	3.11723E-10	Echinoderm microtubule-associated protein-like 2	Eml2
Hypervirulent	-2.231936073	7.65154E-05	Small ubiquitin-related modifier 2;Small ubiquitin-related modifier 3;Small ubiquitin-related modifier	Sumo2;Sumo3;Gm49325
Hypervirulent	-2.223509789	0.024844998	ATP synthase subunit O, mitochondrial	Atp5po
Hypervirulent	-2.22322998	5.6278E-07	Ferritin light chain 1;Ferritin;Ferritin light chain 2	Ftl1;Ftl2
Hypervirulent	-2.214826393	2.32043E-09	Testin	Tes
Hypervirulent	-2.214398766	5.18404E-06	Large ribosomal subunit protein eL38	Rpl38
Hypervirulent	-2.209884644	8.41004E-05	Immunoglobulin heavy constant gamma 2B	Ighg2b;Ighg2c
Hypervirulent	-2.207728767	1.50372E-06	DNA repair nuclease/redox regulator APEX1;DNA repair nuclease/redox regulator APEX1, mitochondrial;DNA-(apurinic or apyrimidinic site) endonuclease	Apex1
Hypervirulent	-2.200776863	6.41155E-08	Alpha-1-antitrypsin 1-4	Serpina1d
Hypervirulent	-2.199144745	0.000274601	Tetratricopeptide repeat protein 21A	Ttc21a
Hypervirulent	-2.194093704	6.98675E-09	UTP--glucose-1-phosphate uridylyltransferase	Ugp2
Hypervirulent	-2.193840599	3.06628E-07	Destrin	Dstn
Hypervirulent	-2.184612083	0.000118892	Resistin-like alpha	Retnla
Hypervirulent	-2.182744217	1.06984E-05	Immunoglobulin heavy variable 9	Ighv9-4;Ighv9-1;Ighv9-3;Ighv9-2
Hypervirulent	-2.180503654	0.000180855	Phosphoglycerate mutase 2	Pgam2
Hypervirulent	-2.1784235	3.07217E-06	MYG1 exonuclease	Myg1
Hypervirulent	-2.172509193	4.8692E-08	Aldo-keto reductase family 1 member A1	Akr1a1
Hypervirulent	-2.171499252	2.47858E-05	Protein SET	Set
Hypervirulent	-2.158523941	2.76156E-09	Signal transducer and activator of transcription 3;Signal transducer and activator of transcription	Stat3
Hypervirulent	-2.155062294	5.59041E-11	LIM and SH3 domain protein 1	Lasp1
Hypervirulent	-2.153440666	3.08192E-06	Tubulin-specific chaperone A	Tbca
Hypervirulent	-2.152613831	6.77833E-07	Haptoglobin;Haptoglobin alpha chain;Haptoglobin beta chain	Hp
Hypervirulent	-2.149247169	1.44552E-09	von Willebrand factor A domain-containing protein 5A	Vwa5a
Hypervirulent	-2.142235374	1.36485E-09	Glyceraldehyde-3-phosphate dehydrogenase	Gapdh;Gapdhrt;Gapdhrt2
Hypervirulent	-2.140872192	0.000527574	T-cell-specific guanine nucleotide triphosphate-binding protein 1;T-cell-specific guanine nucleotide triphosphate-binding protein 2	Tgtp1;Tgtp2
Hypervirulent	-2.138528252	2.2935E-08	Bifunctional phosphoribosylaminoimidazole carboxylase/phosphoribosylaminoimidazole succinocarboxamide synthetase;Phosphoribosylaminoimidazole carboxylase;Phosphoribosylaminoimidazole succinocarboxamide synthetase	Paics
Hypervirulent	-2.131761742	5.61077E-09	Aldo-keto reductase family 1, member B10	Akr1b10
Hypervirulent	-2.126699638	5.2596E-12	V-type proton ATPase subunit B, brain isoform	Atp6v1b2
Hypervirulent	-2.123912048	0.001120295	Nuclear migration protein nudC	Nudc
Hypervirulent	-2.12384491	3.20711E-10	Glycogen phosphorylase, brain form	Pygb
Hypervirulent	-2.12030735	2.21797E-07	Proteasome subunit beta type-9	Psmb9
Hypervirulent	-2.119765663	4.02856E-06	14-3-3 protein gamma;14-3-3 protein gamma, N-terminally processed	Ywhag
Hypervirulent	-2.116608047	6.56965E-07	Mannose-1-phosphate guanyltransferase alpha	Gmppa
Hypervirulent	-2.114967346	3.32493E-05	Glutamine--fructose-6-phosphate aminotransferase [isomerizing] 1	Gfpt1
Hypervirulent	-2.110175133	7.83539E-08	Fructose-bisphosphate aldolase A;Fructose-bisphosphate aldolase	Aldoa;Aldoart1
Hypervirulent	-2.10673542	0.00087464	Beta-enolase;phosphopyruvate hydratase	Eno3
Hypervirulent	-2.102618217	0.001908326	Uteroglobin	Scgb1a1
Hypervirulent	-2.101598167	1.16372E-08	Hematopoietic lineage cell-specific protein	Hcls1
Hypervirulent	-2.101463699	7.7933E-14	Threonine--tRNA ligase 1, cytoplasmic	Tars1
Hypervirulent	-2.100554276	5.17267E-08	Alcohol dehydrogenase class-3	Adh5
Hypervirulent	-2.098134422	4.60466E-11	Phosphoglycerate kinase 1;Phosphoglycerate kinase	Pgk1
Hypervirulent	-2.093214035	4.94211E-08	Osteoclast-stimulating factor 1	Ostf1
Hypervirulent	-2.0919487	1.55766E-06	Inositol monophosphatase 2	Impa2
Hypervirulent	-2.086496735	2.72957E-06	Farnesyl pyrophosphate synthase	Fdps
Hypervirulent	-2.085538864	1.00635E-08	Glycogen phosphorylase, liver form;Alpha-1,4 glucan phosphorylase	Pygl

Hypervirulent	-2.081722832	4.81891E-08	Cofilin-1	Cfl1
Hypervirulent	-2.081654739	4.79301E-06	Creatine kinase B-type	Ckb
Hypervirulent	-2.081433487	3.54366E-06	Proteasomal ubiquitin receptor ADRM1	Adrm1;Adrm1b
Hypervirulent	-2.079177094	0.001971214	Immunoglobulin heavy constant alpha chain	Igha
Hypervirulent	-2.078904343	1.95955E-10	Adapter Sh3bgrl	Sh3bgrl
Hypervirulent	-2.077116585	5.76032E-08	EF-hand domain-containing protein D2	Efhd2
Hypervirulent	-2.067289925	4.47779E-08	Calpain-2 catalytic subunit	Capn2
Hypervirulent	-2.066857529	0.000418886	Glutathione S-transferase theta-1	Gstt1
Hypervirulent	-2.063576698	4.32366E-06	14-3-3 protein beta/alpha;14-3-3 protein beta/alpha, N-terminally processed;14-3-3 protein beta/alpha	Ywhab
Hypervirulent	-2.05895977	6.38537E-07	Bifunctional coenzyme A synthase;Phosphopantetheine adenylyltransferase;Dephospho-CoA kinase	Coasy
Hypervirulent	-2.050465012	2.06689E-05	Adenylosuccinate synthetase isozyme 2	Adss2
Hypervirulent	-2.050453186	7.36564E-11	Microtubule-associated protein 4	Map4
Hypervirulent	-2.049821472	7.18925E-07	Calcyclin-binding protein	Cacybp
Hypervirulent	-2.042282867	0.000968618	G2/M phase-specific E3 ubiquitin-protein ligase	G2e3
Hypervirulent	-2.040693283	0.008168421	FHF complex subunit HOOK-interacting protein 2B	Fhip2b
Hypervirulent	-2.040361977	5.43985E-08	Glutathione reductase, mitochondrial	Gsr
Hypervirulent	-2.039046288	3.59883E-11	Complement C3;Complement C3 beta chain;C3-beta-c;Complement C3 alpha chain;C3a anaphylatoxin;Acylation stimulating protein;Complement C3b alpha chain;Complement C3c alpha chain fragment 1;Complement C3dg fragment;Complement C3g fragment;Complement C3d fragment;Complement C3f fragment;Complement C3c alpha chain fragment 2	C3
Hypervirulent	-2.038264656	2.18523E-06	Copper transport protein ATOX1	Atox1
Hypervirulent	-2.030361176	1.29225E-06	Dihydropyrimidinase-related protein 3	Dpysl3
Hypervirulent	-2.029750443	8.68559E-08	Chloride intracellular channel protein 3	Clic3
Hypervirulent	-2.029565048	7.6901E-05	Proteasome activator complex subunit 1	Psme1
Hypervirulent	-2.028275871	3.16614E-06	Ig gamma-3 chain C region	Ighg3
Hypervirulent	-2.025379562	8.80162E-08	Pleckstrin homology domain-containing family O member 2	Plekho2
Hypervirulent	-2.021976471	4.52095E-09	Gasdermin-D;Gasdermin-D, N-terminal;Gasdermin-D, C-terminal;Gasdermin-D, p13;Gasdermin-D, p40	Gsdmd
Hypervirulent	-2.020064163	1.58448E-06	NEDD8-conjugating enzyme Ubc12;E2 ubiquitin-conjugating enzyme	Ube2m
Hypervirulent	-2.013797569	8.40918E-05	Thioredoxin domain-containing protein 17	Txndc17
Hypervirulent	-2.013343811	0.016342002	Family with sequence similarity 186, member B	Fam186b
Hypervirulent	-2.012070274	1.04008E-05	Apolipoprotein A-II;Proapolipoprotein A-II	Apoa2
Hypervirulent	-2.011884117	7.06224E-05	Phosphoacetylglucosamine mutase	Pgm3
Hypervirulent	-2.010093498	1.58566E-05	Contactin-1	Cntn1
Hypervirulent	-2.010074615	6.68955E-09	Sorting nexin-5	Snx5
Hypervirulent	-2.008543015	3.63356E-08	Large ribosomal subunit protein eL42	Rpl36a;Gm6525
Hypervirulent	-2.007526779	7.73978E-07	Guanylate-binding protein 2	Gbp2
Hypervirulent	-2.005712318	1.59871E-07	Calponin-2;Calponin	Cnn2
Hypervirulent	-2.005141449	1.51089E-05	Glutathione S-transferase A4	Gsta4
Hypervirulent	-2.003973007	2.71743E-10	Glycerol-3-phosphate dehydrogenase 1-like protein	Gpd1l
Hypervirulent	-2.003005219	1.93238E-09	Small ribosomal subunit protein uS11	Rps14
Hypervirulent	-2.002647781	3.28576E-07	Transcription factor p65	Rela
Hypervirulent	-2.000606155	1.82486E-09	Poly(rC)-binding protein 1	Pcbp1
Hypervirulent	-1.998386765	0.000337204	Eosinophil cationic protein 2	Ear2
Hypervirulent	-1.996870804	1.22539E-06	Rho GTPase-activating protein 1	Arhgap1
Hypervirulent	-1.996035767	4.19976E-06	SH3 domain-binding glutamic acid-rich-like protein 3	Sh3bgrl3
Hypervirulent	-1.99464798	1.45798E-05	Transgelin	Tagln
Hypervirulent	-1.992470551	0.004799922	Protein S100;Protein S100-A1	S100a1
Hypervirulent	-1.98788147	1.69381E-05	Phenylalanine--tRNA ligase beta subunit	Farsb
Hypervirulent	-1.981637573	6.5263E-07	Mannose-1-phosphate guanyltransferase beta	Gmppb
Hypervirulent	-1.975515747	5.17655E-08	Golgi reassembly-stacking protein 2	Gorasp2
Hypervirulent	-1.974646378	1.85429E-06	Muscleblind-like protein 1;Muscleblind-like protein 2	Mbnl1;Mbnl2
Hypervirulent	-1.973314285	7.42765E-08	14-3-3 protein epsilon	Ywhae
Hypervirulent	-1.970716667	2.05268E-07	Acetyl-CoA acetyltransferase, cytosolic;acetyl-CoA C-acetyltransferase	Acat2;Acat3
Hypervirulent	-1.968168831	0.000572737	cAMP-dependent protein kinase catalytic subunit alpha	Prkaca
Hypervirulent	-1.967821693	4.83868E-08	Far upstream element-binding protein 1	Fubp1
Hypervirulent	-1.965704727	1.3266E-06	Drebrin-like protein	Dbnl

Hypervirulent	-1.962889481	0.000836453	Aldehyde dehydrogenase 1A1	Aldh1a1
Hypervirulent	-1.95594101	3.19145E-07	C-X-C motif chemokine 15	Cxcl15
Hypervirulent	-1.955628395	2.00832E-08	Serine/threonine-protein kinase PAK 2;PAK-2p27;PAK-2p34	Pak2
Hypervirulent	-1.955509949	4.62142E-05	Developmentally-regulated GTP-binding protein 1	Drg1
Hypervirulent	-1.949375343	1.63592E-05	Adenine phosphoribosyltransferase	Aprt
Hypervirulent	-1.948546028	1.96195E-10	V-type proton ATPase catalytic subunit A	Atp6v1a
Hypervirulent	-1.944286346	1.46658E-10	Adenylyl cyclase-associated protein 1	Cap1
Hypervirulent	-1.943544388	1.11711E-11	Protein Niban 2	Niban2
Hypervirulent	-1.938132095	0.034662037	Cysteine protease ATG4A;Cysteine protease	Atg4a;Atg4a-ps
Hypervirulent	-1.936478996	0.000503934	Apoptosis regulator BAX	Bax
Hypervirulent	-1.929376984	1.97094E-10	Protein transport protein Sec31A	Sec31a
Hypervirulent	-1.924955559	2.01328E-06	Hsc70-interacting protein	St13
Hypervirulent	-1.920397758	4.28026E-09	Calcium-binding protein 39	Cab39
Hypervirulent	-1.919826317	5.10551E-05	Sorting nexin-3	Snx3
Hypervirulent	-1.91944313	1.69681E-05	Dual specificity mitogen-activated protein kinase kinase 1	Map2k1
Hypervirulent	-1.918437958	4.89746E-07	Ubiquitin-fold modifier 1	Ufm1
Hypervirulent	-1.913598061	6.82464E-06	Aminopeptidase B	Rnpep
Hypervirulent	-1.910719681	0.000230029	Golgi to ER traffic protein 4 homolog	Get4
Hypervirulent	-1.910658836	0.005517317	Cytoplasmic dynein 1 heavy chain 1	Dync1h1
Hypervirulent	-1.907803726	1.59955E-08	Ubiquitin-conjugating enzyme E2 N;E2 ubiquitin-conjugating enzyme	Ube2n
Hypervirulent	-1.90644207	1.97593E-08	Y-box-binding protein 1	Ybx1
Hypervirulent	-1.905116081	7.84872E-08	14-3-3 protein zeta/delta	Ywhaz
Hypervirulent	-1.9038517	3.13629E-05	Rho GDP-dissociation inhibitor 1	Arhgdia
Hypervirulent	-1.902561188	9.04856E-06	Prostaglandin reductase 1	Ptgr1
Hypervirulent	-1.899101067	2.37826E-08	UMP-CMP kinase	Cmpk1
Hypervirulent	-1.895471954	8.3078E-05	glutathione transferase	Gm20441
Hypervirulent	-1.895306587	1.6457E-07	Heat shock protein beta-1	Hspb1
Hypervirulent	-1.894181061	7.76709E-08	Serine/threonine-protein kinase N1	Pkn1
Hypervirulent	-1.89289341	5.30501E-06	Protein phosphatase 1G	Ppm1g
Hypervirulent	-1.891625023	3.82055E-08	Cytosol aminopeptidase	Lap3
Hypervirulent	-1.891219902	0.001532511	Ubiquitin-like FUBI-ribosomal protein eS30 fusion protein;Ubiquitin-like protein FUBI;Small ribosomal subunit protein eS30;40S ribosomal protein S30	Fau
Hypervirulent	-1.889062309	6.42802E-08	Dipeptidyl peptidase 3	Dpp3
Hypervirulent	-1.887402725	2.92891E-10	Transcription elongation factor A protein 1;Transcription elongation factor	Tcea1
Hypervirulent	-1.886837959	4.22893E-07	Rab GDP dissociation inhibitor alpha	Gdi1
Hypervirulent	-1.885931587	5.20588E-05	Glia maturation factor beta	Gmfb
Hypervirulent	-1.883962631	3.32739E-08	Xaa-Pro aminopeptidase 1	Xpnpep1
Hypervirulent	-1.883553123	8.75244E-07	Glycine--tRNA ligase	Gars1
Hypervirulent	-1.882305908	1.36871E-05	High mobility group protein B3	Hmgb3
Hypervirulent	-1.881617928	8.67046E-06	Tubulin beta-2A chain;Tubulin beta-2B chain	Tubb2a;Tubb2b
Hypervirulent	-1.880287743	3.47647E-08	Sorting nexin-2	Snx2
Hypervirulent	-1.880179024	8.07699E-05	1-acylglycerol-3-phosphate O-acyltransferase ABHD5	Abhd5
Hypervirulent	-1.874839211	2.66275E-09	Inter alpha-trypsin inhibitor, heavy chain 4	Itih4
Hypervirulent	-1.874133492	4.66725E-05	Ig kappa chain V-VI region NQ2-6.1	Igkv4-55
Hypervirulent	-1.87341156	0.015403977	Eosinophil-associated, ribonuclease A family, member 10	Ear10
Hypervirulent	-1.872127724	0.000236841	Alpha-1-antitrypsin 1-1;Alpha-1-antitrypsin 1-3	Serpina1a;Serpina1c
Hypervirulent	-1.870483589	0.001784906	Zinc finger X-linked protein ZXDB	Zxdb
Hypervirulent	-1.86937542	2.8354E-07	ATP-dependent 6-phosphofructokinase, platelet type;ATP-dependent 6-phosphofructokinase	Pfkp
Hypervirulent	-1.866414833	0.000112256	U6 snRNA-associated Sm-like protein LSm1	Lsm1
Hypervirulent	-1.865560532	2.98386E-05	Pantothenate kinase 3	Pank3
Hypervirulent	-1.864718628	0.000157888	Protein S100-A4	S100a4
Hypervirulent	-1.859545898	0.03773302	Helicase ARIP4	Rad54l2
Hypervirulent	-1.85881691	0.000283503	High affinity immunoglobulin epsilon receptor subunit gamma	Fcer1g
Hypervirulent	-1.858794403	6.02821E-05	Sorting nexin-12	Snx12
Hypervirulent	-1.853549576	2.61906E-07	Far upstream element-binding protein 2	Khsrp
Hypervirulent	-1.85069046	6.31169E-09	Inositol-3-phosphate synthase 1	Isyna1
Hypervirulent	-1.850686455	9.34085E-09	Alpha-enolase;phosphopyruvate hydratase	Eno1

Hypervirulent	-1.849114609	2.03894E-09	Protein diaphanous homolog 1	Diaph1
Hypervirulent	-1.846201706	0.005079187	Dynamin-2;dynamin GTPase	Dnm2
Hypervirulent	-1.844924355	4.51792E-07	Polymeric immunoglobulin receptor;Secretory component	Pigr
Hypervirulent	-1.842885208	1.78214E-06	Proteasome subunit beta type-8;Proteasome subunit beta	Psmb8
Hypervirulent	-1.841520119	0.000473158	Tubulin beta-6 chain	Tubb6
Hypervirulent	-1.841071892	4.13947E-08	Coatomer subunit beta	Copb2
Hypervirulent	-1.839700127	3.48232E-05	Tubulin alpha-1A chain;Detyrosinated tubulin alpha-1A chain	Tuba1a
Hypervirulent	-1.837384415	4.42037E-09	Alanine--tRNA ligase, cytoplasmic	Aars1
Hypervirulent	-1.836145401	1.38456E-10	Zyxin	Zyx
Hypervirulent	-1.834711647	0.007379152	V-type proton ATPase subunit G 1	Atp6v1g1
Hypervirulent	-1.831874657	0.00265372	Cell division cycle-associated protein 2	Cdca2
Hypervirulent	-1.830816078	1.32299E-07	Chitinase-like protein 3	Chil3
Hypervirulent	-1.827245522	1.16904E-06	Formin-binding protein 1	Fnbp1
Hypervirulent	-1.826413155	0.001516635	Immunoglobulin kappa chain variable 6-17	Igkv6-17
Hypervirulent	-1.823453331	1.31581E-05	Arfaptin-1	Arfip1;Gm37240
Hypervirulent	-1.822540474	9.64572E-09	Ubiquitin-conjugating enzyme E2 H;E2 ubiquitin-conjugating enzyme	Ube2h
Hypervirulent	-1.820501709	8.64998E-05	Tubulin beta-5 chain	Tubb5
Hypervirulent	-1.820483398	4.43642E-05	Probable aminopeptidase NPEPL1	Npepl1
Hypervirulent	-1.820047569	1.78652E-06	KH domain-containing RNA-binding protein QKI	Qki
Hypervirulent	-1.818752289	0.002741093	Glutamate-rich 6B	Erich6b
Hypervirulent	-1.818303108	1.60952E-10	Coatomer subunit beta	Copb1
Hypervirulent	-1.81718235	0.00358815	Ankyrin repeat domain-containing protein 63	Ankrd63
Hypervirulent	-1.81640625	8.32484E-06	Elongation factor 1-delta	Eef1d
Hypervirulent	-1.815973282	2.10426E-05	Ubiquitin-like-conjugating enzyme ATG3	Atg3
Hypervirulent	-1.8157341	4.64489E-06	Ankyrin-3	Ank3
Hypervirulent	-1.811594963	1.40898E-09	Elongation factor 1-beta	Eef1b;Eef1b2
Hypervirulent	-1.808110619	9.07723E-08	Rab GDP dissociation inhibitor beta;Rab GDP dissociation inhibitor	Gdi2
Hypervirulent	-1.804648781	0.001023292	Putative hydroxypyruvate isomerase	Hyi
Hypervirulent	-1.801306915	1.41942E-10	Glyoxalase domain-containing protein 4	Glod4
Hypervirulent	-1.800620079	0.01787571	Sterile alpha motif domain-containing protein 15	Samd15
Hypervirulent	-1.797462845	0.002295861	Eosinophil peroxidase;Eosinophil peroxidase light chain;Eosinophil peroxidase heavy chain	Epx
Hypervirulent	-1.79516449	3.13574E-05	Eukaryotic translation initiation factor 1A, X-chromosomal;Eukaryotic translation initiation factor 1A;Eukaryotic translation initiation factor 4C	Eif1ax;Eif1a;Eif1ad15
Hypervirulent	-1.794808197	0.023357587	Nuclear transport factor 2	Nutf2
Hypervirulent	-1.794578362	1.73535E-07	Histidine--tRNA ligase, cytoplasmic	Hars1
Hypervirulent	-1.794009399	3.6154E-08	Anamorsin	Ciapin1
Hypervirulent	-1.790837479	4.02105E-09	Actin-related protein 3	Actr3
Hypervirulent	-1.79056015	2.29565E-05	S-adenosylmethionine synthase isoform type-2;S-adenosylmethionine synthase	Mat2a
Hypervirulent	-1.786141777	0.0001117	Tetratricopeptide repeat protein 27	Ttc27
Hypervirulent	-1.783600426	3.56552E-07	Interferon-gamma-inducible GTPase Ifggc1 protein	Ifi47
Hypervirulent	-1.781715965	0.016915472	CXXC-type zinc finger protein 1	Cxxc1
Hypervirulent	-1.778351974	1.88327E-07	Serine protease inhibitor A3M	Serpina3m
Hypervirulent	-1.777487946	0.000627747	F-box only protein 50	Nccrp1
Hypervirulent	-1.775033188	2.90046E-06	Small ribosomal subunit protein uS17	Rps11
Hypervirulent	-1.774650192	0.003456237	Phospholipid phosphatase 1	Plpp1
Hypervirulent	-1.77413559	7.81847E-08	Protein RCC2	Rcc2
Hypervirulent	-1.772302818	2.272E-06	3(2),5-bisphosphate nucleotidase 1	Bpnt1
Hypervirulent	-1.770628548	3.13612E-07	Large ribosomal subunit protein eL31	Rpl31
Hypervirulent	-1.770201874	0.000149423	H-2 class I histocompatibility antigen, Q10 alpha chain	H2-Q10
Hypervirulent	-1.768909645	1.36185E-06	Apoptosis-associated speck-like protein containing a CARD	Pycard
Hypervirulent	-1.768681908	0.001146984	Coatomer subunit zeta;Coatomer subunit zeta-1	Copz1
Hypervirulent	-1.767639351	7.17716E-09	Annexin A1;Annexin Ac2-26;Annexin	Anxa1
Hypervirulent	-1.766510201	5.56417E-09	Synaptobrevin homolog YKT6	Ykt6
Hypervirulent	-1.766441917	5.04148E-05	Vacuolar protein sorting-associated protein 29	Vps29
Hypervirulent	-1.762523651	3.07222E-08	Glycylpeptide N-tetradecanoyltransferase 1	Nmt1
Hypervirulent	-1.761763763	0.000109263	Ribonuclease H2 subunit A;Ribonuclease	Rnaseh2a
Hypervirulent	-1.761343384	1.95461E-05	Complement C1q subcomponent subunit B	C1qb
Hypervirulent	-1.760538292	4.43711E-05	Large ribosomal subunit protein uL29	Rpl35

Hypervirulent	-1.756759071	1.34531E-05	Stathmin	Stmn1
Hypervirulent	-1.75477829	1.44904E-09	Coatomer subunit alpha;Xenin;Proxenin;Coatomer subunit alpha	Copa
Hypervirulent	-1.753346252	2.01173E-06	Inter-alpha-trypsin inhibitor heavy chain H2	Itih2
Hypervirulent	-1.751105881	5.41271E-05	Polyunsaturated fatty acid lipoxygenase ALOX15	Alox15
Hypervirulent	-1.749977875	0.000167422	BLOC-2 complex member HPS3;Hermansky-Pudlak syndrome 3 protein homolog	Hps3
Hypervirulent	-1.749961281	2.79378E-09	Inosine-5-monophosphate dehydrogenase 2;IMP dehydrogenase	Impdh2
Hypervirulent	-1.745967674	4.46663E-07	Stathmin-2	Stmn2
Hypervirulent	-1.744899368	3.11398E-07	Small glutamine-rich tetratricopeptide repeat-containing protein alpha	Sgta
Hypervirulent	-1.744169807	0.001975991	Tetratricopeptide repeat protein 1	Ttc1
Hypervirulent	-1.744059563	1.15197E-08	Sialic acid synthase	Nans
Hypervirulent	-1.742793846	0.003477666	Zinc finger protein 569	Znf569
Hypervirulent	-1.741800499	0.000696579	Platelet-activating factor acetylhydrolase IB subunit alpha2;1-alkyl-2-acetylglycerophosphocholine esterase	Pafah1b2
Hypervirulent	-1.736714935	0.000626706	Transmembrane glycoprotein NMB	Gpnmb
Hypervirulent	-1.736453438	2.56637E-05	Small ribosomal subunit protein eS26	Rps26
Hypervirulent	-1.735427666	1.84451E-06	14-3-3 protein eta	Ywhah
Hypervirulent	-1.734123802	2.62719E-05	Signal recognition particle subunit SRP68	Srp68
Hypervirulent	-1.732787895	0.000348971	Spermidine synthase	Srm
Hypervirulent	-1.722443771	7.22284E-05	Prostaglandin E synthase 3	Ptges3
Hypervirulent	-1.717455864	6.08043E-07	Elongation factor 1-gamma	Eef1g
Hypervirulent	-1.714667892	0.000452	2-aminoethanethiol dioxygenase	Ado
Hypervirulent	-1.711340714	5.2899E-05	Acyl-protein thioesterase 1	Lypla1
Hypervirulent	-1.710956383	2.7381E-08	Apolipoprotein A-IV	Apoa4
Hypervirulent	-1.710612679	7.08326E-05	Prefoldin subunit 5	Pfdn5
Hypervirulent	-1.709058571	1.21251E-06	Proteasome activator complex subunit 2	Psme2
Hypervirulent	-1.708820534	4.01777E-07	Prostaglandin reductase 2	Ptgr2
Hypervirulent	-1.707509995	0.000225508	P2R1A-PPP2R2A-interacting phosphatase regulator 1	Pabir1
Hypervirulent	-1.705912399	8.22252E-06	14 kDa phosphohistidine phosphatase	Phpt1
Hypervirulent	-1.704501724	0.015870537	Creatine kinase M-type	Ckm
Hypervirulent	-1.698164749	9.06054E-06	BRISC and BRCA1-A complex member 2	Babam2
Hypervirulent	-1.697265816	0.00011063	Serine protease inhibitor A3K	Serpina3k
Hypervirulent	-1.696666908	1.02416E-05	Large ribosomal subunit protein uL14m	Mrpl14
Hypervirulent	-1.695896339	1.4739E-07	Serine/threonine-protein phosphatase 2A 55 kDa regulatory subunit B alpha isoform;Serine/threonine-protein phosphatase 2A 55 kDa regulatory subunit B	Ppp2r2a
Hypervirulent	-1.688111496	9.79017E-07	Protein mono-ADP-ribosyltransferase PARP3;Poly [ADP-ribose] polymerase	Parp3
Hypervirulent	-1.683051872	0.010187811	glycogenin glucosyltransferase;Glycogenin-1	Gyg1
Hypervirulent	-1.682903862	0.000287842	Immunoglobulin kappa variable 4	Igkv4-57-1
Hypervirulent	-1.682785988	8.41244E-06	Ubiquitin-like modifier-activating enzyme 5	Uba5
Hypervirulent	-1.681528282	1.26671E-06	Integrin alpha-M	Gm49368;Itgam
Hypervirulent	-1.681438446	0.00034576	N-acylglucosamine 2-epimerase	Renbp
Hypervirulent	-1.680675125	8.25273E-07	Coatomer subunit gamma-2	Copg2
Hypervirulent	-1.679990005	0.003093987	Dystonin	Dst
Hypervirulent	-1.679812431	0.000119304	Peptidyl-prolyl cis-trans isomerase A;Peptidyl-prolyl cis-trans isomerase A, N-terminally processed;Peptidyl-prolyl cis-trans isomerase	Ppia
Hypervirulent	-1.676545143	5.21193E-09	Small ribosomal subunit protein uS5	Rps2
Hypervirulent	-1.675877762	7.96801E-07	Complement factor H	Cfh
Hypervirulent	-1.675039101	0.000124392	Acyl-CoA-binding protein	Dbi
Hypervirulent	-1.667704964	1.91531E-06	Large ribosomal subunit protein uL14	Rpl23
Hypervirulent	-1.665795898	0.003615859	NEDD8	Nedd8
Hypervirulent	-1.66575489	1.51495E-08	Glucose-6-phosphate 1-dehydrogenase X;Glucose-6-phosphate 1-dehydrogenase	G6pdx
Hypervirulent	-1.665501595	2.51665E-09	Switch-associated protein 70	Swap70
Hypervirulent	-1.664705086	0.003853356	Tyrosine-protein kinase;Tyrosine-protein kinase ABL2	Abl2
Hypervirulent	-1.663058662	1.88605E-10	Exportin-2	Cse1l
Hypervirulent	-1.662264252	5.29539E-09	Leucine-rich repeat-containing protein 47	Lrrc47
Hypervirulent	-1.661065483	0.005295102	Zinc finger and BTB domain-containing protein 18	Zbtb18
Hypervirulent	-1.659902382	0.001184855	Small ribosomal subunit protein eS24;40S ribosomal protein S24	Rps24
Hypervirulent	-1.656365585	0.049345892	Glutathione S-transferase theta-3;glutathione transferase	Gstt3

Hypervirulent	-1.655947876	0.022157711	3-alpha-hydroxysteroid dehydrogenase type 1	Akr1c14
Hypervirulent	-1.655640411	2.89153E-06	Aldehyde dehydrogenase family 16 member A1	Aldh16a1
Hypervirulent	-1.6552248	5.57588E-06	Ubiquitin-conjugating enzyme E2 variant 1	Gm20431;Ube2v1
Hypervirulent	-1.655212784	3.36761E-07	Protein SGT1 homolog	Sugt1
Hypervirulent	-1.652908134	0.021493521	Charged multivesicular body protein 5	Chmp5
Hypervirulent	-1.652379227	0.000165964	Vacuolar protein sorting-associated protein 4A	Vps4a
Hypervirulent	-1.652326393	6.02188E-05	Alpha-centractin	Actr1a
Hypervirulent	-1.651712418	0.125324915	tRNA N(3)-methylcytidine methyltransferase METTL2	Mettl2
Hypervirulent	-1.649371147	1.01478E-10	Twinfilin-2	Twf2
Hypervirulent	-1.648501396	1.35391E-07	WD repeat-containing protein 1	Wdr1
Hypervirulent	-1.646271706	0.000117963	CD177 antigen	Cd177
Hypervirulent	-1.645134735	0.000312291	Cystathionine gamma-lyase	Cth
Hypervirulent	-1.644436836	4.89113E-06	Adenosine kinase	Adk
Hypervirulent	-1.633826256	1.94165E-07	Eukaryotic translation initiation factor 2A;Eukaryotic translation initiation factor 2A, N-terminally processed	Eif2a
Hypervirulent	-1.63330822	1.39282E-06	Phosphatidylinositol transfer protein alpha isoform	Pitpna
Hypervirulent	-1.632469749	9.14182E-06	Ras-related GTP-binding protein C;Ras-related GTP-binding protein D;Ras-related GTP-binding protein	Rragc;Rragd
Hypervirulent	-1.631362343	2.28432E-07	Sorting nexin-1	Snx1
Hypervirulent	-1.629819298	6.27502E-05	GMP synthase [glutamine-hydrolyzing]	Gmps
Hypervirulent	-1.628288269	9.55477E-06	Cytoplasmic dynein 1 light intermediate chain 1	Dync1li1
Hypervirulent	-1.628108025	5.17967E-07	eIF5-mimic protein 2	Bzw1
Hypervirulent	-1.628013039	1.5988E-10	Sec24-related gene family, member C	Sec24c
Hypervirulent	-1.626122856	0.090172856	Trimeric intracellular cation channel type A	Tmem38a
Hypervirulent	-1.623368835	3.59417E-06	Aspartyl aminopeptidase	Dnpep
Hypervirulent	-1.622858047	3.77505E-06	Large ribosomal subunit protein uL24	Rpl26
Hypervirulent	-1.622622299	0.02591305	Fucose mutarotase;L-fucose mutarotase	Fuom
Hypervirulent	-1.619812012	1.07402E-06	Arf-GAP with coiled-coil, ANK repeat and PH domain-containing protein 2;Arf-GAP with coiled-coil, ANK repeat and PH domain-containing protein	Acap2
Hypervirulent	-1.617747116	1.46787E-05	Sorbitol dehydrogenase	Sord
Hypervirulent	-1.616664314	3.46396E-05	ADP-ribosylation factor-binding protein GGA1	Gga1
Hypervirulent	-1.616118431	1.09844E-05	Bromodomain-containing protein 9	Brd9
Hypervirulent	-1.613222313	3.24739E-06	COMM domain-containing protein 3	Commd3
Hypervirulent	-1.613144112	0.000939349	U8 snoRNA-decapping enzyme	Nudt16
Hypervirulent	-1.612368202	2.1053E-05	Sepiapterin reductase	Spr
Hypervirulent	-1.611548805	0.002366758	Vacuolar protein sorting-associated protein 26C	Vps26c
Hypervirulent	-1.610552597	3.66916E-07	Argininosuccinate lyase	Asl
Hypervirulent	-1.609738731	0.021988472	Protein ERGIC-53-like	Lman1l
Hypervirulent	-1.609526253	7.79405E-06	Protein phosphatase 1F	Ppm1f
Hypervirulent	-1.609280586	5.54934E-10	Plastin-3	Pls3
Hypervirulent	-1.603812218	5.22495E-06	Programmed cell death 1 ligand 2	Pdcd1lg2
Hypervirulent	-1.602453423	1.03067E-06	Ras-related GTP-binding protein B;Ras-related GTP-binding protein A	Rragb;Rraga
Hypervirulent	-1.601406097	0.000391761	Brain acid soluble protein 1	Basp1
Hypervirulent	-1.600808525	0.053154987	Ankyrin repeat domain-containing protein 53	Ankrd53
Hypervirulent	-1.59864521	9.13567E-06	Glutamine--tRNA ligase;glutamine--tRNA ligase	Qars1
Hypervirulent	-1.598471832	1.24851E-07	Peroxiredoxin-1	Prdx1
Hypervirulent	-1.598412895	7.04388E-05	Signal recognition particle 14 kDa protein	Srp14
Hypervirulent	-1.59780426	0.022205226	Tetratricopeptide repeat protein 22	Ttc22
Hypervirulent	-1.594704819	4.96327E-05	CYFIP-related Rac1 interactor B	Cyrib
Hypervirulent	-1.593945503	0.002168126	Transcriptional regulator protein Pur-beta	Purb
Hypervirulent	-1.59387188	8.44112E-07	Carbonyl reductase [NADPH] 1	Cbr1
Hypervirulent	-1.593539238	7.73997E-05	Small ribosomal subunit protein uS12	Rps23
Hypervirulent	-1.592299271	8.12286E-08	V-type proton ATPase subunit C 1	Atp6v1c1
Hypervirulent	-1.591929626	0.000233987	Platelet-activating factor acetylhydrolase IB subunit alpha1	Pafah1b3
Hypervirulent	-1.587642479	0.000981315	Interferon-induced 35 kDa protein homolog	Ifi35
Hypervirulent	-1.583846855	3.7024E-06	Inositol-1-monophosphatase;Inositol monophosphatase 1	Impa1
Hypervirulent	-1.583423424	0.003621633	ADP-ribosylation factor-like protein 11	Arl11
Hypervirulent	-1.581873322	1.11856E-05	Beta-centractin	Actr1b
Hypervirulent	-1.581175423	0.000160135	Replication protein A 32 kDa subunit	Rpa2

Hypervirulent	-1.580853653	0.007225252	Acidic leucine-rich nuclear phosphoprotein 32 family member;Acidic leucine-rich nuclear phosphoprotein 32 family member E	Anp32e
Hypervirulent	-1.580472374	3.21898E-06	RNA-binding protein with multiple splicing	Rbpms
Hypervirulent	-1.580146217	5.3528E-08	Mitogen-activated protein kinase 3;Mitogen-activated protein kinase;mitogen-activated protein kinase	Mapk3
Hypervirulent	-1.580120468	0.003039469	Protein phosphatase 1 regulatory subunit 14A;Protein phosphatase 1 regulatory subunit 14	Ppp1r14a
Hypervirulent	-1.579791832	4.83116E-08	Large ribosomal subunit protein uL16;Large ribosomal subunit protein uL16-like	Rpl10;Rpl10l
Hypervirulent	-1.57916851	0.000115795	UV excision repair protein RAD23 homolog B	Rad23b
Hypervirulent	-1.578396606	0.005481257	Transient receptor potential cation channel subfamily V member 4	Trpv4
Hypervirulent	-1.576104355	0.00014472	Na(+)/H(+) exchange regulatory cofactor NHE-RF1	Nherf1
Hypervirulent	-1.571762848	0.006353741	Hyaluronan-binding protein 2;Hyaluronan-binding protein 2 50 kDa heavy chain;Hyaluronan-binding protein 2 50 kDa heavy chain alternate form;Hyaluronan-binding protein 2 27 kDa light chain;Hyaluronan-binding protein 2 27 kDa light chain alternate form	Habp2
Hypervirulent	-1.570274925	0.005009588	Putative per-hexamer repeat protein 5	Phxr5
Hypervirulent	-1.56853981	0.000129396	Large ribosomal subunit protein uL22	Rpl17
Hypervirulent	-1.567266273	2.34489E-05	Periodic tryptophan protein 1 homolog	Pwp1
Hypervirulent	-1.564425278	1.5705E-05	Calcium/calmodulin-dependent protein kinase type 1D	Camk1d
Hypervirulent	-1.561118889	8.36163E-07	Ubiquitin-like modifier-activating enzyme 1	Uba1
Hypervirulent	-1.560982132	0.001024232	Acyl-protein thioesterase 2	Lypla2
Hypervirulent	-1.560704231	0.001463418	H-2 class II histocompatibility antigen, E-K alpha chain;H-2 class II histocompatibility antigen, E-D alpha chain	H2-Ea
Hypervirulent	-1.560366631	2.34729E-09	ferroxidase;Ceruloplasmin	Cp
Hypervirulent	-1.559076691	1.0746E-06	Protein N-terminal asparagine amidohydrolase	Ntan1
Hypervirulent	-1.55841732	0.048297394	U8 snoRNA-decapping enzyme	Nudt16l2
Hypervirulent	-1.558057976	0.00225955	V-type proton ATPase subunit F	Atp6v1f
Hypervirulent	-1.557932091	4.47985E-11	Eukaryotic peptide chain release factor subunit 1	Etf1
Hypervirulent	-1.557667732	4.03678E-07	SUMO-conjugating enzyme UBC9	Ube2i
Hypervirulent	-1.557451439	0.001778279	Stromal membrane-associated protein 1	Smap1
Hypervirulent	-1.556887245	7.28766E-08	Methionine aminopeptidase 2	Metap2
Hypervirulent	-1.556502342	0.001014237	Cytokine receptor-like factor 3	Crlf3
Hypervirulent	-1.555546379	0.000173705	Proteasome subunit beta type-4	Psmb4
Hypervirulent	-1.555049133	3.41418E-05	Calcium-activated chloride channel regulator 1	Clca1
Hypervirulent	-1.550905037	1.57826E-05	Vacuolar protein sorting-associated protein 26B	Vps26b
Hypervirulent	-1.546048927	7.62245E-05	Cyclin-dependent kinase 2	Cdk2
Hypervirulent	-1.544461632	1.30644E-07	Coronin-7	Coro7
Hypervirulent	-1.542716789	0.000287084	Trk-fused protein	Tfg
Hypervirulent	-1.542539978	8.84227E-10	Kinesin-1 heavy chain;Kinesin-like protein	Kif5b
Hypervirulent	-1.539408302	0.000287937	Carbonic anhydrase-related protein	Ca8
Hypervirulent	-1.538549232	8.19761E-06	Isochorismatase domain-containing protein 1	Isoc1
Hypervirulent	-1.537892342	0.000285102	Aldehyde oxidase 3	Aox3
Hypervirulent	-1.536604881	0.000467935	Mitogen-activated protein kinase kinase kinase 14	Map3k14
Hypervirulent	-1.5346632	1.14002E-05	Coatomer subunit epsilon	Cope
Hypervirulent	-1.534544182	0.001550108	Cathepsin G	Ctsg
Hypervirulent	-1.534167862	0.000313566	Peptidyl-prolyl cis-trans isomerase FKBP5;peptidylprolyl isomerase	Fkbp5
Hypervirulent	-1.531420898	1.08373E-07	Ribosyldihydronicotinamide dehydrogenase [quinone]	Nqo2
Hypervirulent	-1.529322243	1.73563E-05	Annexin A5	Anxa5
Hypervirulent	-1.526710892	4.47505E-05	Transcriptional activator protein Pur-alpha	Pura
Hypervirulent	-1.523904991	2.59219E-05	S-methyl-5-thioadenosine phosphorylase	Mtap
Hypervirulent	-1.522684669	0.000158775	Large ribosomal subunit protein uL23	Rpl23a
Hypervirulent	-1.519908524	4.64397E-09	Murinoglobulin-1	Mug1
Hypervirulent	-1.516878319	6.40095E-05	ADP-ribosylation factor 5	Arf5
Hypervirulent	-1.51527195	2.32623E-10	Activator of 90 kDa heat shock protein ATPase homolog 1	Ahsa1
Hypervirulent	-1.514962196	2.5832E-06	Actin-related protein 2/3 complex subunit 3	Arpc3
Hypervirulent	-1.514306259	2.81172E-05	Immunoglobulin kappa constant	Igkc
Hypervirulent	-1.51282444	2.74665E-05	Endophilin-B1	Sh3glb1
Hypervirulent	-1.511155701	6.86379E-05	Dual specificity mitogen-activated protein kinase kinase 3	Map2k3
Hypervirulent	-1.511117363	3.25165E-05	Chloride intracellular channel protein 4	Clic4
Hypervirulent	-1.510876465	0.000113977	Alpha-mannosidase 2C1;alpha-mannosidase	Man2c1

Hypervirulent	-1.510589409	0.002037264	U6 snRNA-associated Sm-like protein LSm7	Lsm7
Hypervirulent	-1.50791111	1.09089E-06	Ubiquitin-like modifier-activating enzyme 7	Uba7
Hypervirulent	-1.507453537	5.17999E-08	Bifunctional glutamate/proline--tRNA ligase;Glutamate--tRNA ligase;Proline--tRNA ligase	Eprs1
Hypervirulent	-1.506607246	1.60524E-07	N(G),N(G)-dimethylarginine dimethylaminohydrolase 1	Ddah1
Hypervirulent	-1.50542717	7.11166E-05	Large ribosomal subunit protein eL24	Rpl24
Hypervirulent	-1.504588509	0.00287299	Immunoglobulin kappa variable 8	Igkv8-21;Igkv8-27;Igkv8-30
Hypervirulent	-1.503792381	0.00418824	Periphilin-1	Pphln1
Hypervirulent	-1.500008965	7.2826E-05	Large ribosomal subunit protein eL14	Rpl14
Hypervirulent	-1.49812851	0.000258637	Golgi-associated PDZ and coiled-coil motif-containing protein	Gopc
Hypervirulent	-1.496495438	0.000194073	Lysosome-associated membrane glycoprotein 3	Lamp3
Hypervirulent	-1.494994545	7.37492E-07	Integrin beta-2;Integrin beta	Itgb2
Hypervirulent	-1.49436512	0.003279583	Zinc finger protein 148	Znf148;Zfp148
Hypervirulent	-1.493830872	0.025959837	Mitogen-activated protein kinase 6	Mapk6
Hypervirulent	-1.493387222	0.005922824	Oviduct-specific glycoprotein	Ovgp1
Hypervirulent	-1.493126488	7.93539E-09	Rabankyrin-5	Ankfy1
Hypervirulent	-1.492866135	1.88724E-05	Inhibitor of carbonic anhydrase	Inhca
Hypervirulent	-1.491576576	0.000593871	Glycogen phosphorylase, muscle form;Alpha-1,4 glucan phosphorylase	Pygm
Hypervirulent	-1.490828514	0.008340576	Zinc finger and BTB domain-containing 39	Zbtb39
Hypervirulent	-1.490617752	1.26089E-06	Biliverdin reductase A	Blvra
Hypervirulent	-1.490484619	0.000214125	Charged multivesicular body protein 1B2;Charged multivesicular body protein 1B1	Chmp1b2;Chmp1b1
Hypervirulent	-1.489006615	0.000432308	AP-3 complex subunit mu-1	Ap3m1
Hypervirulent	-1.486181068	0.001054954	Acidic mammalian chitinase	Chia;Chia1
Hypervirulent	-1.48406601	0.010241602	Peptidyl-prolyl cis-trans isomerase NIMA-interacting 4	Pin4
Hypervirulent	-1.483294868	0.003381733	Cysteine-rich protein 1	Crip1
Hypervirulent	-1.483265686	0.000493961	Interferon-induced protein with tetratricopeptide repeats 1	Ifit1
Hypervirulent	-1.483232498	0.00012827	Low affinity immunoglobulin gamma Fc region receptor II	Fcgr2;Fcgr2b
Hypervirulent	-1.48041172	0.000370298	Anterior gradient protein 2 homolog	Agr2
Hypervirulent	-1.479119682	5.04876E-07	Protein transport protein Sec23B	Sec23b
Hypervirulent	-1.478061867	7.2875E-06	Ras GTPase-activating-like protein IQGAP1	Iqgap1
Hypervirulent	-1.476944542	2.53716E-05	15-hydroxyprostaglandin dehydrogenase [NAD(+)]	Hpgd
Hypervirulent	-1.47693367	9.46745E-06	Serine--tRNA ligase, cytoplasmic;serine--tRNA ligase	Sars1
Hypervirulent	-1.474327087	0.090839788	Tropomyosin beta chain	Tpm2
Hypervirulent	-1.47401371	8.46102E-05	Allograft inflammatory factor 1	Aif1
Hypervirulent	-1.472034836	5.15223E-08	Protein LZIC	Lzic
Hypervirulent	-1.471487045	6.47779E-06	V-type proton ATPase subunit E 1	Atp6v1e1
Hypervirulent	-1.470981026	2.87949E-05	Small ribosomal subunit protein eS6	Rps6
Hypervirulent	-1.470492172	0.005326874	Protein phosphatase methylesterase 1	Ppme1
Hypervirulent	-1.468340492	0.04802362	Transmembrane protein 132A	Tmem132a
Hypervirulent	-1.467744446	1.64137E-08	Eukaryotic translation initiation factor 3 subunit L	Eif3l
Hypervirulent	-1.467100525	0.031346737	Ig alpha chain C region	P01878
Hypervirulent	-1.463665009	0.000875253	Serine/threonine-protein phosphatase 5;Serine/threonine-protein phosphatase	Ppp5c
Hypervirulent	-1.463321304	0.000279113	Cytochrome b-245 light chain	Cyba
Hypervirulent	-1.462973404	0.003043698	Aldehyde oxidase 2	Aox2
Hypervirulent	-1.461456108	1.4569E-05	AH receptor-interacting protein	Aip
Hypervirulent	-1.45992794	0.001044982	Neutrophil cytosol factor 4	Ncf4
Hypervirulent	-1.458081436	0.045957353	Predicted gene 45808	Gm45808
Hypervirulent	-1.456887627	3.57827E-08	Aflatoxin B1 aldehyde reductase member 2	Akr7a2
Hypervirulent	-1.456664467	1.93756E-05	Thioredoxin	Txn
Hypervirulent	-1.45644207	1.88693E-06	Vasodilator-stimulated phosphoprotein	Vasp
Hypervirulent	-1.456129265	0.028158723	Phospholipase A and acyltransferase 5	Plaat5
Hypervirulent	-1.455293655	1.51672E-05	DnaJ homolog subfamily C member 8	Dnajc8
Hypervirulent	-1.453989029	8.33403E-06	Septin;Septin-7	Septin7
Hypervirulent	-1.45392437	0.002674556	Guanylate-binding protein 6	Gbp10;Gm43302;Gbp6
Hypervirulent	-1.453635979	1.46069E-08	Proteasome subunit alpha type-4;Proteasome subunit alpha type	Psma4
Hypervirulent	-1.452091789	7.58226E-09	Small ribosomal subunit protein eS1	Rps3a
Hypervirulent	-1.450707054	3.854E-05	6-pyruvoyl tetrahydrobiopterin synthase	Pts
Hypervirulent	-1.450298119	1.66284E-06	Large ribosomal subunit protein eL28	Rpl28

Hypervirulent	-1.449073029	4.96101E-06	Pseudouridylate synthase TRUB1	Trub1
Hypervirulent	-1.448875999	0.001749629	ADP-ribosylation factor 1;ADP-ribosylation factor 3;ADP-ribosylation factor 2	Arf1;Arf3;Arf2
Hypervirulent	-1.445912933	1.40954E-08	Puromycin-sensitive aminopeptidase;Aminopeptidase	Npepps
Hypervirulent	-1.444970322	1.15604E-06	Translationally-controlled tumor protein	Tpt1
Hypervirulent	-1.444495583	0.036969323	Bone marrow proteoglycan;Eosinophil granule major basic protein	Prg2
Hypervirulent	-1.443708038	3.8177E-05	Interferon regulatory factor 3	Irf3
Hypervirulent	-1.441757011	3.21022E-05	H-2 class II histocompatibility antigen, A-K beta chain;H-2 class II histocompatibility antigen, A-F beta chain;H-2 class II histocompatibility antigen, A-U beta chain;H-2 class II histocompatibility antigen, A beta chain	H2-Ab1
Hypervirulent	-1.441205215	0.000715943	Atlastin-2	Atl2
Hypervirulent	-1.439300156	0.004736552	Glutaredoxin-1	Glrx
Hypervirulent	-1.436755753	0.00368091	Large ribosomal subunit protein uL3m	Mrpl3
Hypervirulent	-1.436354065	0.039039634	Small nuclear ribonucleoprotein E	Snrpe
Hypervirulent	-1.434139824	6.23122E-06	Glutamate--cysteine ligase catalytic subunit	Gclc
Hypervirulent	-1.433587837	2.75682E-05	Glutathione synthetase	Gss
Hypervirulent	-1.433123207	5.3162E-05	Sulfhydryl oxidase 1	Qsox1
Hypervirulent	-1.431847572	1.10814E-06	E3 ubiquitin-protein ligase RING2	Rnf2
Hypervirulent	-1.429883766	0.000379582	Interferon-induced protein 44	Ifi44
Hypervirulent	-1.429107094	0.002858892	Ras-related protein Rab-1B;small monomeric GTPase	Rab1b
Hypervirulent	-1.429008293	0.003236313	Centrosomal protein of 41 kDa	Cep41
Hypervirulent	-1.428075218	4.1644E-06	V-type proton ATPase subunit H	Atp6v1h
Hypervirulent	-1.42537365	0.00037293	Dynactin subunit 3	Dctn3
Hypervirulent	-1.424687576	5.53593E-08	Dynactin subunit 4	Dctn4
Hypervirulent	-1.423184967	7.18106E-05	Zinc finger, MYM-type 6	Zmym6
Hypervirulent	-1.422544479	7.35191E-05	Protein kinase C iota type	Prkci
Hypervirulent	-1.422423553	1.24662E-05	Cold shock domain-containing protein E1	Csde1
Hypervirulent	-1.420739365	0.014241724	Cysteine and glycine-rich protein 3	Csrp3
Hypervirulent	-1.418649101	0.04353662	ADP-ribosylation factor-like protein 1	Arl1
Hypervirulent	-1.417358208	7.4716E-08	Fc fragment of IgG-binding protein	Fcgbp
Hypervirulent	-1.417268181	0.000344951	L-fucose kinase	Fcsk
Hypervirulent	-1.414136887	0.050841195	Dual specificity protein phosphatase CDC14A	Cdc14a
Hypervirulent	-1.413093185	0.006936729	Translin-associated protein X	Tsnax
Hypervirulent	-1.413055611	2.65018E-07	Alanine--glyoxylate aminotransferase	Agxt
Hypervirulent	-1.412134933	0.018995023	Acylphosphatase-2	Acyp2
Hypervirulent	-1.41194706	1.62925E-05	Queuosine 5-phosphate N-glycosylase/hydrolase	QNG1
Hypervirulent	-1.411604691	3.01026E-07	26S proteasome non-ATPase regulatory subunit 7	Psmd7
Hypervirulent	-1.409212875	2.34525E-07	Phosphatidylinositol-glycan-specific phospholipase D	Gpld1
Hypervirulent	-1.407483292	0.000416807	Density-regulated protein	Denr
Hypervirulent	-1.40725193	0.001653276	Ribose-phosphate pyrophosphokinase 2	Prps2
Hypervirulent	-1.406894112	3.22991E-06	Myc box-dependent-interacting protein 1	Bin1
Hypervirulent	-1.406819344	5.81566E-05	Polypyrimidine tract-binding protein 3	Ptbp3
Hypervirulent	-1.406308937	0.002606478	Quinone oxidoreductase	Cryz
Hypervirulent	-1.405150032	0.006110537	Poly [ADP-ribose] polymerase tankyrase-2	Tnks2
Hypervirulent	-1.40475502	6.31557E-08	Small ribosomal subunit protein eS19	Rps19
Hypervirulent	-1.404408073	0.011207877	Haloacid dehalogenase-like hydrolase domain-containing protein 2	Hdhd2
Hypervirulent	-1.403809166	0.000168522	Actin-related protein 2/3 complex subunit 1B;Actin-related protein 2/3 complex subunit	Arpc1b
Hypervirulent	-1.400793839	4.45349E-06	Cytosolic Fe-S cluster assembly factor NUBP2	Nubp2
Hypervirulent	-1.397936058	0.091260279	Mitochondrial thiamine pyrophosphate carrier	Slc25a19
Hypervirulent	-1.395299721	0.000968217	Transcription factor BTF3 homolog 4	Btf3l4
Hypervirulent	-1.390921974	2.23763E-06	IST1 homolog	Ist1
Hypervirulent	-1.388931847	0.005172828	Fibroblast growth factor 4	Fgf4
Hypervirulent	-1.386815834	3.50548E-05	Serine/threonine-protein kinase OSR1	Oxsr1
Hypervirulent	-1.38542881	0.063751277	UBX domain-containing protein 2A	Ubxn2a
Hypervirulent	-1.38537426	3.61722E-05	NAD-dependent protein deacetylase sirtuin-2;NAD-dependent protein deacetylase	Sirt2
Hypervirulent	-1.383906555	1.21094E-06	Alpha-2-antiplasmin	Serpinf2
Hypervirulent	-1.383738518	0.003993895	Z-DNA-binding protein 1	Zbp1
Hypervirulent	-1.383428001	9.14889E-05	Charged multivesicular body protein 2a	Chmp2a
Hypervirulent	-1.383102989	1.73252E-06	Cleavage and polyadenylation specificity factor subunit 5	Nudt21

Hypervirulent	-1.381908035	0.003461535	Alpha-1-antitrypsin 1-5	Serpina1e
Hypervirulent	-1.381166649	2.74765E-07	Proliferation-associated protein 2G4	Pa2g4
Hypervirulent	-1.380916786	9.55921E-07	Phosphopentomutase	Pgm2
Hypervirulent	-1.379688835	0.00038344	Enolase-phosphatase E1	Enoph1
Hypervirulent	-1.379278183	4.48576E-06	Eukaryotic initiation factor 4A-I	Eif4a1
Hypervirulent	-1.378992462	0.000134659	Small ribosomal subunit protein eS28	Rps28
Hypervirulent	-1.378895569	1.44245E-07	Large ribosomal subunit protein eL13	Rpl13
Hypervirulent	-1.378113937	0.083569666	AT-rich interactive domain-containing protein 3;AT-rich interactive domain-containing protein 3A	Arid3a
Hypervirulent	-1.378037453	0.000101721	Ubiquitin-conjugating enzyme E2 K;E2 ubiquitin-conjugating enzyme	Ube2k
Hypervirulent	-1.377577019	1.21088E-05	Choline-phosphate cytidylyltransferase A;choline-phosphate cytidylyltransferase	Pcyt1a
Hypervirulent	-1.376774788	5.96957E-07	Heterogeneous nuclear ribonucleoprotein K	Hnrnpk
Hypervirulent	-1.375881767	2.36768E-08	Echinoderm microtubule-associated protein-like 1	Eml1
Hypervirulent	-1.375363541	0.000357542	Importin subunit alpha-4	Kpna3
Hypervirulent	-1.371894264	8.11077E-05	Pumilio homolog 1	Pum1
Hypervirulent	-1.371526527	0.026062231	RIKEN cDNA 2310061I04 gene	2310061I04Rik
Hypervirulent	-1.370368195	7.69179E-06	BTB/POZ domain-containing protein KCTD12	Kctd12
Hypervirulent	-1.368702888	6.22331E-08	Programmed cell death 6-interacting protein	Pdcd6ip
Hypervirulent	-1.365126801	1.41878E-06	Cytochrome b-245 heavy chain	Cybb
Hypervirulent	-1.365077972	9.87092E-05	Nicotinamide phosphoribosyltransferase	Nampt
Hypervirulent	-1.363003922	1.40645E-05	Small ribosomal subunit protein eS7	Rps7
Hypervirulent	-1.361022568	1.42211E-05	Proteasome subunit alpha type-3	Psma3
Hypervirulent	-1.359937668	4.50477E-05	6-phosphogluconolactonase	Pgls
Hypervirulent	-1.359108353	6.12207E-05	Tyrosine-protein kinase SYK;Tyrosine-protein kinase	Syk
Hypervirulent	-1.357917976	7.71251E-05	14-3-3 protein sigma	Sfn
Hypervirulent	-1.35627327	0.023062422	Glutathione S-transferase P 1;Glutathione S-transferase;glutathione transferase;Glutathione S-transferase P 2	Gstp1;Gstp2
Hypervirulent	-1.356136322	0.003438845	Endoribonuclease LACTB2	Lactb2
Hypervirulent	-1.355324936	0.006904071	Prolyl endopeptidase	Prep
Hypervirulent	-1.354394341	0.000192483	Immunity-related GTPase family M protein 2	Irgm2
Hypervirulent	-1.353924561	0.012101392	Slit homolog 1 protein	Slit1
Hypervirulent	-1.350927353	0.000104583	Protein transport protein Sec23A;Protein transport protein SEC23	Sec23a
Hypervirulent	-1.350036812	0.002571484	Charged multivesicular body protein 1a	Chmp1a
Hypervirulent	-1.349956703	0.010787791	Peroxiredoxin-5, mitochondrial;thioredoxin-dependent peroxiredoxin;Peroxiredoxin-5	Prdx5
Hypervirulent	-1.349831581	0.000739675	Glycolipid transfer protein	Gltp
Hypervirulent	-1.348392677	5.71371E-08	Polyadenylate-binding protein 1	Pabpc1
Hypervirulent	-1.34674263	5.09047E-06	Large ribosomal subunit protein uL10	Rplp0
Hypervirulent	-1.346682167	0.00393584	E3 ubiquitin-protein ligase MARCHF9	Marchf9
Hypervirulent	-1.344779015	0.000375738	Immunoglobulin kappa variable 1	Igkv1-135;Igkv1-133
Hypervirulent	-1.344264793	0.014570141	Rho guanine nucleotide exchange factor 28	Arhgef28
Hypervirulent	-1.344054604	3.5896E-05	Adenylosuccinate lyase	Adsl
Hypervirulent	-1.343219757	3.40712E-05	Beta-arrestin-1	Arrb1
Hypervirulent	-1.342419434	0.002265336	Ubiquitin-conjugating enzyme E2 D2;Ubiquitin-conjugating enzyme E2 D3;E2 ubiquitin-conjugating enzyme	Ube2d2;Ube2d3;Ube2d2a
Hypervirulent	-1.341118813	0.00354442	Blood group Rh(D) polypeptide	Rhd
Hypervirulent	-1.339743233	0.029288071	Mediator of RNA polymerase II transcription subunit 15	Med15
Hypervirulent	-1.338440132	0.000924187	Ig kappa chain V-V region L7	Gm10881
Hypervirulent	-1.337862015	0.012579259	COMM domain-containing protein 7	Commd7
Hypervirulent	-1.337239456	2.32661E-06	Dynactin subunit 1	Dctn1
Hypervirulent	-1.337087631	5.38651E-05	DENN domain-containing protein 11	Dennd11
Hypervirulent	-1.335998917	0.000237859	Protein Niban 1	Niban1
Hypervirulent	-1.335466385	5.34694E-07	Mitogen-activated protein kinase 1	Mapk1
Hypervirulent	-1.335213852	2.25823E-06	Large ribosomal subunit protein eL20;60S ribosomal protein L18a	Rpl18a
Hypervirulent	-1.333950043	0.000194005	Stromal cell-derived factor 1	Cxcl12
Hypervirulent	-1.333818054	0.009989898	Splicing factor 1	Sf1
Hypervirulent	-1.333506012	1.69883E-06	Plasma protease C1 inhibitor	Serping1
Hypervirulent	-1.331868935	1.98379E-05	Microtubule-associated protein RP/EB family member 1	Mapre1
Hypervirulent	-1.331522942	0.00564471	Protein NDRG2	Ndrg2

Hypervirulent	-1.329636383	5.76015E-07	Prothrombin;Activation peptide fragment 1;Activation peptide fragment 2;Thrombin light chain;Thrombin heavy chain;Prothrombin	F2
Hypervirulent	-1.329175568	0.002394478	Trans-1,2-dihydrobenzene-1,2-diol dehydrogenase	Dhdh
Hypervirulent	-1.328915024	3.73313E-06	U6 snRNA-associated Sm-like protein LSm2	Lsm2
Hypervirulent	-1.327156067	0.021355253	NCK-interacting protein with SH3 domain	Nckipsd
Hypervirulent	-1.326825142	5.77388E-06	Fibroblast growth factor 1;Multifunctional fusion protein;Fibroblast growth factor 1;Fibroblast growth factor	Fgf1
Hypervirulent	-1.324864578	0.000448131	Pulmonary surfactant-associated protein C	Sftpc
Hypervirulent	-1.324637604	3.68797E-07	26S proteasome regulatory subunit 4	Psmc1
Hypervirulent	-1.323820496	5.54985E-06	Signal transducer and activator of transcription 5B	Stat5b
Hypervirulent	-1.323169327	0.000438078	Vacuolar protein sorting-associated protein 26A	Vps26a
Hypervirulent	-1.32172718	0.005759456	E3 ubiquitin-protein ligase RNF181	Rnf181
Hypervirulent	-1.320573616	0.053302157	Interleukin-1 receptor antagonist protein;Interleukin-1	Il1rn
Hypervirulent	-1.31998291	0.000742561	Septin-8;Septin	Septin8
Hypervirulent	-1.319918823	3.13291E-05	Dynamin-1-like protein	Dnm1l
Hypervirulent	-1.319607544	0.018654259	protein-serine/threonine phosphatase;Protein phosphatase 1B	Ppm1b
Hypervirulent	-1.316970634	1.497E-05	Neutrophil cytosol factor 2	Ncf2
Hypervirulent	-1.316466331	0.009125107	Methionine adenosyltransferase 2 subunit beta	Mat2b
Hypervirulent	-1.316250801	7.25924E-07	Vigilin	Hdlbp
Hypervirulent	-1.314118385	0.000116272	Rap1 GTPase-GDP dissociation stimulator 1	Rap1gds1
Hypervirulent	-1.313638878	0.012719114	Serine/threonine-protein phosphatase 6 regulatory subunit 1	Ppp6r1
Hypervirulent	-1.31265316	0.002133252	Zinc-alpha-2-glycoprotein	Azgp1
Hypervirulent	-1.311902428	6.57996E-05	Peflin	Pef1
Hypervirulent	-1.310924339	6.1972E-06	Ribosomal protein S6 kinase alpha-3	Rps6ka3
Hypervirulent	-1.309347916	0.005289345	Proteasome subunit beta type-3	Psmb3
Hypervirulent	-1.30771656	1.56155E-06	Ketimine reductase mu-crystallin	Crym
Hypervirulent	-1.307630348	0.005364322	A disintegrin-like and metallopeptidase (reprolysin type) with thrombospondin type 1 motif, 9	Adamts9
Hypervirulent	-1.306880951	0.000656739	Protein arginine N-methyltransferase 5	Prmt5
Hypervirulent	-1.305489159	7.21911E-07	Septin-11;Septin	Septin11
Hypervirulent	-1.303142738	4.38116E-06	Antiviral innate immune response receptor RIG-I	Rigi
Hypervirulent	-1.30064621	0.011092271	Protein SEC13 homolog	Sec13
Hypervirulent	-1.300625229	0.021052864	Protein tweety homolog 3	Ttyh3
Hypervirulent	-1.300424957	7.77035E-05	BRISC complex subunit Abraxas 2	Abraxas2
Hypervirulent	-1.300260925	1.61222E-05	Prosaposin;Prosaposin;Saposin-A;Saposin-B-Val;Saposin-B;Saposin-C;Saposin-D	Psap
Hypervirulent	-1.298617363	0.014155391	PRAME family member 20	Pramel15
Hypervirulent	-1.297837448	0.016670582	Eukaryotic translation initiation factor 4H	Eif4h
Hypervirulent	-1.297426414	0.000134894	MAP kinase-activated protein kinase 2	Mapkapk2
Hypervirulent	-1.293494415	0.017002159	7-dehydrocholesterol reductase	Dhcr7
Hypervirulent	-1.293225288	5.32552E-06	Trifunctional purine biosynthetic protein adenosine-3;Phosphoribosylamine--glycine ligase;Phosphoribosylformylglycinamidine cyclo-ligase;Phosphoribosylglycinamide formyltransferase	Gart
Hypervirulent	-1.292941666	0.005371757	N-acetylgalactosamine kinase	Galk2
Hypervirulent	-1.292456818	0.000136145	Dynactin subunit 2	Dctn2
Hypervirulent	-1.292032814	1.76344E-06	26S proteasome regulatory subunit 8	Psmc5
Hypervirulent	-1.291957664	0.002675382	Olfactomedin-4	Olfm4
Hypervirulent	-1.288197708	0.039851405	Immunoglobulin kappa variable 12	Igkv12-44
Hypervirulent	-1.287770081	8.42961E-05	Ubiquitin-conjugating enzyme E2 L3;E2 ubiquitin-conjugating enzyme	Ube2l3
Hypervirulent	-1.286101532	2.05792E-07	Small ribosomal subunit protein uS7;Small ribosomal subunit protein uS7, N-terminally processed	Rps5
Hypervirulent	-1.285723877	4.56144E-07	Regulator of nonsense transcripts 1	Upf1
Hypervirulent	-1.285627365	0.000430636	Guanylate kinase;guanylate kinase	Guk1
Hypervirulent	-1.284734917	0.000240592	Long-chain-fatty-acid--CoA ligase ACSBG2	Acsbg2
Hypervirulent	-1.284729195	0.013700064	Caldesmon 1	Cald1
Hypervirulent	-1.284512138	2.73059E-05	Transportin-1	Tnpo1
Hypervirulent	-1.283756638	0.003821341	Ig heavy chain V region AC38 205.12;Ig heavy chain V region MOPC 104E;Ig heavy chain V region J558	Ighv1-31;Ighv1-42;Ighv1-26;Ighv1-34
Hypervirulent	-1.283028412	3.30708E-06	Heat shock 70 kDa protein 1B;Heat shock 70 kDa protein 1A	Hspa1b;Hspa1a
Hypervirulent	-1.282014847	2.81112E-05	High mobility group protein B1	Hmgb1

Hypervirulent	-1.281273079	0.000877641	Eukaryotic peptide chain release factor GTP-binding subunit ERF3A	Gspt1
Hypervirulent	-1.280918503	0.014648078	MOB kinase activator 2	Mob2
Hypervirulent	-1.280281639	4.7903E-06	Nuclear factor NF-kappa-B p105 subunit;Nuclear factor NF-kappa-B p50 subunit	Nfkb1
Hypervirulent	-1.279489708	0.003260298	BPI fold-containing family A member 1	Bpifa1
Hypervirulent	-1.278866196	0.013862314	Polyhomeotic-like protein 2	Phc2
Hypervirulent	-1.277739906	0.000352499	Bridging integrator 2	Bin2
Hypervirulent	-1.275993729	0.003721406	Exostosin-1	Ext1
Hypervirulent	-1.275528336	5.65878E-07	Lupus La protein homolog	Ssb
Hypervirulent	-1.274725533	1.36484E-06	Copine-3	Cpne3
Hypervirulent	-1.274360847	0.005445381	Tumor protein D52	Tpd52
Hypervirulent	-1.272903442	6.84178E-06	Unconventional myosin-If	Myo1f
Hypervirulent	-1.272564507	8.19655E-06	Antithrombin-III	Serpinc1
Hypervirulent	-1.271689034	0.000302701	Plasma kallikrein;Plasma kallikrein heavy chain;Plasma kallikrein light chain	Klkb1
Hypervirulent	-1.269222832	0.000372205	Adapter molecule crk	Crk
Hypervirulent	-1.268149948	0.026484643	Pulmonary surfactant-associated protein B	Sftpb
Hypervirulent	-1.267568207	0.007530828	Aldose reductase-related protein 2	Akr1b8
Hypervirulent	-1.265798187	0.000660017	Isoaspartyl peptidase/L-asparaginase;Isoaspartyl peptidase/L-asparaginase alpha chain;Isoaspartyl peptidase/L-asparaginase beta chain	Asrgl1
Hypervirulent	-1.265130997	0.000152537	ATP-dependent 6-phosphofructokinase, liver type	Pfkl
Hypervirulent	-1.264602852	0.000547398	BCL2/adenovirus E1B 19 kDa protein-interacting protein 2	Bnip2
Hypervirulent	-1.264367104	0.001253865	ADAMTS-like 5	Adamtsl5
Hypervirulent	-1.263230705	2.68795E-11	Arginine--tRNA ligase, cytoplasmic	Rars1
Hypervirulent	-1.261525917	0.000117889	Actin-like 11	Actl11
Hypervirulent	-1.261041832	0.002567803	Glucosamine-6-phosphate isomerase 1	Gnpda1
Hypervirulent	-1.26044445	0.000915914	Ribose-phosphate pyrophosphokinase 1;ribose-phosphate diphosphokinase	Prps1;Prps1l3;Prps1l1
Hypervirulent	-1.259385681	0.033212703	Anaphase-promoting complex subunit 7	Anapc7
Hypervirulent	-1.257204437	0.014659276	Peroxisomal bifunctional enzyme;Enoyl-CoA hydratase/3,2-trans-enoyl-CoA isomerase;3-hydroxyacyl-CoA dehydrogenase	Ehhadh
Hypervirulent	-1.257144547	9.06613E-07	Cysteine--tRNA ligase, cytoplasmic	Cars1
Hypervirulent	-1.255848312	3.779E-10	Acetyl-CoA carboxylase 1	Acaca
Hypervirulent	-1.254895592	0.006014444	Immunoglobulin heavy variable 14	Ighv14-3;Ighv14-1;Ighv14-4;Ighv14-2
Hypervirulent	-1.254610825	1.11542E-06	AP-1 complex subunit mu-1	Ap1m1
Hypervirulent	-1.254423141	0.009725889	Protein S100-A11	S100a11
Hypervirulent	-1.252660751	0.004567814	Actin-related protein 2/3 complex subunit 4	Arpc4
Hypervirulent	-1.252165413	0.000253561	Fascin	Fscn1
Hypervirulent	-1.252105522	0.036814657	Small ribosomal subunit protein uS14	Rps29
Hypervirulent	-1.251234245	2.97736E-08	Heat shock protein 105 kDa	Hsph1
Hypervirulent	-1.250934601	1.08194E-06	Dedicator of cytokinesis protein 2	Dock2
Hypervirulent	-1.250805473	1.25525E-07	Rho-associated protein kinase 2;Rho-associated protein kinase	Rock2
Hypervirulent	-1.250640678	0.00108187	Selenocysteine lyase	Scly
Hypervirulent	-1.248640251	0.010745452	H-2 class II histocompatibility antigen, E-B beta chain;H-2 class II histocompatibility antigen, I-A beta chain;H-2 class II histocompatibility antigen, E-Q beta chain;H-2 class II histocompatibility antigen, E-S beta chain	H2-Eb1
Hypervirulent	-1.248120117	3.48101E-06	Proteasome subunit alpha type-6	Psma6
Hypervirulent	-1.247974586	1.15564E-05	Phospholipase A-2-activating protein	Plaa
Hypervirulent	-1.247439003	1.02847E-05	Large ribosomal subunit protein eL33	Rpl35a
Hypervirulent	-1.245868492	1.57793E-05	Serine/threonine-protein kinase 24;Serine/threonine-protein kinase 24 35 kDa subunit;Serine/threonine-protein kinase 24 12 kDa subunit	Stk24
Hypervirulent	-1.245499229	5.7201E-05	26S proteasome regulatory subunit 6A	Psmc3
Hypervirulent	-1.245255089	6.36398E-05	Isopentenyl-diphosphate Delta-isomerase 1	Idi1
Hypervirulent	-1.245217133	1.00434E-05	Protein arginine N-methyltransferase 1;type I protein arginine methyltransferase	Prmt1
Hypervirulent	-1.245096779	6.19008E-06	Ankyrin repeat domain-containing protein 13A	Ankrd13a
Hypervirulent	-1.243612099	0.001019412	Small ribosomal subunit protein uS15	Rps13
Hypervirulent	-1.2435009	0.003708493	Thiamin pyrophosphokinase 1	Tpk1

Hypervirulent	-1.243411255	4.00544E-05	COP9 signalosome complex subunit 3	Cops3
Hypervirulent	-1.241291809	3.26511E-05	Tro protein	Tro
Hypervirulent	-1.240855026	3.36425E-05	Dihydropteridine reductase	Qdpr
Hypervirulent	-1.240788841	1.10066E-09	26S proteasome regulatory subunit 7	Psmc2
Hypervirulent	-1.240250778	0.028626558	Potassium voltage-gated channel subfamily KQT member 1	Kcnq1
Hypervirulent	-1.23910408	0.000617191	Large ribosomal subunit protein uL11	Rpl12
Hypervirulent	-1.239086151	7.03212E-06	Cdc42-interacting protein 4	Trip10
Hypervirulent	-1.238409042	3.49277E-05	Immunity-related GTPase family M protein 3	Igtp
Hypervirulent	-1.23694725	0.000475258	Proteasome subunit alpha type-5	Psma5
Hypervirulent	-1.236535072	0.038795407	Protein NYNRIN	Nynrin
Hypervirulent	-1.235697937	0.000206016	Actin-related protein 2/3 complex subunit 2;Arp2/3 complex 34 kDa subunit	Arpc2
Hypervirulent	-1.234107018	1.89952E-05	Peptidyl-prolyl cis-trans isomerase D	Ppid
Hypervirulent	-1.233902168	0.017159561	Ubiquilin-2	Ubqln2
Hypervirulent	-1.233758926	0.000332147	Plasminogen;Plasmin heavy chain A;Activation peptide;Angiostatin;Plasmin heavy chain A, short form;Plasmin light chain B	Plg
Hypervirulent	-1.233592033	0.000142109	Glycogen synthase kinase-3 beta	Gsk3b
Hypervirulent	-1.231823921	0.000325216	Prefoldin subunit 3	Vbp1
Hypervirulent	-1.230900383	0.022707134	Plectin	Plec
Hypervirulent	-1.23007164	1.13753E-06	28 kDa heat- and acid-stable phosphoprotein	Pdap1
Hypervirulent	-1.229003716	0.0164217	Magnesium-dependent phosphatase 1	Mdp1
Hypervirulent	-1.228997421	0.022514842	Diacylglycerol kinase beta	Dgkb
Hypervirulent	-1.228570938	0.011819048	B-cell linker protein	Blnk
Hypervirulent	-1.226085854	0.002626827	Tumor necrosis factor alpha-induced protein 8-like protein 2	Tnfaip8l2
Hypervirulent	-1.22574501	0.000297568	Tubulin beta-4B chain	Tubb4b
Hypervirulent	-1.224407387	0.001655558	Serine/threonine-protein phosphatase 2A 56 kDa regulatory subunit epsilon isoform;Serine/threonine-protein phosphatase 2A 56 kDa regulatory subunit	Ppp2r5e
Hypervirulent	-1.223676872	6.30747E-05	Gephyrin;Molybdopterin adenylyltransferase;Molybdopterin molybdenumtransferase;Gephyrin;Molybdopterin molybdenumtransferase;Molybdopterin adenylyltransferase	Gphn
Hypervirulent	-1.222467232	1.77817E-06	Stress-induced-phosphoprotein 1	Stip1
Hypervirulent	-1.221901321	0.000551665	CYFIP-related Rac1 interactor A	Cyria
Hypervirulent	-1.221835518	0.007895693	Stromal membrane-associated protein 2	Smap2
Hypervirulent	-1.220257378	0.000776415	Secernin-2	Scrn2
Hypervirulent	-1.220190811	2.11238E-07	COP9 signalosome complex subunit 2	Cops2
Hypervirulent	-1.219790459	0.000766825	Calpain small subunit 1	Capns1
Hypervirulent	-1.218013763	0.001832722	Glutamine amidotransferase-like class 1 domain-containing protein 1	Gatd1
Hypervirulent	-1.21718235	0.000126297	Carboxypeptidase N subunit 2	Cpn2
Hypervirulent	-1.215406418	1.91609E-05	Apolipoprotein N	Apon
Hypervirulent	-1.214266968	0.003066076	RNA 3-terminal phosphate cyclase	RtcA;Rtca
Hypervirulent	-1.213538742	0.000168252	GTP-binding protein SAR1a	Sar1a
Hypervirulent	-1.210515213	0.001418833	Protein EVI2B	Evi2b
Hypervirulent	-1.20935936	1.26364E-06	General vesicular transport factor p115	Uso1
Hypervirulent	-1.209050179	0.003724655	UBX domain-containing protein 1	Ubxn1
Hypervirulent	-1.207568169	0.000822779	Endophilin-B2	Sh3glb2
Hypervirulent	-1.206873322	0.014732784	Fanconi anemia group J protein homolog	Brip1
Hypervirulent	-1.206586647	0.001681464	Microtubule-associated protein RP/EB family member 2	Mapre2
Hypervirulent	-1.206399918	0.001952474	TAR RNA-binding protein 1	Tarbp1
Hypervirulent	-1.202048874	0.002067206	Methylthioribulose-1-phosphate dehydratase	Apip
Hypervirulent	-1.20169754	2.70305E-08	Septin-9	Septin9
Hypervirulent	-1.201666069	0.001413761	Zinc finger protein 948	Zfp948
Hypervirulent	-1.20125885	0.000886322	Heterogeneous nuclear ribonucleoprotein A/B	Hnrnpab
Hypervirulent	-1.200588226	0.003140984	Protein CDV3	Cdv3
Hypervirulent	-1.200576401	6.05746E-08	Serine/threonine-protein phosphatase 2A 65 kDa regulatory subunit A alpha isoform	Ppp2r1a
Hypervirulent	-1.199184418	0.000301224	Galactokinase	Galk1
Hypervirulent	-1.197982216	0.011571229	Kinesin light chain;Kinesin light chain 1	Klc1
Hypervirulent	-1.197003365	0.00057548	Eukaryotic translation initiation factor 3 subunit G	Eif3g
Hypervirulent	-1.196822929	0.002182403	Glutamine synthetase;Palmitoyltransferase GLUL	Glul
Hypervirulent	-1.196032143	0.001496732	tRNA pseudouridine synthase-like 1	Pusl1
Hypervirulent	-1.195885849	0.000746219	Protein mono-ADP-ribosyltransferase PARP9	Parp9
Hypervirulent	-1.192464638	0.003105926	Pro-interleukin-16;Interleukin-16	Il16

Hypervirulent	-1.192208481	0.00206298	Transcription factor BTF3	Btf3
Hypervirulent	-1.191274834	0.000580518	Engulfment and cell motility protein 1	Elmo1
Hypervirulent	-1.190452576	0.019032854	Rearranged L-myc fusion sequence	Rlf
Hypervirulent	-1.190293312	0.010027809	p53-induced death domain-containing protein 1;PIDD-N;PIDD-C;PIDD-CC	Pidd1
Hypervirulent	-1.190089989	0.022597759	Protocadherin gamma subfamily A, 6	Pcdhga6
Hypervirulent	-1.188628769	0.000298267	Actin-related protein 10	Actr10
Hypervirulent	-1.187063026	0.000394926	Pyridoxal-dependent decarboxylase domain-containing protein 1	Pdxdc1
Hypervirulent	-1.186366272	0.000229174	BRISC and BRCA1-A complex member 1	Babam1
Hypervirulent	-1.185094452	0.001637725	Caspase-1;Caspase-1 subunit p20;Caspase-1 subunit p10	Casp1
Hypervirulent	-1.184388733	0.000584115	Large ribosomal subunit protein bL28m	Mrpl28
Hypervirulent	-1.18390274	3.42858E-05	Translin	Tsn
Hypervirulent	-1.183286476	4.92137E-08	Proteasome subunit alpha type-1	Psma1
Hypervirulent	-1.182881165	0.002595545	Dynactin subunit 6	Dctn6
Hypervirulent	-1.182413673	0.004478066	Translation initiation factor eIF2B subunit gamma	Eif2b3
Hypervirulent	-1.182312965	2.85061E-06	Isoleucine--tRNA ligase, cytoplasmic	Iars1
Hypervirulent	-1.182261467	4.10234E-05	Rho-associated protein kinase 1	Rock1
Hypervirulent	-1.180633163	4.15134E-05	Cell division cycle protein 23 homolog	Cdc23
Hypervirulent	-1.178827667	0.007495174	WW domain-binding protein 2	Wbp2
Hypervirulent	-1.176961136	0.001275825	Asparagine synthetase [glutamine-hydrolyzing]	Asns
Hypervirulent	-1.176068306	1.34689E-05	Trafficking protein particle complex subunit 13	Trappc13
Hypervirulent	-1.175494766	1.2267E-06	Coronin-1B;Coronin	Coro1b
Hypervirulent	-1.173544884	0.0112842	NEDD8-activating enzyme E1 regulatory subunit	Nae1
Hypervirulent	-1.171986771	0.017303974	Procathepsin L;Cathepsin L;Cathepsin L heavy chain;Cathepsin L light chain	Ctsl
Hypervirulent	-1.170939064	0.015532931	Cathepsin S	Ctss
Hypervirulent	-1.170682144	2.25634E-07	26S proteasome non-ATPase regulatory subunit 11	Psmd11
Hypervirulent	-1.16974144	7.07163E-05	Transforming protein RhoA;Rho-related GTP-binding protein RhoC	Rhoa;Rhoc
Hypervirulent	-1.169332886	4.05525E-06	Eukaryotic translation initiation factor 5A-1;Eukaryotic translation initiation factor 5A;Eukaryotic translation initiation factor 5A-2	Eif5a;Eif5a2
Hypervirulent	-1.169001007	0.003168992	Y-box-binding protein 3	Ybx3
Hypervirulent	-1.168080711	0.0008854	Vacuolar protein sorting-associated protein VTA1 homolog	Vta1
Hypervirulent	-1.168061638	0.003278288	ATPase GET3	Get3
Hypervirulent	-1.167822647	0.002436276	THUMP domain-containing protein 1	Thumpd1
Hypervirulent	-1.165560913	1.30767E-05	Eukaryotic translation initiation factor 5B	Eif5b
Hypervirulent	-1.164365196	0.003420889	Vacuolar protein-sorting-associated protein 25	Vps25
Hypervirulent	-1.163854599	0.015606923	Glutathione S-transferase theta-2;glutathione transferase	Gstt2
Hypervirulent	-1.162334633	2.44537E-07	PDZ and LIM domain protein 1	Pdlim1
Hypervirulent	-1.161874008	5.76658E-07	Squamous cell carcinoma antigen recognized by T-cells 3	Sart3
Hypervirulent	-1.161448288	3.5717E-07	Eukaryotic translation initiation factor 3 subunit E	Eif3e
Hypervirulent	-1.159607315	0.010899239	RasGEF domain family, member 1A	Rasgef1a
Hypervirulent	-1.158683586	0.000462281	Protein O-mannosyl-transferase TMTC2	Tmtc2
Hypervirulent	-1.158606529	0.009006978	WAP four-disulfide core domain protein 2	Wfdc2
Hypervirulent	-1.158235931	2.9988E-06	Proteasome activator complex subunit 3	Psme3
Hypervirulent	-1.155779266	0.00039326	RNA-binding protein 3	Rbm3
Hypervirulent	-1.155584335	0.005162011	Activated RNA polymerase II transcriptional coactivator p15	Sub1
Hypervirulent	-1.153226471	2.08759E-06	Tyrosine-protein kinase CSK	Csk
Hypervirulent	-1.152351952	3.9995E-07	Protein kinase C delta type;Protein kinase C delta type regulatory subunit;Protein kinase C delta type catalytic subunit;Protein kinase C	Prkcd
Hypervirulent	-1.15226593	4.83902E-05	Heparin cofactor 2	Serpind1
Hypervirulent	-1.152251434	0.001920813	Phosphoribosyl pyrophosphate synthase-associated protein 2	Prpsap2
Hypervirulent	-1.152093887	0.000187658	DnaJ homolog subfamily C member 2	Dnajc2
Hypervirulent	-1.151693726	4.00806E-06	Ras GTPase-activating protein-binding protein 1	G3bp1
Hypervirulent	-1.149482346	5.89537E-06	Eukaryotic translation initiation factor 3 subunit F	Eif3f
Hypervirulent	-1.149192429	3.92408E-06	AP-3 complex subunit beta-1	Ap3b1
Hypervirulent	-1.145612144	0.000748445	Carabin	Tbc1d10c
Hypervirulent	-1.145474052	0.000136137	Protein unc-13 homolog D	Unc13d
Hypervirulent	-1.144270134	7.42324E-05	E3 ubiquitin-protein ligase ARIH1;RBR-type E3 ubiquitin transferase	Arih1
Hypervirulent	-1.143403625	4.06379E-06	Trafficking protein particle complex subunit 3	Trappc3
Hypervirulent	-1.143185425	0.006689349	Tetratricopeptide repeat protein 38	Ttc38
Hypervirulent	-1.143013382	0.00370769	GTP-binding protein SAR1b	Sar1b
Hypervirulent	-1.142337799	4.21925E-05	GTP-binding protein Rheb	Rheb
Hypervirulent	-1.142202568	0.003337461	Pirin	Pir

Hypervirulent	-1.141322327	0.000195831	DNA replication licensing factor MCM5	Mcm5
Hypervirulent	-1.141171074	1.70456E-05	Purine nucleoside phosphorylase	Pnp;Gm49342
Hypervirulent	-1.140296745	8.46098E-05	Eukaryotic translation initiation factor 3 subunit D	Eif3d
Hypervirulent	-1.138694763	0.001184226	DNA replication licensing factor MCM2	Mcm2
Hypervirulent	-1.138592148	0.00074431	Actin-related protein 2	Actr2
Hypervirulent	-1.138407707	6.92865E-07	COP9 signalosome complex subunit 1	Gps1
Hypervirulent	-1.137946129	9.50722E-05	Complement C1s-1 subcomponent;Complement C1s-A subcomponent heavy chain;Complement C1s-A subcomponent light chain	C1s1
Hypervirulent	-1.136785126	0.004198044	Apolipoprotein A-I;Proapolipoprotein A-I;Truncated apolipoprotein A-I	Apoa1
Hypervirulent	-1.135258484	0.009753169	A-kinase anchor protein 3	Akap3
Hypervirulent	-1.134784889	3.35057E-06	Proteasome subunit alpha type-2	Psma2
Hypervirulent	-1.133864594	0.005569976	NPC intracellular cholesterol transporter 2	Npc2
Hypervirulent	-1.133181763	6.406E-05	Protein VAC14 homolog	Vac14
Hypervirulent	-1.132645035	8.31068E-05	SH2/SH3 adapter protein Nck1	Nck1
Hypervirulent	-1.132471848	0.000884721	Hsp90 co-chaperone Cdc37;Hsp90 co-chaperone Cdc37, N-terminally processed	Cdc37
Hypervirulent	-1.131296349	0.002244765	Phosphoserine aminotransferase	Psat1
Hypervirulent	-1.128071594	8.0368E-08	Septin-2	Septin2
Hypervirulent	-1.125014877	0.000236641	Ganglioside GM2 activator	Gm2a
Hypervirulent	-1.12460556	1.22056E-05	Polypyrimidine tract-binding protein 1	Ptbp1
Hypervirulent	-1.124281311	3.64732E-08	Guanylate cyclase soluble subunit alpha-1	Gucy1a1
Hypervirulent	-1.123962021	3.26294E-05	Adenosylhomocysteinase	Ahcy
Hypervirulent	-1.123802185	0.000849393	Protein FAM3C	Fam3c
Hypervirulent	-1.122358704	0.00044333	STE20-like serine/threonine-protein kinase	Slk
Hypervirulent	-1.122237778	0.000704338	Protein pelota homolog	Pelo
Hypervirulent	-1.121881104	1.73941E-06	Ubiquitin carboxyl-terminal hydrolase 5;Ubiquitin carboxyl-terminal hydrolase	Usp5
Hypervirulent	-1.121142197	0.000163834	Small ribosomal subunit protein eS27-like;Small ribosomal subunit protein eS27;40S ribosomal protein S27	Rps27l;Rps27
Hypervirulent	-1.119816208	0.003938119	Asparagine--tRNA ligase, cytoplasmic;asparagine--tRNA ligase	Nars1
Hypervirulent	-1.119175339	1.19788E-05	Major vault protein	Mvp
Hypervirulent	-1.118861389	5.23306E-05	Sec24-related gene family, member B	Sec24b
Hypervirulent	-1.118717194	6.30926E-08	AP-1 complex subunit gamma-1;AP-1 complex subunit gamma	Ap1g1
Hypervirulent	-1.117921448	0.006278783	Interleukin-1 receptor-associated kinase 4	Irak4
Hypervirulent	-1.117887878	0.000120034	Large ribosomal subunit protein eL34	Rpl34
Hypervirulent	-1.114411736	4.45275E-05	Annexin A4;Annexin	Anxa4
Hypervirulent	-1.111966515	0.000145878	Lipoma-preferred partner homolog	Lpp
Hypervirulent	-1.111247444	1.41191E-05	Large ribosomal subunit protein uL30	Rpl7
Hypervirulent	-1.111211777	5.67208E-06	Eukaryotic translation initiation factor 3 subunit B	Eif3b
Hypervirulent	-1.110178566	0.003239165	GDP-L-fucose synthase	Gfus
Hypervirulent	-1.109922981	1.11454E-08	Multifunctional protein CAD;Glutamine-dependent carbamoyl-phosphate synthase;Glutamine amidotransferase;Ammonium-dependent carbamoyl phosphate synthase;Aspartate carbamoyltransferase;Dihydroorotase	Cad
Hypervirulent	-1.10966053	0.003913671	Nucleobindin-2;Nesfatin-1	Nucb2
Hypervirulent	-1.109436035	0.004476324	Proteasome subunit beta type-2	Psmb2
Hypervirulent	-1.104734421	0.000886513	Elongin-C	Eloc
Hypervirulent	-1.104362869	0.000208607	SAP domain-containing ribonucleoprotein	Sarnp
Hypervirulent	-1.102126503	0.000810699	Neogenin	Neo1
Hypervirulent	-1.101279068	7.57664E-05	AP-1 complex subunit beta-1;AP complex subunit beta	Ap1b1
Hypervirulent	-1.100558662	0.000351306	Cell division control protein 42 homolog	Cdc42
Hypervirulent	-1.100297928	0.000891047	Pleckstrin homology domain-containing family F member 2	Plekhf2
Hypervirulent	-1.099415016	0.002305385	Trafficking protein particle complex subunit 4;Trafficking protein particle complex subunit	Trappc4
Hypervirulent	-1.098231697	0.002889011	Serine/arginine-rich splicing factor 4	Srsf4
Hypervirulent	-1.098096466	0.00035123	G-protein-signaling modulator 3	Gpsm3
Hypervirulent	-1.098074341	2.99649E-06	Glutamate decarboxylase 1	Gad1
Hypervirulent	-1.097083282	0.000409459	SEC23-interacting protein	Sec23ip
Hypervirulent	-1.096743584	5.01088E-05	Scinderin	Scin
Hypervirulent	-1.095985985	0.003166079	Striatin-interacting protein 1	Strip1
Hypervirulent	-1.095143127	0.001462495	RalBP1-associated Eps domain-containing protein 2	Reps2
Hypervirulent	-1.09245491	9.86293E-06	1-phosphatidylinositol 4,5-bisphosphate phosphodiesterase gamma-2	Plcg2

Hypervirulent	-1.09094696	0.002151987	Plasma alpha-L-fucosidase	Fuca2
Hypervirulent	-1.090625191	0.000584205	Pigment epithelium-derived factor	Serpinf1
Hypervirulent	-1.089157677	8.49518E-05	26S proteasome regulatory subunit 6B	Psmc4
Hypervirulent	-1.088881683	0.003652921	Nicotinamide/nicotinic acid mononucleotide adenylyltransferase 1	Nmnat1
Hypervirulent	-1.088432884	0.000128464	Large ribosomal subunit protein uL5	Rpl11
Hypervirulent	-1.087031746	0.000545189	Serine/threonine-protein kinase 4;Serine/threonine-protein kinase 4 37kDa subunit;Serine/threonine-protein kinase 4 18kDa subunit	Stk4
Hypervirulent	-1.085012245	0.000188624	Tetranectin	Clec3b
Hypervirulent	-1.083810997	3.7633E-06	Large ribosomal subunit protein eL8	Rpl7a
Hypervirulent	-1.083442688	0.000208032	Tyrosine-protein phosphatase non-receptor type 11	Ptpn11
Hypervirulent	-1.082826805	6.39203E-07	182 kDa tankyrase-1-binding protein	Tnks1bp1
Hypervirulent	-1.078333282	0.000108944	C-terminal-binding protein 2	Ctbp2
Hypervirulent	-1.0781147	0.000278664	Pendrin	Slc26a4
Hypervirulent	-1.076998901	0.003185645	Kv channel-interacting protein 4	Kcnip4
Hypervirulent	-1.076696205	1.08611E-06	Eukaryotic translation initiation factor 3 subunit C	Eif3c
Hypervirulent	-1.075383949	0.000693415	Vacuolar protein sorting-associated protein 4B	Vps4b
Hypervirulent	-1.074230003	0.001347377	Glucosamine-6-phosphate isomerase 2;Glucosamine-6-phosphate isomerase	Gnpda2
Hypervirulent	-1.073075867	0.001755425	Large ribosomal subunit protein eL32	Rpl32
Hypervirulent	-1.072844887	0.000154046	Vacuolar protein sorting-associated protein 35	Vps35
Hypervirulent	-1.072010994	0.002090817	Protein PRRC1	Prrc1
Hypervirulent	-1.071374893	0.000973783	GTP-binding protein 1	Gtpbp1
Hypervirulent	-1.070086479	2.0386E-05	Cytosolic purine 5-nucleotidase	Nt5c2
Hypervirulent	-1.069556808	0.0001681	Bifunctional polynucleotide phosphatase/kinase;Polynucleotide 3-phosphatase;Polynucleotide 5-hydroxyl-kinase	Pnkp
Hypervirulent	-1.068411636	0.000297266	Rho guanine nucleotide exchange factor 6	Arhgef6
Hypervirulent	-1.067868042	1.80511E-06	Cullin-associated NEDD8-dissociated protein 1	Cand1
Hypervirulent	-1.0666605	3.80594E-07	Eukaryotic translation initiation factor 4 gamma 1	Eif4g1
Hypervirulent	-1.066338348	0.00016999	Oxysterol-binding protein-related protein 11;Oxysterol-binding protein	Osbpl11
Hypervirulent	-1.064768982	2.63848E-05	Calcium homeostasis modulator protein 6	Calhm6
Hypervirulent	-1.062615013	0.000896415	Actin-related protein 2/3 complex subunit 5	Arpc5
Hypervirulent	-1.061013794	0.000106324	Thimet oligopeptidase	Thop1
Hypervirulent	-1.059579277	0.000101082	Lysophosphatidylcholine acyltransferase 2	Lpcat2
Hypervirulent	-1.05952034	0.000570348	Serine/threonine-protein phosphatase 6 catalytic subunit;Serine/threonine-protein phosphatase	Ppp6c
Hypervirulent	-1.058548927	0.002067901	Galectin-3;Galectin	Lgals3
Hypervirulent	-1.058238792	1.39474E-06	Heterogeneous nuclear ribonucleoprotein Q	Syncrip
Hypervirulent	-1.054501152	0.000494852	Superoxide dismutase [Cu-Zn]	Sod1
Hypervirulent	-1.054236412	0.000715817	Obg-like ATPase 1	Ola1
Hypervirulent	-1.053536606	0.000381129	Integrin alpha-X	Itgax
Hypervirulent	-1.052458382	9.62417E-07	Heat shock 70 kDa protein 4	Hspa4
Hypervirulent	-1.051750183	7.66588E-07	Actin-binding Rho-activating protein	Abra
Hypervirulent	-1.050119781	9.32303E-06	Malignant T-cell-amplified sequence 1	Mcts1
Hypervirulent	-1.049911308	2.43478E-05	Heat shock cognate 71 kDa protein	Hspa8
Hypervirulent	-1.047085953	1.09547E-05	Cell cycle and apoptosis regulator protein 2	Ccar2
Hypervirulent	-1.044927597	9.72186E-06	Histidine-rich glycoprotein	Hrg
Hypervirulent	-1.044155121	4.56407E-06	Macrophage mannose receptor 1	Mrc1
Hypervirulent	-1.043800545	0.000101895	Elongin-B	Elob
Hypervirulent	-1.043591881	7.41522E-05	L-selectin	Sell
Hypervirulent	-1.040995979	0.000296341	Ethylmalonyl-CoA decarboxylase	Echdc1
Hypervirulent	-1.040554619	0.00079378	ATP-binding cassette sub-family F member 1	Abcf1
Hypervirulent	-1.03680687	4.50383E-05	Immunoglobulin-binding protein 1	Igbp1
Hypervirulent	-1.035950851	0.000530894	MARCKS-related protein	Marcksl1
Hypervirulent	-1.034822273	0.000400123	Cytoplasmic dynein 1 heavy chain 1	Dync1h1
Hypervirulent	-1.034474182	0.000970049	Transforming acidic coiled-coil-containing protein 1	Tacc1
Hypervirulent	-1.0342062	2.23035E-06	26S proteasome non-ATPase regulatory subunit 1	Psmd1
Hypervirulent	-1.033809853	4.33529E-06	26S proteasome non-ATPase regulatory subunit 2	Psmd2
Hypervirulent	-1.033252907	0.000356749	Pro-cathepsin H;Cathepsin H mini chain;Cathepsin H;Cathepsin H heavy chain;Cathepsin H light chain;Pro-cathepsin H	Ctsh
Hypervirulent	-1.030298996	0.00051205	Alpha-aminoadipic semialdehyde dehydrogenase;aldehyde dehydrogenase (NAD(+))	Aldh7a1
Hypervirulent	-1.029800797	0.000121954	Ras-related protein Rab-1A	Rab1a;Rab1A

Hypervirulent	-1.025774765	0.000615015	Phosphomannomutase 2	Pmm2
Hypervirulent	-1.025532341	1.44657E-05	26S proteasome non-ATPase regulatory subunit 12	Psmd12
Hypervirulent	-1.021543312	0.000215688	Serine protease inhibitor A3N	Serpina3n
Hypervirulent	-1.018413353	1.36523E-07	Ras GTPase-activating-like protein IQGAP2	Iqgap2
Hypervirulent	-1.017605019	4.45366E-06	Cytoplasmic dynein 1 light intermediate chain 2;Dynein light intermediate chain	Dync1li2
Hypervirulent	-1.017072296	1.467E-06	Aminopeptidase N	Anpep
Hypervirulent	-1.016552353	4.00406E-05	Protein Largen	Prr16
Hypervirulent	-1.01537323	2.26609E-06	Phospholipid-transporting ATPase ABCA3;150 Kda mature form	Abca3
Hypervirulent	-1.013269806	0.000240936	UDP-N-acetylhexosamine pyrophosphorylase	Uap1
Hypervirulent	-1.013037872	0.000193676	Large ribosomal subunit protein uL18	Rpl5
Hypervirulent	-1.012342834	3.12844E-06	Protein disulfide-isomerase A4	Pdia4
Hypervirulent	-1.00271759	4.88701E-05	Insulin-degrading enzyme	Ide
Hypervirulent	-1.002699471	5.29212E-05	Constitutive coactivator of PPAR-gamma-like protein 1	FAM120A
Hypervirulent	-1.001502419	0.000104197	Beta-1,4 N-acetylgalactosaminyltransferase 1	B4galnt1
Hypervirulent	-0.996361732	7.17357E-05	Septin-4	Septin4
Hypervirulent	-0.989731026	1.7499E-06	Growth factor receptor-bound protein 2	Grb2
Hypervirulent	-0.989652061	9.20574E-05	EH domain-containing protein 1	Ehd1
Hypervirulent	-0.988554764	2.2847E-05	Endoplasmic reticulum aminopeptidase 1	Erap1
Hypervirulent	-0.984703445	6.14571E-06	Importin-7	Ipo7
Hypervirulent	-0.981459618	9.95837E-06	TAR DNA-binding protein 43	Tardbp
Hypervirulent	-0.980976105	3.88614E-06	Eukaryotic translation initiation factor 3 subunit A	Eif3a
Hypervirulent	-0.977660751	1.56493E-06	Large ribosomal subunit protein uL3	Rpl3
Latent	0.959079742	5.50847E-07	Heterochromatin protein 1-binding protein 3	Hp1bp3
Latent	0.978273201	1.89683E-05	Nucleolar transcription factor 1	Ubtf
Latent	0.981153107	3.51106E-09	Integrin alpha-6;Integrin alpha-6 heavy chain;Integrin alpha-6 light chain;Processed integrin alpha-6	Itga6
Latent	0.983045769	1.65706E-05	Myosin-10	Myh10
Latent	0.998971939	2.35405E-05	rRNA 2-O-methyltransferase fibrillarin	Fbl
Latent	1.003815079	2.06498E-05	Flavin-containing monooxygenase 1	Fmo1
Latent	1.017887115	0.000437408	Tropomodulin-1	Tmod1
Latent	1.018624306	2.82838E-05	Nesprin-2	Syne2
Latent	1.019000626	0.00040398	Carbonic anhydrase 4	Ca4;Car4
Latent	1.020404625	0.000533668	Histone H2AX	H2ax
Latent	1.023772621	5.87256E-06	Guanine nucleotide-binding protein G(i) subunit alpha-1	Gnai1
Latent	1.02514782	0.000652506	Integrin beta-1	Itgb1
Latent	1.029786682	4.5545E-06	Equilibrative nucleoside transporter 1	Slc29a1
Latent	1.032592201	0.000573817	Immnoglobulin heavy variable 8	Ighv8-11
Latent	1.034607315	0.000606173	Junctional adhesion molecule A	F11r
Latent	1.039638901	0.000283773	Collagen alpha-1(XII) chain	Col12a1
Latent	1.044926262	7.39071E-07	Junction plakoglobin	Jup
Latent	1.047211266	9.68787E-07	Platelet endothelial cell adhesion molecule	Pecam1
Latent	1.04738884	0.000738934	Polycystin-2	Pkd2
Latent	1.047521019	1.07386E-05	Desmin	Des
Latent	1.055035782	0.000130037	Cadherin-5	Cdh5
Latent	1.056051445	0.001933867	Cysteine-rich and transmembrane domain-containing protein 1	Cystm1
Latent	1.059929466	1.95265E-05	Neprilysin	Mme
Latent	1.061550713	4.84019E-06	Hematopoietic progenitor cell antigen CD34	Cd34
Latent	1.061747551	0.000646327	Optic atrophy 3 protein homolog	Opa3
Latent	1.064051437	0.00194832	Actin, alpha cardiac muscle 1;Actin, alpha cardiac muscle 1, intermediate form;Actin, aortic smooth muscle;Actin, aortic smooth muscle, intermediate form;Actin, alpha skeletal muscle;Actin, alpha skeletal muscle, intermediate form;Actin, gamma-enteric smooth muscle;Actin, gamma-enteric smooth muscle, intermediate form;Actin, aortic smooth muscle	Actc1;Acta2;Acta1;Actg2
Latent	1.066545105	5.09352E-07	Collagen alpha-1(VI) chain	Col6a1
Latent	1.06918869	0.002087728	Troponin I, cardiac muscle	Tnni3
Latent	1.070176697	1.56153E-06	DNA topoisomerase 2-beta	Top2b
Latent	1.080343819	0.003758474	Hepatocyte growth factor receptor	Met
Latent	1.085607719	3.50973E-05	Lysyl oxidase homolog 1	Loxl1
Latent	1.088150597	2.92409E-05	H/ACA ribonucleoprotein complex subunit 3	Nop10
Latent	1.092188072	0.002329766	Ligand-dependent nuclear receptor corepressor-like protein	Lcorl

Latent	1.096608734	4.88566E-05	DNA-directed primase/polymerase protein	Primpol
Latent	1.096761513	0.001891001	F-box only protein 38	Fbxo38
Latent	1.097249031	0.000194241	Plectin	Plec
Latent	1.097420311	0.001935678	Coiled-coil domain-containing protein 42	Ccdc42
Latent	1.098497391	0.002050326	Ceramide synthase 5	Cers5
Latent	1.099843788	0.002295418	Nck-associated protein 5-like	Nckap5l
Latent	1.101467133	0.004386789	Integrin beta-7	Itgb7
Latent	1.10369091	0.006785679	Regulator of telomere elongation helicase 1	Rtel1
Latent	1.105585861	0.005505593	Integrin alpha-11	Itga11
Latent	1.108638	0.000584856	Myosin-14	Myh14
Latent	1.109131813	0.002329615	Guanylate-binding protein 4	Gbp4
Latent	1.109885979	0.001660494	Vang-like protein 1;Vang-like protein	Vangl1
Latent	1.1102108	0.000397443	Dematin	Dmtn
Latent	1.112181473	0.001583341	Myosin phosphatase Rho-interacting protein	Mprip
Latent	1.11487484	7.3804E-06	Lamin-B1	Lmnb1
Latent	1.115283012	0.000174256	Metallothionein-1	Mt1
Latent	1.122109985	0.008252284	Nucleoporin Nup43	Nup43
Latent	1.123631668	0.009948981	WD repeat-containing protein 3	Wdr3
Latent	1.124030495	0.008735151	Pyruvate kinase;Pyruvate kinase PKLR	Pklr
Latent	1.125590897	0.004306062	RIKEN cDNA 4930407I10 gene	4930407I10Rik
Latent	1.127663803	4.26857E-06	Fibronectin;Fibronectin;Anastellin	Fn1
Latent	1.12930603	0.007329049	Beclin-2	Becn2
Latent	1.130997849	1.77572E-05	Alkaline phosphatase, tissue-nonspecific isozyme	Alpl
Latent	1.13148632	0.001897567	Gem (Nuclear organelle) associated protein 4	Gemin4
Latent	1.13314991	6.32147E-05	Dipeptidase 1;Dipeptidase	Dpep1
Latent	1.133717537	0.000344635	Caveolae-associated protein 1	Cavin1
Latent	1.134555244	0.000312326	Delta-sarcoglycan	Sgcd
Latent	1.134910583	0.001797103	Connector enhancer of kinase suppressor of Ras 1	Cnksr1
Latent	1.137138939	0.001566304	Nurim	Nrm
Latent	1.146217537	0.011514878	Interferon regulatory factor 2-binding protein 2	Irf2bp2
Latent	1.146852303	0.000208262	Peroxiredoxin-2	Prdx2
Latent	1.148352432	0.003837877	Chondroadherin	Chad
Latent	1.148801994	6.97358E-05	55 kDa erythrocyte membrane protein	Mpp1
Latent	1.149482155	0.010765779	Probable E3 ubiquitin-protein ligase TRIML1	Triml1
Latent	1.151226616	0.004728367	Netrin-4	Ntn4
Latent	1.151380348	0.006902407	PEST proteolytic signal-containing nuclear protein	Pcnp
Latent	1.152119827	0.007702328	Ras and EF-hand domain-containing protein homolog	Rasef
Latent	1.152512932	0.010385136	Protein THEMIS3	Themis3
Latent	1.152601051	0.010607517	SR-related and CTD-associated factor 8	Scaf8
Latent	1.154411125	3.5242E-05	Beta-2-syntrophin	Sntb2
Latent	1.155237007	0.001537403	CEP295 N-terminal-like protein	Cep295nl
Latent	1.15551281	0.003017417	N-alpha-acetyltransferase 20	Naa20
Latent	1.155808067	0.00043694	Transcription factor Sp7	Sp7
Latent	1.160591698	0.001162089	Solute carrier organic anion transporter family member 2A1	Slco2a1
Latent	1.160628891	0.015817467	Pyroglutamyl-peptidase 1;Pyroglutamyl-peptidase I	Pgpep1
Latent	1.161701012	0.000107995	Fibrinogen beta chain;Fibrinopeptide B;Fibrinogen beta chain	Fgb
Latent	1.162734795	0.006067302	E3 ubiquitin-protein ligase TRIM47	Trim47
Latent	1.167924309	0.01139666	Schlafen family member 1	Slfn1
Latent	1.171263695	2.01181E-05	Cadherin-13	Cdh13
Latent	1.171559906	0.006867287	Titin	Ttn
Latent	1.173310852	1.42972E-05	Splicing factor C9orf78 homolog	
Latent	1.174225998	0.008390391	Pre-mRNA-splicing factor 38A	Prpf38a
Latent	1.17431469	0.009737941	Kinesin-like protein KIF23;Kinesin-like protein	Kif23
Latent	1.174887085	0.006203009	Leucine-rich repeat-containing 10B	Lrrc10b
Latent	1.175565338	0.003358213	Rhomboid-related protein 2;Rhomboid-related protein 2, N-terminal fragment;Rhomboid-related protein 2, C-terminal fragment	Rhbdl2
Latent	1.175757217	0.000372932	Hemoglobin, theta 1B	Hbq1b
Latent	1.17619381	1.1707E-05	Disintegrin and metalloproteinase domain-containing protein 10	Adam10
Latent	1.17649765	0.000110184	Spectrin beta chain, non-erythrocytic 1	Sptbn1
Latent	1.178339005	0.000111734	Alpha-1-acid glycoprotein 2	Orm2
Latent	1.179646873	3.73439E-07	Laminin subunit alpha-4	Lama4

Latent	1.1813097	0.002881851	Centrobin	Cntrob
Latent	1.181402206	2.23289E-05	Platelet glycoprotein 4	Cd36
Latent	1.181788254	0.000636647	Regulator of G-protein signaling 7-binding protein	Rgs7bp
Latent	1.181983948	0.008186992	Collagen type V alpha 3 chain	Col5a3
Latent	1.185441399	0.023078867	Disintegrin and metalloproteinase domain-containing protein 25	Adam25
Latent	1.188096809	0.004857057	USP6 N-terminal-like protein	Usp6nl
Latent	1.189280891	0.001846201	Acyl-CoA synthetase short-chain family member 3, mitochondrial	Acss3
Latent	1.190809441	0.01971413	Gamma-1-syntrophin	Sntg1
Latent	1.191231155	0.003120071	rRNA/tRNA 2-O-methyltransferase fibrillarin-like protein 1	Fbll1
Latent	1.191376305	0.002619864	ETS translocation variant 3	Etv3
Latent	1.191618538	0.01125779	Transmembrane protein 119	Tmem119
Latent	1.19261055	4.25824E-05	Spectrin alpha chain, non-erythrocytic 1;	Sptan1
Latent	1.193294716	0.000107796	Occludin	Ocln
Latent	1.193865967	0.000213634	Golgi-associated kinase 1A	Gask1a
Latent	1.194037628	0.013884092	Serine/threonine-protein kinase RIO1;non-specific serine/threonine protein kinase	Riok1
Latent	1.195587921	0.023890025	Phosphorylase b kinase gamma catalytic chain, liver/testis isoform	Phkg2
Latent	1.197084427	0.000839382	C-type lectin domain family 1 member A	Clec1a
Latent	1.197854996	0.022179259	N-acetylmuramoyl-L-alanine amidase	Pglyrp2
Latent	1.19806385	0.007706157	MICAL-like protein 1	Micall1
Latent	1.198300934	4.77982E-06	E3 ubiquitin-protein ligase MIB2	Mib2
Latent	1.199936295	0.007681477	Rab-interacting lysosomal protein	Rilp
Latent	1.205172157	4.73812E-05	Neuroplastin	Nptn
Latent	1.205381393	0.002155113	Coiled-coil domain-containing 192	Ccdc192
Latent	1.208086777	0.004478275	Acylcarnitine hydrolase	Ces2c
Latent	1.208446312	0.00288953	5-AMP-activated protein kinase subunit gamma-2	Prkag2
Latent	1.213368988	0.001265604	Gap junction alpha-5 protein;Gap junction protein	Gja5
Latent	1.214864349	0.004735221	Oxysterol-binding protein	Osbpl7
Latent	1.216143417	0.004083222	DNA-binding protein RFX6	Rfx6
Latent	1.216800308	0.001023725	Coiled-coil domain-containing protein 8 homolog	Ccdc8
Latent	1.216924095	0.002261484	Basic helix-loop-helix ARNT-like protein 2	Bmal2
Latent	1.217388535	0.020128808	Cadherin-10	Cdh10
Latent	1.217778206	0.015988535	Sperm flagellar protein 2	Spef2
Latent	1.217797661	0.026956748	Zinc finger protein 445	Znf445
Latent	1.217996025	0.021185064	Synapsin-3	Syn3
Latent	1.218593597	0.014069896	Hyaluronan and proteoglycan link protein 4	Hapln4
Latent	1.219524002	0.003060952	Hemoglobin subunit epsilon-Y2	Hbb-y
Latent	1.219937706	0.014911876	Troponin C, slow skeletal and cardiac muscles	Tnnc1
Latent	1.224769974	0.006776245	ADP-ribosylation factor-like protein 15	Arl15
Latent	1.226635361	0.006827428	Platelet factor 4	Pf4
Latent	1.228559875	3.50677E-06	Collagen alpha-1(XVIII) chain;Endostatin;Non-collagenous domain 1	Col18a1
Latent	1.229219055	3.37329E-07	Integrin alpha-1	Itga1
Latent	1.230005074	0.000279974	Glutaminyl-peptide cyclotransferase-like protein	Qpctl
Latent	1.232042885	0.000266627	Cadherin-2	Cdh2
Latent	1.232535553	6.49852E-05	Caveolae-associated protein 2	Cavin2
Latent	1.232929993	7.10097E-07	Intercellular adhesion molecule 2	Icam2
Latent	1.233627319	0.001281006	Protein cereblon	Crbn
Latent	1.23481884	0.000334424	Fibrinogen alpha chain;Fibrinopeptide A;Fibrinogen alpha chain	Fga
Latent	1.234848976	0.033446706	Tryptase beta-2	Tpsb2
Latent	1.236060333	0.003083996	Vomeromodulin	Bpifb9a;Bpifb9b
Latent	1.236203003	0.023091463	UDP-N-acetylglucosamine transferase subunit ALG14 homolog	Alg14
Latent	1.240678406	0.001207224	Ral GTPase-activating protein subunit alpha-2	Ralgapa2
Latent	1.241534615	0.012914768	Mitochondrial disaggregase;Mitochondrial disaggregase, cleaved form	Clpb
Latent	1.242116356	0.007293607	IQ motif-containing GTPase-activating protein 3	Iqgap3
Latent	1.244030762	0.000166674	Calcium-independent phospholipase A2-gamma	Pnpla8
Latent	1.245462418	4.00957E-05	Amine oxidase [copper-containing] 3;Amine oxidase	Aoc3
Latent	1.245509911	0.00300147	Hairy/enhancer-of-split related with YRPW motif-like protein	Heyl
Latent	1.245802689	0.041136547	Cytochrome b5	Cyb5a
Latent	1.246422958	0.004276714	Lysine-specific histone demethylase 1B	Kdm1b
Latent	1.246718407	1.0877E-07	Axin-2	Axin2

Latent	1.249671364	7.77957E-05	Zinc finger protein 804B	Zfp804b
Latent	1.249865913	0.038696292	Zinc finger protein 687	Znf687
Latent	1.250520325	0.000301808	Histone H3.3;Histone H3;Histone H3.3C;Histone H3.1;Histone H3.2	H3-3a;H3f3a;H3-5;H3c1;H3c2;H3c14;H3f4
Latent	1.252041626	0.004818155	Selenocysteine insertion sequence-binding protein 2-like	Secisbp2l
Latent	1.254106903	5.90054E-07	Beta-adducin	Add2
Latent	1.254503822	8.16764E-05	Angiotensin-converting enzyme;Angiotensin-converting enzyme, soluble form;Angiotensin-converting enzyme	Ace
Latent	1.254982185	0.011600656	Myotubularin-related protein 14	Mtmr14
Latent	1.256684494	0.024702106	Matrix Gla protein	Mgp
Latent	1.258666229	0.016981586	Kinesin-like protein KIF3A;Kinesin-like protein	Kif3a
Latent	1.259057617	0.011850581	GTP-binding protein REM 1	Rem1
Latent	1.259301186	0.027965281	Phosphatidylinositol 3-kinase C2 domain-containing subunit gamma	Pik3c2g
Latent	1.260079956	0.011392978	Cardiac-enriched FHL2-interacting protein	CEFIP
Latent	1.260321808	0.001281209	Nectin-2	Nectin2
Latent	1.260829926	0.004535495	Coiled-coil domain-containing protein 124	Ccdc124
Latent	1.264855766	1.66445E-07	SUN domain-containing protein 1	Sun1
Latent	1.266390419	3.14876E-05	Ribonuclease 4	Rnase4
Latent	1.266633224	5.26258E-07	Collagen alpha-1(III) chain	Col3a1
Latent	1.268943787	0.008099333	Immunoglobulin superfamily containing leucine-rich repeat protein 2	Islr2
Latent	1.269360161	0.000409461	Protein S100-A16	S100a16
Latent	1.26956501	0.030028673	Uncharacterized protein C16orf96 homolog	
Latent	1.272087288	0.007602496	Ras association domain-containing protein 8	Rassf8
Latent	1.2731287	0.003965808	Retrotransposon-derived protein PEG10	Peg10
Latent	1.277509499	0.011791996	tRNA-specific adenosine deaminase 1	Adat1
Latent	1.278984451	6.4902E-05	Histone-lysine N-methyltransferase KMT5C	Kmt5c
Latent	1.279732132	0.000126111	Decorin	Dcn
Latent	1.279959869	0.015915303	3-5 exoribonuclease HELZ2;RNA helicase	Helz2
Latent	1.280749893	6.40823E-05	CREB-regulated transcription coactivator 2	Crtc2
Latent	1.281346512	0.004619476	Chromosome transmission fidelity protein 18 homolog	Chtf18
Latent	1.281521034	0.013033497	Kinase suppressor of Ras 1	Ksr1
Latent	1.281999588	0.006671555	Guanine nucleotide-binding protein-like 3-like protein	Gnl3l
Latent	1.284321404	0.032629574	Barrier-to-autointegration factor;Barrier-to-autointegration factor, N-terminally processed	Banf1
Latent	1.284747696	0.003410933	Advanced glycosylation end product-specific receptor	Ager
Latent	1.285070801	0.008343539	Protein misato homolog 1	Msto1
Latent	1.28561554	0.011876845	Sialate:O-sulfotransferase 2	Wscd2
Latent	1.286489677	0.007982262	Ankyrin repeat and SOCS box protein 1	Asb1
Latent	1.289490128	0.000119197	Oxysterol-binding protein 1	Osbp
Latent	1.29172554	0.049443585	Serine protease 57	Prss57
Latent	1.292643929	0.001947518	Sodium- and chloride-dependent GABA transporter 3	Slc6a11
Latent	1.294966125	0.03942946	Solute carrier family 35 member F3	Slc35f3
Latent	1.296912384	0.046721395	Dual specificity tyrosine-phosphorylation-regulated kinase 1B	Dyrk1b
Latent	1.297428322	0.000518126	Chaperonin-containing TCP1, subunit 8 (theta)-like 1	Cct8l1
Latent	1.299013901	0.006913218	Spermatogenesis-associated protein 21	Spata21
Latent	1.299459648	0.012573286	Low-density lipoprotein receptor-related protein 5	Lrp5
Latent	1.299748802	0.02484771	WD repeat-containing protein 76	Wdr76
Latent	1.299813843	0.02300996	V-type proton ATPase subunit C 2;V-type proton ATPase subunit C	Atp6v1c2
Latent	1.299829483	5.9036E-06	Centromere protein V	Cenpv
Latent	1.299998665	0.005673842	Tyrosine-protein phosphatase non-receptor type 13	Ptpn13
Latent	1.301134682	0.010582334	RNA helicase;Putative helicase MOV-10	Mov10
Latent	1.301711082	0.024930446	Glutamate receptor ionotropic, delta-1	Grid1
Latent	1.302997971	0.04120295	DNA annealing helicase and endonuclease ZRANB3;DNA annealing helicase ZRANB3;Endonuclease ZRANB3	Zranb3
Latent	1.303204155	0.003152441	Short transient receptor potential channel 1	Trpc1
Latent	1.304111671	0.005608669	DCN1-like protein 5;DCN1-like protein	Dcun1d5
Latent	1.30497303	0.003280725	Immunoglobulin heavy constant epsilon	IGHE
Latent	1.305290413	0.004240392	Succinate dehydrogenase assembly factor 1, mitochondrial	Sdhaf1

Latent	1.30685482	0.011330069	Natriuretic peptides A;Long-acting natriuretic peptide;Vessel dilator;Kaliuretic peptide;Urodilatin;Auriculin-C;Auriculin-D;Atrial natriuretic peptide;Auriculin-B;Auriculin-A;Atriopeptin-1;Atriopeptin-2;Atriopeptin-3	Nppa
Latent	1.306933594	6.59749E-07	Periostin	Postn
Latent	1.307324219	0.021649487	Voltage-dependent calcium channel subunit alpha-2/delta-4;Voltage-dependent calcium channel subunit alpha-2-4;Voltage-dependent calcium channel subunit delta-4	Cacna2d4
Latent	1.308435631	8.85078E-06	Caveolin-1;Caveolin	Cav1
Latent	1.30949688	0.031098741	Phospholipase DDHD1	Ddhd1
Latent	1.310161591	0.029898839	DBH-like monooxygenase protein 2	Moxd2
Latent	1.311088753	0.019627297	Protein phosphatase 1 regulatory inhibitor subunit 16B	Ppp1r16b
Latent	1.312641907	0.02429574	Iron-sulfur cluster assembly 1 homolog, mitochondrial	Isca1;AK157302
Latent	1.313829803	0.000232332	FACT complex subunit SSRP1	Ssrp1
Latent	1.314494133	0.005742526	Rho GTPase-activating protein 5	Arhgap5
Latent	1.315075684	1.31846E-08	EMILIN-1	Emilin1
Latent	1.315112877	0.000113334	Podocalyxin	Podxl
Latent	1.315283775	0.048514655	Disintegrin and metalloproteinase domain-containing protein 7	Adam7
Latent	1.315815735	0.02760273	demilune cell and parotid protein	Dcpp1;Dcpp3;Dcpp2
Latent	1.315895081	0.014786115	Tetraspanin;Tetraspanin-7	Tspan7
Latent	1.316650963	0.043388423	Zinc finger protein 786	Znf786
Latent	1.31690979	0.000313826	Retinol dehydrogenase 12	Rdh12
Latent	1.317401314	0.000609977	Histone deacetylase 7;Histone deacetylase;histone deacetylase	Hdac7
Latent	1.318619156	0.046537905	Zinc finger protein 42	Zfp42
Latent	1.319688225	5.23982E-05	DNA-directed RNA polymerase I subunit RPA43	Polr1f
Latent	1.321097183	0.006183162	Coiled-coil domain-containing protein 116	Ccdc116
Latent	1.321898651	0.00524217	Collagen, type IV, alpha 5	Col4a5
Latent	1.328923607	0.039649682	SURP and G-patch domain-containing protein 1	Sugp1
Latent	1.331869888	1.46094E-05	Histone H1.0;Histone H1.0, N-terminally processed	H1-0
Latent	1.332895851	1.97121E-06	Integrin alpha-8;Integrin alpha-8 heavy chain;Integrin alpha-8 light chain	Itga8
Latent	1.336946297	0.049326581	Protein capicua homolog	Cic
Latent	1.339025497	0.049303021	Interferon-induced GTP-binding protein Mx2	Mx2
Latent	1.340410614	0.001845777	RING finger protein unkempt homolog	Unk
Latent	1.341241074	6.8501E-06	Guanine nucleotide-binding protein G(o) subunit alpha	Gnao1
Latent	1.341755104	0.004683745	Tetraspanin-8	Tspan8
Latent	1.342035675	0.026508466	Metabotropic glutamate receptor 2	Grm2
Latent	1.342910385	0.000152412	Golgi-associated RAB2 interactor protein 2	Garin2
Latent	1.343324852	0.030276325	Semaphorin-6C	Sema6c
Latent	1.343610764	3.56398E-05	Kelch-like 34	Klhl34
Latent	1.343654633	0.052967263	Inhibitor of nuclear factor kappa-B kinase-interacting protein	Ikbip
Latent	1.344581413	5.05507E-08	Latent-transforming growth factor beta-binding protein 4	Ltbp4
Latent	1.345141983	0.014570017	Keratin 78	Krt78
Latent	1.346707916	0.075394681	PHD finger protein 12	Phf12
Latent	1.347036171	1.42088E-05	Transmembrane protein 201	Tmem201
Latent	1.347616196	0.00041637	Ribosomal protein S6 kinase beta-2;non-specific serine/threonine protein kinase	Rps6kb2
Latent	1.348176575	0.044319768	Cell division cycle-associated protein 3	Cdca3
Latent	1.348785591	0.014455132	Myosin XVB	Myo15b
Latent	1.3539217	0.000104335	4-hydroxybenzoate polyprenyltransferase, mitochondrial	Coq2
Latent	1.354134178	0.007652979	UPF0415 protein C7orf25 homolog	
Latent	1.356364822	0.000104947	Myocyte-specific enhancer factor 2B	Mef2b
Latent	1.357200623	0.006402308	Keratin, type II cytoskeletal 1b	Krt77
Latent	1.357546043	2.76962E-05	Lamin-B2	Lmnb2
Latent	1.359191895	0.008408936	Protein downstream neighbor of Son	Donson
Latent	1.359452629	4.26591E-05	Prelamin-A/C;Lamin-A/C	Lmna
Latent	1.360417366	0.002092189	Calcyphosin-2	Caps2
Latent	1.360930252	0.049564646	Embryonal Fyn-associated substrate	Efs
Latent	1.361766815	0.002893821	Ceramide kinase	Cerk
Latent	1.361841583	0.002318139	Protein O-GlcNAcase	Oga

Latent	1.362113762	0.008571506	E3 ubiquitin-protein ligase HECW2	Hecw2
Latent	1.362674904	4.10445E-05	Mast cell protease 4	Mcpt4
Latent	1.363178062	0.002446381	Lipase maturation factor 2	Lmf2
Latent	1.363265991	0.001184477	Doublesex- and mab-3-related transcription factor A1	Dmrta1
Latent	1.363314438	0.007269153	Polyhomeotic-like protein 1	Phc1
Latent	1.36518116	0.078676808	SH3 domain-containing protein 21	Sh3d21
Latent	1.373672104	0.014907312	Growth arrest-specific protein 6	Gas6
Latent	1.376455879	0.05216726	Alpha-catulin	Ctnnal1
Latent	1.378120804	0.042408793	Transcriptional enhancer factor TEF-3	Tead4
Latent	1.379245758	0.00040736	WD repeat-containing protein 73	Wdr73
Latent	1.37970562	0.018401839	Enoyl-CoA hydratase domain-containing protein 3, mitochondrial	Echdc3
Latent	1.381550789	0.006496864	Pecanex-like protein 3	Pcnx3
Latent	1.383432961	2.1082E-07	Laminin subunit beta-1	Lamb1
Latent	1.383775902	0.001030852	Zinc finger protein 319	Znf319
Latent	1.384355927	2.43997E-05	Zinc finger protein 182	Znf182;Zfp182
Latent	1.384563828	0.006130414	Ubiquinone biosynthesis O-methyltransferase, mitochondrial	Coq3
Latent	1.384591293	0.002736973	Zinc finger BED domain-containing protein 6	Zbed6
Latent	1.388765717	0.073672606	ATP-dependent DNA helicase Q5	Recql5
Latent	1.389770508	0.015318253	Metal transporter CNNM2	Cnnm2
Latent	1.392347527	0.055068973	Large ribosomal subunit protein mL64	Gadd45gip1
Latent	1.392390442	0.041767847	Predicted pseudogene 5478	Gm5478
Latent	1.394058609	0.000905566	Ankyrin repeat domain-containing protein SOWAHA	Sowaha
Latent	1.394695663	0.052267132	KAT8 regulatory NSL complex subunit 1-like protein	Kansl1l
Latent	1.395645714	0.010195397	Disks large-associated protein 5	Dlgap5
Latent	1.396417809	0.001779315	Estradiol 17 beta-dehydrogenase 5;Aldo-keto reductase family 1 member C18;Aldo-keto reductase family 1 member C21	Akr1c6;Akr1c18;Akr1cl;Akr1c21
Latent	1.399967575	0.01886768	Growth/differentiation factor 5	Gdf5
Latent	1.400933838	7.4996E-05	Peroxisome proliferator-activated receptor gamma coactivator 1-alpha	Ppargc1a
Latent	1.401692581	0.005490071	Teneurin-3	Tenm3
Latent	1.403987694	0.001333715	ATP-dependent RNA helicase DDX50	Ddx50
Latent	1.406878471	6.94638E-06	Zinc finger protein 22	Znf22
Latent	1.407269287	0.000251039	Ankyrin repeat and BTB/POZ domain-containing protein 1	Abtb1
Latent	1.40747242	0.027943056	Fucose-1-phosphate guanylyltransferase	Fpgt
Latent	1.409959602	0.039200501	Myosin-3	Myh3
Latent	1.414293289	0.055970361	Calmodulin-binding transcription activator 1	Camta1
Latent	1.414687347	0.016143753	Fibulin-1	Fbln1
Latent	1.415208244	0.001531164	Carbohydrate sulfotransferase	Chst13
Latent	1.415545845	0.080540198	Vomeronasal 2 receptor 111/112	Vmn2r111;Vmn2r112
Latent	1.416184425	0.000992483	CUB domain-containing protein 1	Cdcp1
Latent	1.416519165	0.004507585	Spermatogenesis associated glutamate (E)-rich protein 4A	Gm1979;Speer4a3;Speer4a2
Latent	1.417967415	0.003592787	Trinucleotide repeat-containing gene 18 protein	Tnrc18
Latent	1.420541573	0.000876139	Golgi associated RAB2 interactor family member 5B	Garin5b
Latent	1.421409798	0.009213461	Alpha-2A adrenergic receptor	Adra2a
Latent	1.421818542	2.69593E-07	Collagen alpha-2(VI) chain	Col6a2
Latent	1.422884369	0.024550922	Large ribosomal subunit protein mL53	Mrpl53
Latent	1.431332207	0.085600124	Mismatch repair endonuclease PMS2	Pms2
Latent	1.43157711	1.21023E-05	Protein 4.1	Epb41
Latent	1.435104561	0.041741857	5-nucleotidase domain-containing protein 3	Nt5dc3
Latent	1.435619926	0.044645774	Ubiquitin carboxyl-terminal hydrolase 44	Usp44
Latent	1.435738564	2.94668E-05	Cytosolic iron-sulfur assembly component 2B	Ciao2b
Latent	1.436560249	0.046499402	Tetraspanin-9	Tspan9
Latent	1.437463379	0.028357058	N-lysine methyltransferase SETD6	Setd6
Latent	1.440357971	5.06332E-05	Semaphorin-3C	Sema3c
Latent	1.445361328	0.02833897	MORC family CW-type zinc finger protein 3	Morc3
Latent	1.448894501	0.072693267	Epithelial cell-transforming sequence 2 oncogene-like	Ect2l
Latent	1.449805069	0.002591545	Ephrin-B1;Ephrin-B1 C-terminal fragment;Ephrin-B1 intracellular domain	Efnb1
Latent	1.449865341	9.64426E-09	Core histone macro-H2A.2	Macroh2a2
Latent	1.450232124	0.002693875	Ral guanine nucleotide dissociation stimulator-like 1	Rgl1

Latent	1.451376534	0.033156308	Protocadherin beta 22	Pcdhb22
Latent	1.453426933	0.005049745	Alpha-actinin-2	Actn2
Latent	1.454539108	0.008899309	Ankyrin repeat and EF-hand domain-containing protein 1	Ankef1
Latent	1.455308723	0.005123684	Zonadhesin	Zan
Latent	1.456075478	0.071901114	Rhophilin-1	Rhpn1
Latent	1.456212425	0.013759279	Poly(A) RNA polymerase, mitochondrial	Mtpap
Latent	1.456552887	0.000190109	Potassium voltage-gated channel subfamily KQT member 3	Kcnq3
Latent	1.456950569	0.001701704	Cytochrome P450, family 4, subfamily a, polypeptide 29	Cyp4a29
Latent	1.459361839	0.011915592	Phosphatidylinositol glycan anchor biosynthesis class U protein	Pigu
Latent	1.462984276	1.7399E-06	Laminin subunit gamma-1	Lamc1
Latent	1.466135979	0.055572435	Cochlin	Coch
Latent	1.470871925	0.008517292	Coiled-coil domain-containing 170	Ccdc170
Latent	1.472522926	0.083982917	Probable carboxypeptidase X1	Cpxm1
Latent	1.473259926	0.009424908	Transformer-2 protein homolog alpha	Tra2a
Latent	1.474163437	0.000850041	Paraneoplastic antigen Ma3 homolog	Pnma3
Latent	1.475159073	0.114292782	Kinesin-like protein KIF3B;Kinesin-like protein KIF3B, N-terminally processed	Kif3b
Latent	1.477270126	0.062233245	Myotubularin-related protein 6	Mtmr6
Latent	1.478333473	0.020499145	C-type lectin domain family 11 member A	Clec11a
Latent	1.481014252	0.014917368	NEDD4-binding protein 2	N4bp2
Latent	1.482295036	4.91083E-05	Musculoskeletal embryonic nuclear protein 1	Mustn1
Latent	1.483960152	0.044224978	C4b-binding protein	C4bpa
Latent	1.486563301	8.19372E-09	Nephronectin	Npnt
Latent	1.48950901	1.79007E-06	Probable RNA-binding protein 19	Rbm19
Latent	1.490225983	0.001342291	Aggrecan core protein	Acan
Latent	1.490832901	0.002025729	LIX1-like protein	Lix1l
Latent	1.490878868	0.078638348	Nuclear distribution protein nudE-like 1	Ndel1
Latent	1.490944862	0.04152398	RalA-binding protein 1	Ralbp1
Latent	1.491582108	0.001124783	CD81 antigen	Cd81
Latent	1.493716812	0.002727027	Capping protein, Arp2/3 and myosin-I linker protein 2	Carmil2
Latent	1.493740082	4.1407E-07	Constitutive coactivator of peroxisome proliferator-activated receptor gamma	Fam120b
Latent	1.4998106	0.032542074	RING-type E3 ubiquitin transferase	Toporsl
Latent	1.501538467	3.41396E-05	Collagen alpha-3(VI) chain	Col6a3
Latent	1.502210999	0.000703959	Coiled-coil domain-containing protein 91	Ccdc91
Latent	1.503719902	0.002300706	Short stature homeobox protein 2	Shox2
Latent	1.504614258	0.00453751	Collagen alpha-1(X) chain	Col10a1
Latent	1.505456352	0.002394411	Vacuolar protein sorting-associated protein 37A	Vps37a
Latent	1.50553093	0.005473267	eIF-2-alpha kinase GCN2;non-specific serine/threonine protein kinase	Eif2ak4
Latent	1.507085991	7.69723E-08	Core histone macro-H2A.1	Macroh2a1
Latent	1.514003181	0.001051004	Gamma-aminobutyric acid type B receptor subunit 1	Gabbr1
Latent	1.515363884	0.018441721	Cyclic AMP-dependent transcription factor ATF-6 beta;Processed cyclic AMP-dependent transcription factor ATF-6 beta	Atf6b
Latent	1.516472816	7.48082E-09	Myocardial zonula adherens protein	Myzap
Latent	1.519823074	0.077707084	Chromodomain-helicase-DNA-binding protein 9;DNA helicase	Chd9
Latent	1.51997509	0.117982045	Solute carrier family 35 member D3	Slc35d3
Latent	1.527090454	0.102096773	Plasma membrane calcium-transporting ATPase 2;Calcium-transporting ATPase	Atp2b2
Latent	1.527113152	0.000353402	Vitamin K-dependent protein Z	Proz
Latent	1.527223778	0.000266327	Fanconi anemia group I protein homolog	Fanci
Latent	1.527304459	1.47793E-05	Trophoblast glycoprotein	Tpbg
Latent	1.531894493	0.008523698	N-myc proto-oncogene protein	Mycn
Latent	1.532130432	0.006787205	Filaggrin	Flg
Latent	1.532529449	0.036086346	Keratin, type II cytoskeletal 2 oral	Krt76
Latent	1.533582497	0.025048546	Protein OSCP1	Oscp1
Latent	1.534283066	0.015754251	Chromodomain-helicase-DNA-binding protein 1-like	Chd1l
Latent	1.534835815	1.56203E-06	Collagen alpha-2(IV) chain;Canstatin	Col4a2
Latent	1.535803223	2.02506E-05	Protein ELFN1	Elfn1
Latent	1.537335777	0.000111028	Trafficking kinesin-binding protein 1	Trak1
Latent	1.537606812	0.055175054	Lebercilin-like protein	Lca5l
Latent	1.53771553	0.011615741	E3 ubiquitin-protein ligase PPP1R11	Ppp1r11

Latent	1.53959198	0.010014105	Transportin-2	Tnpo2
Latent	1.541124535	0.009293685	Cartilage intermediate layer protein 2;Cartilage intermediate layer protein 2 C1;Cartilage intermediate layer protein 2 C2	Cilp2
Latent	1.541343307	0.007171179	Low-density lipoprotein receptor-related protein 6;Low-density lipoprotein receptor-related protein	Lrp6
Latent	1.543089294	0.028366351	SH2B adapter protein 3	Sh2b3
Latent	1.54603157	0.009000796	Espin-like protein	Espnl
Latent	1.54615345	4.84318E-08	Dedicator of cytokinesis protein 9	Dock9
Latent	1.549338531	0.020096512	Baculoviral IAP repeat-containing protein 2	Birc2
Latent	1.549927139	0.000521517	NHP2-like protein 1;NHP2-like protein 1, N-terminally processed	Snu13
Latent	1.550603676	0.082836085	HEAT repeat-containing protein 6	Heatr6
Latent	1.551038551	0.010254204	Na(+)/H(+) exchange regulatory cofactor NHE-RF3	Pdzk1
Latent	1.551939201	0.010497109	Kinesin-like protein;Kinesin-like protein KIF9	Kif9
Latent	1.557217789	0.007670727	Grainyhead-like protein 1 homolog	Grhl1
Latent	1.560548592	4.99621E-06	Myosin regulatory light chain 2, atrial isoform	Myl7
Latent	1.564465904	0.012477796	Glutathione hydrolase 1 proenzyme;Glutathione hydrolase 1 heavy chain;Glutathione hydrolase 1 light chain	Ggt1
Latent	1.564520264	0.011543276	Coiled-coil domain-containing protein 82	Ccdc82
Latent	1.565426254	0.009267333	Ectonucleotide pyrophosphatase/phosphodiesterase family member 5	Enpp5
Latent	1.565961838	4.04502E-06	Regulator of chromosome condensation	Rcc1
Latent	1.567174149	0.053984184	Protein shortage in chiasmata 1 ortholog	Shoc1
Latent	1.569573212	0.035444443	ADP-ribosylation factor-like protein 6-interacting protein 4	Arl6ip4
Latent	1.573561478	0.011193409	Epithelial splicing regulatory protein 1	Esrp1
Latent	1.576868057	0.006176175	Phosphorylated adapter RNA export protein	Phax
Latent	1.577040482	0.024395798	Cyclin-dependent kinase 4	Cdk4
Latent	1.577243042	0.005675952	Interleukin-36 gamma	Il36g
Latent	1.577939415	0.006472273	Testis-specific serine/threonine-protein kinase 4	Tssk4
Latent	1.579775047	0.042737199	Coiled-coil domain-containing 74A	Ccdc74a
Latent	1.582778931	0.008928557	LON peptidase N-terminal domain and RING finger protein 3	Lonrf3
Latent	1.583831406	0.007780828	Protein IMPACT	Impact
Latent	1.587212753	0.000825116	MAM domain-containing glycosylphosphatidylinositol anchor protein 1	Mdga1
Latent	1.588136673	0.000123426	Disks large homolog 3	Dlg3
Latent	1.588157463	0.087708643	Cotranscriptional regulator ARB2A	Arb2a
Latent	1.58980217	2.93604E-08	Nidogen-2	Nid2
Latent	1.59095726	0.000343354	Solute carrier family 22 member 23	Slc22a23
Latent	1.591023445	0.015823108	Phosphatidylinositol phosphatase PTPRQ	Ptprq
Latent	1.592376328	0.000254033	Unconventional myosin-XVIIIa	Myo18a
Latent	1.594315529	0.002729553	Methyl-CpG-binding domain protein 6	Mbd6
Latent	1.59591713	9.37868E-05	Mimecan	Ogn
Latent	1.596741104	0.063861361	Carcinoembryonic antigen-related cell adhesion molecule 20	Ceacam20
Latent	1.597775459	0.028772078	Ankyrin repeat domain 34A	Ankrd34a
Latent	1.598511696	0.011458774	Dyslexia-associated protein KIAA0319-like protein	Kiaa0319l;AU040320
Latent	1.599847221	0.003344168	Tropomodulin-4	Tmod4
Latent	1.602478218	6.84103E-05	Myosin-6	Myh6
Latent	1.606081772	0.012321244	Mitochondrial import receptor subunit TOM40B	Tomm40l
Latent	1.607208061	0.002573537	Insulin-like growth factor-binding protein 6	Igfbp6
Latent	1.609181976	0.017455585	Importin-8	Ipo8
Latent	1.611925316	0.004486331	Leucine-rich repeat-containing protein 39	Lrrc39
Latent	1.612361145	0.049369693	RIKEN cDNA 4921511C20 gene	4921511C20Rik
Latent	1.615424156	0.095367875	G protein-coupled receptor associated sorting protein 3	Gprasp3;Bhlhb9
Latent	1.616437721	0.049542296	Tyrosine-protein phosphatase non-receptor type 4;protein-tyrosine-phosphatase	Ptpn4
Latent	1.616569901	0.010516735	Cartilage acidic protein 1	Crtac1
Latent	1.616680336	6.09662E-05	Receptor-type tyrosine-protein phosphatase zeta;protein-tyrosine-phosphatase	Ptprz1
Latent	1.620536232	0.010216907	Polycomb group RING finger protein 2	Pcgf2
Latent	1.623505783	1.65354E-08	Carbonic anhydrase 2	Ca2
Latent	1.624059105	0.11532668	Cerebral cavernous malformations 2 protein-like	Ccm2l

Latent	1.625237274	7.65744E-06	Lysophospholipase-like protein 1;palmitoyl-protein hydrolase	Lyplal1
Latent	1.625543785	0.000160541	Guided entry of tail-anchored proteins factor CAMLG	Camlg
Latent	1.629999352	0.083210703	Negative elongation factor A	Nelfa
Latent	1.630973244	0.009604575	Seipin	Bscl2
Latent	1.634315491	0.00041546	Elastin	Eln
Latent	1.637845421	0.066942831	DDB1- and CUL4-associated factor 6	Dcaf6
Latent	1.642382431	0.000161998	Myc target protein 1	Myct1
Latent	1.645010185	0.004832277	Actin-associated protein FAM107A	Fam107a
Latent	1.647887421	9.87989E-05	DNA excision repair protein ERCC-5	Ercc5
Latent	1.652495766	0.000367231	Prostate tumor-overexpressed gene 1 protein homolog	Ptov1
Latent	1.652913666	0.003413716	Anoctamin-3	Ano3
Latent	1.654925728	0.02523419	Piwi-like protein 1	Piwil1
Latent	1.657893753	0.046645658	Ankyrin-repeat and fibronectin type III domain-containing 1	Ankfn1
Latent	1.659468269	0.00081669	Solute carrier organic anion transporter family member;Solute carrier organic anion transporter family member 1A1	Slco1a1
Latent	1.661332512	0.000306536	Claudin-5	Cldn5
Latent	1.671927071	0.000149943	Ankyrin repeat domain 50	Ankrd50
Latent	1.673666	8.294E-06	Aquaporin-1	Aqp1
Latent	1.675600243	0.020218271	NKAP domain-containing 1	Nkapd1
Latent	1.675976181	0.00066281	Pre-mRNA-processing factor 39	Prpf39
Latent	1.676173019	0.023484251	UPF0450 protein C17orf58 homolog	1810010H24Rik
Latent	1.680789375	0.011978031	Ephrin type-B receptor 6	Ephb6
Latent	1.682861328	0.011949273	Lipoxygenase homology domain-containing protein 1	Loxhd1
Latent	1.68288765	3.62557E-05	Tubulointerstitial nephritis antigen-like	Tinagl1
Latent	1.683587074	0.00156994	Suppressor of fused homolog	Sufu
Latent	1.685977745	0.057510497	TNF receptor-associated factor 4	Traf4
Latent	1.688171577	1.26676E-06	Stomatin	Stom
Latent	1.689287376	4.14456E-07	Nidogen-1	Nid1
Latent	1.690071869	0.095244444	Potassium voltage-gated channel subfamily H member 8	Kcnh8
Latent	1.693283653	0.002041158	Pleckstrin homology domain-containing family G member 2	Plekhg2
Latent	1.69753933	3.00703E-09	Laminin subunit alpha-3	Lama3
Latent	1.702627373	0.002418556	UPF0729 protein C18orf32 homolog	
Latent	1.707771111	0.056658981	Cilia- and flagella-associated protein 410	Cfap410
Latent	1.714058304	0.027444164	PH and SEC7 domain-containing protein 1	Psd
Latent	1.714293861	0.017558141	Protocadherin Fat 2	Fat2
Latent	1.719302559	0.032010604	Mitochondrial 10-formyltetrahydrofolate dehydrogenase;formyltetrahydrofolate dehydrogenase	Aldh1l2
Latent	1.724233055	6.28855E-05	Protocadherin Gamma Subfamily B	Pcdhgb6;Pcdhgb7
Latent	1.724263	1.83572E-08	Laminin subunit beta-2	Lamb2
Latent	1.73270607	0.006541978	MAP7 domain-containing protein 2	Map7d2
Latent	1.734251022	0.000111196	Ubiquitin carboxyl-terminal hydrolase MINDY-3;Ubiquitin carboxyl-terminal hydrolase MINDY	Mindy3
Latent	1.736851692	0.000240969	Flavin-containing monooxygenase	Fmo9
Latent	1.739138985	0.000351634	Hyaluronan and proteoglycan link protein 1	Hapln1
Latent	1.739735413	0.002572758	1-acyl-sn-glycerol-3-phosphate acyltransferase epsilon	Agpat5
Latent	1.739900208	1.43346E-08	Laminin subunit alpha-5	Lama5
Latent	1.739938354	0.042962808	Dynein regulatory complex protein 1	Drc1
Latent	1.741130447	0.000203662	Jouberin	Ahi1
Latent	1.742265701	6.99686E-08	Basement membrane-specific heparan sulfate proteoglycan core protein	Hspg2
Latent	1.746251297	1.67722E-08	Spectrin beta chain;Spectrin beta chain, erythrocytic	Sptb
Latent	1.75233078	2.79519E-10	Laminin subunit beta-3	Lamb3
Latent	1.755783272	0.029096003	UDP-glucuronosyltransferase	Ugt2b34
Latent	1.757382774	3.86511E-05	Profilin-2;Profilin	Pfn2
Latent	1.762685776	9.51048E-06	Transcription termination factor 2	Ttf2
Latent	1.76746769	0.004300663	SPATS2-like protein	Spats2l
Latent	1.768142509	0.029354263	Homeobox protein MOX-2	Meox2
Latent	1.772636032	0.0572107	Macrophage-stimulating protein receptor;Macrophage-stimulating protein receptor alpha chain;Macrophage-stimulating protein receptor beta chain	Mst1r
Latent	1.774206734	0.018772762	Rho GTPase-activating protein 28	Arhgap28

Latent	1.777366829	0.028685531	Chondroitin sulfate synthase 1	Chsy1
Latent	1.783746719	0.010497494	Protein FAM3B	Fam3b
Latent	1.793510246	0.002062069	Uncharacterized serine/threonine-protein kinase SBK3	Sbk3
Latent	1.795216942	5.22071E-05	WD repeat domain 49	Wdr49
Latent	1.798822403	0.016695958	Protein Hikeshi	Hikeshi
Latent	1.800599861	0.011219727	Band 4.1-like protein 4A	Epb41l4a
Latent	1.800876236	2.70935E-07	Laminin subunit gamma-2	Lamc2
Latent	1.808944511	0.002611489	Copine-9	Cpne9
Latent	1.80925808	3.5596E-08	Protein 4.2	Epb42
Latent	1.813375664	6.67166E-07	C-C motif chemokine 24	Ccl24
Latent	1.81423645	0.036592471	Zinc finger CCCH-type with G patch domain-containing protein	Zgpat
Latent	1.817768288	0.001312574	Merlin	Nf2
Latent	1.818494606	0.000483526	Ribosome-recycling factor, mitochondrial	Mrrf
Latent	1.820347595	0.000567941	Dehydrogenase/reductase SDR family member 11	Dhrs11
Latent	1.820676613	0.009753043	Oxysterol-binding protein-related protein 3;Oxysterol-binding protein	Osbpl3
Latent	1.827956581	0.158131187	Predicted gene 17349	Gm17349
Latent	1.828887367	0.017685005	Plasminogen activator inhibitor 1	Serpine1
Latent	1.831655502	2.79795E-06	Methyltransferase N6AMT1	N6amt1
Latent	1.837080193	4.0569E-09	Ig kappa chain V-VI region NQ5-78.2.6;Ig kappa chain V-VI region NQ6-8.3.1;Ig kappa chain V-VI region NQ2-48.2.2;Ig kappa chain V-VI region NQ2-17.4.1	Igkv4-70;Igkv4-59
Latent	1.838044357	1.8591E-06	Flavin reductase (NADPH)	Blvrb
Latent	1.838593674	1.51758E-06	Ankyrin-1	Ank1
Latent	1.840476799	0.026392863	AT-rich interactive domain-containing protein 5A	Arid5a
Latent	1.845072937	0.006483008	Maltase-glucoamylase 2, pseudogene	Mgam2-ps
Latent	1.848808479	0.005460638	Kelch-like 33	Klhl33
Latent	1.858300209	0.00323017	Junctophilin-3	Jph3
Latent	1.869714355	0.010923606	Bridge-like lipid transfer protein family member 2	Bltp2
Latent	1.872443581	0.005907666	Glutathione S-transferase 3, mitochondrial	Mgst3
Latent	1.877790642	0.005494711	Solute carrier family 9 member C1	Slc9c1
Latent	1.882609177	0.002296072	Adenylyl cyclase-associated protein 2;Adenylyl cyclase-associated protein	Cap2
Latent	1.883780098	0.001591113	Zinc finger protein 235	Zfp235
Latent	1.884856606	0.010659827	Protocadherin 11 X-linked	Pcdh11x
Latent	1.886100006	0.000104375	Histone H2A.V;Histone H2A.Z;Histone H2A	H2az2;H2az1
Latent	1.887956238	0.000594371	F-box and leucine-rich protein 22	Fbxl22
Latent	1.888074303	0.127132709	Potassium voltage-gated channel subfamily H member 1	Kcnh1
Latent	1.889106941	0.000264958	Interleukin-1 receptor-associated kinase 3	Irak3
Latent	1.891507721	0.005662754	BEN domain-containing protein 5	Bend5
Latent	1.894118309	1.66658E-05	ATP-binding cassette sub-family G member 5	Abcg5
Latent	1.899031639	0.021635687	Ankyrin repeat domain-containing protein SOWAHB	Sowahb
Latent	1.901691628	0.006153837	Tudor domain-containing protein 7	Tdrd7
Latent	1.905590248	0.011556868	Serine/threonine-protein kinase VRK2	Vrk2
Latent	1.918754387	0.001667462	Equilibrative nucleobase transporter 1	Slc43a3
Latent	1.919023132	0.071073023	Zinc finger protein 579	Znf579;Zfp579
Latent	1.928285789	4.49362E-05	NTPase KAP family P-loop domain-containing protein 1	Nkpd1
Latent	1.928996277	2.22647E-08	Spectrin alpha chain, erythrocytic 1	Spta1
Latent	1.931985664	0.000361685	Interferon gamma receptor 1	Ifngr1
Latent	1.934418106	0.020303721	BAI1-associated protein 3	Baiap3
Latent	1.946129799	0.010717487	cAMP-regulated phosphoprotein 19	Arpp19
Latent	1.946631813	1.96582E-05	F-box only protein 31	Fbxo31
Latent	1.953273773	0.019169296	SLIT-ROBO Rho GTPase-activating protein 3	Srgap3
Latent	1.954184914	0.00096112	E3 ubiquitin-protein ligase pellino homolog;E3 ubiquitin-protein ligase pellino homolog 3	Peli3
Latent	1.955487633	2.24962E-06	EMI domain-containing protein 1	Emid1
Latent	1.957034111	0.004310185	Vomeronasal type-1 receptor	Vmn1r199
Latent	1.958749771	0.000253632	Collagen alpha-1(XXIV) chain	Col24a1
Latent	1.966230774	0.016473071	Atlastin-1	Atl1
Latent	1.968610764	0.000324047	Transcription initiation factor TFIID subunit 2	Taf2
Latent	1.970966721	2.06467E-06	Speckle-type POZ protein-like	Spopl
Latent	1.974523735	1.01925E-09	Immunoglobulin kappa variable 6	Igkv6-32
Latent	1.976396179	0.000915895	HAUS augmin-like complex, subunit 6	Haus6

Latent	1.981229019	2.20795E-05	Collagen alpha-2(V) chain	Col5a2
Latent	1.98540287	0.012036673	Insulin-like growth factor-binding protein 2	Igfbp2
Latent	1.993283272	0.004035478	Alpha-tectorin	Tecta
Latent	1.994203949	1.11456E-05	Myosin light chain 4	Myl4
Latent	2.002671242	0.000296624	dTDP-D-glucose 4,6-dehydratase	Tgds
Latent	2.003915977	8.15316E-08	Carbonic anhydrase 1	Ca1
Latent	2.013201904	1.02632E-08	Band 3 anion transport protein	Slc4a1
Latent	2.017071342	0.000145706	Rho GTPase-activating protein 44	Arhgap44
Latent	2.019520569	0.003491116	Myelin protein P0	Mpz
Latent	2.025425529	0.011892678	Serine/threonine-protein kinase LATS2;non-specific serine/threonine protein kinase	Lats2;Gm49361
Latent	2.035579681	2.0603E-05	Alpha-synuclein	Snca
Latent	2.036853409	0.004602085	RNA N6-adenosine-methyltransferase METTL16;U6 small nuclear RNA (adenine-(43)-N(6))-methyltransferase	Mettl16
Latent	2.043773079	5.96099E-05	TSC22 domain family protein 1	Tsc22d1
Latent	2.04700489	0.000296374	Collagen alpha-1(V) chain	Col5a1
Latent	2.050126839	0.00383215	Homeobox protein Hox-A7	Hoxa7
Latent	2.06094265	0.005943656	Protein BNIP5	Bnip5
Latent	2.069117737	1.02185E-10	EKC/KEOPS complex subunit Lage3	Lage3
Latent	2.074674225	0.010264193	N-terminal Xaa-Pro-Lys N-methyltransferase 1;N-terminal Xaa-Pro-Lys N-methyltransferase 1, N-terminally processed;N-terminal Xaa-Pro-Lys N-methyltransferase 1	Ntmt1
Latent	2.082697105	0.004889948	Tubby-like protein;Tubby-related protein 1	Tulp1
Latent	2.088409996	0.000928285	Nuclear receptor subfamily 1 group D member 2	Nr1d2
Latent	2.094228554	0.004830389	DNA-binding protein inhibitor ID-1	Id1
Latent	2.095091248	0.028572795	RIKEN cDNA 4933430I17 gene	4933430I17Rik
Latent	2.102856445	1.0062E-05	Cilia- and flagella- associated protein 210	Cfap210
Latent	2.106055832	7.40115E-05	WW domain-containing oxidoreductase	Wwox
Latent	2.107892609	0.014843933	5 exonuclease Apollo	Dclre1b
Latent	2.108791065	0.000533841	Transmembrane protein 8B	Tmem8b
Latent	2.135118294	3.79876E-05	Transcription factor IIIB 50 kDa subunit	Brf2
Latent	2.148676872	0.000127056	Tubulin--tyrosine ligase	Ttl
Latent	2.160375023	0.000617877	Caveolin-2	Cav2
Latent	2.163150787	0.000812229	Sphingosine 1-phosphate receptor 5	S1pr5
Latent	2.174855995	2.49744E-05	Paired-Ig-like receptor A12	Pira12
Latent	2.183505249	0.000128762	Protein eva-1 homolog B	Eva1b
Latent	2.18476944	0.003428929	Spermatogenesis associated 31 subfamily E member 1	Spata31e1
Latent	2.191817284	6.5264E-05	RNA-binding protein 43	Rbm43
Latent	2.192595863	5.74863E-10	T-cell activation Rho GTPase-activating protein;T-cell activation GTPase-activating protein 1	Tagap;Tagap1
Latent	2.197647095	0.000417578	Proto-oncogene tyrosine-protein kinase ROS;receptor protein-tyrosine kinase	Ros1
Latent	2.197671509	1.45605E-05	Bisphosphoglycerate mutase;Phosphoglycerate mutase	Bpgm
Latent	2.207847977	0.005373173	Fasciculation and elongation protein zeta-2	Fez2
Latent	2.216858101	3.57969E-08	Collagen alpha-2(I) chain	Col1a2
Latent	2.220004082	0.000802364	Nephrocystin-1	Nphp1
Latent	2.260362244	0.000757985	Zinc finger protein 58	Zfp58
Latent	2.270828438	0.024661949	guanylate cyclase	Gucy1b2
Latent	2.272069931	0.221995874	Leiomodin-3	Lmod3
Latent	2.276210022	0.000801284	CCN family member 2	Ccn2
Latent	2.276877022	0.00015253	Prostamide/prostaglandin F synthase	Prxl2b
Latent	2.280726624	7.72314E-05	StAR-related lipid transfer protein 5	Stard5
Latent	2.289172935	0.000422972	Golgi phosphoprotein 3-like	Golph3l
Latent	2.304193878	0.003342934	LINE-1 retrotransposable element ORF2 protein;Reverse transcriptase;Endonuclease	Pol
Latent	2.310611916	0.023682597	Caspase recruitment domain-containing protein 14	Card14
Latent	2.321126556	0.000245229	Voltage-dependent L-type calcium channel subunit alpha;Voltage-dependent L-type calcium channel subunit alpha-1F	Cacna1f
Latent	2.351010513	3.92045E-05	ADP-ribosylation factor-like protein 6	Arl6
Latent	2.353142357	3.73863E-06	Ubiquitin-associated protein 1	Ubap1
Latent	2.360364532	5.94814E-06	Phosphoinositide 3-kinase adapter protein 1	Pik3ap1
Latent	2.365032959	0.008110275	F-box only protein 40	Fbxo40

Latent	2.365545464	0.05535477	Potassium/sodium hyperpolarization-activated cyclic nucleotide-gated channel 3	Hcn3
Latent	2.390465927	2.13904E-07	Collagen alpha-1(I) chain	Col1a1
Latent	2.404984283	0.012702429	Forkhead box protein D4	Foxd4
Latent	2.417224693	0.004604341	Dachshund homolog 2	Dach2
Latent	2.427598	0.007842715	Toll-like receptor 8	Tlr8
Latent	2.444767952	0.002227496	Cyclin-L1	Ccnl1
Latent	2.467278099	0.004777921	Cyclin-F	Ccnf
Latent	2.467645454	0.001636791	Rab GTPase-activating protein 1-like	Rabgap1l
Latent	2.472881508	0.00075884	Ammonium transporter Rh type A	Rhag
Latent	2.491950607	0.000689655	Patatin-like phospholipase domain-containing protein 2	Pnpla2
Latent	2.496335411	0.002934076	CMRF35-like molecule 1	Cd300lf
Latent	2.540499306	0.014085697	Small ribosomal subunit protein uS2m	Mrps2
Latent	2.56758976	0.00513086	G protein pathway suppressor 2	Gps2
Latent	2.572849274	1.41411E-05	Axonemal dynein light intermediate polypeptide 1	Dnali1
Latent	2.601799774	0.01505991	IQ motif and ankyrin repeat domain-containing protein 1	Iqank1
Latent	2.613819313	0.000157856	Basement membrane-specific heparan sulfate proteoglycan core protein;Endorepellin;LG3 peptide	Hspg2
Latent	2.620173264	0.008497256	Centrosomal protein of 78 kDa	Cep78
Latent	2.718218613	2.15994E-05	Protein Smaug homolog 2	Samd4b
Latent	2.738171768	0.000225363	Platelet glycoprotein Ib beta chain	Gp1bb
Latent	2.743901062	0.016200284	Polyphosphoinositide phosphatase	Fig4
Latent	2.819205666	0.00152613	Hemicentin-2	Hmcn2
Latent	2.820311546	0.000172972	Serum response factor	Srf
Latent	2.824894333	1.18642E-06	H(+)/Cl(-) exchange transporter 5	Clcn5
Latent	2.846100616	0.000236441	Rho GTPase-activating protein 8	Arhgap8
Latent	2.908808327	0.001298853	RILP-like protein 2	Rilpl2
Latent	2.937101746	5.91982E-07	Aquaporin-5	Aqp5
Latent	2.951729012	1.92258E-05	PACRG-like protein	Pacrgl;5730480H06Rik
Latent	2.993377876	0.002341961	Carboxyl-terminal PDZ ligand of neuronal nitric oxide synthase protein	Nos1ap
Latent	3.002774429	1.01439E-05	Coiled-coil domain-containing protein 34	Ccdc34
Latent	3.008416557	9.14835E-10	EEF1A lysine methyltransferase 2	Eef1akmt2
Latent	3.165781403	4.28766E-07	Transmembrane protein 14C	Tmem14c
Latent	3.182744026	0.000324262	AT-rich interactive domain-containing protein 3B;AT-rich interactive domain-containing protein 3	Arid3b
Latent	3.307299995	0.009703569	Speriolin	Spatc1
Latent	3.35314064	3.68101E-06	Syntaxin-19	Stx19
Latent	3.379618263	0.001884114	Cell cycle exit and neuronal differentiation protein 1	Cend1
Latent	3.450105667	1.10106E-06	Tetratricopeptide repeat protein 9C	Ttc9c
Latent	3.757102203	7.22563E-05	Uncharacterized protein C7orf50 homolog	3110082I17Rik
Latent	3.930462837	0.008239579	EH domain-binding protein 1	Ehbp1

Murine Proteins: Typical versus Hypervirulent				
**Enriched in:**	**Fold Change**	**p-value**	**Protein Name**	**Gene**
Hypervirulent	-2.502694554	0.000237766	Aryl hydrocarbon receptor nuclear translocator	Arnt
Hypervirulent	-1.736510468	5.28814E-05	Immunity-related GTPase family M protein 2	Irgm2
Typical	2.215282673	1.77697E-05	Arginase-1	Arg1

Supplemental Table 2: Significant *C. neoformans* culture protein identifications. Cultures of typical (KN99α), latent (UgCl223), and hypervirulent (UgCl422) *C. neoformans* strains were grown and processed for LC-MS/MS proteomics. Culture proteomes were compared to each other and significant proteins identified from Student's t-test statistical analyses. Tables were generated from the identified significant proteins for each comparison.

Cryptococcus neoformans Culture Proteins: Latent versus Typical				
**Enriched in:**	**Fold Change**	**p-value**	**Protein Name**	**Gene**
Typical	-4.312538147	9.69143E-06	Sorbitol dehydrogenase	CNAG_00269
Typical	-4.007034779	0.03654906	Alcohol dehydrogenase	CNAG_00038
Typical	-3.926552773	1.74666E-06	Glutathione peroxidase	CNAG_06287
Typical	-3.302467346	8.39364E-06	COX assembly mitochondrial protein	CNAG_02190
Typical	-3.187716484	0.001224563	WSC domain-containing protein	CNAG_02864
Typical	-3.112656593	0.028115637	IMS import disulfide relay-system CHCH-CHCH-like Cx9C domain-containing protein	CNAG_06344
Typical	-3.106978893	0.009614464	Aromatic amino acid aminotransferase I	CNAG_03808
Typical	-3.051720619	0.008784895	N-acetyltransferase domain-containing protein	CNAG_01267
Typical	-2.922665596	0.000444982	Alternative oxidase, mitochondrial	AOX1
Typical	-2.898115635	0.006591725	Ricin B lectin domain-containing protein	CNAG_00588
Typical	-2.829852104	0.006838671	Cellular nucleic acid-binding protein	CNAG_02338
Typical	-2.799077511	0.000167414	Cystathionine gamma-lyase	CNAG_06448
Typical	-2.77214241	0.036719334	Atypical/ABC1/ABC1-C protein kinase	CNAG_07359
Typical	-2.654384136	0.000202183	2-oxoadipate dioxygenase/decarboxylase	CNAG_06294
Typical	-2.580316067	0.023137286	Rho family protein	CNAG_02883
Typical	-2.520565987	0.000246217	Histone H1	CNAG_02924
Typical	-2.511962891	3.96904E-06	Ribokinase	CNAG_02296
Typical	-2.494310379	0.015738061	Cation diffusion facilitator	CNAG_06317
Typical	-2.458886623	0.002958606	Mediator of RNA polymerase II transcription subunit 11	MED11
Typical	-2.45715332	0.009349034	Nuclear protein	CNAG_02990
Typical	-2.41337347	0.000844138	Cytochrome c oxidase assembly factor 5	CNAG_05287
Typical	-2.40566206	0.009845541	Mitochondrial import inner membrane translocase subunit	CNAG_03301
Typical	-2.387980461	8.42376E-05	Xylitol dehydrogenase	CNAG_07589
Typical	-2.386084557	0.002016877	Galactose-1-phosphate uridylyltransferase	CNAG_03875
Typical	-2.337789059	0.005996403	Non-structural maintenance of chromosomes element 4	CNAG_02491
Typical	-2.256576538	0.003434752	Bola-like protein	CNAG_03927
Typical	-2.235671997	8.44757E-05	aldehyde dehydrogenase (NAD(+))	CNAG_00735
Typical	-2.222736359	0.001751023	phosphopyruvate hydratase	CNAG_06868
Typical	-2.195787907	0.001962901	Ribosomal protein	CNAG_01974
Typical	-2.149485111	0.009438531	IMS import disulfide relay-system CHCH-CHCH-like Cx9C domain-containing protein	CNAG_07999
Typical	-2.125526428	0.000157712	Endoribonuclease L-PSP	CNAG_03084
Typical	-2.045500278	0.017222543	Uncharacterized protein	CNAG_04436
Typical	-2.044955254	0.017766714	Endoribonuclease	CNAG_05831

Typical	-2.038244247	0.007571978	POT family proton-dependent oligopeptide transporter	CNAG_01119
Typical	-1.978486538	0.000305635	Isopenicillin N synthase-like Fe(2+) 2OG dioxygenase domain-containing protein	CNAG_04968
Typical	-1.973416805	0.015463574	Solute carrier family 25, member 33/36	CNAG_01288
Typical	-1.961675644	0.00474769	Copper metallothionein 2	CMT2
Typical	-1.93503046	0.000156444	Isochorismatase hydrolase	CNAG_06935
Typical	-1.933071613	0.013848076	Biotin-[acetyl-CoA-carboxylase] ligase	CNAG_01565
Typical	-1.932109833	0.000379638	Oxo-4-hydroxy-4-carboxy-5-ureidoimidazoline decarboxylase domain-containing protein	CNAG_00639
Typical	-1.87735939	0.000124124	Omega-6 fatty acid desaturase (Delta-12 desaturase)	CNAG_01150
Typical	-1.874978065	0.00498988	Glucosamine 6-phosphate N-acetyltransferase	CNAG_06692
Typical	-1.872030735	0.021190139	CCR4-NOT complex subunit CAF16	CNAG_02059
Typical	-1.866200924	0.00331143	Phosphoglycerate mutase	CNAG_02772
Typical	-1.849063873	0.017846248	ATP-binding cassette, subfamily B (MDR/TAP), member 10	CNAG_03684
Typical	-1.820329189	0.001066882	Agmatinase	CNAG_01231
Typical	-1.814527035	0.020045587	ATP-dependent RNA helicase VAD1	VAD1
Typical	-1.807675838	0.018695485	EVE domain-containing protein	CNAG_07608
Typical	-1.792687416	0.018926166	Endoribonuclease L-PSP	CNAG_06589
Typical	-1.791287422	0.007799812	Uricase	CNAG_04307
Typical	-1.760315895	0.008319326	Ribosomal protein L37	CNAG_03015
Typical	-1.751374245	0.00569174	Fork-head domain-containing protein	CNAG_00558
Typical	-1.744879723	0.013477278	RING-type domain-containing protein	CNAG_06693
Typical	-1.683899403	0.016724122	C2H2-type domain-containing protein	CNAG_03788
Typical	-1.652081966	0.000337713	Alpha-aminoadipic semialdehyde synthase	CNAG_00247
Typical	-1.651376724	0.00027207	BSD domain-containing protein	CNAG_05215
Typical	-1.643749714	0.004868515	Zn(2)-C6 fungal-type domain-containing protein	CNAG_02555
Typical	-1.640759945	0.002531675	COX assembly mitochondrial protein	CNAG_02952
Typical	-1.633944988	0.009539315	Solute carrier family 35, member E1	CNAG_05674
Typical	-1.620560169	0.000574455	Uncharacterized protein	CNAG_03609
Typical	-1.600000381	0.000973856	ATPase GET3	CNAG_01923
Typical	-1.599716663	0.003812023	Lathosterol oxidase	CNAG_00519
Typical	-1.580983639	0.000320314	Large ribosomal subunit protein eL42	CNAG_04884
Typical	-1.579073429	0.010349896	Aryl-alcohol dehydrogenase	CNAG_01952
Typical	-1.558574677	0.004095638	Ceramide very long chain fatty acid hydroxylase	CNAG_02577
Typical	-1.529966354	0.003183212	Cell division control protein 42 homolog	CNAG_05348
Typical	-1.510369301	0.004130882	Short-chain dehydrogenase	CNAG_02752
Typical	-1.506356239	0.000258058	Large subunit ribosomal protein L37a	CNAG_04011
Typical	-1.490386963	0.009802303	Cytochrome c oxidase assembly protein subunit 17	CNAG_05573
Typical	-1.462995052	0.005708999	Transcriptional regulator	CNAG_07797
Typical	-1.456021309	0.009058317	Thiamine-binding protein domain-containing protein	CNAG_06669
Typical	-1.4432621	0.008047949	SH3 domain-containing protein	CNAG_00398
Typical	-1.443193913	0.014820573	Phosphoglycerate mutase	CNAG_05892

Typical	-1.429395199	0.006071013	Transcription initiation factor TFIIH subunit 1	CNAG_01826
Typical	-1.424762726	0.007583479	Phosphatidylinositol glycan, class K	CNAG_06655
Typical	-1.414282799	0.009018301	Uncharacterized protein	CNAG_01585
Typical	-1.403726101	0.001994731	Regucalcin	CNAG_05379
Typical	-1.398816586	0.007034852	Spliceosomal protein DIB1	CNAG_04387
Typical	-1.36852932	0.000928662	Endonuclease/exonuclease/phosphatase	CNAG_01519
Typical	-1.363707066	0.000937121	Cell cycle checkpoint protein	CNAG_04055
Typical	-1.356033325	0.002517764	Large subunit acidic ribosomal protein P1	CNAG_00655
Typical	-1.35004282	0.000474208	Conidiation-specific protein 6	CNAG_03759
Typical	-1.348399639	0.006793717	CTP synthase	CNAG_07502
Typical	-1.337978363	0.00336469	Cyclophilin	CNAG_00135
Typical	-1.335409164	0.006964067	U6 snRNA-associated Sm-like protein LSm7	CNAG_05241
Typical	-1.326539516	0.00963448	SGNH hydrolase-type esterase domain-containing protein	CNAG_01654
Typical	-1.29927206	0.001090177	Methyltransferase domain-containing protein	CNAG_06520
Typical	-1.282008648	0.001676011	Oxidoreductase	CNAG_04112
Typical	-1.271633148	0.007668223	YCII-related domain-containing protein	CNAG_06328
Typical	-1.266978741	0.001394875	Protein translocase SEC61 complex gamma subunit	CNAG_00067
Typical	-1.225590706	0.008761197	Acyl-CoA dehydrogenase	CNAG_04688
Typical	-1.221777916	0.004399945	Acyl-CoA-dependent ceramide synthase	CNAG_02086
Typical	-1.212609291	0.006571132	Maltose O-acetyltransferase	CNAG_01489
Typical	-1.196510315	0.008005142	Aromatic amino acid aminotransferase I	CNAG_03828
Typical	-1.180658817	0.005860942	THO complex subunit 7	CNAG_05776
Typical	-1.17326355	0.008521924	Mitochondrial distribution and morphology protein 10	MDM10
Typical	-1.139549732	0.005102499	Iron-sulfur clusters transporter ATM1, mitochondrial	ATM1
Typical	-1.096096039	0.001128519	Malonic semialdehyde reductase	CNAG_02935
Typical	-1.034795761	0.002875368	Phosphatidylethanolamine N-methyltransferase	CNAG_03139
Typical	-1.022837639	7.37922E-05	Uncharacterized protein	CNAG_04027
Typical	-1.019859791	0.003166721	Carnitine O-acetyltransferase	CNAG_06551
Typical	-0.997647285	0.00431145	Arylformamidase	CNAG_05532
Typical	-0.970471382	0.000809948	Xaa-Pro dipeptidase	CNAG_02327
Typical	-0.93732214	0.001458644	Antiphagocytic protein 1	CNAG_06574
Typical	-0.900063992	0.000335628	Phosphoketolase	CNAG_06923
Typical	-0.899894714	0.001102328	Altered inheritance of mitochondria protein 41	AIM41
Typical	-0.892121792	0.000350701	Voltage-gated potassium channel protein beta-2 subunit	CNAG_04209
Typical	-0.838469505	0.000178117	Uncharacterized protein	CNAG_01281
Typical	-0.688681602	0.000168009	Mitochondrial outer membrane 72K protein	CNAG_01682
Latent	0.76377964	5.40835E-05	Nuclear protein	CNAG_03388
Latent	0.841704369	0.001583788	Cytoplasmic protein	CNAG_01015
Latent	0.873208523	0.00012543	Uncharacterized protein	CNAG_01173
Latent	0.887532234	0.001937522	18S rRNA factor 2	CNAG_06756
Latent	0.888711452	0.000104302	Uncharacterized protein	CNAG_00485
Latent	0.893188	0.001018152	Hydrolase	CNAG_02078

Latent	0.919220924	0.002749635	Glycosyl transferase CAP10 domain-containing protein	CNAG_06994
Latent	0.919771671	0.001479519	Structural maintenance of chromosomes protein	CNAG_03767
Latent	1.015497684	0.001869656	Glutathione S-transferase Gst3	CNAG_01893
Latent	1.02276659	0.001093336	Anon-23da protein	CNAG_06922
Latent	1.036419868	0.004660187	non-specific serine/threonine protein kinase	CNAG_05558
Latent	1.059763432	1.7961E-05	Beta-fructofuranosidase	CNAG_06924
Latent	1.060044765	0.000158909	ATP-dependent DNA helicase II subunit 2	CNAG_03637
Latent	1.078996181	0.000791756	Aromatic-L-amino-acid decarboxylase	CNAG_00966
Latent	1.091556072	0.002152392	DNA (cytosine-5-)-methyltransferase DMT5	DMT5
Latent	1.099090099	7.53771E-06	Urease accessory protein 2	URE2
Latent	1.100142956	0.001389308	Mitogen-activated protein kinase	CNAG_04282
Latent	1.121604443	0.00171964	Two-component system sensor protein	CNAG_00106
Latent	1.130786419	0.000256752	Hydrolase	CNAG_01584
Latent	1.142872334	0.000115314	Cohesin complex subunit SA-1/2	CNAG_00812
Latent	1.154268742	0.00016677	cAMP-independent regulatory protein	CNAG_01983
Latent	1.195250034	0.004273397	Trafficking protein particle complex subunit 2	CNAG_04814
Latent	1.219392776	0.009193541	Zn(2)-C6 fungal-type domain-containing protein	CNAG_04473
Latent	1.234344959	0.006476337	NAD(P)-binding domain-containing protein	CNAG_00932
Latent	1.237404346	0.007996912	DNA-directed RNA polymerase II subunit RPB4	CNAG_01444
Latent	1.273220539	0.003303149	Fructose-bisphosphate aldolase	CNAG_01675
Latent	1.291388988	0.010530685	Palmitoyl-protein thioesterase 1	CNAG_00098
Latent	1.294796467	0.000435367	DNA repair protein RAD5	CNAG_00299
Latent	1.368513584	0.012753344	Ribonucleoside-diphosphate reductase subunit M2	CNAG_01915
Latent	1.369181633	0.00750274	Calcineurin-like phosphoesterase domain-containing protein	CNAG_04782
Latent	1.374690056	0.001081125	Cation diffusion facilitator 1	CNAG_04338
Latent	1.37628746	0.008653438	Solute carrier family 25 (Peroxisomal adenine nucleotide transporter), member 17	CNAG_05178
Latent	1.381114006	0.00027367	non-specific serine/threonine protein kinase	CNAG_03167
Latent	1.383724689	0.001654119	Protein bir1	CNAG_04708
Latent	1.401754379	0.005944123	N-acetyltransferase domain-containing protein	CNAG_02047
Latent	1.416965961	0.006590562	NADH dehydrogenase [ubiquinone] 1 beta subcomplex subunit 11, mitochondrial	CNAG_07326
Latent	1.421800613	0.013462448	Histone-lysine N-methyltransferase, H3 lysine-36 specific	CNAG_03188
Latent	1.431535244	0.002175063	Esterase	CNAG_06560
Latent	1.433978558	0.013914284	Uncharacterized protein	CNAG_01065
Latent	1.442046165	0.004429241	Lysophospholipase	CNAG_03786
Latent	1.471574783	0.001091873	BZIP domain-containing protein	CNAG_02589
Latent	1.473927975	0.010081703	Uncharacterized protein	CNAG_04612
Latent	1.483043671	0.010206026	DNAJ domain-containing protein	CNAG_03487
Latent	1.487319469	0.004958216	Uncharacterized protein	CNAG_06573
Latent	1.488704205	0.004806497	Rab-GAP TBC domain-containing protein	CNAG_05039
Latent	1.491259575	0.015810253	Nucleoside-diphosphate-sugar epimerase	CNAG_00831
Latent	1.492451668	0.003220978	glucan endo-1,3-beta-D-glucosidase	CNAG_06336

Latent	1.495985985	0.011733489	Ceramide glucosyltransferase	GCS1
Latent	1.517688274	0.008734884	Replication factor A2	CNAG_01316
Latent	1.52050209	0.001545354	Thioredoxin reductase	CNAG_05847
Latent	1.523076057	0.003869164	SANT domain-containing protein	CNAG_00051
Latent	1.557003975	3.91513E-06	Replication protein A subunit	CNAG_01144
Latent	1.569159508	0.008993538	Pentatricopeptide repeat domain-containing protein	CNAG_01038
Latent	1.572219372	0.000316635	Peptidase S9 prolyl oligopeptidase catalytic domain-containing protein	CNAG_07674
Latent	1.577195644	0.016553709	IQ domain-containing calmodulin-binding protein	CNAG_01602
Latent	1.585292816	0.000547082	TatD DNase family Scn1	CNAG_02543
Latent	1.589644432	0.000658093	SUN domain-containing protein	CNAG_03781
Latent	1.593193531	0.015069399	Nitrogen permease regulator 3	CNAG_05919
Latent	1.596487999	0.004535496	hydroxymethylglutaryl-CoA lyase	CNAG_03067
Latent	1.62177515	0.005873099	protein-L-isoaspartate(D-aspartate) O-methyltransferase	CNAG_03837
Latent	1.639386177	0.014162234	Uncharacterized protein	CNAG_07498
Latent	1.647285461	0.002357408	Short-chain dehydrogenase/reductase SDR	CNAG_03754
Latent	1.658857346	0.003201347	DNA repair protein RAD51 homolog	CNAG_00720
Latent	1.678166389	0.00280781	alpha-glucosidase	CNAG_05471
Latent	1.77010107	0.016216439	Uncharacterized protein	CNAG_00391
Latent	1.775164127	0.018171104	BTB domain-containing protein	CNAG_02716
Latent	1.781851768	0.006280789	BolA protein	CNAG_03352
Latent	1.816999435	0.022782815	Uncharacterized protein	CNAG_00096
Latent	1.8197999	0.004863207	Uncharacterized protein	CNAG_07703
Latent	1.869307995	0.004606824	GTPase	CNAG_05058
Latent	1.875487804	0.01378811	Uncharacterized protein	CNAG_04030
Latent	1.902561188	0.014476585	Uncharacterized protein	CNAG_01077
Latent	1.93462944	0.018424649	Uncharacterized protein	CNAG_03480
Latent	1.939462185	0.007347308	Uncharacterized protein	CNAG_00667
Latent	1.955485821	0.003921508	Aconitate hydratase, mitochondrial	CNAG_07908
Latent	1.968052864	0.00124394	Gfd2/YDR514C-like C-terminal domain-containing protein	CNAG_05187
Latent	2.001200676	0.001917659	Transmembrane receptor	CNAG_03524
Latent	2.045231342	0.016958593	DNA repair protein RAD16	CNAG_02512
Latent	2.097401142	0.02569062	Glucan endo-1,3-alpha-glucosidase agn1	CNAG_02850
Latent	2.13171339	0.010297317	Carboxypeptidase	CNAG_02966
Latent	2.141479015	0.000930052	Aldehyde dehydrogenase (NAD)	CNAG_01078
Latent	2.143662453	0.00052424	Alginate lyase	CNAG_03413
Latent	2.158965588	0.00219638	Glucose-methanol-choline oxidoreductase	CNAG_05258
Latent	2.183533669	0.007921598	Deacetylase	CNAG_06291
Latent	2.205523014	0.005380309	Phosphoglycerate mutase	CNAG_01346
Latent	2.222082138	0.000194982	C3H1-type domain-containing protein	CNAG_06916
Latent	2.226902485	0.009060441	Chitin synthase 8	CHS8
Latent	2.266595364	0.006014817	Endoplasmic reticulum protein	CNAG_06825
Latent	2.267127514	0.008004356	DNA replication complex GINS protein PSF3	CNAG_04682
Latent	2.277686596	0.007707607	Uncharacterized protein	CNAG_01796

Latent	2.321792603	0.000478004	catalase	CNAG_05256
Latent	2.422729969	4.1105E-05	G2/mitotic-specific cyclin 1/2	CNAG_04575
Latent	2.42787075	1.80405E-05	Sarcosine oxidase	CNAG_02984
Latent	2.430183411	0.002479565	PH domain-containing protein	CNAG_03655
Latent	2.455698967	0.032514303	Chitinase	CNAG_04245
Latent	2.462319851	0.010053139	Carbohydrate kinase PfkB domain-containing protein	CNAG_00414
Latent	2.493137836	0.014649168	non-specific serine/threonine protein kinase	CNAG_06174
Latent	2.560507298	0.024153791	Uncharacterized protein	CNAG_04585
Latent	2.63295126	0.000264807	Transcriptional coactivator p15 (PC4) C-terminal domain-containing protein	CNAG_00133
Latent	2.6960392	0.001094464	Glutathione hydrolase	CNAG_02888
Latent	2.777289867	0.000324025	Uncharacterized protein	CNAG_02713
Latent	2.780612469	0.005272026	Small nuclear ribonucleoprotein Sm D3	CNAG_04050
Latent	2.83627224	0.027541017	Ricin B lectin domain-containing protein	CNAG_04891
Latent	2.866146088	0.012897101	START domain-containing protein	CNAG_05588
Latent	3.067204237	0.003316476	Uncharacterized protein	CNAG_00837
Latent	3.128165245	0.029015221	SCF-associated factor 1	CNAG_07482
Latent	3.141826153	0.003246774	NAD-dependent epimerase/dehydratase domain-containing protein	CNAG_01081
Latent	4.506289005	0.0002412	Aryl-alcohol dehydrogenase	CNAG_06027

Cryptococcus neoformans Culture Proteins: Latent versus Hypervirulent				
**Enriched in:**	**Fold Change**	**p-value**	**Protein Name**	**Gene**
Hypervirulent	-3.411491394	9.49471E-06	Glutathione peroxidase	CNAG_06287
Hypervirulent	-3.346595764	0.00394023	Pericentrin/AKAP-450 centrosomal targeting domain-containing protein	CNAG_06687
Hypervirulent	-2.918278217	0.000149849	HIT-type domain-containing protein	CNAG_05810
Hypervirulent	-2.756605148	0.00127891	GATA type zinc finger protein asd-4	CNAG_00193
Hypervirulent	-2.737813473	0.001241879	Maltose O-acetyltransferase	CNAG_01489
Hypervirulent	-2.5745821	0.002251602	POT family proton-dependent oligopeptide transporter	CNAG_01119
Hypervirulent	-2.573719501	1.61922E-06	Ribokinase	CNAG_02296
Hypervirulent	-2.434117317	0.003451784	phosphopyruvate hydratase	CNAG_06868
Hypervirulent	-2.355042458	0.000229237	Cytochrome c oxidase assembly factor 5	CNAG_05287
Hypervirulent	-2.103336811	0.000105847	Conidiation-specific protein 6	CNAG_03759
Hypervirulent	-2.075523853	0.002080947	Uncharacterized protein	CNAG_05608
Hypervirulent	-1.869448662	0.002750722	Uncharacterized protein	CNAG_01585
Hypervirulent	-1.561032772	0.000579784	Uncharacterized protein	CNAG_06800
Hypervirulent	-1.507640839	0.000355307	BSD domain-containing protein	CNAG_05215
Hypervirulent	-1.391145706	2.61335E-05	DUF6987 domain-containing protein	CNAG_00848
Hypervirulent	-1.283712387	1.43639E-05	Isomerase YbhE	CNAG_02655
Latent	1.133093834	1.77584E-05	Replication protein A subunit	CNAG_01144
Latent	1.155762196	3.02807E-05	S-formylglutathione hydrolase	CNAG_06170
Latent	1.205147743	0.000357245	Urease accessory protein 2	URE2
Latent	1.363685608	9.78703E-05	Cohesin complex subunit SA-1/2	CNAG_00812
Latent	1.521577835	0.00072975	Response regulator receiver protein	CNAG_02446
Latent	1.596176147	0.000221517	Translation initiation factor 5	CNAG_01709
Latent	1.662403584	1.84351E-05	Xylitol dehydrogenase	CNAG_04677
Latent	1.678205013	0.001443459	DNA repair protein RAD51 homolog	CNAG_00720
Latent	1.715657234	4.34559E-05	N-acetyltransferase domain-containing protein	CNAG_02508
Latent	1.802822113	0.002532759	non-specific serine/threonine protein kinase	CNAG_03167
Latent	1.836979866	0.001543495	Malic enzyme	CNAG_06374
Latent	1.890740871	0.00067241	NAD-dependent epimerase/dehydratase domain-containing protein	CNAG_01081
Latent	1.896499157	0.001776051	Membrane transporter	CNAG_06569
Latent	1.909658909	0.00220545	Gly-Xaa carboxypeptidase	CNAG_03960
Latent	1.98691082	0.0011637	hydroxymethylglutaryl-CoA lyase	CNAG_03067
Latent	2.055724621	0.003667435	Transcriptional regulator Medusa	CNAG_03859
Latent	2.099789619	0.002983787	VOC domain-containing protein	CNAG_02043
Latent	2.143832207	2.78747E-05	Beta-fructofuranosidase	CNAG_06924
Latent	2.264534473	0.001407209	Enoyl-CoA hydratase/isomerase	CNAG_03010
Latent	2.604475975	0.00156109	Glutathione hydrolase	CNAG_02888
Latent	2.794770718	0.00607084	alpha-glucosidase	CNAG_05471
Latent	3.344613075	0.006765573	Nuclear protein	CNAG_07545

Cryptococcus neoformans Culture Proteins: Typical versus Hypervirulent				
**Enriched in:**	**Fold Change**	**p-value**	**Protein Name**	**Gene**
Hypervirulent	-3.72058773	4.30154E-05	catalase	CNAG_05256
Hypervirulent	-3.298488617	0.000225898	Acetyltransferase	CNAG_02988
Hypervirulent	-3.254861832	0.000298442	Glucose-methanol-choline oxidoreductase	CNAG_05258
Hypervirulent	-2.938158512	0.002756499	Uncharacterized protein	CNAG_03566
Hypervirulent	-2.860544682	8.17826E-05	Short-chain dehydrogenase/reductase SDR	CNAG_03754
Hypervirulent	-2.671867371	0.00942691	Pericentrin/AKAP-450 centrosomal targeting domain-containing protein	CNAG_06687
Hypervirulent	-2.519280434	0.03651051	HIT-type domain-containing protein	CNAG_05810
Hypervirulent	-2.438562393	0.000162497	Small nuclear ribonucleoprotein B and B	CNAG_04840
Hypervirulent	-2.38851738	0.000444935	Phosphoglycerate mutase	CNAG_01346
Hypervirulent	-2.346815109	0.000399306	Opsin 1	CNAG_03572
Hypervirulent	-2.257540703	0.003073609	Uncharacterized protein	CNAG_02911
Hypervirulent	-2.25572443	0.023953796	Small subunit ribosomal protein S13	CNAG_05110
Hypervirulent	-2.236570835	0.002506308	Leucine carboxyl methyltransferase 1	CNAG_03994
Hypervirulent	-2.235795975	0.00683342	Deacetylase	CNAG_06291
Hypervirulent	-2.215668201	0.001389231	Mannose-6-phosphate isomerase	CNAG_04744
Hypervirulent	-2.187374592	0.015502118	Flavin reductase like domain-containing protein	CNAG_05705
Hypervirulent	-2.069476604	9.1358E-05	Isochorismatase-like domain-containing protein	CNAG_02439
Hypervirulent	-2.058523178	0.001026175	Uncharacterized protein	CNAG_02713
Hypervirulent	-2.029839039	0.028864081	Uncharacterized protein	CNAG_07889
Hypervirulent	-2.009722233	0.039679509	DH domain-containing protein	CNAG_00547
Hypervirulent	-1.911946297	0.000263558	Uncharacterized protein	CNAG_03416
Hypervirulent	-1.872119427	0.01521109	WD40 repeat-like protein	CNAG_05816
Hypervirulent	-1.864802361	0.017593991	Uncharacterized protein	CNAG_04479
Hypervirulent	-1.860921383	0.011002988	Mitochondrial GTPase 1	CNAG_07537
Hypervirulent	-1.831076622	0.003089396	Uncharacterized protein	CNAG_07478
Hypervirulent	-1.796122074	0.023419337	Uncharacterized protein	CNAG_06090
Hypervirulent	-1.780678272	0.005400095	Chlorophyll synthesis pathway protein BchC	CNAG_01558
Hypervirulent	-1.775261879	0.000310787	Aldehyde dehydrogenase (NAD)	CNAG_01078
Hypervirulent	-1.765641212	0.012201356	G2/mitotic-specific cyclin 1/2	CNAG_04575
Hypervirulent	-1.758010864	0.03069568	Aldo-keto reductase	CNAG_01615
Hypervirulent	-1.745722294	0.001351579	AAT family amino acid transporter	CNAG_07902
Hypervirulent	-1.731251717	0.000935519	Lysophospholipase	CNAG_03786
Hypervirulent	-1.714028358	0.000879977	protein-ribulosamine 3-kinase	CNAG_02542
Hypervirulent	-1.709042549	0.006117953	AP endonuclease 1	CNAG_05468
Hypervirulent	-1.705093384	0.019675754	Actin-like protein ARP6	CNAG_04650
Hypervirulent	-1.692988873	7.81486E-05	Aromatic-L-amino-acid decarboxylase	CNAG_00966
Hypervirulent	-1.684109211	0.00035861	Uncharacterized protein	CNAG_06800
Hypervirulent	-1.644856453	0.002783227	Carboxypeptidase	CNAG_02966
Hypervirulent	-1.635536194	0.006527939	Pyridoxamine 5'-phosphate oxidase Alr4036 family FMN-binding domain-containing protein	CNAG_04750
Hypervirulent	-1.622136593	0.00493351	Nitrogen permease regulator 3	CNAG_05919
Hypervirulent	-1.617415905	0.007661271	protein-L-isoaspartate(D-aspartate) O-methyltransferase	CNAG_03837

Hypervirulent	-1.601079464	0.00046713	Sarcosine oxidase	CNAG_02984
Hypervirulent	-1.589001179	0.016192646	Phospholipid/glycerol acyltransferase domain-containing protein	CNAG_04522
Hypervirulent	-1.585987091	0.00230524	SANT domain-containing protein	CNAG_00051
Hypervirulent	-1.583382607	0.013991529	Phospholipase/carboxylesterase/thioesterase domain-containing protein	CNAG_03520
Hypervirulent	-1.580114841	0.003647818	SMP domain-containing protein	CNAG_03873
Hypervirulent	-1.575601578	0.003975841	DNA replication complex GINS protein PSF1	CNAG_03374
Hypervirulent	-1.554771423	8.02046E-05	Transmembrane protein	CNAG_03511
Hypervirulent	-1.553881645	0.001173758	Uncharacterized protein	CNAG_05608
Hypervirulent	-1.534250736	0.00451787	ICE2 family protein	CNAG_00428
Hypervirulent	-1.525204182	0.01606709	Maltose O-acetyltransferase	CNAG_01489
Hypervirulent	-1.510269165	0.027219781	Uncharacterized protein	CNAG_00667
Hypervirulent	-1.507582664	0.00201837	Replication factor A3	CNAG_03813
Hypervirulent	-1.495131493	0.011622375	Delta 8-(E)-sphingolipid desaturase	CNAG_03870
Hypervirulent	-1.472117424	0.010184605	BolA protein	CNAG_03352
Hypervirulent	-1.470354557	0.007434627	Fructose-bisphosphate aldolase	CNAG_01675
Hypervirulent	-1.461906433	0.016098676	SH3 domain-containing protein	CNAG_01728
Hypervirulent	-1.458658218	0.008924828	Mitochondrial import receptor subunit TOM7	CNAG_00307
Hypervirulent	-1.458622932	0.002693283	C4-hydroxylase	CNAG_03834
Hypervirulent	-1.42285347	0.01022766	MFS transporter, SIT family, siderophore-iron:H symporter	CNAG_00815
Hypervirulent	-1.41235733	0.01625582	Tartrate transporter	CNAG_02600
Hypervirulent	-1.400410175	0.004141485	Non-histone chromosomal protein 6	CNAG_06544
Hypervirulent	-1.390759468	0.000910561	Cupin domain-containing protein	CNAG_03082
Hypervirulent	-1.385902405	0.00171601	Uncharacterized protein	CNAG_01052
Hypervirulent	-1.369122028	0.008997671	COPII-coated vesicle component Erv46	CNAG_01637
Hypervirulent	-1.350022316	1.77356E-05	sterol 3beta-glucosyltransferase	CNAG_02834
Hypervirulent	-1.349741459	0.000425554	Delta(3,5)-Delta(2,4)-dienoyl-CoA isomerase	CNAG_01135
Hypervirulent	-1.32182312	2.03533E-05	NAD(P)-binding domain-containing protein	CNAG_00932
Hypervirulent	-1.317676067	0.018180288	Uncharacterized protein	CNAG_07319
Hypervirulent	-1.311576366	0.00030048	Sulfatase-modifying factor enzyme domain-containing protein	CNAG_04737
Hypervirulent	-1.307244778	0.00249913	UBA/TS-N domain-containing protein	CNAG_00426
Hypervirulent	-1.290748596	0.004820599	Large subunit acidic ribosomal protein P2	CNAG_05762
Hypervirulent	-1.272419453	0.004837973	WW domain-containing protein	CNAG_03495
Hypervirulent	-1.266922951	0.006565875	Cytoplasmic protein	CNAG_00605
Hypervirulent	-1.263489723	0.000350376	ATP-binding cassette transporter	CNAG_01575
Hypervirulent	-1.257002354	0.003920175	NAD(P)H:quinone oxidoreductase, type IV	CNAG_03936
Hypervirulent	-1.24254179	0.003780124	Protein YOP1	CNAG_01031
Hypervirulent	-1.231544495	0.009922648	RNA polymerase II holoenzyme cyclin-like subunit	CNAG_00440
Hypervirulent	-1.219637394	1.80794E-05	DUF6987 domain-containing protein	CNAG_00848
Hypervirulent	-1.209997654	0.003674276	Uncharacterized protein	CNAG_00391
Hypervirulent	-1.202834129	0.002021888	DUF1769-domain-containing protein	CNAG_07775
Hypervirulent	-1.195534229	0.016420558	Uncharacterized protein	CNAG_04804

Hypervirulent	-1.163693428	0.000272655	Hydrolase	CNAG_01584
Hypervirulent	-1.153088093	0.001525142	peptide chain release factor N(5)-glutamine methyltransferase	CNAG_01546
Hypervirulent	-1.146548748	0.014485166	Trafficking protein particle complex subunit 2	CNAG_04814
Hypervirulent	-1.118165493	0.002733743	Small ribosomal subunit protein mS29	CNAG_00512
Hypervirulent	-1.109081268	0.011916147	Ubiquitin thioesterase OTU	CNAG_06428
Hypervirulent	-1.103703976	0.008572068	DNA-directed RNA polymerase II subunit RPB4	CNAG_01444
Hypervirulent	-1.078479767	0.000776604	Cytochrome b-c1 complex subunit 2, mitochondrial	QCR2
Hypervirulent	-1.076581001	0.005544531	Cytokinesis protein	CNAG_04815
Hypervirulent	-1.076254845	0.008803134	glutathione transferase	CNAG_04110
Hypervirulent	-1.058906078	0.007807753	Uncharacterized protein	CNAG_06286
Hypervirulent	-1.055213451	0.005864563	Methylglutaconyl-CoA hydratase	CNAG_00371
Hypervirulent	-1.055147171	0.002671774	Mitochondrial protein	CNAG_06668
Hypervirulent	-1.043836117	0.002415965	Uncharacterized protein	CNAG_03884
Hypervirulent	-1.041633606	0.003340457	YTH domain-containing protein	CNAG_07941
Hypervirulent	-1.035693645	0.001099115	Uncharacterized protein	CNAG_02129
Hypervirulent	-1.025003433	0.004502791	Dehydrin	CNAG_06109
Hypervirulent	-1.02043581	0.009388943	Uncharacterized protein	CNAG_02188
Hypervirulent	-1.016454697	0.004062875	Serine/threonine-protein phosphatase 2B catalytic subunit A1	CNA1
Hypervirulent	-1.010968685	0.00043319	Metallochaperone	CNAG_05296
Hypervirulent	-1.009660244	0.005677167	Phosphodiesterase	CNAG_07470
Hypervirulent	-1.001224995	0.001368479	CORD and CS domain-containing protein	CNAG_01553
Hypervirulent	-0.976256847	0.005899631	Fungal specific transcription factor	CNAG_06425
Hypervirulent	-0.970698833	0.003363033	Cullin 1	CNAG_06387
Hypervirulent	-0.968411446	0.004704401	Uncharacterized protein	CNAG_00093
Hypervirulent	-0.962011337	0.004688044	Glycosyl transferase CAP10 domain-containing protein	CNAG_06994
Hypervirulent	-0.960473537	0.004710506	rRNA adenine N(6)-methyltransferase	CNAG_04571
Hypervirulent	-0.941293716	0.004879175	Uncharacterized protein	CNAG_01161
Hypervirulent	-0.935472012	0.001847543	AMME syndrome candidate protein	CNAG_03236
Hypervirulent	-0.924608231	0.001204178	Wiskott-Aldrich syndrome protein	CNAG_02029
Hypervirulent	-0.92165947	0.00018602	BTB domain-containing protein	CNAG_03903
Hypervirulent	-0.912388802	0.000320892	C2 domain-containing protein, variant	CNAG_00522
Hypervirulent	-0.867239475	0.001242879	Ysc84 actin-binding domain-containing protein	CNAG_01344
Hypervirulent	-0.845245838	8.14674E-05	Trehalase	NTH1
Hypervirulent	-0.836765766	0.003188102	Large subunit ribosomal protein L5e	CNAG_02928
Hypervirulent	-0.826424599	0.002687912	Uncharacterized protein	CNAG_03553
Hypervirulent	-0.818863392	0.002432832	Mechanosensitive ion channel protein	CNAG_01704
Hypervirulent	-0.816264629	0.000733417	J domain-containing protein	CNAG_00326
Hypervirulent	-0.807569981	9.83138E-05	Uncharacterized protein	CNAG_05309
Hypervirulent	-0.796611309	6.96663E-05	Calcium uniporter protein, mitochondrial	CNAG_00107
Hypervirulent	-0.794264793	0.000269564	AGC/PKA protein kinase	CNAG_00396
Hypervirulent	-0.791754246	0.00207569	Homoserine dehydrogenase	CNAG_04776
Hypervirulent	-0.783071995	0.00202674	Flavoprotein oxygenase	CNAG_02996

Hypervirulent	-0.781455994	0.000564171	1,4-alpha-glucan-branching enzyme	CNAG_00393
Hypervirulent	-0.768189907	0.000283648	Saccharopine dehydrogenase [NAD(+), L-lysine-forming]	CNAG_06849
Hypervirulent	-0.746879578	0.000594352	Glutamate--cysteine ligase	CNAG_06300
Hypervirulent	-0.733597755	0.000403389	Glutathione S-transferase Gst3	CNAG_01893
Hypervirulent	-0.72964859	0.000201358	Proteophosphoglycan ppg4	CNAG_04163
Hypervirulent	-0.661746502	0.000204797	Phosphotransferase	CNAG_05480
Typical	0.689042568	0.000283935	Protein GET1	GET1
Typical	0.702812672	0.000681307	U3 small nucleolar RNA-associated protein 4	CNAG_06005
Typical	0.768762112	0.00017871	Succinate--CoA ligase [ADP-forming] subunit beta, mitochondrial	CNAG_00747
Typical	0.770492077	0.000733507	DNA replication licensing factor MCM2	CNAG_03341
Typical	0.790429592	0.001069887	Malic enzyme	CNAG_06638
Typical	0.799003124	0.00132999	Carbamoyl phosphate synthase arginine-specific small chain	CNAG_00976
Typical	0.81837368	0.001393207	ubiquitinyl hydrolase 1	CNAG_07333
Typical	0.832175255	0.000245818	Aconitate hydratase, mitochondrial	CNAG_01137
Typical	0.83656168	0.000808894	3-hydroxyanthranilate 3,4-dioxygenase	BNA1
Typical	0.842031479	7.95532E-05	Fumarate reductase	CNAG_07862
Typical	0.881098747	0.003665781	Chromosome transmission fidelity protein 4	CNAG_04662
Typical	0.901006222	0.001315589	Allantoicase	CNAG_01108
Typical	0.914401054	0.001999663	Copper-dependent transcription factor 1	CUF1
Typical	0.936048985	0.001138419	Exosome complex component RRP41	CNAG_07416
Typical	0.951600075	0.003198243	Alpha-1,4 glucan phosphorylase	CNAG_06666
Typical	0.979419708	0.000368443	alanine transaminase	CNAG_03436
Typical	0.993470192	0.007564227	Arylformamidase	CNAG_05532
Typical	1.001342773	0.000814386	40S ribosomal protein S4	CNAG_00640
Typical	1.005271435	0.004156369	Phosphatidylinositol glycan, class K	CNAG_06655
Typical	1.006361008	0.000216863	Uncharacterized protein	CNAG_06743
Typical	1.009620667	0.006582961	L-fuconate dehydratase	CNAG_03599
Typical	1.018402576	0.000442419	Aminomethyltransferase	CNAG_02818
Typical	1.024551392	0.002769183	Succinate--CoA ligase [ADP-forming] subunit alpha, mitochondrial	CNAG_03375
Typical	1.026935101	0.002123512	hydroxyacid-oxoacid transhydrogenase	CNAG_07316
Typical	1.033486843	0.003035736	Origin recognition complex subunit 2	CNAG_07162
Typical	1.036776066	0.008182127	Peptidyl-prolyl cis-trans isomerase	CNAG_03486
Typical	1.0397439	0.00994602	CUE domain-containing protein	CNAG_05533
Typical	1.054973125	0.009147873	Membrane fraction protein	CNAG_05154
Typical	1.076760769	0.000539789	DUF221-domain-containing protein	CNAG_06060
Typical	1.079614639	0.000332214	2,4-dienoyl-CoA reductase	CNAG_04228
Typical	1.082589149	0.001265319	AMP-binding protein	CNAG_06433
Typical	1.084068775	0.001085849	Beta-fructofuranosidase	CNAG_06924
Typical	1.095612526	0.000677298	37S ribosomal protein mrp10, mitochondrial	CNAG_00538
Typical	1.097173691	0.004373622	Regucalcin	CNAG_05379
Typical	1.099066734	0.000456555	Lathosterol oxidase	CNAG_00519
Typical	1.100827694	0.010524936	Glutamine amidotransferase	CNAG_03743

Typical	1.100837231	0.00362258	Mitochondrial protein	CNAG_04757
Typical	1.103437424	0.002343722	N-acetyltransferase domain-containing protein	CNAG_02508
Typical	1.113362312	0.011000706	Large subunit ribosomal protein L31e	CNAG_00703
Typical	1.113995075	0.010878133	Fe2OG dioxygenase domain-containing protein	CNAG_02979
Typical	1.11627388	0.001678416	Deoxyuridine 5'-triphosphate nucleotidohydrolase	CNAG_06141
Typical	1.11931324	0.009640766	Phosphatidylinositol transfer protein SFH5	CNAG_02104
Typical	1.143451214	0.000993904	Signal sequence binding protein	CNAG_02223
Typical	1.147827148	0.009323732	Uncharacterized protein	CNAG_06565
Typical	1.152094841	0.005332507	Ubiquinone biosynthesis O-methyltransferase, mitochondrial	COQ3
Typical	1.157171726	0.003941136	Golgi-body localization protein domain-containing protein	CNAG_01115
Typical	1.173897743	0.001718491	Glycine cleavage system P protein	CNAG_01594
Typical	1.178737164	0.006451454	Uncharacterized protein	CNAG_01562
Typical	1.196866035	0.003399061	Response regulator receiver protein	CNAG_02446
Typical	1.197406769	0.006091274	Phosphoglycerate mutase	CNAG_02772
Typical	1.202408314	0.009491592	Rho GDP-dissociation inhibitor	CNAG_03316
Typical	1.204046726	0.003345565	Gly-Xaa carboxypeptidase	CNAG_03420
Typical	1.212896824	0.012460502	DNA ligase 1	CNAG_00566
Typical	1.215657234	0.015528947	NADH dehydrogenase (Ubiquinone) 1 beta subcomplex 3	CNAG_04290
Typical	1.247264385	0.004572809	C2H2-type domain-containing protein	CNAG_04790
Typical	1.26763773	0.01423015	Uncharacterized protein	CNAG_03755
Typical	1.267842293	0.013748615	Mediator of RNA polymerase II transcription subunit 21	CNAG_05405
Typical	1.277204037	0.008514451	Nuclear movement protein nudC	CNAG_06224
Typical	1.278122902	0.016083569	Membrane transporter	CNAG_06569
Typical	1.300361633	0.020475425	Pre-rRNA-processing protein TSR2	CNAG_03293
Typical	1.306698799	0.007133953	Myosin regulatory light chain cdc4	CNAG_00808
Typical	1.308789253	0.017478635	Elongation of fatty acids protein	CNAG_03080
Typical	1.308817863	0.004959847	YCII-related domain-containing protein	CNAG_06328
Typical	1.310417652	0.015973168	Condensin complex subunit 1	CNAG_01959
Typical	1.312675476	0.002705091	Uncharacterized protein	CNAG_03558
Typical	1.320485592	0.007984797	Beta-hexosaminidase	CNAG_06659
Typical	1.329162598	0.001756502	Gluconolactonase	CNAG_04753
Typical	1.3335042	0.007516364	COP9 signalosome complex subunit 7	CNAG_04177
Typical	1.352604866	0.005837531	Protein farnesyltransferase/geranylgeranyltransferase type-1 subunit alpha	CNAG_02229
Typical	1.356976509	0.003623425	Zinc-finger domain-containing protein	CNAG_02699
Typical	1.365894318	0.009700688	Mitochondrial import inner membrane translocase subunit Tim21	CNAG_01569
Typical	1.380989552	0.007070745	Mediator of RNA polymerase II transcription subunit 4	MED4
Typical	1.384386539	0.007072479	UDP-N-acetylglucosamine transferase subunit ALG13	ALG13

Typical	1.403018951	0.010934952	Alpha-glucosidase	CNAG_05913
Typical	1.40538168	0.000397334	Thiol-specific antioxidant protein 3	CNAG_06917
Typical	1.409474373	0.002887619	Large ribosomal subunit protein bL27m	CNAG_00722
Typical	1.414524078	0.007232124	Conserved oligomeric Golgi complex subunit 6	CNAG_05385
Typical	1.419939995	0.000150159	Sugar phosphate phosphatase	CNAG_06566
Typical	1.427583694	0.015225888	Atypical/ABC1/ABC1-B protein kinase	CNAG_04843
Typical	1.438755989	1.47365E-05	Nuclear protein	CNAG_02990
Typical	1.440370083	0.012374624	Chaperone regulator	CNAG_03016
Typical	1.446510315	0.014247705	Uncharacterized protein	CNAG_05595
Typical	1.446712494	0.005906779	Transcription factor C subunit 6	CNAG_04172
Typical	1.449971199	0.000310182	Translation initiation factor 5	CNAG_01709
Typical	1.480809689	0.00025994	Acyl-CoA dehydrogenase	CNAG_03666
Typical	1.485401154	0.009716701	Ribonuclease H1 N-terminal domain-containing protein	CNAG_08025
Typical	1.488996506	3.25025E-05	Malonic semialdehyde reductase	CNAG_02935
Typical	1.489038467	0.003328045	Acid phosphatase	CNAG_02944
Typical	1.49590826	0.002451699	Metallo-beta-lactamase domain-containing protein	CNAG_03361
Typical	1.508872986	0.002408746	Antiphagocytic protein 1	CNAG_06574
Typical	1.520533085	0.013387821	Bud site selection protein	CNAG_00259
Typical	1.529916286	0.000882888	Auxin-induced protein	CNAG_00161
Typical	1.536702633	0.000219471	Agmatinase	CNAG_01231
Typical	1.54416275	0.011860976	Carbonic anhydrase	CNAG_05144
Typical	1.549636841	0.025757777	HIT-type domain-containing protein	CNAG_01124
Typical	1.553294659	0.016797628	Xylitol dehydrogenase	CNAG_07589
Typical	1.556181431	0.001919243	DUF803-domain-containing protein	CNAG_03135
Typical	1.607251167	0.012741008	WD40 repeat-like protein	CNAG_04117
Typical	1.617309093	0.004652213	Omega-6 fatty acid desaturase (Delta-12 desaturase)	CNAG_01150
Typical	1.624578476	0.013190206	Rab family protein	CNAG_02289
Typical	1.643872738	0.000598422	Inositol-3-phosphate synthase	INO1
Typical	1.704008102	0.028605281	Oxidoreductase	CNAG_05299
Typical	1.716406345	0.010444364	Bola-like protein	CNAG_03927
Typical	1.728112698	0.004648725	Methylsterol monooxygenase	CNAG_01737
Typical	1.745360374	4.52652E-06	Alpha-aminoadipic semialdehyde synthase	CNAG_00247
Typical	1.750803471	0.006952042	Cytochrome P450	CNAG_05842
Typical	1.768894196	0.004693428	VOC domain-containing protein	CNAG_02043
Typical	1.771024227	0.008378851	TIGR01458 family HAD hydrolase	CNAG_01604
Typical	1.810103893	0.013339161	Oxo-4-hydroxy-4-carboxy-5-ureidoimidazoline decarboxylase domain-containing protein	CNAG_00639
Typical	1.84175539	0.007625334	Cytochrome c oxidase subunit 1	COI
Typical	1.852476597	0.00375892	Endoplasmic reticulum protein	CNAG_02473
Typical	1.858771801	0.003508737	Peptidase S9 prolyl oligopeptidase catalytic domain-containing protein	CNAG_07337
Typical	1.867171764	0.013699658	Adrenodoxin-type ferredoxin	CNAG_03589
Typical	1.86962986	0.015238582	DNA mismatch repair protein MLH1 (Fragment)	CNAG_02073

Typical	1.876835346	0.036385481	Large subunit ribosomal protein L12	CNAG_01480
Typical	1.889370918	0.0196127	Xylitol dehydrogenase	CNAG_04677
Typical	1.896695614	0.001467503	Alternative oxidase, mitochondrial	AOX1
Typical	1.905426025	0.024827139	Sec14 cytosolic factor	CNAG_00036
Typical	1.928617477	0.01181927	Glucosidase	CNAG_00897
Typical	1.946464539	0.001988652	Cyclin N-terminal domain-containing protein	CNAG_05901
Typical	1.950937271	7.53781E-05	Enoyl-CoA hydratase/isomerase	CNAG_03010
Typical	1.960569382	0.011557638	Chitobiosyldiphosphodolichol beta-mannosyltransferase	CNAG_00689
Typical	1.97209692	0.010822374	Alginate lyase	CNAG_04373
Typical	1.974601269	0.000294613	RP/EB family microtubule-associated protein	CNAG_03993
Typical	2.060267448	1.6773E-06	aldehyde dehydrogenase (NAD(+))	CNAG_00735
Typical	2.11352253	0.000154536	Uncharacterized protein	CNAG_04081
Typical	2.116616249	0.010446245	Uncharacterized protein	CNAG_01574
Typical	2.212471008	0.003513241	Cation diffusion facilitator	CNAG_06317
Typical	2.235118866	0.035673648	MARVEL domain-containing protein	CNAG_01043
Typical	2.27660656	0.001724633	U6 snRNA-associated Sm-like protein LSm3	LSM3
Typical	2.330414772	0.001498154	Uncharacterized protein	CNAG_07556
Typical	2.386911392	0.001112799	Isochorismatase hydrolase	CNAG_06935
Typical	2.449224949	0.000512226	histone acetyltransferase	CNAG_05429
Typical	2.460640907	0.000518349	Transcriptional regulator	CNAG_07797
Typical	2.4845438	0.000868909	2-oxoadipate dioxygenase/decarboxylase	CNAG_06294
Typical	2.535266876	0.047471178	Aromatic amino acid aminotransferase I	CNAG_03808
Typical	2.538495064	0.000279561	Gly-Xaa carboxypeptidase	CNAG_03960
Typical	2.563499928	0.000362314	Retinoic acid induced 16-like protein-domain-containing protein	CNAG_01134
Typical	2.707773209	0.004225321	Holocytochrome c-type synthase	CNAG_00022
Typical	2.953915119	0.005099199	Endoribonuclease L-PSP	CNAG_03084
Typical	2.971222401	0.013908071	Superoxide dismutase [Cu-Zn]	SOD1
Typical	3.017261505	0.000353979	Cell division control protein 24	CNAG_04243
Typical	3.153846264	0.000710323	Endoribonuclease L-PSP	CNAG_06589
Typical	3.266384125	0.009971624	Dihydrolipoamide acetyltransferase component of pyruvate dehydrogenase complex	CNAG_00484
Typical	3.327486038	0.000476766	Endoribonuclease	CNAG_05831
Typical	3.849938393	0.000636861	Transaldolase	CNAG_04025
Typical	3.992593288	5.05714E-05	Sorbitol dehydrogenase	CNAG_00269

Supplemental Table 3: Significant *C. neoformans* infection-derived protein identifications. *C. neoformans* proteome profiles were assessed from the lungs of mice infected with either typical (KN99α), latent (UgCl223), or hypervirulent (UgCl422) strains. Cryptococcus proteomes were then compared to each other and significant proteins identified by Student's t-test. Tables were generated from the identified significant proteins for each comparison.

***Cryptococcus neoformans* Infection Proteins: Latent versus Typical**				
**Enriched in:**	**Fold change**	**p-value**	**Protein Name**	**Gene**
Typical	-2.464521122	8.83184E-08	Elongation factor 2	CNAG_06840
Typical	-2.372786999	0.000158993	Peptide chain release factor 1	CNAG_02948
Typical	-2.049097157	2.21842E-05	S-adenosylmethionine synthase	CNAG_00418
Typical	-1.910790157	0.000119209	Vacuolar protein-sorting-associated protein 4	CNAG_04510
Typical	-1.696996593	1.13369E-05	Elongation factor 1-alpha	CNAG_06125
Typical	-1.512960529	0.010341857	Hsp90-like protein	CNAG_06150
Typical	-1.508752728	0.000225092	Signal recognition particle 54 kDa protein	CNAG_02174
Latent	1.271279621	0.000954698	Aconitate hydratase, mitochondrial	CNAG_01137
Latent	1.280775166	9.21211E-05	NADH-quinone oxidoreductase subunit B 2	CNAG_02266
Latent	1.482935619	0.000802371	Serine/threonine-protein phosphatase	CNAG_03706
Latent	1.528453159	0.001023577	small monomeric GTPase	CNAG_00293
Latent	1.555352306	3.64077E-06	ATP synthase subunit alpha	CNAG_05750
Latent	1.610011959	1.35687E-06	[RNA-polymerase]-subunit kinase	CNAG_06445
Latent	1.69186964	4.43063E-05	ATP synthase subunit beta	CNAG_05918
Latent	1.862039471	0.060095641	Prohibitin	CNAG_00006
Latent	2.132989597	0.027441514	ADP/ATP translocase	CNAG_06101
Latent	2.223504353	0.00188442	H/ACA ribonucleoprotein complex subunit CBF5	CNAG_01168
Latent	2.761834049	3.60127E-06	Histone H2A	CNAG_05221

Cryptococcus neoformans Infection Proteins: Latent versus Hypervirulent				
**Enriched in:**	**Fold Change**	**p-value**	**Protein Name**	**Gene**
Hypervirulent	-2.747316551	1.98962E-05	Peptide chain release factor 1	CNAG_02948
Hypervirulent	-2.365231705	1.39344E-07	Elongation factor 2	CNAG_06840
Hypervirulent	-1.959983253	2.45785E-05	Vacuolar protein-sorting-associated protein 4	CNAG_04510
Hypervirulent	-1.635354614	2.51424E-05	Signal recognition particle 54 kDa protein	CNAG_02174
Hypervirulent	-1.611671638	0.014724752	DNA replication licensing factor MCM3	CNAG_00099
Hypervirulent	-1.462434006	0.005596328	S-adenosylmethionine synthase	CNAG_00418
Hypervirulent	-1.434976959	0.006700812	Hsp90-like protein	CNAG_06150
Hypervirulent	-1.255983162	0.007948456	Elongation factor 1-alpha	CNAG_06125
Latent	1.151808929	0.000133051	Polyubiquitin	CNAG_00370
Latent	1.312606621	0.005184353	Rab family protein	CNAG_04771
Latent	1.345160866	0.001969022	Pre-mRNA-splicing factor RSE1	CNAG_00464
Latent	1.490148354	0.001327003	GTP-binding protein ryh1	CNAG_06049
Latent	1.593759727	0.000339465	Elongation factor Tu	CNAG_03263
Latent	1.616533279	0.000459061	small monomeric GTPase	CNAG_00293
Latent	1.676876068	0.000143881	ATP synthase subunit beta	CNAG_05918
Latent	1.681412125	1.77075E-05	Aconitate hydratase, mitochondrial	CNAG_01137
Latent	1.817332649	1.78783E-06	Calmodulin	CNAG_01557
Latent	1.967961884	0.000890209	Histone H4	CNAG_01648
Latent	1.978735352	0.000717956	Histone H3	CNAG_06745
Latent	2.051222038	0.002395654	H/ACA ribonucleoprotein complex subunit CBF5	CNAG_01168
Latent	2.075635719	2.94432E-06	[RNA-polymerase]-subunit kinase	CNAG_06445
Latent	2.742738914	1.47272E-05	Histone H2A	CNAG_05221
